# Supplementary material for: Formation and degradation of strongly reducing cyanoarene-based radical anions towards efficient radical anion-mediated photoredox catalysis
Source: Nat Commun. 2023 Jan 6;14:92. doi: 10.1038/s41467-022-35774-5 (PMC9822901; doi:10.1038/s41467-022-35774-5)
Supplement: Supplementary file 1 — Supplementary Information [file 41467_2022_35774_MOESM1_ESM.pdf]

Supplementary information

Formation and degradation of strongly reducing cyanoarene-based radical anions towards efficient radical anion-mediated photoredox catalysis

Yonghwan Kwon,[1,2] Jungwook Lee,[1] Yeonjin Noh,[1,2] Doyon Kim,[2] Yungyeong Lee,[1] Changhoon Yu,[1] Juan Carlos Roldao,[3] Siyang Feng,[3] Johannes Gierschner,[3,*] Reinhold Wannemacher,[3,*] Min Sang Kwon[1,*]

[1] Department of Materials Science and Engineering, Seoul National University, Seoul 08826, Republic of Korea;

[2] Department of Materials Science and Engineering, Ulsan National Institute of Science and Technology (UNIST), Ulsan 44919, Republic of Korea;

[3] Madrid Institute for Advanced Studies, IMDEA Nanoscience, Calle Faraday 9, Campus Cantoblanco, 28049 Madrid, Spain

[*] Correspondence: minsang@snu.ac.kr (M.S.K.); reinhold.wannemacher@imdea.org (R.W.); johannes.gierschner@imdea.org (J.G.)

Contents

Supplementary Note 1. General information 3

1.1 Instrumentation 3

1.2 General experimental procedures 5

1.3 Syntheses of cyanoarene-based PCs 6

1.4 DFT computational details 10

Supplementary Note 2. Previous study about photoredox catalysis 11

Supplementary Note 3. PCs characterization 13

Supplementary Note 4. PL decay quenching experiments 19

Supplementary Note 5. Tertiary amine effect in photoredox reductive dehalogenation 20

Supplementary Note 6. Reproducibility test in photoredox reductive dehalogenation 21

Supplementary Note 7. Generation of radical anion of PCs 22

Supplementary Note 8. Validation of ConPET process in photoredox reductive dehalogenation 25

Supplementary Note 9. Computational study: DFT and analytical model 26

9.1 Analytical model for exciton population of PCs 26

9.2 TD-DFT results for 4DP-IPN 28

9.3 Kinetic simulation of photoredox reductive dehalogenation 29

9.4 Derivation of rate constants from calculated and experimental methods 32

9.5 Matlab code 39

Supplementary Note 10. Photodegradation behavior of cyanoarenes-based PC 43

Supplementary Note 11. Oxygen tolerance in photoredox reductive dehalogenation 55

Supplementary Note 12. Subsequential injection of 4DP-IPN in photoredox reductive dehalogenation 57

Supplementary Note 13. NMR analysis of PCs 58

Supplementary Note 14. Validation of broad scope of aryl/alkyl halides 73

Supplementary Note 15. Gram-scaled reaction of photoredox reductive dehalogenation 135

Supplementary References 137

Supplementary Note 1. General information

■ Chemicals

Commercially available PCs (Ir- complexes, Ru- complexes, Rh6G and PDI) were purchased in Aldrich, TCI and Alfa Aesar. Other PCs were synthesized according to the synthetic procedures.[1] All chemicals and solvents were purchased commercially and used without further purifications.

1.1 Instrumentation

■ Photophysical measurements

UV-Vis absorbance measurements were performed with a V-770 (JASCO) UV-Vis-NIR spectrometer. Steady-state PL emission spectra at RT were obtained using a HORIBA Jobin Yvon Fluoromax-4 spectrofluorimeter equipped with a 150 W Xe short arc lamp and Hamamatsu R928P PMT detector; the emission spectra were corrected for the sensitivity of the photomultiplier.

PL decay measurements were carried out by the time-correlated single photon counting (TCSPC) technique. The excitation source was a 377 nm pulsed diode laser (LDH series PicoQuant) of pulse width (FWHM) < 49 ps. A NanoHarp−250 TCSPC event timer with 25 ns time resolution was employed to measure decays of delayed fluorescence. The decay time fitting procedure was carried out by using the Fluofit software (PicoQuant). For temperature-dependent PL studies a Picoquant LDH−D−C−405 pulsed diode laser and a Picoquant HydraHarp−400 TCSPC event timer with 1 ps time resolution were used to measure the decays of the prompt fluorescence, and a 355 nm Nd:YAG laser with a pulse width of 300 ps (Teemphotonics) and a Picoquant TimeHarp 260 nano TCSPC electronics with 1 ns resolution were employed on short (nanosecond to microsecond) and long (microsecond to second) time scales, respectively. Low-temperature PL was dispersed in wavelength using an Acton SP2500 spectrometer and detected either by a Princeton Instruments Spec10:400BR CCD camera or by a low dark current hybrid photomultiplier (PMA 06, PicoQuant), both attached to the spectrometer. Gated PL spectra were acquired with a delay of 250 ms, using a cw 405 nm laser module with TTL modulation input (maximum modulation frequency 20 kHz) and suitable triggering of the CCD. Trigger pulses for the laser and the CCD camera were provided by a Stanford Research Systems DG645 pulse and delay generator with 5 ps resolution. The decay time fitting procedure was carried out by using the Fluofit software (PicoQuant).

■ Electrochemical measurements

Cyclic voltammetry (CV) experiments were carried out with a VersaSTAT3-200 (Princeton Applied Research) using a one compartment electrolysis cell consisting of a glassy carbon working electrode, a platinum wire counter electrode, and a quasi Ag^{+}/Ag (sat. KCl aqueous solution) reference electrode bought from AT FRONTIER (Part No. R303).

Specifically, the electrode is a silver wire that is coated with a thin layer of silver chloride and an insulated lead wire connects the silver wire with measuring instrument. The electrode also consists of a porous plug on the one end which will allow contact between the field environment with the silver chloride electrolyte. Saturated potassium chloride is added inside the body of the electrode to stabilize the silver chloride concentration and in this condition the electrode's reference potential is known to be +0.197 V at 25 °C. The measurements were done in 0.2 mM CH_3CN solution with 0.1 M tetrabutylammonium hexafluorophosphate (n-Bu_4NPF_6, Aldrich, Electrochemical grade) as supporting electrolyte at a scan rate of 100 mV/s. The redox potential was calibrated after each experiment against the ferrocenium/ferrocene couple (Fc^+/Fc), which allowed conversion of all potentials to the aqueous saturated calomel electrode (SCE) scaled by using E^0 (Fc^+/Fc) = 0.42 V vs SCE in CH_3CN. The working solution was degassed with Ar for 15 min before measurement and then kept under a positive Ar pressure during the measurement.

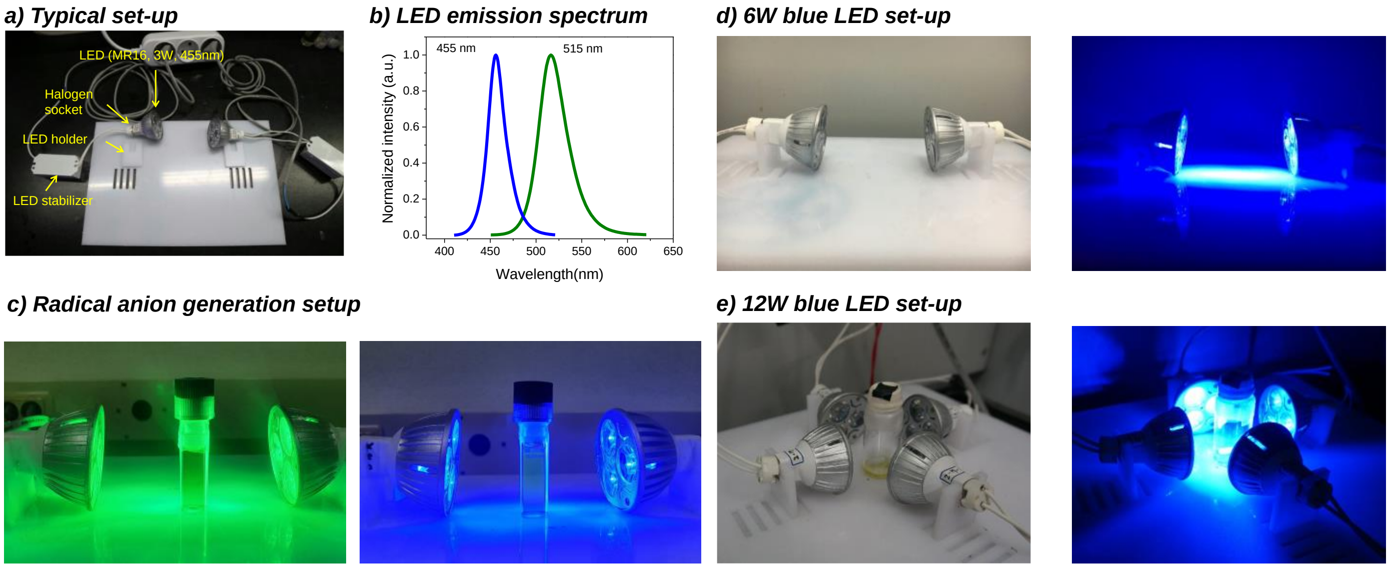

Supplementary Figure 1 (**a–e**) Information of irradiation set-up and equipment to conduct photoreaction. The optical power of LEDs was measured by energy-meter (Thorlab, PM100D).

■ Sample measurements

Newly dehalogenated products were characterized by gas chromatography (GC) and ^{1}H NMR spectrometer (400 MHz) with $CDCl_3$ or DMSO-d_6 as the solvent. The yields of dehalogenated products were determined by GC, coupled with flame ionization detector (FID) and (5%-phenyl)-methylpolysiloxane column (Agilent, HP-5), or ^{1}H NMR in the presence of 1,3,5-trimethoxybenzene as the internal standard to determine conversion and yields. GC-FID was programmed with gradient temperature analysis using nitrogen gas as the carrier gas.

1.2 General experimental procedures

■ General procedure for PC radical anion generation

Inside the glove box, a sealable quartz cuvette was charged with PC (1.0×10^{-4} M) and DIPEA (0.5 M) in 3 mL of anhydrous CH_3CN. After that, the quartz cuvette was capped with a screw cap and sealed with parafilm. Subsequently, the reaction was carried out under illumination of two 3W 455 or 515 nm LEDs for 1 min at RT. After illumination, UV-Vis absorption spectra of the illuminated solutions were measured immediately. In preparation of the reaction, pre-prepared stock solutions of the PCs were used for higher reproducibility of results.

■ General procedure for PC photodegradation

Outside the glove box, a 20 mL glass vial equipped with a stirring bar was charged with PC (1.0×10^{-4} M) and DIPEA (0.5 M) in 1 mL of anhydrous CH_3CN as a solvent. Then, the vial was capped with a rubber septum or screw cap and sealed with parafilm. The reaction batches were purged with N_2 (99.999%) for 30 min outside the glove box. Subsequently, the reaction was carried out for 2 hours under illumination of two 3W 455 nm LEDs at RT. Without work-up process, the aliquots of crude reaction mixture were diluted by CH_2Cl_2 and monitored by TLC. In preparation of the reaction, pre-prepared stock solutions of the PCs were used for higher reproducibility of results. For scale-up reaction of photodegradation, a 20 mL glass vial equipped with a stirring bar was charged with PC (1.0×10^{-2} M) and DIPEA (1 M) in 5–6 mL of anhydrous CH_3CN as a solvent. Then, the vial was capped with a rubber septum or screw cap and sealed with parafilm. Subsequently, the reaction was carried out for 2 hours under illumination of four 3W 455 nm LEDs at RT without any degassing process.[2] Afterwards, the reaction mixture was evaporated under low-pressure and the concentrated crude products were further purified by column chromatography on silica gel.

■ General procedure for photoredox reductive dehalogenation

Inside the glove box, a 20 mL glass vial equipped with a stirring bar was charged with aryl halides (0.1 mmol), DIPEA (174 μL, 1 mmol), PC (5-0.001 mol% to relative aryl halides), 1,3,5-trimethoxybenzene (33.6 mg, 0.2 mmol) as internal standard for GC-FID and ^{1}H NMR, and anhydrous CH_3CN (1 mL, 0.1 M of aryl halides) as solvent. After, the vial was capped with a rubber septum or screw cap and sealed with parafilm. The reaction batches were purged with Ar (99.9999%) (or with air or with O_2 (99.995%)) for 30 min outside the glove box. Subsequently, the reaction was carried out for hours under illumination of two 3W 455 nm LEDs at RT. Without isolation process, the aliquots of crude reaction mixture were analyzed by GC-FID or ^{1}H NMR to obtain yields of dehalogenated products. In preparation of the reaction, pre-prepared stock solutions of the PCs were used for the higher reproducibility of results.

1.3 Syntheses of cyanoarene-based PCs

4-nitrosobenzonitrile: A solution of 4-aminobenzonitrile (2.0 g, 16.9 mmol), potassium peroxymonosulfate (20.8 g, 33.86 mmol) in CH_2Cl_2 (56 mL) and water (200 mL) was rigorously stirred for 3 hours under ambient conditions. After 3 hours, the organic layers were extracted by 1 M HCl aqueous solution (50 mL), neutralized by saturated $NaHCO_3$ aqueous solution (60 mL) and washed by brine 3 times. Subsequently, the organic layers were dried over $NaSO_4$ and concentrated under reduced pressure. The collected crude product was a gray greenish solid and used for next synthesis of 4-monocyanodiphenylamine without further purification. Data were in full agreement with those reported in literature.[3] ^1H NMR (400 MHz, $CDCl_3$): δ 7.97 (br s, 4H).

4-monocyanodiphenylamine: For the synthesis, a procedure using metal-free Chan-Evans-Lam coupling was followed.[4] A solution **of 4-nitrosobenzonitrile (1**.0 g, 7.57 mmol), phenylboronic acid (1.38 g, 11.4 mmol) and triethyl phosphite (1.51 g, 9.10 mmol) in anhydrous THF (20 mL) was stirred for 1 hour under ambient conditions. After 1 hour, the reaction mixture was filtered through a small plug of silica (EA:hexanes, 1:9 v/v). After the filtration, the mixture was further purified by column chromatography on silica gel with gradient CH_2Cl_2/hexanes mixtures to give pure product as yellow solid. Data were in full agreement with those reported in literature.[5] ^1H NMR (400 MHz, $CDCl_3$): δ 7.4 –7.42 (m, 2H), 7.39–7.30 (m, 2H), 7.20–7.13 (m, 2H), 7.13–7.08 (m, 1H), 6.99–6.93 (m, 2H), 6.24 (s, 1H).

2,4'-dicyanodiphenylamine: A solution of potassium tert-butoxide (1.39 g, 12.39 mmol), 2-aminobenzonitrile (1.17 g, 9.91 mmol) in anhydrous DMSO (15 mL) was stirred for 1 hour at RT under a nitrogen atmosphere. After 1 hour, 4-fluorobenzonitrile (1.2 g, 16.5 mmol) dissolved in DMSO (5 mL) was slowly added to the reaction mixture and stirred further at RT for 24 hours. Afterwards, distilled water was poured into the reaction mixture to quench the excess potassium tert-butoxide and to precipitate the crude product, which was further purified by reprecipitation in MeOH/water to give pure product as pale red solid (0.98 g, 45%). Data were in full agreement with those reported in literature.[6] ^1H NMR (400 MHz, $CDCl_3$): δ 7.60 (dd, 3H), 7.55–7.48 (m, 1H), 7.42 (dd, 1H), 7.20–7.14 (m, 2H), 7.08 (td, 1H), 6.57 (s, 1H).

4,4′-dicyanodiphenylamine: A solution of potassium tert-butoxide (2.22 g, 19.8 mmol), 4-aminobenzonitrile (1.95 g, 16.5 mmol) in anhydrous DMSO (30 mL) was stirred for 1 hour at RT under a nitrogen atmosphere. After 1 hour, 4-fluorobenzonitrile (2.0 g, 16.5 mmol) dissolved in DMSO (10 mL) was slowly added to the reaction mixture and stirred further at RT overnight. Afterwards, distilled water was poured into the reaction mixture to quench the excess potassium tert-butoxide and to precipitate the crude product, which was further purified by reprecipitation in MeOH/water to give pure product as pale red solid (1.74 g, 48%). Data were in full agreement with those reported in literature.[7] ^1H NMR (400 MHz, $CDCl_3$): δ 7.62–7.57 (dt, 4H), 7.18–7.15 (dt, 4H), 6.38–7.15 (s, 1H).

4DP-IPN: A solution of NaH (60% in mineral oil, 0.477g, 11.94 mmol) and diphenylamine (1.48 g, 8.75 mmol) in anhydrous DMAc (5 mL) was stirred for 30 min in ice bath under a nitrogen atmosphere. After 30 min, 2,4,5,6-tetrafluoroisophthalonitrile (0.4 g, 1.99 mmol) dissolved in DMAc (5 mL) was slowly added to the reaction mixture and

stirred further at 100 °C for 10 h. Afterwards, distilled water (2 mL) was poured into the reaction mixture to quench the excess NaH and, methanol was added to precipitate the crude product, which was further purified by column chromatography on silica gel (CH_2Cl_2:hexanes, 2:3 v/v) to give pure product as yellow solid (1.32 g, 83%). Data were in full agreement with those reported in literature.[1,8] ^{1}H NMR (400 MHz, DMSO-D_6): δ 7.31 7.21 (m, 8H), 7.10–7.05 (t, 8H), 7.03–6.97 (t. 2H), 6.91–6.75 (m, 16H), 6.68–6.62 (m, 6H).

4tCz-IPN: A solution of *t*-BuOK (0.193 g, 1.72 mmol) and 3,6-di-tert-butyl-9H-carbazole (0.400 g, 1.43 mmol) in anhydrous THF (40 mL) was stirred for 30 min in ice bath under a nitrogen atmosphere. After, 2,4,5,6-tetrafluoroisophthalonitrile (0.057 g, 0.28 mmol) was slowly added to the reaction mixture and stirred further for 12 h. After completion of the reaction, distilled water (2 mL) was poured into the reaction mixture to quench the excess NaH. The resulting solution was concentrated under reduced pressure followed by washing several times with water and ethanol to yield the crude product, which was purified by column chromatography on silica gel (CH_2Cl_2:hexanes, 2:1 v/v) to give pure product (0.160 g, 45%). Data were in full agreement with those reported in literature.[1] ^{1}H NMR (400 MHz, $CDCl_3$): δ 8.21 (d, 2H), 7.74 (dd, 2H), 7.61–7.59 (m, 6H), 7.18 (d, 2H), 7.05–7.00 (m, 8H), 6.51 (dd, 2H), 6.44 (d, 2H), 1.53 (s, 18H), 1.30 (s, 36H), 1.22 (s, 18H).

4Cz-IPN: A solution of NaH (60% in mineral oil, 0.738 g, 18.45 mmol) and carbazole (2.16 g, 12.30 mmol) in anhydrous THF (40 mL) was stirred for 30 min in ice bath under a nitrogen atmosphere. After, 2,4,5,6-tetrafluoroisophthalonitrile (0.5 g, 2.46 mmol) was slowly added to the reaction mixture and stirred further for 12 h. After completion of the reaction, distilled water (2 mL) was poured into the reaction mixture to quench the excess NaH. The resulting solution was concentrated under reduced pressure followed by washing several times with water and ethanol to yield the crude product, which was purified by column chromatography on silica gel (CH_2Cl_2:hexanes, 2:1 v/v) to give pure product (1.48 g, 93%) Data were in full agreement with those reported in literature.[1,9,10] ^{1}H NMR (400 MHz, CDCl3): δ 8.22 (dt, 2H), 7.74–7.67 (m, 8H), 7.49 (ddd, 2H), 7.33 (dt, 2H), 7.23–7.21 (m, 4H), 7.12–7.05 (m, 8H), 6.82 (td, 4H), 6.63 (ddd, 2H).

4-p,p-DCDP-IPN: A solution of NaH (60% in mineral oil, 0.120 g, 3 mmol) and 4,4′-dicyanodiphenylamine (0.548 g, 2.5 mmol) in anhydrous DMAc (5 mL) was stirred for 30 min in ice bath under a nitrogen atmosphere. After 30 min, 2,4,5,6-tetrafluoroisophthalonitrile (0.1 g, 0.5 mmol) dissolved in DMAc (5 mL) was slowly added to the reaction mixture and stirred further at 60 °C for 12 h. Afterwards, distilled water (2 mL) was poured into the reaction mixture to quench the excess NaH and, methanol was added to precipitate the crude product, which was further purified by reprecipitation in CH2Cl2$_/$/ethyl acetate to give pure product as yellow solid (0.140 g, 28%). ^{1}H NMR (400 MHz, acetone-d_6): δ 7.87–7.81 (d, 4H), 7.70–7.62 (d, 8H), 7.55–7.48 (td. 8H), 7.20–7.15 (d, 8H), 7.10–7.03 (d, 4H). MS (GC-FAB-HRMS): calculated for $C_{64}H_{33}N_{14}$ $[M+H]^+$: 997.3013; found as 997.3005.

3DP-F-IPN: A procedure similar to the synthesis of 4Cz-IPN is followed. A solution of potassium tert-butoxide (2.424 g, 21.6 mmol), diphenylamine (3.03 g, 18.0 mmol) in anhydrous DMAc (20 mL) was stirred for 2 hours in ice bath

under a nitrogen atmosphere. After 2 hours, 2,4,5,6-tetrafluoroisophthalonitrile (1.2 g, 6.0 mmol) dissolved in DMAc (5 mL) was slowly added to the reaction mixture and stirred further at RT for 48 hours. Afterwards, distilled water (2 mL) was poured into the reaction mixture to quench the excess potassium tert-butoxide and, methanol was added to precipitate the crude product, which was further purified by recrystallization in $CHCl_3$/MeOH to give pure product as yellow solid (1.02 g, 26%). Data were in full agreement with those reported in literature.[11] ^{1}H NMR (400 MHz, $CDCl_3$): δ 7.29–7.22 (m, 12H), 7.09–7.02 (m, 6H), 7.02–6.95 (m, 12H).

3DP-Cz-IPN: A solution of potassium tert-butoxide (0.124 g, 1.10 mmol), carbazole (0.154 g, 0.927 mmol) in anhydrous DMAc (5 mL) was stirred for 1 hour in ice bath under a nitrogen atmosphere. After 1 hour, 3DP-F-IPN (0.5 g, 0.772 mmol) dissolved in DMAc (5 mL) was slowly added to the reaction mixture and stirred further at 70 °C for 6 hours. Afterwards, distilled water (2 mL) was poured into the reaction mixture to quench the excess potassium tert-butoxide and, methanol was added to precipitate the crude product, which was further purified by column chromatography on silica gel with gradient CH_2Cl_2/hexanes mixtures to give pure product as yellow solid. ^{1}H NMR (400 MHz, $CDCl_3$): δ 7.55–7.50 (d, 2H), 7.40–7.35 (t, 4H), 7.20–7.15 (d, 4H), 7.14–7.08 (t, 2H), 7.07–7.03 (t, 2H), 6.98– 6.88 (dt, 4H), 6.80–6.71 (t, 8H), 6.65–6.58 (t, 4H), 6.48–6.40 (t, 8H). MS (GC-FAB-HRMS): calculated for $C_{56}H_{39}N_6$ $[M+H]^+$: 795.3236; found as 795.3231.

3DP-DCDP-IPN: A solution of sodium hydride (0.060 g, 1.50 mmol) and 4,4′-dicyanodiphenylamine (0.202 g, 0.922 mmol) in anhydrous DMF/DMSO (3/7) (10 mL) was stirred for 1 hour in ice bath under a nitrogen atmosphere. After 1 hour, **3DP-F-IPN** (0.5 g, 0.772 mmol) was slowly added to the reaction mixture and stirred further at 120 °C for 17 hours. Afterwards, distilled water (2 mL) was poured into the reaction mixture to quench the excess NaH and, methanol was added to precipitate the crude product, which was further purified by column chromatography on silica gel with gradient CH_2Cl_2/hexanes mixtures to give pure product as yellow solid (0.117 g, 18%). ^{1}H NMR (400 MHz, DMSO-d_6): δ 7.40–7.35 (d, 4H), 7.30–7.25 (d, 8H), 7.13–7.07 (t, 8H), 7.05–7.00 (q, 2H), 6.90–6.75 (m, 16H). MS (GC-FAB-HRMS): calculated for $C_{58}H_{39}N_8$ $[M+H]^+$: 847.3298; found as 847.3290.

3DP-DMDP-IPN: A solution of sodium hydride (0.037 g, 0.926 mmol) and 4,4'-dimethoxydiphenylamine (0.127 g, 0.556 mmol) in anhydrous DMF/DMSO (3/7) (10 mL) was stirred for 0.5 h in ice bath under a nitrogen atmosphere. After 1 hour, 3DP-F-IPN (0.3 g, 0.463 mmol) was slowly added to the reaction mixture and stirred further at 120 °C for 16 hours. Afterwards, distilled water (2 mL) was poured into the reaction mixture to quench the excess sodium hydride and methanol was added to precipitate the crude product, which was further purified by column chromatography on silica gel with gradient CH_2Cl_2/hexanes mixtures to give the pure product as reddish orange powder. ^{1}H NMR (400 MHz, $CDCl_3$): δ 7.25 (d, 4H), 7.07 (m, 12H), 7.00 (t, 2H), 6.87 (t, 4H), 6.70 (d, 8H), 6.46–6.38 (m, 8H), 3.60 (s, 6H). MS (GC-FAB-HRMS): calculated for $C_{58}H_{45}N_6O_2$ $[M+H]^+$: 857.3604; found as 857.3602.

i

4-p-MCDP-IPN: A solution of potassium tert-butoxide (0.741 g, 6.6 mmol), 4-cyanodiphenylamine (0.855 g, 4.4 mmol) in anhydrous DMSO (5 mL) was stirred for 1 hour in ice bath under a nitrogen atmosphere. After 1 hour, 2,4,5,6-

tetrafluoroisophthalonitrile (0.2 g, 1.0 mmol) dissolved in DMSO (5 mL) was slowly added to the reaction mixture and stirred further at 60 °C overnight. Afterwards, distilled water (2 mL) was poured into the reaction mixture to quench the excess potassium tert-butoxide and, methanol was added to precipitate the crude product, which was further purified by washing with CH_2Cl_2 to give pure product as yellow solid. ^{1}H NMR (400 MHz, acetone-d_6): δ 7.64–7.57 (m, 8H), 7.39 (dd, 8H), 7.34–7.24 (m, 8H), 7.24–7.09 (m, 12H). MS (MALDI-TOF-LRMS): calculated for $C_{60}H_{36}N_{10}$ $[M+H]^+$: 897.3203; found as 897.9657. MS (GC-FAB-HRMS): not found.

4-o,p-DCDP-IPN: A solution of NaH (60% in mineral oil, 0.120g, 3 mmol) and 2,4′-dicyanodiphenylamine (0.548g, 2.5 mmol) in anhydrous DMAc (3 mL) was stirred for 30 min in ice bath under a nitrogen atmosphere. After 30 min, 2,4,5,6-tetrafluoroisophthalonitrile (0.1 g, 0.5 mmol) dissolved in DMAc (2 mL) was slowly added to the reaction mixture and stirred further at 60 °C for 6 hours. Afterwards, distilled water (2 mL) was poured into the reaction mixture to quench the excess NaH. The organic layers were extracted by EA and brine 3 times. Subsequently, the organic layers were dried over $NaSO_4$ and concentrated under reduced pressure which was further purified by column chromatography on silica gel with gradient CH_2Cl_2/hexanes mixtures to give pure product as reddish orange solid. ^{1}H NMR (400 MHz, acetone-d_6): δ 7.68–7.56 (m, 8H), 7.51 (d, 8H), 7.45 (d, 1H), 7.36 (d, 3H), 7.26–7.15 (m, 7H), 7.07 (d, 5H). MS (GC-FAB-HRMS): not found.

1.4 DFT computational details

Density functional theory (DFT) and time-dependent (TD) DFT calculations were performed with the B3LYP functional and 6–311++G* basis set, as all implemented in the Gaussian16 program package. The geometries (S_0 and D_0) optimization and single point energies were calculated in CH_3CN solution employing the polarizable continuum model (PCM). In all geometry optimization calculations, frequency calculations were performed both to verify that geometries were true minima and to obtain free energies at 298.15 K. To obtain the calculated UV-Vis absorption spectrum of the neutral (S_0) and radical anion (D_0) species, vertical transition energies for the first 60 excited states were obtained by single-point calculations on the optimized S_0 and D_0 geometries, respectively. Molecular orbital (MO) topologies were obtained by employed in Gaussian09w and plotted with GaussView 6.0. To derive calculated redox potentials, the Gibbs free energies, $\Delta G_{solv}^{\circ,redox}$, between radical and neutral species were converted to SCE by a given process, $E_{redox,SCE}^{\circ}(\text{V vs SCE}) = E_{redox}^{\circ}(eV) - 4.68\ eV$. The Gibbs free energy and enthalpy with relevant standard state reference were obtained from following relations,[12] G^0 *(298.15 K, 1 M) =* G^{0*} *(298.15 K, 1 atm) + 1.89 kcal/mol* and H^0 *(298.15 K, 1 M) =* H^{0*} *(298.15 K, 1 atm) + 0.59 kcal/mol.*

In fact, we have done comparisons with different functionals (including CAM-B3LYP, M06-2X and wB97XD), and we saw very different behaviors for absolute and relative energies of the S_2 and T_n states (and in particular with respect to the ΔE_{ST}) as well as for the state descriptions. The B3LYP functional was chosen because if its good performance of the ΔE_{ST} with respect to experiment as shown by us earlier.[1] For the calculated radical anion absorption, the lowest excited state was actually calculated to be lower with CAM-B3LYP and M06-2X in comparison with B3LYP, so that the latter method still appears quite reasonable to describe the electronic nature of ground and excited states properties for both the neutral and the radical anion species.

Supplementary Note 2. Previous study about photoredox catalysis

Supplementary Table 1 Summary of previously reported photoredox reductive dehalogenation

PC	*Reaction conditions*	*Substrate*	*ref*
$Ir(ppy)_3$ (0.05 mol%)	TBA (5 eq.), HCOOH (5 eq.), RT, CH_3CN, blue or white LED in flow reactor	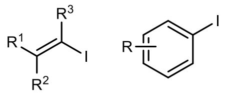	(13)
PDI (5 mol%)	TEA (8 eq.), 40 °C, DMF, 455 nm LED	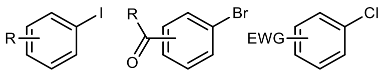	(14)
PTH (5 mol%)	Bu_3N (5 eq.), HCOOH (5 eq.), RT, CH_3CN, 380 nm LED	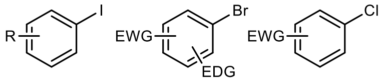	(15)
$Ru(bpy)_3Cl_2$ (1 mol%) + Pyrene (5 mol%)	DIPEA (1.4 eq.), RT, DMSO, 455 nm LED	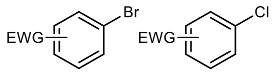	(16)
$Ru(bpy)_3Cl_2$ (1~2 mol%)	Sodium dodecyl sulfate, ascorbic acid (3.2 eq.), RT, aqueous solution, 532 nm LED	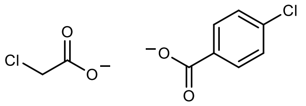	(17)
$Ru(bpy)_3Cl_2$ (5 mol%) + APA (1.2 eq.)	RT, aqueous solution, 532 nm LED in the presence of O_2	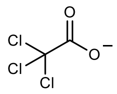	(18)
No PC	^tBuOK (5 eq.), 35 °C, DMF 27 W 405 nm LED	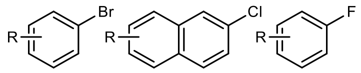	(19)
Mes-Acr-BF_4 (10 mol%)	DIPEA (3 eq.), RT, CH_3CN, 390 nm LED	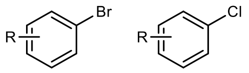	(20)
4Cz-IPN (5 mol%)	TEA (3 eq.), methyl thioglycolate (20 mol%), RT, CH_3CN, H_2O, 444 nm LED	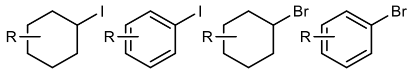	(21)
NpMI (10 mol%)	Electrochemical RVC(+)|RVC(-) 0.8 mA, RT, CH_3CN, 440 nm LED	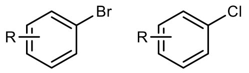	(22)
4DP-IPN (0.005 ~ 5 mol%)	DIPEA (10 eq.), RT, CH_3CN, 455 nm LED in the presence of O_2	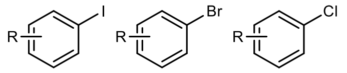	Current work

Supplementary Figure 2 Summary of redox potentials of conventional previously studied purely organic PCs.[23]

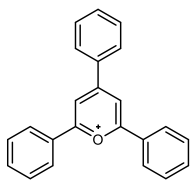

Triphenylpyrylium

$E_{red,\,S_1}^{*}$ ($PC^{*}/PC^{\bullet-}$) = +2.55 V
$E_{red,\,T_1}^{*}$ ($PC^{*}/PC^{\bullet-}$) = +2.02 V
E_{red}^{0} ($PC/PC^{\bullet-}$) = –0.32 V

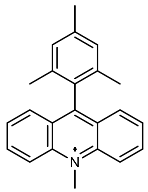

9-mesityl-10-methylacridinium

$E_{red,\,S_1}^{*}$ ($PC^{*}/PC^{\bullet-}$) = +2.18 V
$E_{red,\,T_1}^{*}$ ($PC/PC^{\bullet-}$) = +1.45 V
E_{red}^{0} ($PC/PC^{\bullet-}$) = –0.49 V

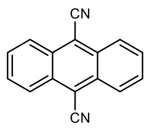

9,10-dicyanoanthracene

$E_{red,\,S_1}^{*}$ ($PC^{*}/PC^{\bullet-}$) = +1.99 V
$E_{red,\,T_1}^{*}$ ($PC/PC^{\bullet-}$) = +0.9 V
E_{red}^{0} ($PC/PC^{\bullet-}$) = –0.91 V

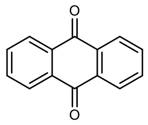

Anthraquinone

$E_{red,\,T_1}^{*}$ ($PC^{*}/PC^{\bullet-}$) = +1.77 V
E_{red}^{0} ($PC/PC^{\bullet-}$) = –0.96 V

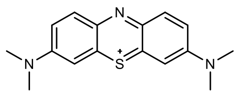

Methylene Blue

$E_{red,\,S_1}^{*}$ ($PC^{*}/PC^{\bullet-}$) = +1.56 V
$E_{ox,\,S_1}^{*}$ ($PC^{\bullet+}/PC^{*}$) = –0.73 V
$E_{red,\,T_1}^{*}$ ($PC^{*}/PC^{\bullet-}$) = +1.60 V
$E_{ox,\,T_1}^{*}$ ($PC^{\bullet+}/PC^{*}$) = –0.68 V
E_{red}^{0} ($PC/PC^{\bullet-}$) = –0.30 V
E_{ox}^{0} ($PC^{\bullet+}/PC$) = +1.13 V

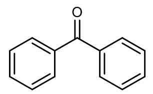

Benzophenone

$E_{red,\,S_1}^{*}$ ($PC^{*}/PC^{\bullet-}$) = +1.50 V
$E_{ox,\,S_1}^{*}$ ($PC^{\bullet+}/PC^{*}$) = –0.83 V
$E_{red,\,T_1}^{*}$ ($PC^{*}/PC^{\bullet-}$) = +1.28 V
$E_{ox,\,T_1}^{*}$ ($PC^{\bullet+}/PC^{*}$) = –0.61 V
E_{red}^{0} ($PC/PC^{\bullet-}$) = –1.72 V
E_{ox}^{0} ($PC^{\bullet+}/PC$) = +2.39 V

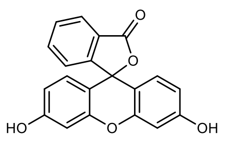

Fluorescein

$E_{red,\,S_1}^{*}$ ($PC^{*}/PC^{\bullet-}$) = +1.25 V
$E_{ox,\,S_1}^{*}$ ($PC^{\bullet+}/PC^{*}$) = –1.55 V
$E_{red,\,T_1}^{*}$ ($PC^{*}/PC^{\bullet-}$) = +0.77 V
$E_{ox,\,T_1}^{*}$ ($PC^{\bullet+}/PC^{*}$) = –1.07 V
E_{red}^{0} ($PC/PC^{\bullet-}$) = –1.17 V
E_{ox}^{0} ($PC^{\bullet+}/PC$) = +0.87 V

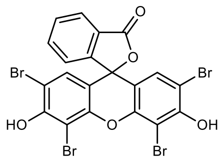

Eosin Y

$E_{red,\,S_1}^{*}$ ($PC^{*}/PC^{\bullet-}$) = +1.23 V
$E_{ox,\,S_1}^{*}$ ($PC^{\bullet+}/PC^{*}$) = –1.58 V
$E_{red,\,T_1}^{*}$ ($PC^{*}/PC^{\bullet-}$) = +0.83V
$E_{ox,\,T_1}^{*}$ ($PC^{\bullet+}/PC^{*}$) = –1.15 V
E_{red}^{0} ($PC/PC^{\bullet-}$) = –1.08 V
E_{ox}^{0} ($PC^{\bullet+}/PC$) = +0.76 V

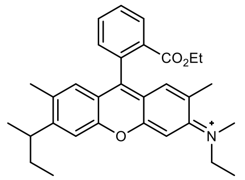

Rhodamine 6G

$E_{red,\,S_1}^{*}$ ($PC^{*}/PC^{\bullet-}$) = +1.18 V
$E_{ox,\,S_1}^{*}$ ($PC^{\bullet+}/PC^{*}$) = –1.09 V
$E_{red,\,T_1}^{*}$ ($PC^{*}/PC^{\bullet-}$) = +0.95 V
$E_{ox,\,T_1}^{*}$ ($PC^{\bullet+}/PC^{*}$) = –0.86 V
E_{red}^{0} ($PC/PC^{\bullet-}$) = –1.14 V
E_{ox}^{0} ($PC^{\bullet+}/PC$) = +1.23 V

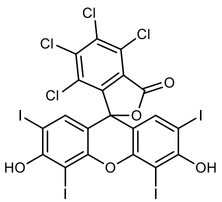

Rose Bengal

$E_{red,\,S_1}^{*}$ ($PC^{*}/PC^{\bullet-}$) = +1.18 V
$E_{ox,\,S_1}^{*}$ ($PC^{\bullet+}/PC^{*}$) = –1.33 V
$E_{red,\,T_1}^{*}$ ($PC^{*}/PC^{\bullet-}$) = +0.81 V
$E_{ox,\,T_1}^{*}$ ($PC^{\bullet+}/PC^{*}$) = –0.96 V
E_{red}^{0} ($PC/PC^{\bullet-}$) = –0.99 V
E_{ox}^{0} ($PC^{\bullet+}/PC$) = +0.84 V

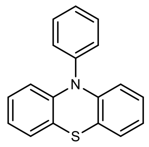

PTH

$E_{ox,\,S_1}^{*}$ ($PC^{\bullet+}/PC^{*}$) = –2.10 V
$E_{ox,\,T_1}^{*}$ ($PC^{\bullet+}/PC^{*}$) = –1.70 V
E_{ox}^{0} ($PC^{\bullet+}/PC$) = +0.68 V

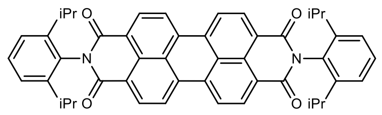

PDI

$E_{red,\,S_1}^{*}$ ($PC^{*}/PC^{\bullet-}$) = +1.92 V
$E_{ox,\,S_1}^{*}$ ($PC^{\bullet+}/PC^{*}$) = –0.72 V
$E_{red,\,T_1}^{*}$ ($PC^{*}/PC^{\bullet-}$) = +0.77 V
$E_{ox,\,T_1}^{*}$ ($PC^{\bullet+}/PC^{*}$) = +0.43 V
E_{red}^{0} ($PC/PC^{\bullet-}$) = –0.43 V
E_{ox}^{0} ($PC^{\bullet+}/PC$) = +1.63 V

Supplementary Note 3. PCs characterization

Supplementary Table 2 Summary of redox potentials of PCs studied in the current work

PC	E^{0}_{red}	E^{0}_{ox}	$E^{*}_{red,\ S1}$	$E^{*}_{red,\ T1}$	$E^{*}_{ox,\ S1}$	$E^{*}_{ox,\ T1}$
$Ru(bpy)_3Cl_2{\cdot}6H_2O$[24]	-1.33	+1.29	-	+0.77	-	-0.81
$Ru(phen)_3(PF_6)_2$[24]	-1.36	+1.26	-	+0.82	-	-0.87
$Ir(ppy)_3$[24]	-2.19	+0.77	-	+0.31	-	-1.73
Rh6G[23]	-1.14	+1.23	+1.18	+0.95	-1.09	-0.86
4Cz-IPN[1]	-1.21	+1.52	+1.38	+1.33	-1.07	-1.02
4DP-IPN[1,a]	-1.66	+1.01	+0.79	+0.63	-1.44	-1.28

Redox potential values are against SCE and referred to literature.[1,23,24] [a]Redox potentials values were measured in CH_3CN in the current work.

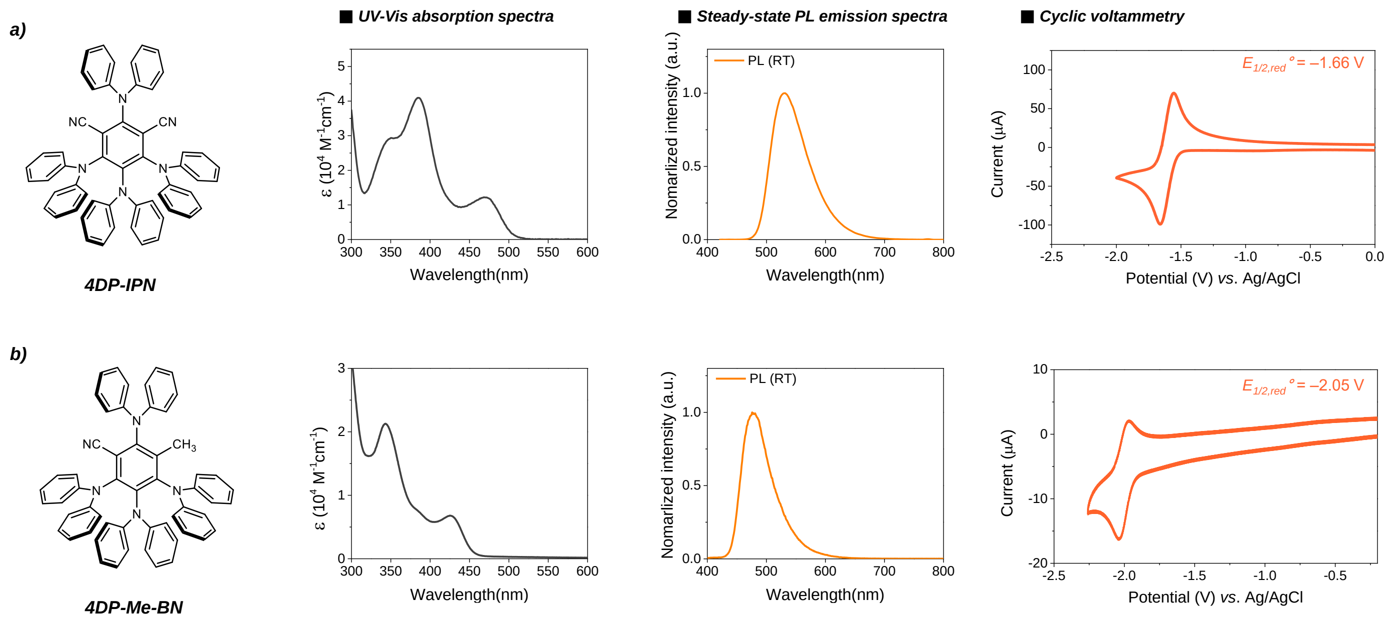

Supplementary Figure 3 Characterization of (**a**) 4DP-IPN and (**b**) 4DP-Me-BN. UV-Vis absorption spectra of PC (1.0×10^{-5} M) in CH_3CN solution were measured at RT. Steady-state photoluminescence (PL) spectra of PCs (1.0×10^{-5} M) in CH_3CN at RT. CV spectra of reduction cycle of PCs (2.0×10^{-4} M) were obtained in CH_3CN at RT after the degassing process by purging with Ar for 15 min, all potentials were converted to the SCE scale by using $E^{0}(Fc^{+}/Fc)$ = 0.42 V vs SCE in CH_3CN.

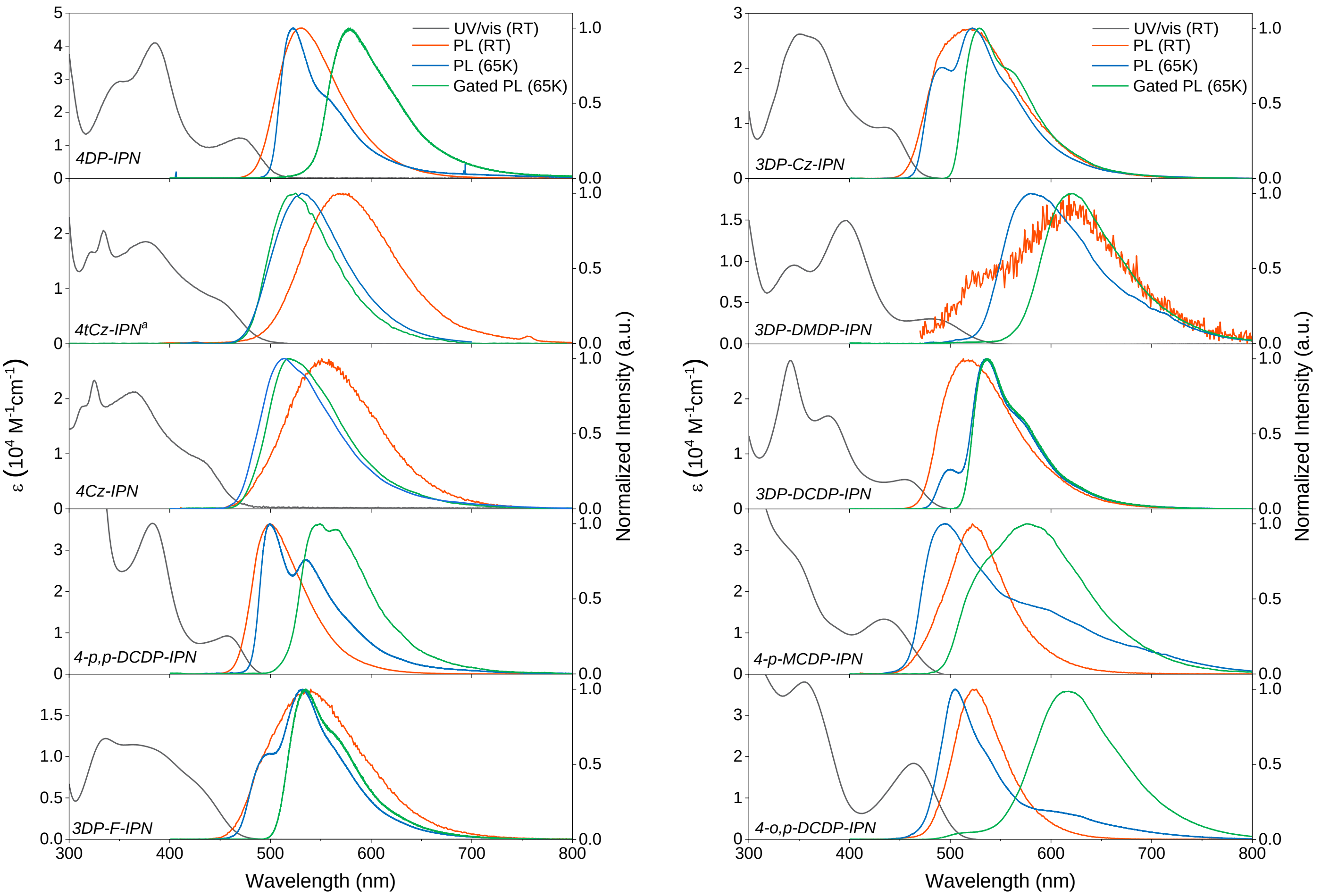

Supplementary Figure 4 UV-Vis absorption and PL emission spectra of PCs studied in current work. UV-Vis absorption spectra of PC (1.0×10^{-5} M) in CH_3CN solution were measured at RT. Steady-state photoluminescence (PL) spectra of PCs (1.0×10^{-5} M) in CH_3CN at RT and 65K. [a]UV-Vis absorption and PL emission spectra of 4tCz-IPN were referred to liturature.[1]

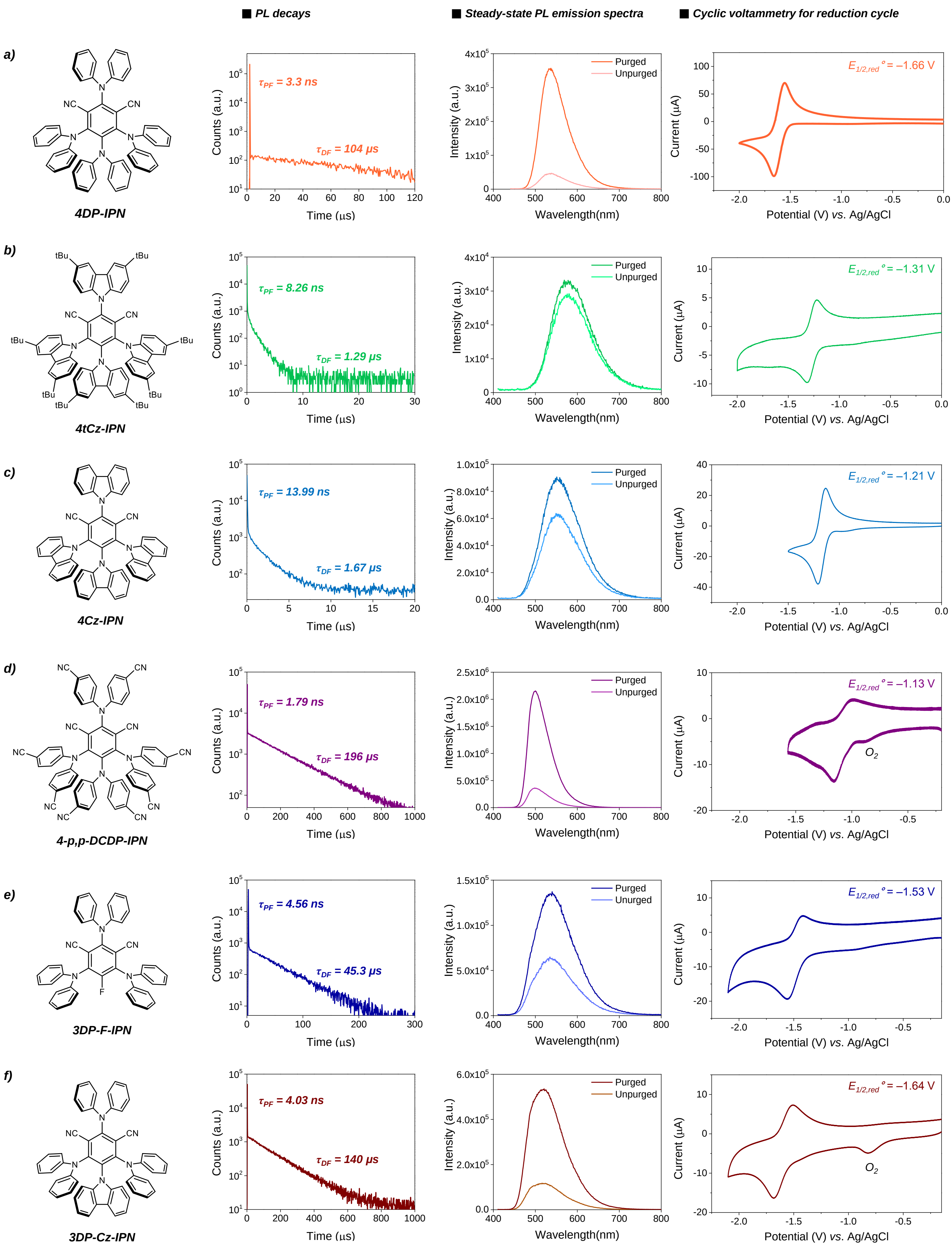
PL decays
Steady-state PL emission spectra
Cyclic voltammetry for reduction cycle
a)
4DP-IPN
τPF = 3.3 ns
τDF = 104 μs
Counts (a.u.)
Time (μs)
Purged
Unpurged
Intensity (a.u.)
Wavelength(nm)
E1/2,red° = –1.66 V
Current (μA)
Potential (V) vs. Ag/AgCl
b)
4tCz-IPN
τPF = 8.26 ns
τDF = 1.29 μs
E1/2,red° = –1.31 V
c)
4Cz-IPN
τPF = 13.99 ns
τDF = 1.67 μs
E1/2,red° = –1.21 V
d)
4-p,p-DCDP-IPN
τPF = 1.79 ns
τDF = 196 μs
E1/2,red° = –1.13 V
O2
e)
3DP-F-IPN
τPF = 4.56 ns
τDF = 45.3 μs
E1/2,red° = –1.53 V
f)
3DP-Cz-IPN
τPF = 4.03 ns
τDF = 140 μs
E1/2,red° = –1.64 V
O2

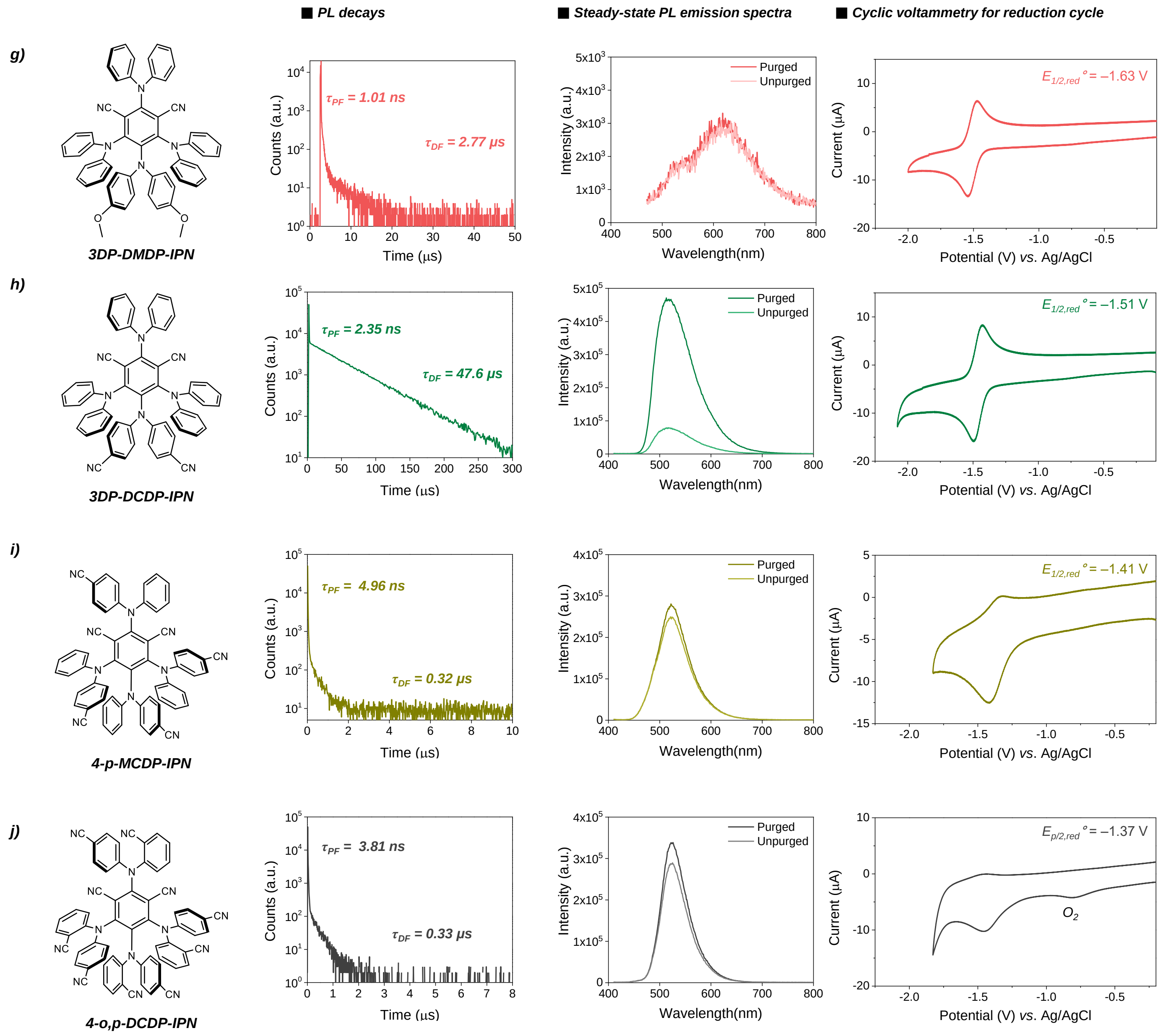

Supplementary Figure 5 Chemical structures, PL decay (left), steady-state PL at RT (center) and CV spectra of reduction cycle (right) of PCs studied in the current work. (**a**) 4DP-IPN, (**b**) 4tCz-IPN, (**c**) 4Cz-PN, (**d**) 4-p,p-DCDP-IPN, (**e**) 3DP-F-IPN, (**f**) 3DP-Cz-IPN, (**g**) 3DP-DMDP-IPN, (**h**) 3DP-DCDP-IPN, (**i**) 4-p-MCDP-IPN and (**j**) 4-o,p-DCDP-IPN. Generally, PL decay and steady-state PL at RT spectra were taken from the degassed solutions of PCs (1.0×10^{-5} M, except for 3DP-DMDP-IPN as 1.0×10^{-4} M due to low PL intensity) in CH_3CN at RT. CV spectra of reduction cycle of PC (2.0×10^{-4} M) (except for 4-o,p-DCDP-IPN as 1.0×10^{-3} M) were obtained in CH_3CN at RT after the degassing process by purging with Ar for 15 min, all potentials were converted to the SCE scale by using $E^0\,(Fc^+/Fc)$ = 0.42 V vs SCE in CH_3CN.

Supplementary Table 3 Photoluminescence quantum yield (Φ_F) of PCs. The Φ_F of PCs relatively measured against coumarin 153 are mainly studied in the current work.

PC	*Solvent*	*Reference*	*Φ_F*	
			N_2 purged	*Air*
4DP-IPN	*DMSO*	-[a]	0.75	0.16
		Fluorescein[b]	0.85	0.14
		Coumarin 153[c]	0.62	0.09
	ACN	-[a]	0.69	0.10
		Fluorescein[b]	0.63	0.08
		Coumarin 153[c]	0.61	0.07
	EA	*Fluorescein*[b]	0.27	0.07
		Coumarin 153[c]	0.26	0.06
4tCz-IPN	*ACN*	*Coumarin 153*[c]	0.05	0.04
4Cz-IPN	*ACN*	*Fluorescein*[b]	0.20	0.14
		Coumarin 153[c]	0.18	0.12
	EA	*Fluorescein*[b]	0.74	0.25
		Coumarin 153 [c]	0.64	0.28
4-p,p-DCDP-IPN	*ACN*	*Coumarin 153* [c]	0.61	0.16
	EA	*Coumarin 153*[c]	0.59	0.10
3DP-F-IPN	*ACN*	*Coumarin 153*[c]	0.17	0.07
3DP-Cz-IPN	*ACN*	*Coumarin 153*[c]	0.52	0.11
3DP-DMDP-IPN	*ACN*	*Coumarin 153*[c]	< 0.01	< 0.01
3DP-DCDP-IPN	*ACN*	*Coumarin 153*[c]	0.43	0.07
4-p-MCDP-IPN	*ACN*	*Coumarin 153*[c]	0.40	0.36
	EA	*Coumarin 153*[c]	0.19	0.17
4-o,p-DCDP-IPN	*ACN*	*Coumarin 153*[c]	0.34	0.29
	EA	*Coumarin 153*[c]	0.26	0.23

The relative Φ_F measurements vs. coumarin 153 are expected to be the most reliable, because (i) the spectral range of emission agrees best with the samples, (ii) the absorption is quite broad, which allows to define an intersection point (used for PL excitation) between sample and reference absorption spectra which are in both close to the plateau of the spectra, so that errors are minimized. [a]Absolute PLQY measurement. Relative PLQY measurement against [b]fluorescein (1.0×10^{-5} M) in 0.1 M NaOH aqueous solution ($\Phi_F = 0.824 \pm 0.049$) or [c]coumarin 153 (1.0×10^{-5} M) in ethanol solution ($\Phi_F = 0.514 \pm 0.031$).[25]

Supplementary Table 4 Photophysical properties of PCs in CH_3CN. The photophysical rate constants were derived by experimental values for τ_{PF}, τ_{DF} and Φ_F (all given in Supplementary Figure 5 and Supplementary Table 3) and the TD-DFT calculated oscillator strength (*f*), the spectral positions for absorption and emission followed by the procedure described in the literature[26] by using equations (25–28) under the assumption, i) $k_{nr,T}$, k_{PH} << k_{RISC} and ii) k_{ISC} >> k_{RISC} and estimating $k_{r,S1}$ via the Strickler-Berg equation,[27,28] equation (25).

PC	E_{0-0} (S_1) (eV)[a]	E_{0-0} (T_1) (eV)[b]	τ_{PF} (ns)	τ_{DF} (μs)	$\lambda_{max,abs}$ (nm)	$\lambda_{max,em}$ (nm)	$f_{S0\rightarrow S1}$ [c]	Φ_F	$k_{r,S1}$ (10^7 s^{-1})	$k_{nr,S1}$ (10^7 s^{-1})	k_{ISC} (10^8 s^{-1})	$k_{r,T1}$ (s^{-1})	$k_{nr,T1}$ (10^3 s^{-1})	k_{RISC} (10^5 s^{-1})	Remark
4DP-IPN	2.45	2.29	3.30	104	469	531	0.0790	0.61	3.0	1.9	2.5	2.5	-	0.60	
									2.1	1.4	2.7	2.6	0	0.84	Unbiased simulation[f] ($k_{nr,T1}$ = 0)
									2.1	0	2.8	2.6	4.2	0.84	Unbiased simulation[f] ($k_{nr,S1}$ = 0)
4tCz-IPN	2.62[d]	2.62[d]	8.26	1.29	440[d]	578[d]	0.0863	0.05	2.4	45	-[e]	-	-	2.0	
4Cz-IPN	2.65	2.60	13.99	1.67	420	551	0.0697	0.18	2.1	9.5	-[e]	-	-	3.7	
4-p,p-DCDP-IPN	2.58	2.41	1.79	196	458	500	0.0964	0.61	4.2	2.7	4.9	-	-	0.41	
3DP-F-IPN	2.65	2.46	4.56	45.3	414	540	0.0192	0.17	0.6	3.0	1.8	-	-	1.4	
3DP-Cz-IPN	2.67	2.46	4.03	140	435	521	0.0355	0.52	1.3	1.2	2.2	-	-	0.7	
3DP-DMDP-IPN	2.34	2.19	1.01	2.77	480	618	0.0065	< 0.01	0.16	16	8.3	-	-	23	
3DP-DCDP-IPN	2.59	2.43	2.35	47.6	455	513	0.0385	0.43	1.6	2.1	3.9	-	-	2.5	
4-p-MCDP-IPN	2.72	2.53	4.96	0.32	434	522	0.0738	0.40	2.7	4.0	1.3	-	-	93	
4-o,p-DCDP-IPN	2.63	2.25	3.81	0.33	464	526	0.0503	0.34	1.9	3.7	2.1	-	-	140	

[a,b]E_{0-0} were extracted from the [a]onset of PL in CH_3CN at RT and [b]onset of gated PL in CH_3CN at 65 K, respectively. The onsets were obtained by tangential method; i.e., the intersection of the tangent, set at the high energy slope of the spectrum, with the x-axis.[29] [c]Oscillator strengths were obtained by TD-DFT calculation. [d]The photophysical properties were referred to literature measured in DMF.[1] [e]Because of i) the approximation conditions and/or ii) the error in the experimental and theoretical (Strickler-Berg equation) determination of k_{ISC}, the rate constants were evaluated as negative values. [f]The evaluation relies on unbiased kinetic modelling, which will be detailed in a forthcoming paper.

Supplementary Note 4. PL decay quenching experiments

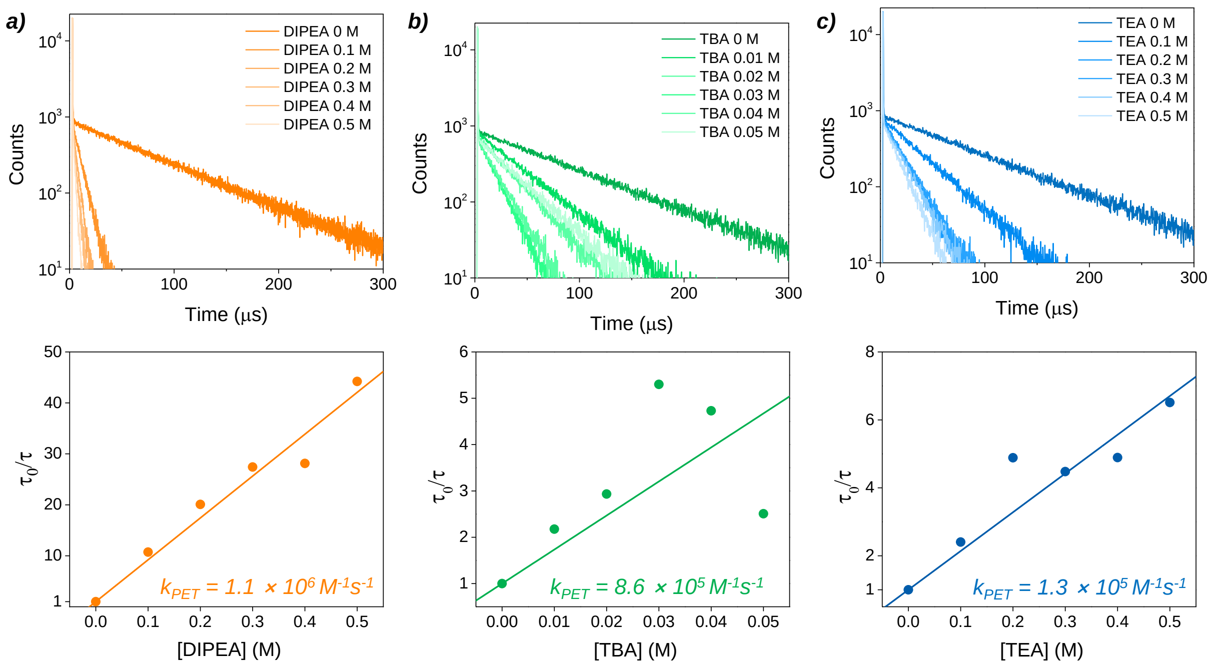

Supplementary Figure 6 Stern-Volmer plots of delayed fluorescence of 4DP-IPN quenched by (**a**) DIPEA, (**b**) TBA and (**c**) TEA in CH_3CN (1.0×10^{-5} M) monitored at λ_{det} = 550 nm.

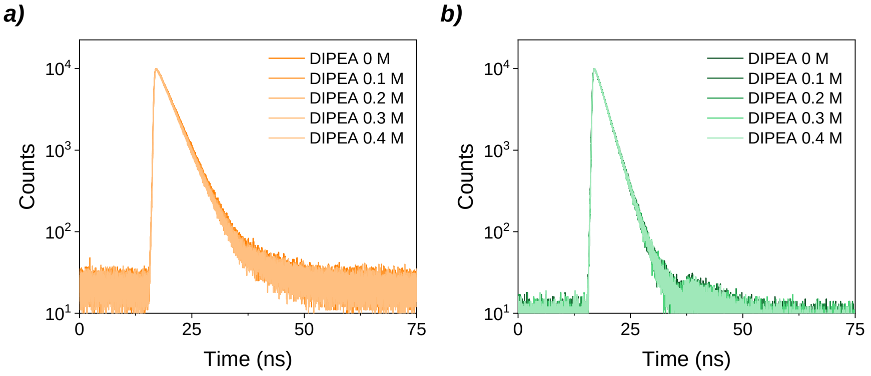

Supplementary Figure 7 Stern-Volmer plots of prompt fluorescence of (**a**) 4DP-IPN and (**b**) 4DP-Me-BN quenched by DIPEA (0 – 0.4 M) in CH_3CN (1.0×10^{-5} M).

Supplementary Note 5. Tertiary amine effect in photoredox reductive dehalogenation

Supplementary Table 5 Screening of conventional tertiary amines in photoredox reductive dehalogenation. Redox potential values are against SCE and referred to literature.[30,31]

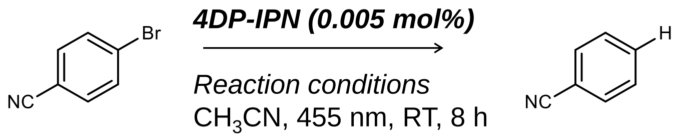

Entry	*Reaction conditions*	*E_{ox}^0*	*Yield (%)*
1	TEA (5.0 equiv.)	0.96[30]	0
2	TEA (10.0 equiv.)		5
3	TBA (5.0 equiv.)	0.88[31]	2
4	TBA (10.0 equiv.)		73
5	DIPEA (5.0 equiv.)	0.68[30]	75
6	DIPEA (10.0 equiv.)		100

Reactions were performed with 4-bromobenzonitrile (0.1 M), 4DP-IPN (0.005 mol%), tertiary amine (10.0 equiv.) in CH_3CN (1 mL) under illumination of two 3W 455 nm LEDs for 8 hours. All solutions were prepared inside glovebox and were degassed by bubbling with Ar. Yields were determined by GC-FID using 1,3,5-trimethoxybenzene as an internal standard.

Supplementary Note 6. Reproducibility test in photoredox reductive dehalogenation

Supplementary Table 6 Reproducibility test for the dehalogenation of 4-bromobenzonitrile.

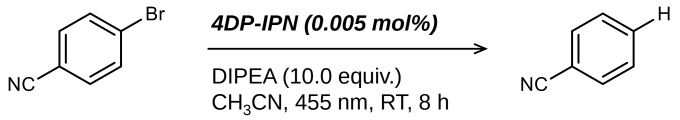

Entry	*LED setup*	*Yield (%)*
1	Setup 1	100
2		97.8
3	Setup 2	96.1
4		100
5	Setup 3	100
6		100
7	Setup 4	98.2
8		99.4

Reactions were performed with 4-bromobenzonitrile (0.1 M), 4DP-IPN (0.005 mol%), DIPEA (10.0 equiv.) in CH_3CN (1 mL) under two 3W 455 nm LEDs for 8 hours. All solutions were prepared inside glovebox and were degassed by bubbling with Ar. Yields were determined by GC-FID using 1,3,5-trimethoxybenzene as an internal standard.

Supplementary Note 7. Generation of radical anion of PCs

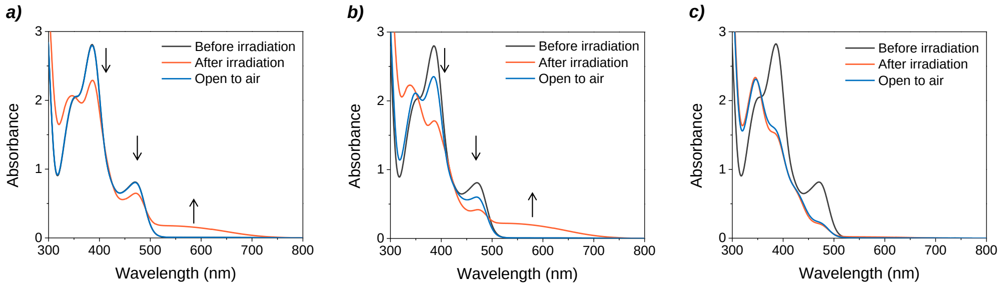

Supplementary Figure 8 UV-Vis absorption of 4DP-IPN / 4DP-IPN$^{•-}$. The generation of 4DP-IPN$^{•-}$ was performed with 4DP-IPN (1.0×10^{-4} M), DIPEA (0.5 M) in CH_3CN under illumination of (**a**) two 3W 515 nm LEDs for 1 min, (**b**) two 3W 455 nm LEDs for 1 min and (**c**) two 3W 455 nm LEDs for 5 min at RT.

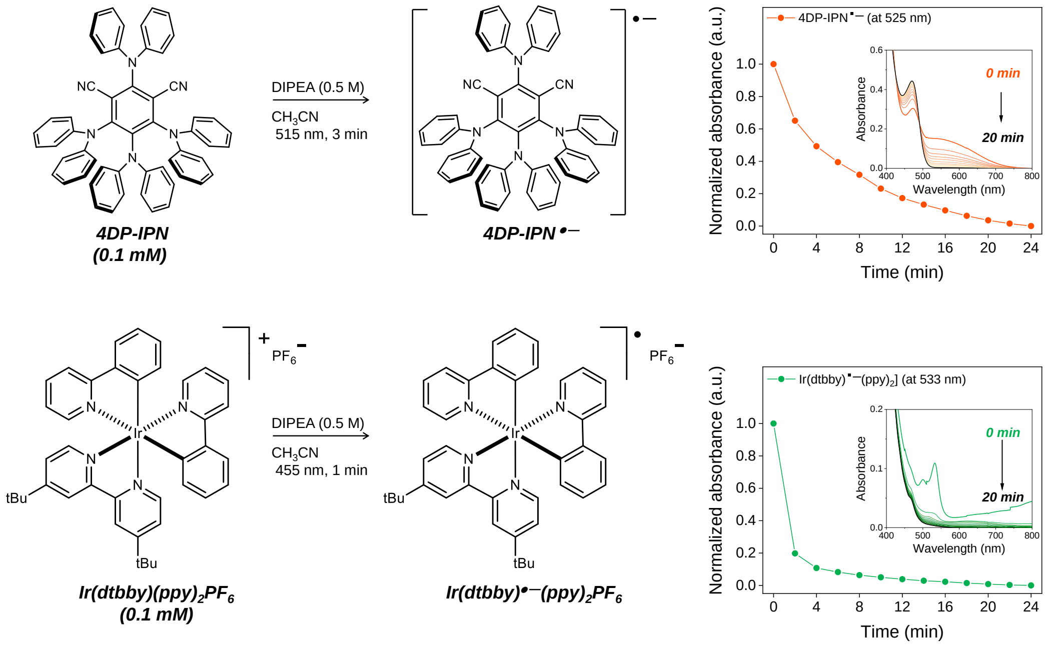

Supplementary Figure 9 Time-dependent changes of the UV-Vis absorbance of 4DP-IPN$^{•-}$ at 525 nm and Ir(dtbby)$^{•-}$(ppy)$_2$PF$_6$ at 533 nm as a function of time. Generally, the PC$^{•-}$ was generated from the degassed mixture solutions of PCs (1.0×10^{-4} M) and DIPEA (0.5 M) in CH_3CN under the illumination of two 3W 515 nm LEDs for 3 min (for 4DP-IPN) or two 3W 455 nm LEDs for 1 min (for Ir(dtbby)(ppy)$_2$PF$_6$) at RT. Changes in the UV-Vis spectrum of freshly generated PC$^{•-}$ were recorded at every 2 min under perfectly dark conditions.

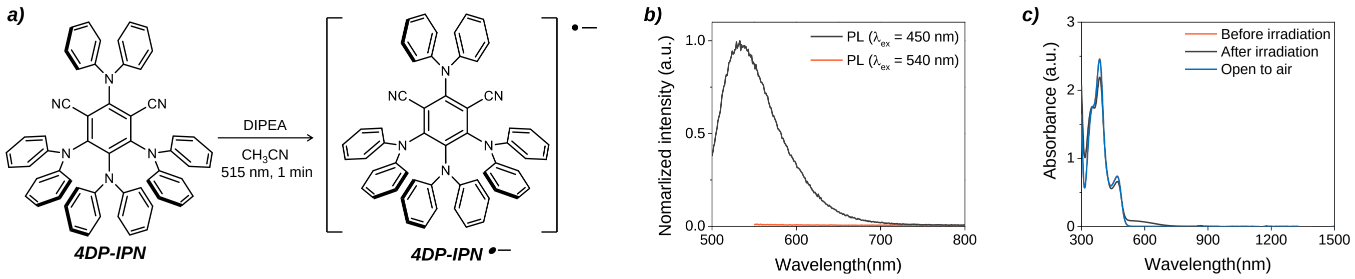

Supplementary Figure 10 (**a**) The 4DP-IPN$^{•−}$ were formed with 4DP-IPN (1.0 × 10^{-4} M), DIPEA (0.5 M) in CH_3CN under illumination of two 3W 515 nm LEDs for 1 min at RT. (**b**) Steady-state PL spectra after generation of 4DP-IPN$^{•−}$. PL of unreacted 4DP-IPN was observed (at λ_{ex} = 450 nm), however, no PL of 4DP-IPN$^{•−}$ was observed (at λ_{ex} = 540 nm). (**c**) UV-Vis absorption of 4DP-IPN / 4DP-IPN$^{•−}$ measurements by near-IR (NIR) region.

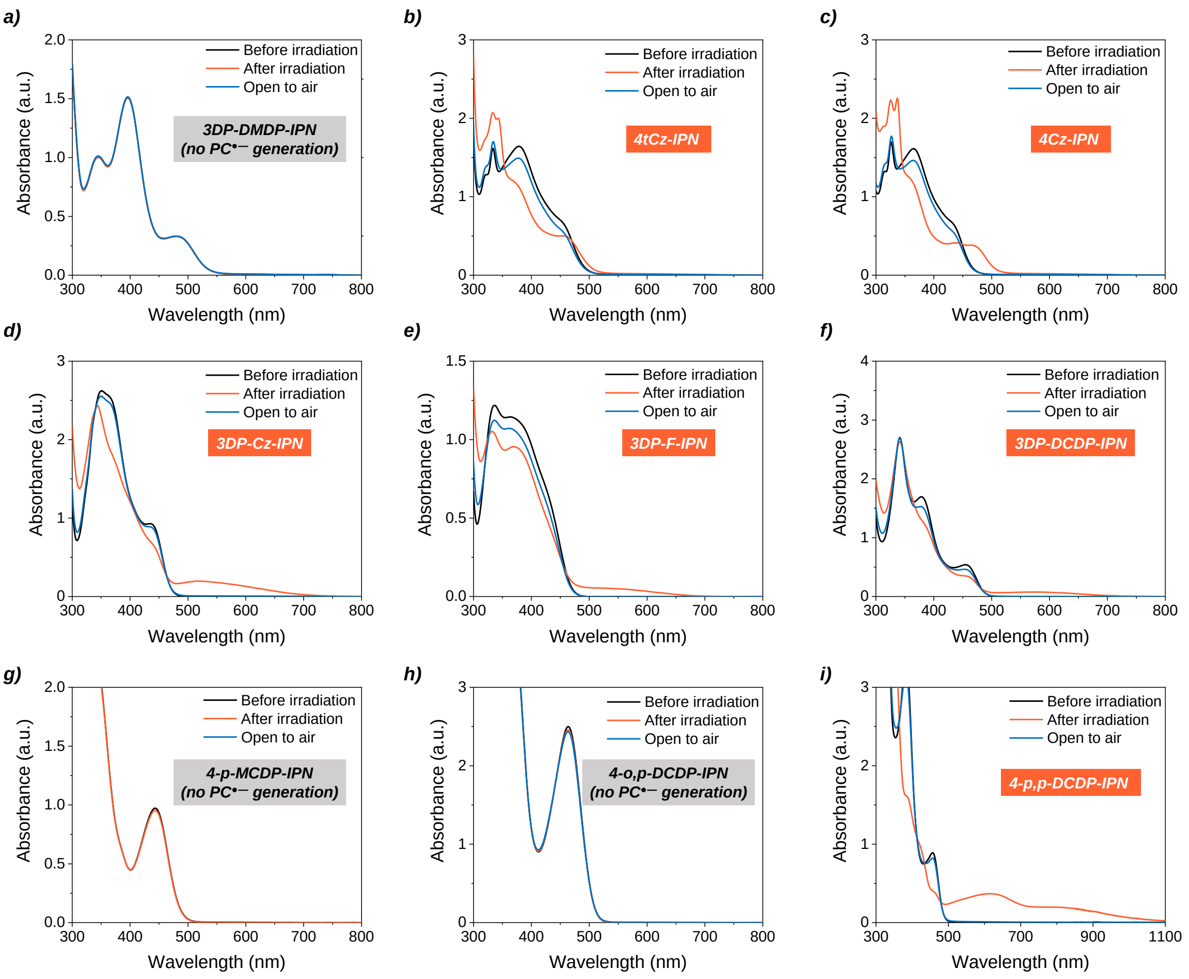

Supplementary Figure 11 Chemical structures and UV-Vis absorption spectra of radical anion generation of selected PCs before irradiation (black line), after irradiation (orange line) and after exposure to air (blue line). Generally, UV-Vis absorption spectra were taken from the degassed mixture solutions of PCs (1.0×10^{-4} M) and DIPEA (0.5 M) in CH_3CN right after illumination of two 3W 455 nm LEDs for 1 min at RT, (**a**) 3DP-DMDP-IPN, (**b**) 4tCz-IPN, (**c**) 4Cz-IPN, (**d**) 3DP-Cz-IPN, (**e**) 3DP-F-IPN, (**f**) 3DP-DCDP-IPN, (**g**) 4-p-MCDP-IPN, (**h**) 4-o,p-DCDP-IPN and (**i**) 4-p,p-DCDP-IPN. All solutions were prepared in a glove box and fully degassed. Most of PCs can be regenerated by exposure to air.

Supplementary Note 8. Validation of ConPET process in photoredox reductive dehalogenation

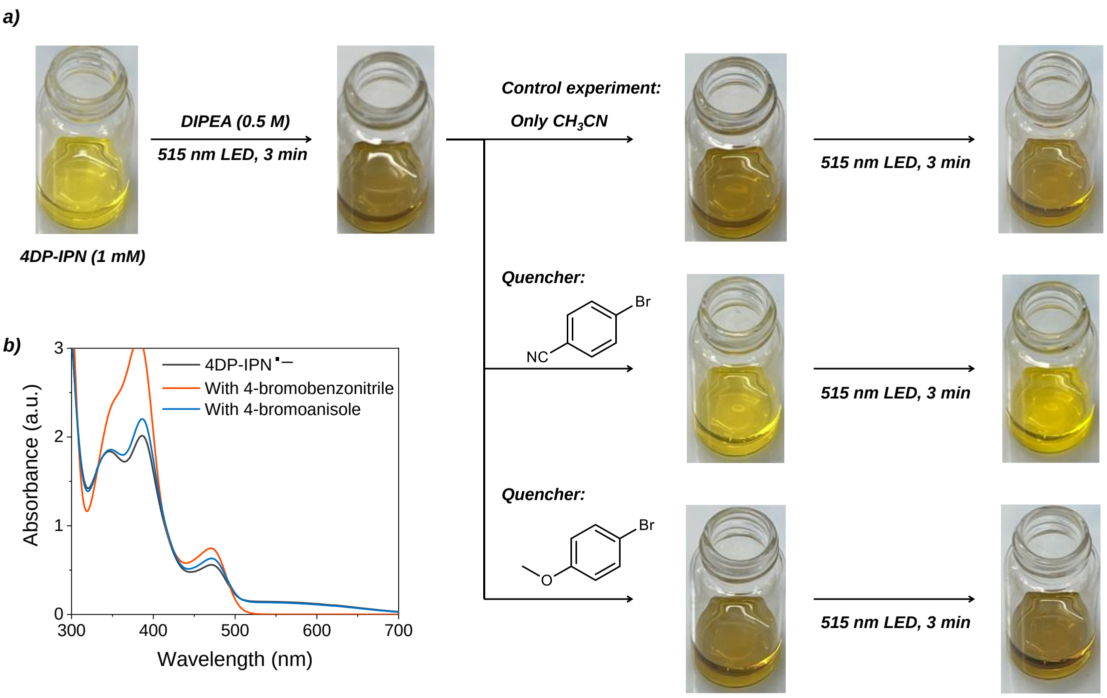

Supplementary Figure 12 Evaluation of PET process in photoredox reductive dehalogenation. (**a**) Inside a glovebox using flame-dried glass vials, the radical anion of 4DP-IPN (1.0×10^{-3} M) was generated with DIPEA (0.5 M) in CH_3CN (2 mL) under illumination of a 3W 515 nm LED for 3 min, aryl bromides solution (0.1 M) as quencher in CH_3CN (0.2 mL) were added. Subsequently, the added solutions were re-illuminated by a 3W 515 nm LED for 3 min. All pictures were taken immediately without any additional delay. (**b**) UV-Vis absorption spectra of 4DP-IPN$^{\bullet-}$ (1.0×10^{-4} M; orange line) in the presence of aryl halide (1.0×10^{-2} M) generated with DIPEA (0.5 M) in CH_3CN under illumination of two 3W 515 nm LEDs for 3 min.

Supplementary Note 9. Computational study: DFT and analytical model

9.1 Analytical model for exciton population of PCs

To analyze quantitatively exciton dynamics of selected PCs, we visualized the excited state concentration of PCs benchmarked by Adachi group.[32] The concentration of S_1 and T_1 can be described by following system of ordinary differential equations (ODE).

$$\frac{d[S_1]}{dt} = k_{RISC}[T_1] - \left(k_{ISC} + k_{r,S_1} + k_{nr,S_1}\right)[S_1] \tag{1}$$

$$\frac{d[T_1]}{dt} = k_{ISC}[S_1] - (k_{RISC} + k_{r,T_1} + k_{nr,T_1})[T_1] \tag{2}$$

where k_{ISC}, k_{RISC}, $k_{r,S1}$, $k_{r,T1}$, $k_{nr,S1}$ and $k_{nr,T1}$ denote the rate constants of intersystem crossing, reverse-intersystem crossing, radiative decay from $^1PC^*$ (S_1), radiative decay from $^3PC^*$ (T_1), non-radiative decay from S_1, and non-radiative decay from T_1, respectively. These coupled nonlinear rate equations enable to trace time-dependent decay of the S_1 and T_1 state generated by single pulsed-photoexcitation. Based on the rate constants obtained from literature,[23,33–37] the ODEs were solved using ODE15s solvers implemented in the Matlab program package.

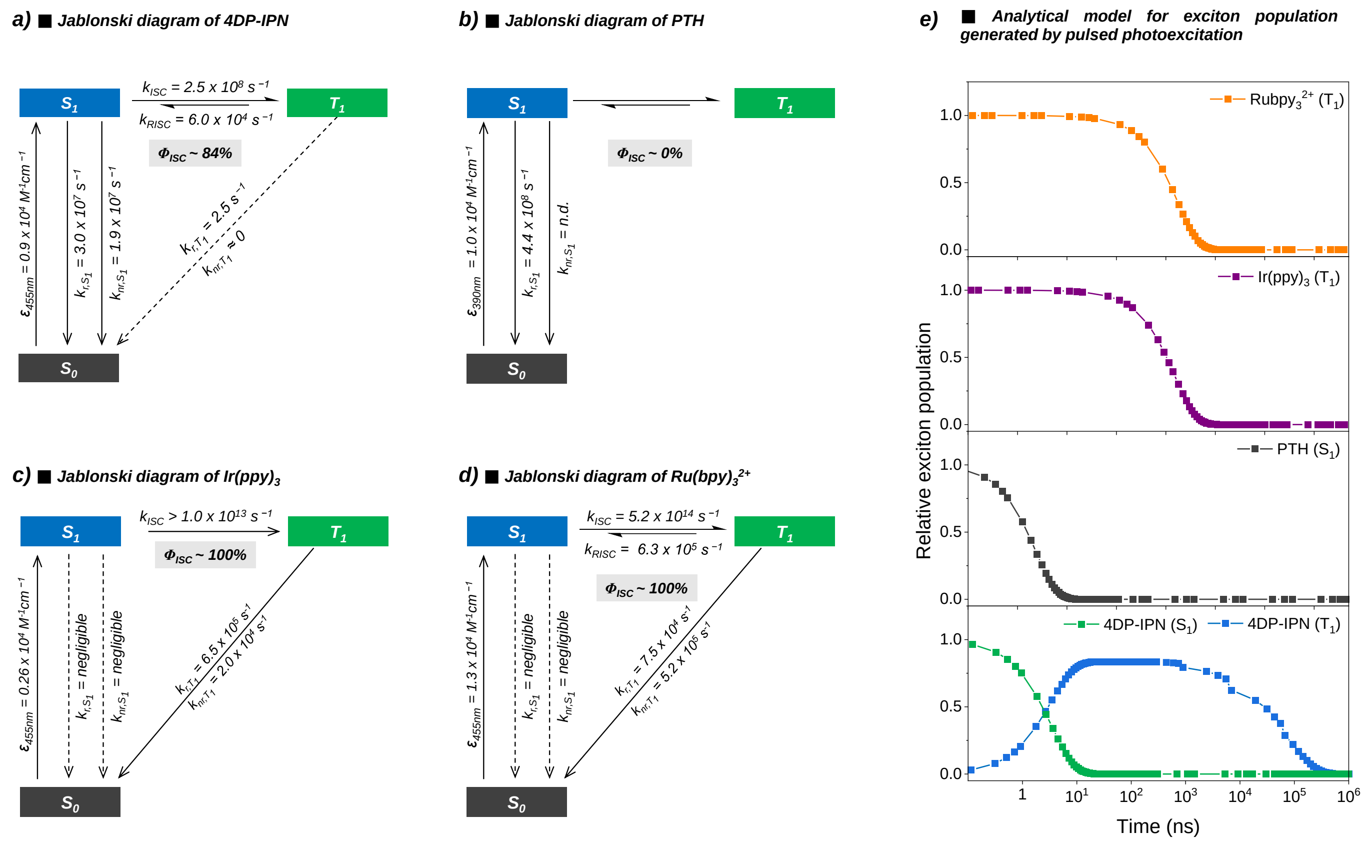

Supplementary Figure 13 Jablonski diagrams of (**a**) 4DP-IPN, (**b**) PTH,[23] (**c**) $Ir(ppy)_3$[35,36] and d) $Ru(bpy)_3^{2+}$[37] based on photophysical rate constants obtained from the current work and referred to literature; The extinction coefficients of 4DP-IPN, $Ir(ppy)_3$ and $Ru(bpy)_3^{2+}$ were measured in CH_3CN in our group. (**e**) the exciton dynamics of selected PCs generated by pulsed photoexcitation.

9.2 TD-DFT results for 4DP-IPN

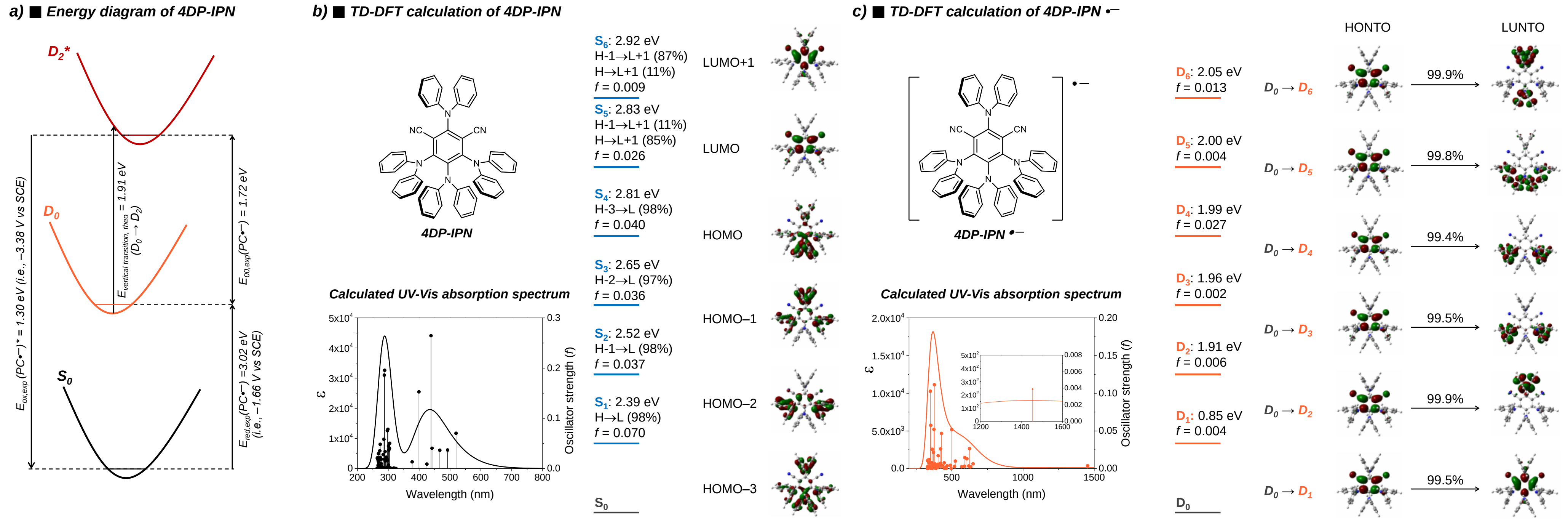

Supplementary Figure 14 (**a**) Energy diagram of 4DP-IPN derived from the experiments and time-dependent (TD) density functional theory (DFT) calculations. TD-DFT results of (**b**) 4DP-IPN and (**c**) 4DP-IPN•−. TD-DFT calculations were performed with the B3LYP functional and 6–311++G* basis set. Using optimized geometries (S_0 and D_0), vertical transition energies were calculated in CH_3CN solution employing polarizable continuum model (PCM). The percentage contributions of each transition were evaluated by $C_i^2 * 2 * 100$ (%) where C_i is excitation coefficient for each vertical transition in closed-shell system, or $\alpha / 2 * 100$ (%) where α is paired coefficient in natural transition orbital (NTO) in open-shell system.

9.3 Kinetic simulation of photoredox reductive dehalogenation

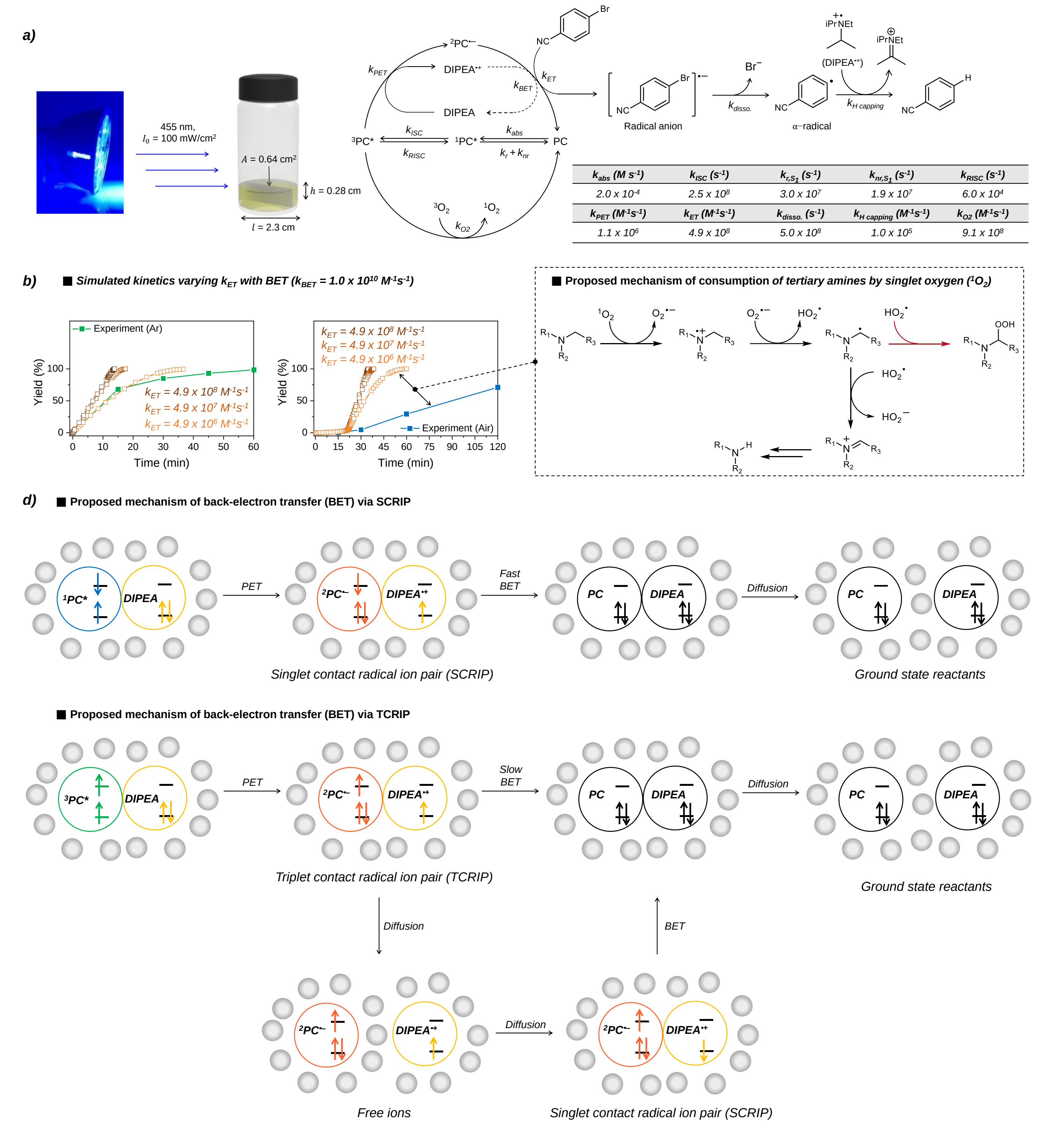

k_{abs} (M s⁻¹)	k_{ISC} (s⁻¹)	k_{r,S_1} (s⁻¹)	k_{nr,S_1} (s⁻¹)	k_{RISC} (s⁻¹)
2.0 x 10⁻⁴	2.5 x 10⁸	3.0 x 10⁷	1.9 x 10⁷	6.0 x 10⁴
**k_{PET} (M⁻¹s⁻¹)**	**k_{ET} (M⁻¹s⁻¹)**	**$k_{disso.}$ (s⁻¹)**	**$k_{H\ capping}$ (M⁻¹s⁻¹)**	**k_{O2} (M⁻¹s⁻¹)**
1.1 x 10⁶	4.9 x 10⁸	5.0 x 10⁸	1.0 x 10⁵	9.1 x 10⁸

Supplementary Figure 15 Kinetic simulation of photoredox reductive dehalogenation. (**a**) Scheme of reaction batch for the reaction and the rate constants for the simulation. All rate constants were obtained from experimental/computational methods in the current work or were referred to literature.[23,33,38–45] (**b**) Comparison between experimental kinetics and simulated kinetics with k_{BET} (1.0×10^{10} $M^{-1}s^{-1}$) varying the k_{ET}. (**c**) Proposed mechanism of depletion of tertiary amines by singlet oxygen ($^1O_2$).[39,40] (**d**) Scheme of back electron transfer via singlet/triplet contact radical ion pair.

To analyze the photocatalytic behavior of 4DP-IPN quantitatively including O_2 tolerance, kinetic simulation of photoredox reductive dehalogenation were performed based on the rate law. To simplify the simulation, we modelled the reaction with several assumptions. Based on following assumptions, we set up our kinetic simulation model. The mass balance equations of each species are listed in Supplementary Table 7 and derivations of each rate constants are described in following section.

1. No back-electron transfer (BET) from 4DP-IPN$^{•−}$ to DIPEA happens because spin-flipping is forbidden, however, BET process was involved in a specific case (see the Supplementary Figure 15). Nevertheless, because the concentration of 4DP-IPN•− and DIPEA$^{•+}$ (~10^{-12} M in the simulation) are much lower than the concentration of the DIPEA (~1 M), the BET rate is far smaller than forward ET rate, $k_{BET} >> k_{PET}$.
2. No ConPET process happens in dehalogenation of 4-bromobenzonitrile. in the case of 4-bromobenzonitrile, electron transfer from 4DP-IPN•− occurs predominately (see the Supplementary Figure 12).
3. Photodegradation of 4DP-IPN is negligible.
4. Internal conversion (IC) is fast compared to our time scale of interest.
5. No triplet-triplet- annihilation (TTA) process.
16. Under air atmosphere, all $^3O_2$ in head space of vial is also considered as being fully diluted in the reaction solution.
7. No side-reaction from superoxide anion ($O_2^{•−}$) or $^1O_2$.; in fact, the reactive O_2 species ($O_2^{•−}$ or $^1O_2$) are very reactive to tertiary amines,[39,40] however, we did not consider the reactivities of DIPEA.

Supplementary Table 7 Kinetic equations for all species in photoredox reductive dehalogenation of 4-bromobenzonitrile

Species	***Mass balance equations***
Ground state PC (S_0)	$\frac{d[S_0]}{dt} = \left(k_{r,S_1} + k_{nr,S_1}\right)[S_1] + k_{ET}[D_0][BBN] + k_{O2}[T_1][\ {}^3O_2] - k_{abs}\left(1 - 10^{-\varepsilon\times l\times [S_0]}\right) + \ k_{BET}[D_0][DIPEARC]$
${}^1PC^*$ (S_1)	$\frac{d[S_1]}{dt} = k_{abs}(1 - 10^{-\varepsilon\times l\times [S_0]}) + k_{RISC}[T_1] - (k_{ISC} + k_{nr,S_1} + k_{nr,S_1})[S_1]$
${}^3PC^*$ (T_1)	$\frac{d[T_1]}{dt} = k_{ISC}[S_1] - k_{RISC}[T_1] - k_{PET}[T_1][DIPEA] - k_{O2}[T_1][\ {}^3O_2]$
${}^2PC^{\bullet-}$ (D_0)	$\frac{d[D_0]}{dt} = k_{PET}[T_1][DIPEA] - k_{ET}[D_0][BBN] - k_{BET}[D_0][DIPEARC]$
4-Bromobenzonitrile (BBN)	$\frac{d[BBN]}{dt} = -k_{ET}[D_0][BBN]$
4-Bromobenzonitrile$^{\bullet-}$ *(BBNRA)*	$\frac{d[BBNRA]}{dt} = k_{ET}[D_0][BBN] - k_{disso.}[BBNRA]$
Benzonitrile radical (BNR)	$\frac{d[BNR]}{dt} = k_{disso.}[BBNRA] - k_{H\ capping}[BNR][DIPEARC]$
DIPEA	$\frac{d[DIPEA]}{dt} = -k_{PET}[T_1][DIPEA] + k_{BET}[D_0][DIPEARC]$
DIPEA radical cation (DIPEARC)	$\frac{d[DIPEARC]}{dt} = k_{PET}[T_1][DIPEA] - k_{H\ capping}[BNR][DIPEARC] - k_{BET}[D_0][DIPEARC]$
Deprotonated DIPEA (DIPEADH)	$\frac{d[DIPEADH]}{dt} = k_{H\ capping}[BNR][DIPEARC]$
Bromide ion (Br^-)	$\frac{d[Br^-]}{dt} = k_{disso.}[BBNRA]$
${}^3O_2$	$\frac{d[\ {}^3O_2]}{dt} = -k_{O2}[T_1][\ {}^3O_2]$
${}^1O_2$	$\frac{d[\ {}^1O_2]}{dt} = k_{O2}[T_1][\ {}^3O_2]$
Benzonitrile (BN)	$\frac{d[BN]}{dt} = k_{H\ capping}[BNR][DIPEARC]$

9.4 Derivation of rate constants from calculated and experimental methods

■ Derivation of k_{abs}

Our LED setups are based on two 3W 455 nm LEDs (totally 6W). To check the power of effective irradiation, photonflux (the number of photon $m^{-2}s^{-1}$) was measured, I_0 = 100 mW/cm^2. The concentration of excited S_1 state can be expressed by following terms of equation (3).[41,42]

$$\frac{d[S_1]}{dt} = \phi \times \frac{A}{V_0 N_A} \times \frac{I_0}{h\nu} \times \varepsilon c l \times F, \quad \text{where } F \text{ is photo kinetic factor}, \quad F = \frac{1-10^{-\varepsilon c l}}{\varepsilon c l} \qquad (3)$$

where ϕ is quantum yield of the transformation from $S_0 \rightarrow S_1$, A is cross-sectional area (0.64 cm^2), V_0 is the reaction volume (1.174 mL), N_A is Avogadro number, h is Planck constant, ν is frequency of the photon, ε is the extinction coefficient of PC (0.9 x 10^4 $M^{-1}cm^{-1}$ in the case of 4DP-IPN), c is the concentration of PC and l is the optical path length (2.3 cm). Because it is tricky to evaluate all the factors affecting photonflux such as a vial's curvature/refractive index, they are not involved in the simulation. It is assumed ϕ is unity, and by replacing c by the concentration of PC in the ground state $[S_0]$, equation (3) can be converted to equation (4).

$$\frac{d[S_1]}{dt} = 2.0 \times 10^{-4}\ \mathrm{Ms^{-1}} \times \left(1 - 10^{-2.3\,cm \times 0.9 \times 10^4\,M^{-1}cm^{-1} \times [S_0]}\right) = k_{abs} \times \left(1 - 10^{-2.1 \times 10^4\,M^{-1} \times [S_0]}\right) \qquad (4)$$

■ Evaluation of k_{PET}

To obtain the rate constant of PET from DIPEA to $^3PC^*$, we measured the decay of delayed fluorescence of 4DP-IPN along with addition of DIPEA. In the Stern-Volmer plot (see the Supplementary Figure 6), a linear relationship was observed and the rate constant was evaluated as 1.1 x 10^6 $M^{-1}s^{-1}$.

■ Evaluation of k_{ET}

Because k_{ET} is one of the rate constants in dark reaction, it is tricky to estimate the rate constants from the direct experiments. Therefore, we evaluated k_{ET} with computation and indirect experimental methods. For computational methods, we used DFT calculations using the Gaussian16 program package.

Because electron transfer to aryl bromides is based on a stepwise mechanism,[43,44] according to Marcus–Savéant theory, the k_{ET} can be calculated by the following equation (5).

$$k_{\mathrm{ET}} = Z\,\exp(-\frac{\Delta G^{\ddagger}}{RT}) \qquad (5)$$

$$\Delta G^{\ddagger} \approx \frac{(\lambda+\Delta G^{\circ})^2}{4\lambda} = \frac{\lambda}{4}\left(1+\frac{\Delta G^{\circ}}{\lambda}\right)^2 \qquad (6)$$

where Z is the pre-exponential factor, $\Delta G^{\ddagger}$ is the activation energy of the reaction, ΔG° is the driving force of the reaction, and λ is the reorganization energy. In the case of electron transfer via a stepwise mechanism, the activation energy can be approximated regarding the driving force and reorganization energy.[45] In other words, to obtain k_{ET}, only three terms are required; the reorganization energy, the driving force and the pre-exponential factor. The detailed derivations for the three terms are followed.

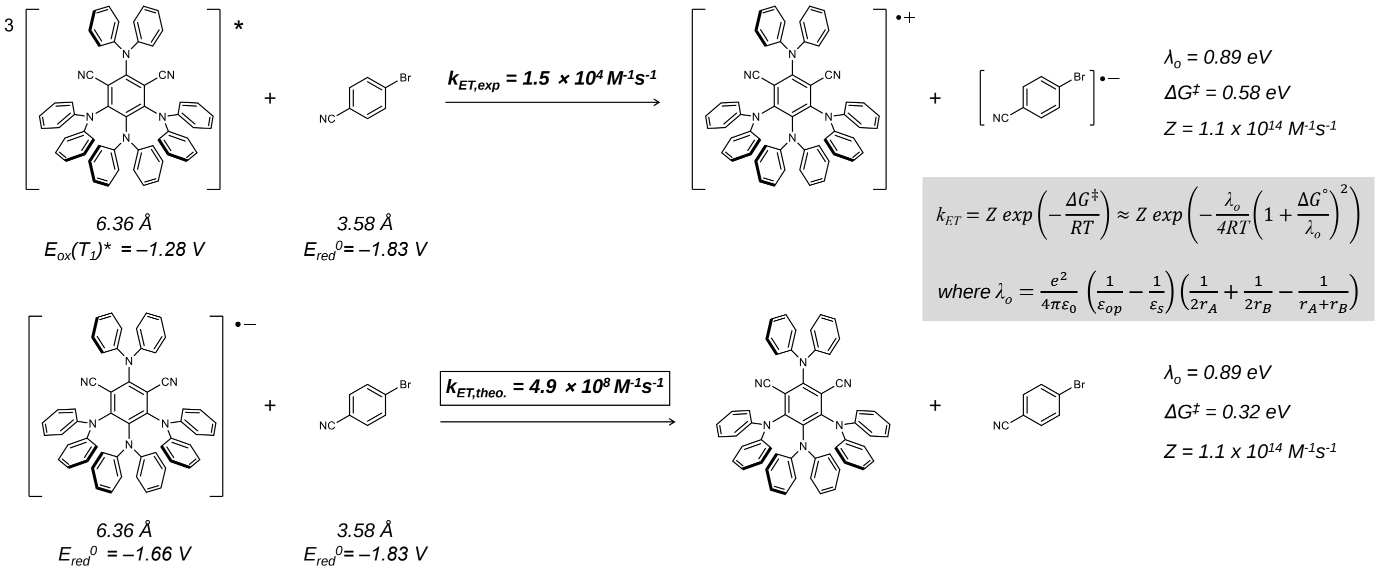

Supplementary Figure 16 Summary of the experimental and calculated values to obtain the rate constant, k_{ET}, for electron transfer from 4DP-IPN$^{\bullet-}$ to 4-bromobenzonitrile; molecular radii of each species are indicated under the chemical structures, Z is the pre-exponential factor, $\Delta G^{\ddagger}$ is the activation energy of the reaction, and λ_0 is the reorganization energy.

■ **Evaluation of reoranization energy, λ.**

Because there is no significant change of molecular structure of 4DP-IPN in the electron transfer process, internal reorganization, λ_i, is fairly negligible for 4DP-IPN, therefore, λ can be approximated by the external solvent reorganization energy, λ_o, $\lambda = \lambda_i + \lambda_o \approx \lambda_o$.[45]

$$\lambda_o = \frac{e^2}{4\pi\varepsilon_0}\left(\frac{1}{\varepsilon_{op}} - \frac{1}{\varepsilon_s}\right)\left(\frac{1}{2r_A} + \frac{1}{2r_B} - \frac{1}{r_A+r_B}\right) \quad (7)$$

where e is the electron charge. ε_0 is the vacuum permittivity, ε_{op}, ε_s are the solvent optical and static dielectric constants, respectively. The r_s are the radii of the equivalent spheres of the subscript species. We calculated the radii of each speices (Supplementary Figure 16) with DFT calculations using B3LYP/6-311G*_PCM(=CH_3CN).

■ **Evaluation of driving force, Δ*G*°.**

The driving force, $\Delta G°$, for the electron transfer were calculated from the standard potentials of the electron donor and electron acceptor redox potential coluples in the ground state and the energy of the excited state, $E_{0\text{-}0}$, of 4DP-IPN using the Rehm-Weller equation;[23] in equations (8–9), Coulombic term is omitted as it is normally small (< 0.1 eV) in polar solvents.

$$\Delta G_{PET}° = -F\,[E_{red}(Sub/Sub^{\bullet -}) - E^{*}_{ox}(PC^{\bullet +}/{}^{3}PC)]$$ for PET from 4DP-IPN (T_1) to 4-bromobenzonitrile (8)

$$\Delta G_{ET}° = -F\,[E_{red}(Sub/Sub^{\bullet -}) - E_{red}(PC/PC^{\bullet -})]$$ for ET from 4DP-IPN$^{\bullet -}$ to 4-bromobenzonitrile (9)

Considering the ground state reduction potential of 4-bromobenzonitrile ($E_{red}^{0} = -1.83\ V$),[46] both electron transfer from 4DP-IPN (T_1) ($E_{ox}^{*} = -1.28\ V$) and 4DP-IPN$^{\bullet -}$ ($E_{red}^{0} = -1.66\ V$) are endogonic. However, it is notable that the evaluation of driving force ($-\Delta G_{PET}$) for the PET is still unclear, even $E_{0\text{-}0}$ energies for PCs might be derived from intersect between normalized spectra of reduced UV-Vis absorption and PL emission, onset of UV-Vis absorption and onset/maxima of PL emission.[47] Also, the method of E_{red}^{0} evaluation is diverse as followed by onset, half-peak or peak potential of CV waves. These inaccuracies can be up to 0.5 eV or more making our evaluation of rate constants over-/underestimated.

■ Evaluation of pre-exponential factor, *Z*.

Because the size difference between the volume 4DP-IPN (T_1) and 4DP-IPN$^{•−}$ is negligible, the pre-exponential factor, *Z*, for electron transfer from 4DP-IPN$^{•−}$ to 4-bromobenzonitrile can be obtained from the rate constant for PET from 4DP-IPN (T_1) to 4-bromobenzonitrile (k_{ET} = 1.5 × 10^4 $M^{-1}s^{-1}$, Supplementary Figure 16). The equations (7–9) can provide the pre-exponential factor for PET from 4DP-IPN (T_1) to 4-bromobenzonitrile using the reorganization energy and the driving force, finally, pre-exponential factor, *Z*, is 1.1 × 10^{14} $M^{-1}s^{-1}$. This pre-exponential factor value should be same for the electron transfer from 4DP-IPN$^{•−}$ to 4-bromobenzonitrile also.

Finally, using equation (5) based on the derived reorganization energy, λ, driving force, $\Delta G°$, and pre-exponential factor, *Z*, we evaluated k_{ET} as 4.9 × 10^8 $M^{-1}s^{-1}$. However, it is notable that the previously reported pre-exponential factor, *Z*, is ~10^{11} $M^{-1}s^{-1}$,[48,49] thus, our k_{ET} might be overestimated. As mentioned above, this discrepancy might be from the inaccurate extraction of values from experimental results.

■ Evaluation of $k_{H\ capping}$

Because aryl radical is easy to be reduced and hydrogen atom transfer reaction is one of fastest reactions, it is reasonable to estimate that hydrogen capping is nearly diffusion-limited. Indeed, rate constants of αC-H deprotonation of simple tertiary amine raical cations have been known to be very fast ranging from 10^5 ~ 10^8 $M^{-1}s^{-1}$;[23] therefore, it is accetable to estimate the deprotonaion rate constant of DIPEA radical cations as 10^5 $M^{-1}s^{-1}$.

■ Evaluation of k_{O2}.

In the dehalogenation reaction, O_2 quenching pathway can be (1) energy transfer from T_1 to $^3O_2$ generating $^1O_2$, (2) electron transfer from T_1 to $^3O_2$ generating $O_2^{•−}$ and (3) electron transfer from PC$^{•−}$ to O_2. From the Stern-Volmer plot (Supplementary Figure 17), we can easily evaluate pathway (1) and (2), however, it is hard to experimentally evaluate pathway (3). Therefore, we assumed pathway (3) is negligible because pathway (1) and (2) is much faster than the generation of 4DP-IPN$^{•−}$; despite only pathway (1) is depicted as being the main contributor to $^3O_2$ quenching process in Supplementary Figure 15, both O_2 quenching (1) and (2) are involved in the actual dehaloenation reaction.

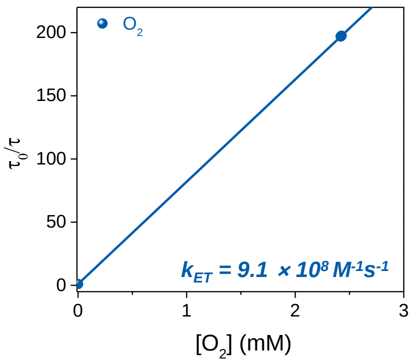

Supplementary Figure 17 Stern-Volmer relationship of PL decays of 4DP-IPN with air. Because the sample was fully exposed to air, it could be assumed that the sample was saturated with air; O_2 solubility to CH_3CN is 2.42 x 10^{-3} M at 25 °C and 1.0 atm of air.[50]

■ **Evaluation of $k_{r,S1}$, $k_{nr,S1}$, k_{ISC} and k_{RISC}** (see Supplementary Table 4)

The general TADF kinetics can be obtained from the differential equations for the singlet and triplet excited state (S_1, T_1) deactivation.

$$\frac{d[S_1]}{dt} = -k_S \cdot [S_1] + k_{RISC} \cdot [T_1] + \alpha \cdot I \quad (10)$$

$$\frac{d[T_1]}{dt} = k_{ISC} \cdot [S_1] - k_T \cdot [T_1] \quad (11)$$

where $k_S = k_{r,S1} + k_{nr,S1} + k_{ISC}$, $k_T = k_{r,T1} + k_{nr,T1} + k_{RISC}$, I is the intensity of excitation, and α is the absorption coefficient; the solutions are

$$S_1(t) = \frac{S_1(0)}{A_2 - A_1}[(A_2 - k_S)\,exp(-A_1 t) + (k_S - A_1)\,exp(-A_2 t)] \quad (12)$$

$$T_1(t) = \frac{S_1(0) \cdot k_{ISC}}{A_2 - A_1}[exp(-A_1 t) - exp(-A_2 t)] \quad (S13)$$

and the total intensity is obtained as

$$I(t) = k_{r,S_1} S_1(t) + k_{r,T_1} T_1(t) \quad (15)$$

$$I(t) = \frac{S_1(0)}{A_2 - A_1}\left[\left(k_{r,S_1}(A_2 - A_S) + k_{r,T_1} k_{ISC}\right) exp(-A_1 t) + \left(k_{r,S_1}(k_S - A_1) - k_{r,T_1} k_{ISC}\right) exp(-A_2 t)\right] \quad (16)$$

The exponents $A_{1,2}$ (which correspond to the reciprocal values of the prompt/delayed PL lifetime constants, i.e., $\tau_{PF,DF}^{-1}$, respectively) are given by

$$A_{1,2} = \frac{1}{2}\left(k_S + k_T \mp (k_T - k_S)\sqrt{1 + 4 \cdot k_{ISC} k_{RISC}/(k_T - k_S)^2}\right) \quad (17)$$

The total PL quantum yield Φ_{PL} is given as the sum of fluorescence and phosphorescence quantum yields ($\Phi_{PL} = \Phi_F + \Phi_{PH}$), where Φ_F consists of a prompt fluorescence (Φ_{PF}) and delayed fluorescence (Φ_{DF}) part; the prompt part is defined by

$$\Phi_{PF} = \frac{k_{r,S_1}}{k_S} \tag{18}$$

In the presence of large number of TADF cycles, the total Φ_F (along with Φ_{PH}) under steady state is obtained as,[51]

$$\Phi_F = \Phi_{PF}\frac{1}{(1-\eta_{ISC}\cdot\eta_{RISC})} \tag{19}$$

where $\eta_{ISC} = k_{ISC}/k_S$ and $\eta_{RISC} = k_{RISC}/k_T$ are the efficiencies for ISC and RISC, respectively. Similarly, Φ_{PH} is obtained as

$$\Phi_{PH} = \frac{\eta_{ISC}k_{PH}}{k_T(1-\eta_{ISC}\cdot\eta_{RISC})} \tag{20}$$

The condition for strong TADF emitters translates to $k_{RISC} >> k_{r,T1}$, $k_{nr,T1}$, so that $\eta_{RISC} \approx 1$; the phosphorescence lifetime of 4DP-IPN at 65 K is about 407 ms, therefore, the sum of $k_{r,T1}$ and $k_{nr,T1}$ is about 2.5 s^{-1} at 65 K. This simplifies equation (19) to

$$\Phi_F = \frac{\Phi_{PF}}{(1-\eta_{ISC}\cdot\eta_{RISC})} \approx \frac{\Phi_{PF}}{(1-\eta_{ISC})} = \frac{k_S\Phi_{PF}}{(k_{r,S_1}+k_{nr,S_1})} = \frac{k_{r,S_1}}{(k_{r,S_1}+k_{nr,S_1})} \tag{21}$$

Furthermore, for TADF compounds with a non-negligible $\Delta E_{ST} \gtrsim 0.1$ eV (which is indeed the case for the 4DP-IPN as discussed further up), RISC is much smaller than ISC; i.e., $k_{RISC} << k_{ISC}$. Under these conditions, with a Taylor expansion ($y = \sqrt{1+x} \approx 1 + \frac{x}{2}$ for x << 1), the solutions for $A_{1,2}$ simplify to

$$A_1 = {\tau_{PF}}^{-1} = \frac{1}{2}\left(k_S + k_T - (k_T - k_S)\sqrt{1 + 4\cdot k_{ISC}k_{RISC}/(k_T-k_S)^2}\right) = k_S - \frac{k_{ISC}k_{RISC}}{k_T-k_S} \approx k_S \tag{22}$$

$$A_2 = {\tau_{DF}}^{-1} = \frac{1}{2}\left(k_S + k_T + (k_T - k_S)\sqrt{1 + 4\cdot k_{ISC}k_{RISC}/(k_T-k_S)^2}\right) = k_T\left(1 - \frac{k_{ISC}k_{RISC}}{k_Tk_S}\right) \approx k_{RISC}(1-\eta_{ISC}) \tag{23}$$

Finally, the radiative rate constant k_{r,S_1} can be estimated from the Strickler-Berg formula, which in its simplified form reads,[27,28]

$$k_{\mathrm{r},S_1,\mathrm{SB}} = 0.667(s^{-1}cm^2)\frac{E^3_{\mathrm{F,vert}}}{E_{\mathrm{A,vert}}}n^2f = 4.34\cdot10^7(s^{-1}\mathrm{eV}^{-2})\frac{E^3_{\mathrm{F,vert}}}{E_{\mathrm{A,vert}}}n^2f \tag{24}$$

where f is the TD-DFT calculated oscillator strength of vertical absorption, n is the refractive index of solvent and E is the energy of vertical absorption and emission respectively for the lowest energetic CT transition.

In summary, the photophysical rate constants of PCs in jablonski diagram were evaluated by experimental (i.e., prompt/delayed fluorescence decays) and computational method (i.e., TD-DFT), which each relation is simplified to

$$k_{\mathrm{r},S_1} = 4.34 \cdot 10^7 (s^{-1}\mathrm{eV}^{-2}) \frac{E^3_{\mathrm{F,vert}}}{E_{\mathrm{A,vert}}} n^2 f \tag{25}$$

$$k_{\mathrm{nr},S_1} = \frac{k_{r,S_1}}{\Phi_F} - k_{r,S_1} = k_{r,S_1}\left(\frac{1}{\Phi_F} - 1\right) \tag{26}$$

$$k_{\mathrm{ISC}} = {\tau_{PF}}^{-1} - \frac{k_{r,S_1}}{\Phi_F} \tag{27}$$

$$k_{\mathrm{RISC}} = \frac{{\tau_{DF}}^{-1}}{1 - k_{ISC}\tau_{PF}} \tag{28}$$

where f, n, E, Φ_F, τ_{PF}, and τ_{DF} have been defined earlier.

9.5 Matlab code

Matlab code is described as below for exciton population of PCs and kinetic simulation of reductive dehalogenations. As exemplified by 4DP-IPN case, the Matlab codes of kinetic simulation are described. Our Mathlab script was composed with simple rate laws, therefore, to calculate exciton population and reductive dehalogenation, one can easily change initial concentrations in Runfile script and/or each rate constant in Function script as according to the calculational needs.

■ Runfile script

```
clear
clc

%Define initial concentrations
C0 = [0.005, 0, 0, 0, 0.1, 0, 0, 0, 1, 0, 0, 0, 0, 0];
% = [S0, S1, T1, D0, Bromobenzonitrile, Bromobenzonitrile radical anion, Benzonitrile radical, Bromide ion, DIPEA, DIPEA radical cation, Deprotonated DIPEA, O2, OR2, Product]
%0.005 = 5 mM = 5 mol%; %0.03 mol% = 0.00003; % 0.16M (under air atmosphere) & 0.76M (under oxygen atmosphere).

%Define time span
tspan = [0,1000];

%Run ODE solver (another option: ode23s)
[t, y] = ode15s(@Function_name, tspan, C0);
```

■ Function script

```
function dC = Photoredox_reductive_dehalogenation_mechanism(t, C)

    % Photoredox reductive dehalogenation_Reductive quenching cycle
    S0 = C(1);              % Ground state PC
    S1 = C(2);              % Singlet excited state PC
    T1 = C(3);              % Triplet excited state PC
    D0 = C(4);              % PC radical anion
    BBN = C(5);             % 4-Bromobenzonitrile
    BBNRA = C(6);           % 4-Bromobenzonitrile radical anion
    BNR = C(7);             % Benzonitrile radical
    Br = C(8);              % Bromide ion
    DIPEA = C(9);           % DIPEA
    DIPEARC = C(10);        % DIPEA radical cation
    DIPEADH = C(11);        % Deprotonated DIPEA
    O2 = C(12);             % Oxygen
    OR2 = C(13);            % Reactive oxygen species
    BN = C(14);             % Benzonitrile product

    %Rate constants
    k1 = 2.0e-4;            % Photoexcitation_455nm_6W LED; photonflux (3.8e-3 mol m-2 s-1), (extinction coefficient of 4DP-IPN: 0.9e4 cm-1M-1 from measurement in CH3CN)
    k2 = 2.5e8;             % Intersystem crossing from singlet to triplet excited state PC (evaluated in the current work)
    k3 = 3.0e7;             % Radiative decay from singlet excited state PC (evaluated in the current work)
    k4 = 1.9e7;             % Nonradiative decay from singlet excited state PC (evaluated in the current work)
    k5 = 6.0e4;             % Reverse intersystem crossing from triplet to singlet excited state PC (evaluated in the current work)
    k6 = 2.5;               % Radiative decay from triplet excited state PC (evaluated in the current work)
    k7 = 0;                 % Nonradiative decay from triplet excited state PC (evaluated in the current work)
```

```
k8 = 1.1e6;        % PET rate constant from DIPEA to triplet excited state PC (evaluated in the current work)
k9 = 4.9e8;        % Electron transfer from PC radical anion to 4-bromobenzonitrile (computationally evaluated in the current work)
k10 = 5e8;         % Bond dissociation of 4-bromobenzonitrile radical anion (from reference, J. Am. Chem. Soc. 126, 16051-16057 (2004).)
k11 = 1e5;         % Hydrogen capping (from reference, Chem. Rev. 116, 1007-10166 (2016).)
k12 = 9.1e8;       % Energy/electron transfer from triplet excited state PC to oxygen (evaluated in the current work)
k13 = 0;           % Back electron transfer from D0 to DIPEARC
k14 = 0;           % Back electron transfer from Bromobenzonitrile radical anion to DIPEA radical cation

%Rate laws
r1 = k1*(1-10^(-0.9e4*S0*2.3));      % Photoexcitation quantum yield 100%; optical length 2.3 cm (vial inner length); batch surface (0.6 cm2)
r2 = k2*S1;                          % Intersystem crossing from singlet to triplet excited state PC
r3 = k3*S1;                          % Radiative decay from singlet excited state PC
r4 = k4*S1;                          % Nonradiative decay from singlet excited state PC
r5 = k5*T1;                          % Reverse intersystem crossing from triplet to singlet excited state PC
r6 = k6*T1;                          % Radiative decay from triplet excited state PC (negligible)
r7 = k7*T1;                          % Nonradiative decay from triplet excited state PC (negeligible)
r8 = k8*T1*DIPEA;                    % PET rate from DIPEA to triplet excited state PC
r9 = k9*D0*BBN;                      % Electron transfer from PC radical anion to 4-bromobenzonitrile
r10 = k10*BBNRA;                     % Bond dissociation of 4-bromobenzonitrile radical anion
r11 = k11*BNR*DIPEARC;               % H atom capping to benzonitrile radical
r12 = k12*T1*O2;                     % Energy/electron transfer from triplet excited state PC to oxygen
r13 = k13*D0*DIPEARC;                % Back electron transfer from D0 to DIPEARC
r14 = k14*DIPEARC*BBNRA;             % Back electron transfer from Bromobenzonitrile radical anion to DIPEA radical cation

%Mass balances
dS0 = -r1+r3+r4+r6+r7+r9+r12+r13;    % Ground state PC
dS1 = r1+r5-r2-r3-r4;                % Singlet excited state PC
dT1 = r2-r5-r6-r7-r8-r12;            % Triplet excited state PC
```

```
dD0 = r8-r9-r13;                     % PC Radical anion
dBBN = -r9+r14;                      % 4-Bromobenzonitrile
dBBNRA = r9-r10-r14;                 % 4-Bromobenzonitrile radical anion
dBNR = r10-r11;                      % Benzonitrile radical
dBr = r10;                           % Bromide ion
dDIPEA = -r8+r13+r14;                % DIPEA
dDIPEARC = r8-r11-r13-r14;           % DIPEA radical cation
dDIPEADH = r11;                      % Deprotonated DIPEA
dO2 = -r12;                          % Oxygen
dOR2 = r12;                          % Reactive oxygen species
dBN = r11;                           % Benzonitrile product

%Assign output variables
dC(1,:) = dS0;
dC(2,:) = dS1;
dC(3,:) = dT1;
dC(4,:) = dD0;
dC(5,:) = dBBN;
dC(6,:) = dBBNRA;
dC(7,:) = dBNR;
dC(8,:) = dBr;
dC(9,:) = dDIPEA;
dC(10,:) = dDIPEARC;
dC(11,:) = dDIPEADH;
dC(12,:) = dO2;
dC(13,:) = dOR2;
dC(14,:) = dBN;
```

Supplementary Note 10. Photodegradation behavior of cyanoarenes-based PC

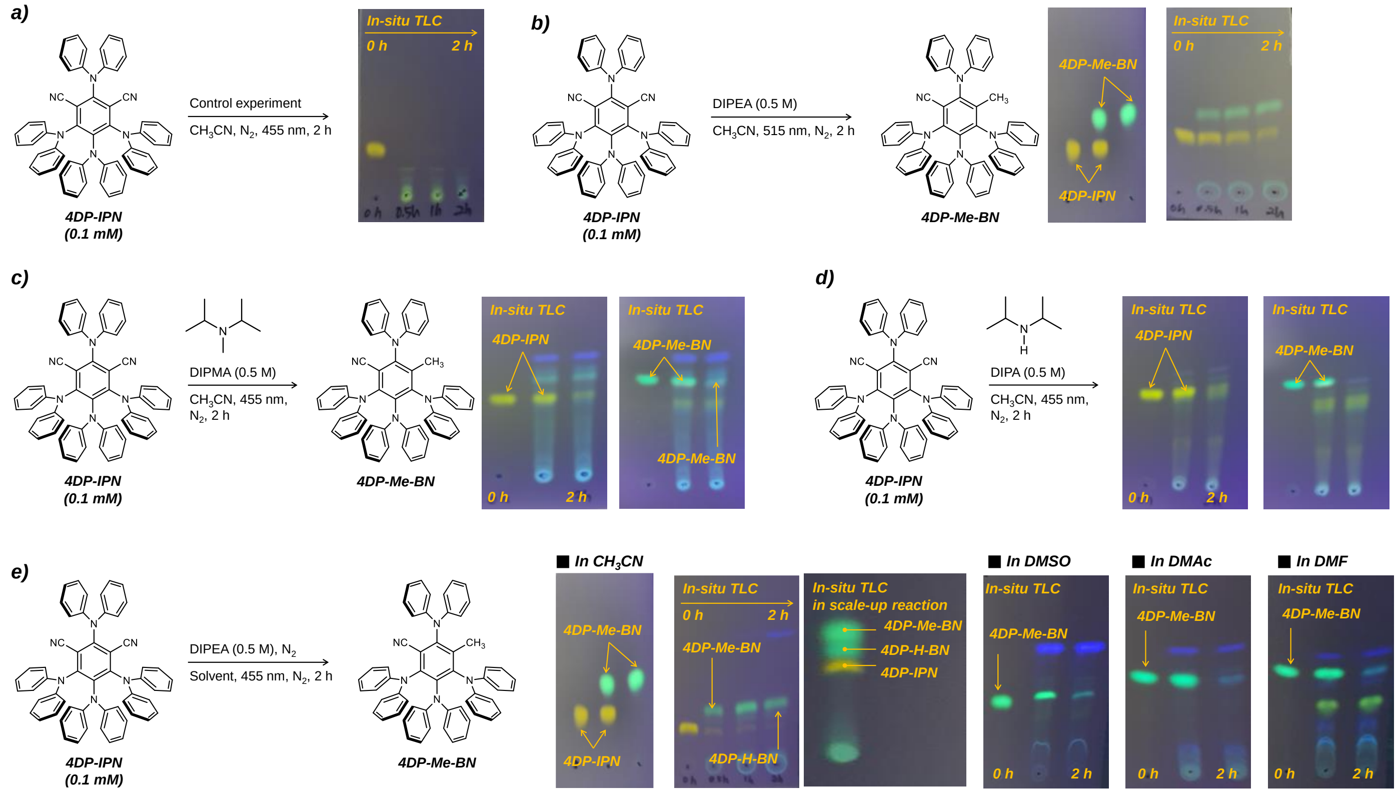

Supplementary Figure 18 Estimation of photodegradation behaviour of 4DP-IPN. Reaction condition was the same described above for the general procedure, 4DP-IPN (1.0×10^{-4} M), DIPEA (0.5 M) in CH_3CN (1 mL). Reactions were monitored by thin layer chromatography (TLC) (EA:hexanes, 1:4 v/v). (**a**) Photodegradation behaviour of 4DP-IPN was monitored without DIPEA as a control experiment. (**b**) In presence of DIPEA, the photodegradation experiments were carried out under illumination of two 3W 515 nm LEDs. (**c,d**) The photodegradation experiments were carried out under illumination of two 3W 455 nm LEDs in the presence of (**c**) diisopropylmethylamine (DIPMA) and (**d**) diisopropylamine (DIPA) as a reducing agent instead of DIPEA. (**e**) The photodegradation experiments were carried out under illumination of two 3W 515 nm LEDs in various organic solvents at RT.

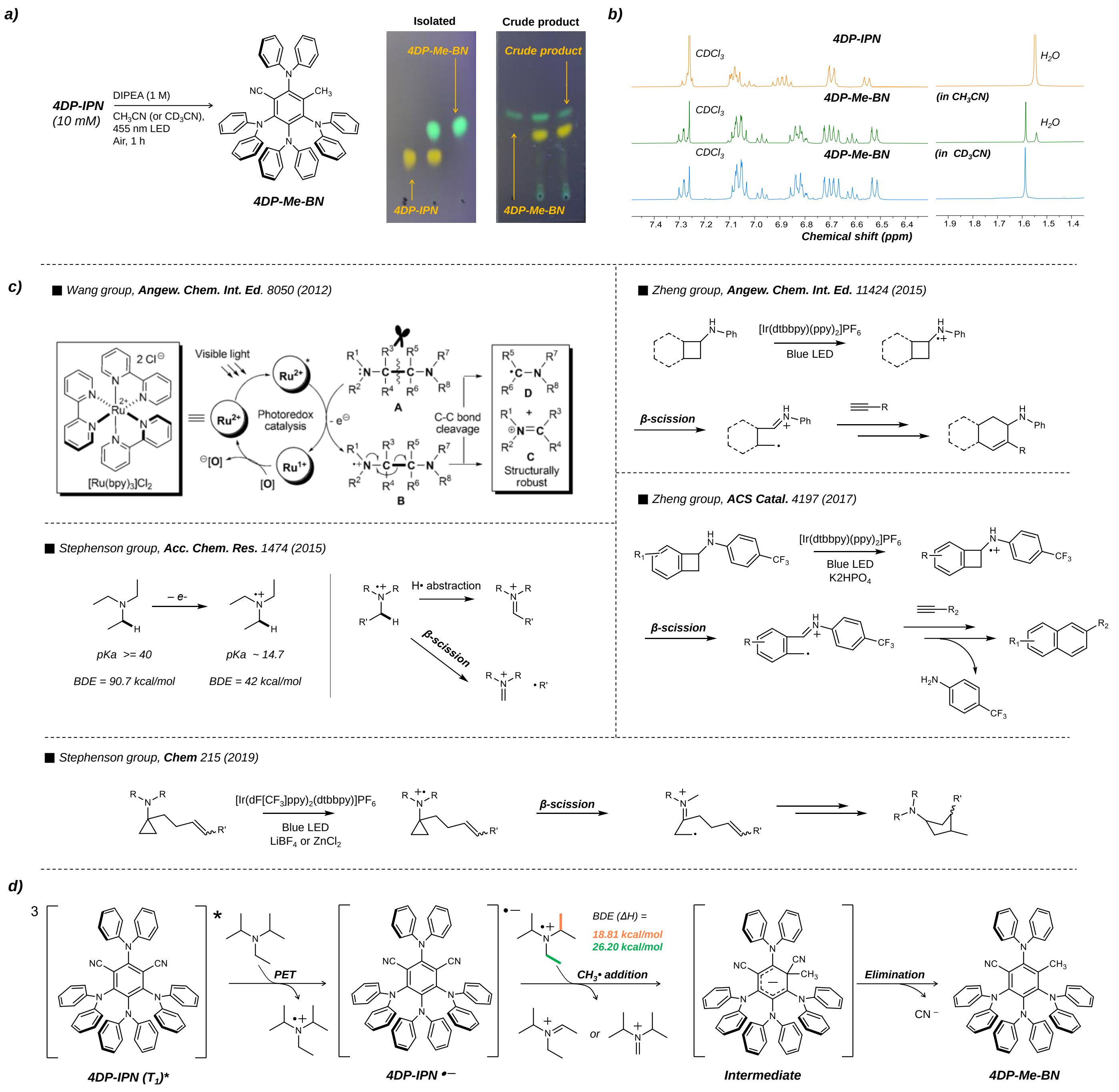

Supplementary Figure 19 Validation of the origin of $CH_3^{\bullet}$ to formation of 4DP-Me-BN. (**a**) Reaction scheme of photodegradation reaction of 4DP-IPN. Reactions were performed with 4DP-IPN (1.0×10^{-2} M) and DIPEA (1 M) in CH_3CN (or CD_3CN) solution under illumination of four 3W 455 nm LEDs for 4 hours at RT without degassing process to promote photosubstitution.[2] PC degradations were monitored in-situ by TLC (EA:hexanes, 1:4 v/v). (**b**) ^{1}H NMR spectra of isolated 4DP-IPN and 4DP-Me-BN synthesized in CH_3CN (or CD_3CN). (**c**) Reaction schemes reported for organic synthesis using β-scission of radical cation of tertiary alkylamine.[52–56] (**d**) Proposed mechanistic pathway for photodegradation behaviour of 4DP-IPN in the presence of DIPEA and DFT calculations for BDE of β-scission in $DIPEA^{\bullet+}$.

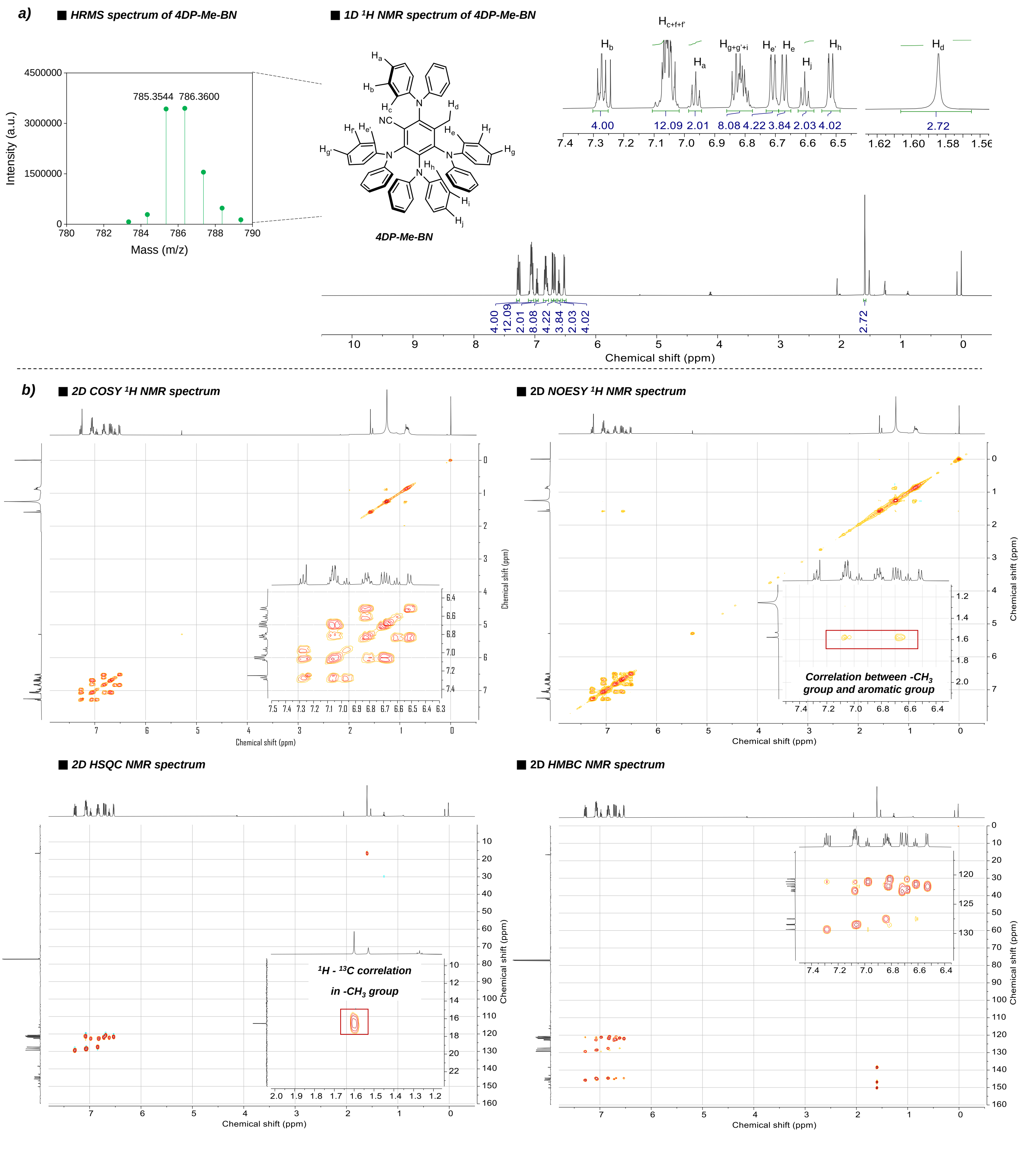

Supplementary Figure 20 Structural characterization of 4DP-Me-BN. Structural analysis of 4DP-Me-BN with (**a**) 1D ^{1}H NMR analyses combined with HRMS, MS (GC-FAB-HRMS): calc'd for $C_{56}H_{44}N_{5}$ $[M+H]^{+}$: 786.3597; found as 786.3600, and (**b**) intense 2D NMR analyses including COSY, NOESY, HSQC and HMBC NMR. ^{1}H NMR (600 MHz, $CDCl_3$): δ 7.30–7.25 (t, 4H), 7.11–7.02 (m, 12H), 6.96 (t, 2H), 6.86–6.78 (m, 8H), 6.73–6.69 (d, 4H), 6.69–6.65 (d, 4H), 6.60 (t, 2H), 6.55–6.49 (d, 4H), 1.58 (s, 3H). ^{13}C NMR (151 MHz, $CDCl_3$): δ 150.15, 148.35, 146.81, 145.76, 145.15, 144.58, 144.56, 142.97, 138.44, 129.31, 128.53, 128.44, 127.50, 122.83, 122.65, 122.46, 122.07, 121.90, 121.55, 121.14, 120.70, 116.15, 114.68, 16.54.

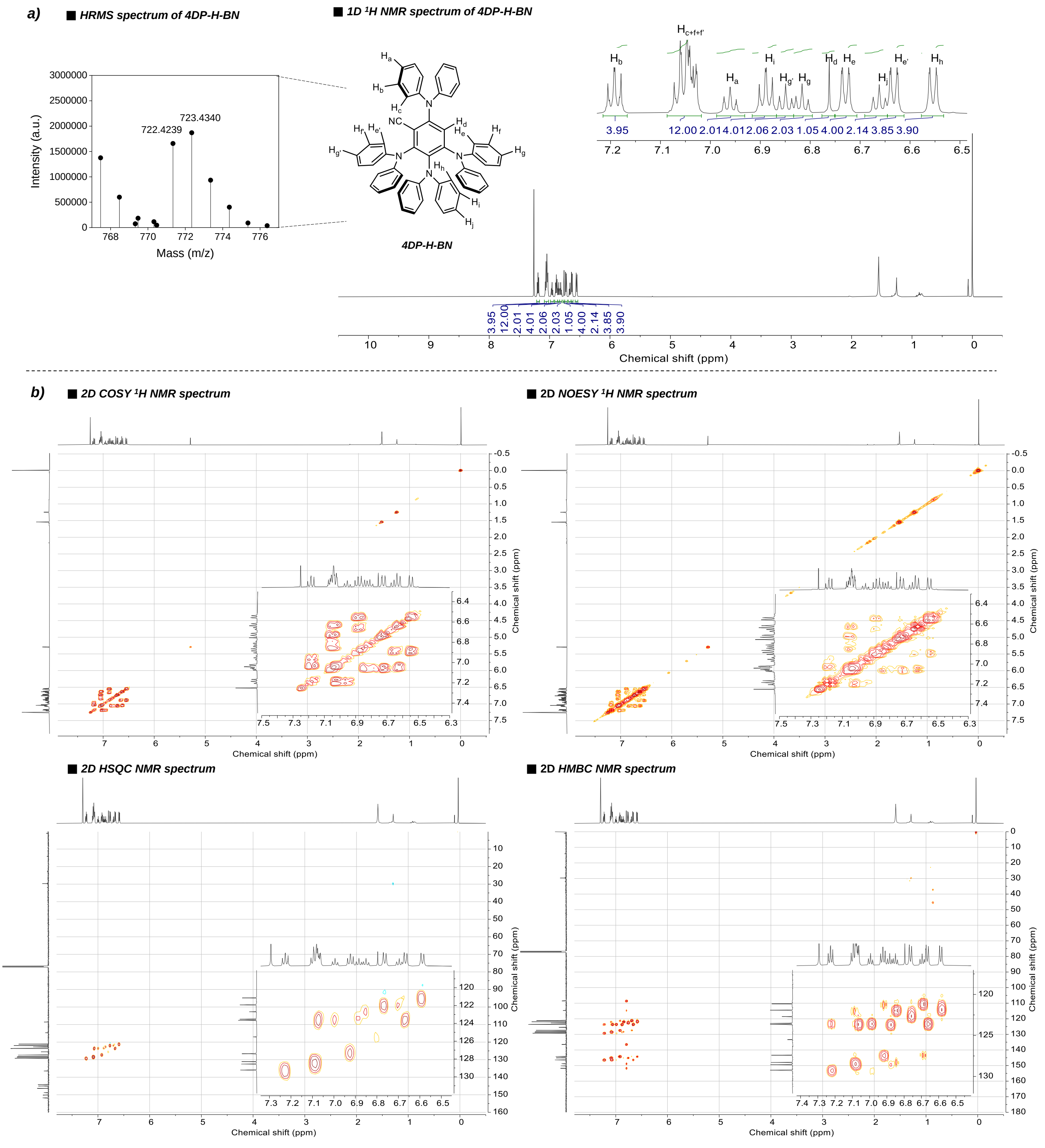

Supplementary Figure 21 Structural characterization of 4DP-H-BN. Structural analysis of 4DP-H-BN with (**a**) 1D ^{1}H NMR analyses combined with HRMS, MS (GC-FAB-HRMS): calc'd for $C_{55}H_{42}N_5$ $[M+H]^+$: 772.3440; found as 772.3444, and (**b**) intense 2D NMR analyses including COSY, NOESY, HSQC and HMBC NMR. ^{1}H NMR (600 MHz, $CDCl_3$): δ 7.22–7.17 (t, 4H), 7.09–7.02 (m, 12H), 6.96 (t, 2H), 6.92–6.87 (t, 4H), 6.87–6.83 (t, 2H), 6.83–6.80 (t, 2H), 6.76 (s, 1H), 6.75–6.71 (d, 4H), 6.69–6.65 (t, 2H), 6.65–6.61 (m, 4H), 6.58–6.53 (t, 4H). ^{13}C NMR (151 MHz, $CDCl_3$): δ 151.76, 150.19, 148.79, 146.38, 146.37, 145.05, 144.21, 136.47, 129.23, 128.56, 128.30, 127.41, 125.51, 123.66, 123.53, 123.51, 122.70, 121.92, 121.15, 114.66, 108.55.

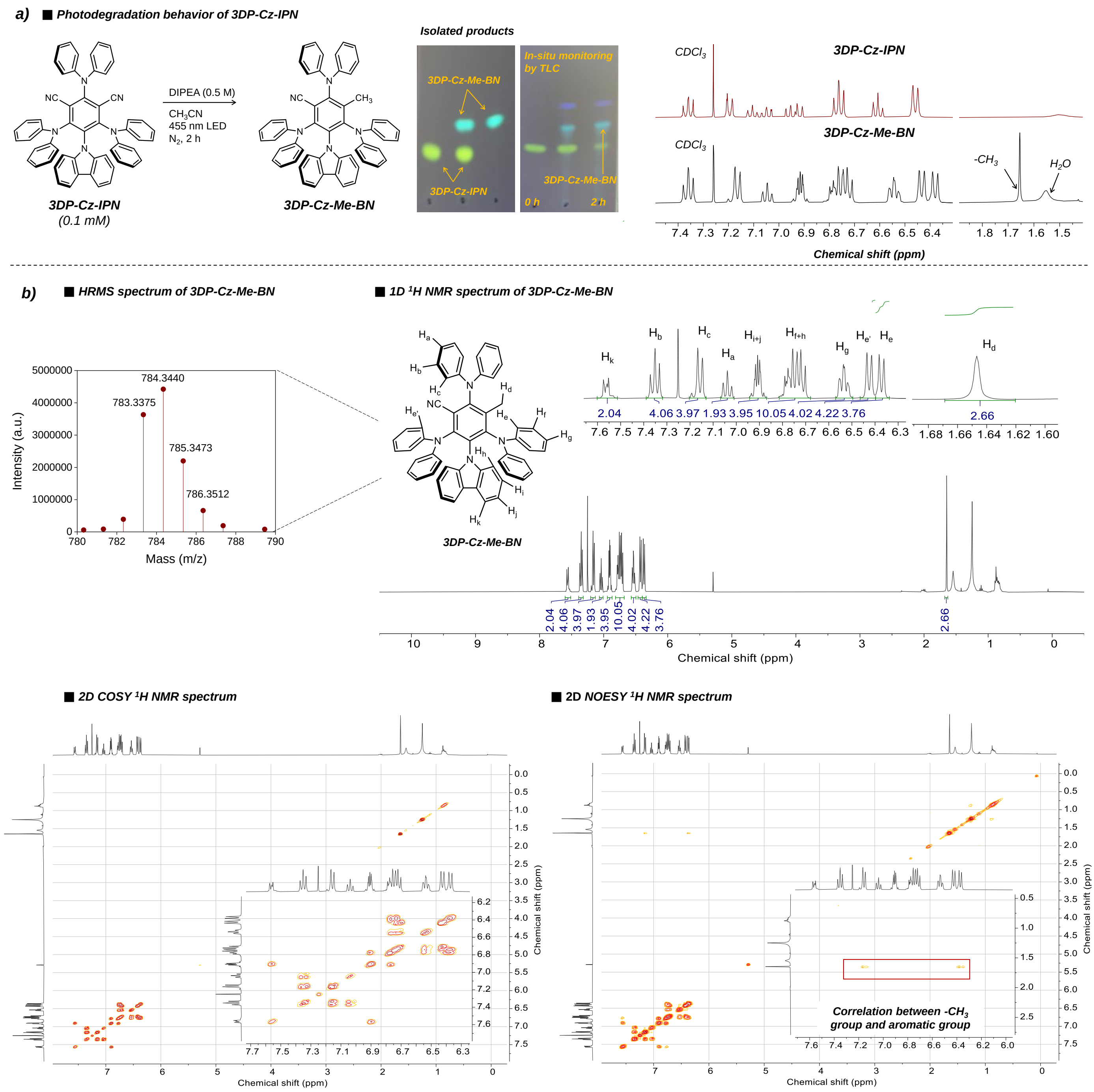

Supplementary Figure 22 (**a**) Photodegradation behavior of 3DP-Cz-IPN. Reactions were performed with PC (1.0×10^{-4} M) and DIPEA (0.5 M) in CH_3CN under the illumination of two 3W 455 nm LEDs for 2 hours at RT. PC degradations were monitored in-situ by TLC (EA:hexanes,1:2 v/v); left/right spots were collected at before/after irradiation and middle spot was co-spot. The photodegraded products were successfully isolated by column chromatography, which gives ^{1}H NMR spectra confirming that a methyl substitution reaction occurred at the CN position. (**b**) Structural analysis of 3DP-Cz-Me-BN with 2D ^{1}H NMR analyses combined with HRMS, MS (GC-FAB-HRMS): calc'd for $C_{56}H_{42}N_5$ $[M+H]^+$: 784.3440; found as 784.3440. ^{1}H NMR (400 MHz, $CDCl_3$): δ 7.60–7.51 (m, 2H), 7.35 (t, 4H), 7.16 (d, 4H), 7.04 (t, 2H), 6.94–6.87 (m, 4H), 6.81–6.68 (m, 10H), 6.53 (td, 4H), 6.43 (d, 4H), 6.37 (d, 4H), 1.65 (s, 3H).

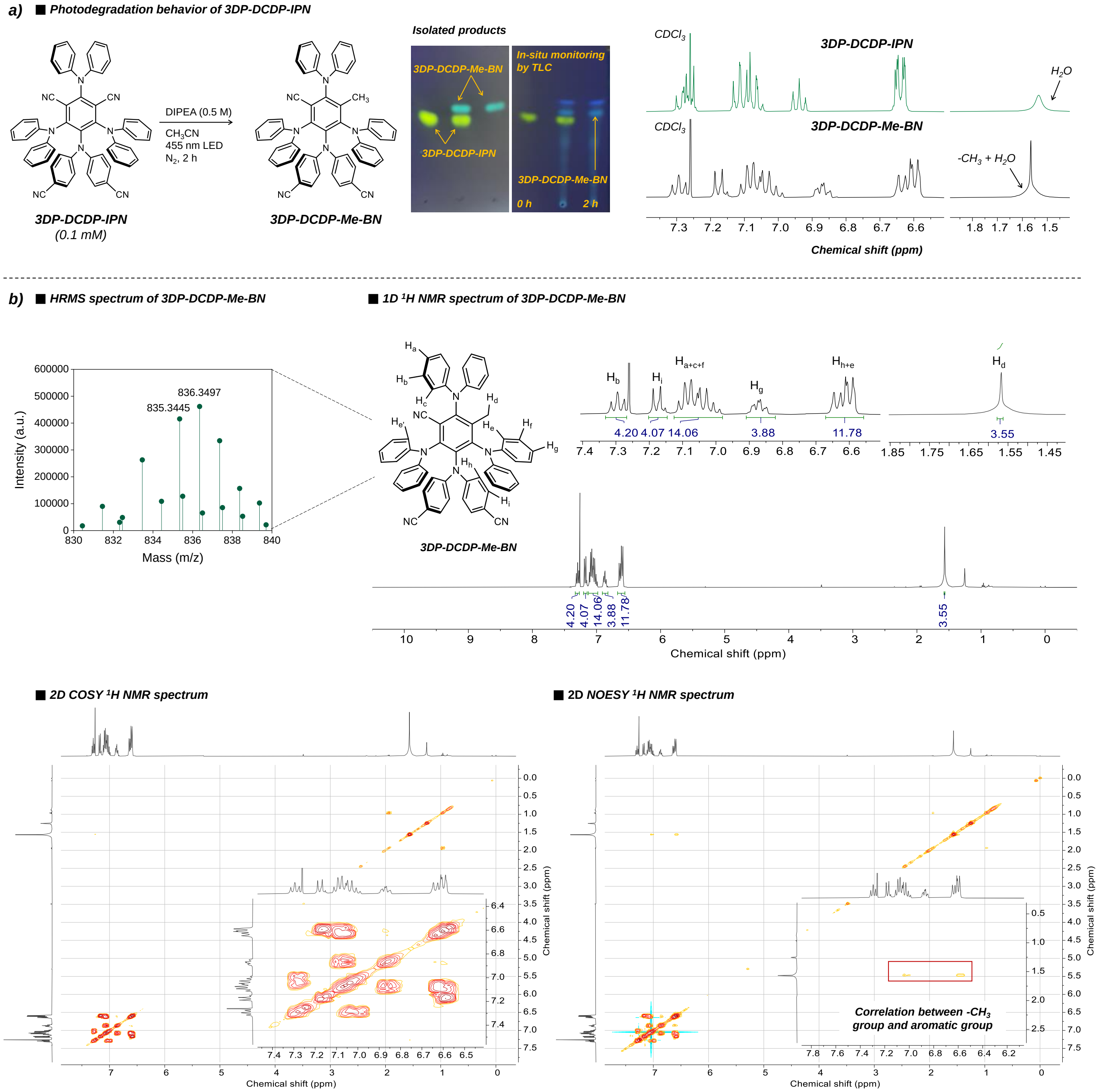

Supplementary Figure 23 (**a**) Photodegradation behavior of 3DP-DCDP-IPN. Reactions were performed with PC (1.0 × 10^{-4} M) and DIPEA (0.5 M) in CH_3CN under the illumination of two 3W 455 nm LEDs for 2 hours at RT. PC degradations were monitored in-situ by TLC (EA:hexanes, 1:1 v/v); left/right spots were collected at before/after irradiation and middle spot was co-spot. The photodegraded products were successfully isolated by column chromatography, which gives ^{1}H NMR spectra confirming that a methyl substitution reaction occurred at the CN position. (**b**) Structural analysis of 3DP-DCDP-Me-BN with 2D ^{1}H NMR analyses combined with HRMS, MS (GC-FAB-HRMS): calc'd for $C_{58}H_{42}N_7$ $[M+H]^+$: 836.3502; found as 836.3497. ^{1}H NMR (400 MHz, $CDCl_3$): δ 7.29 (t, 4H), 7.17 (d, 4H), 7.13–6.98 (m, 14H), 6.87 (td, 4H), 6.67–6.56 (m, 12H), 1.58 (s, 3H).

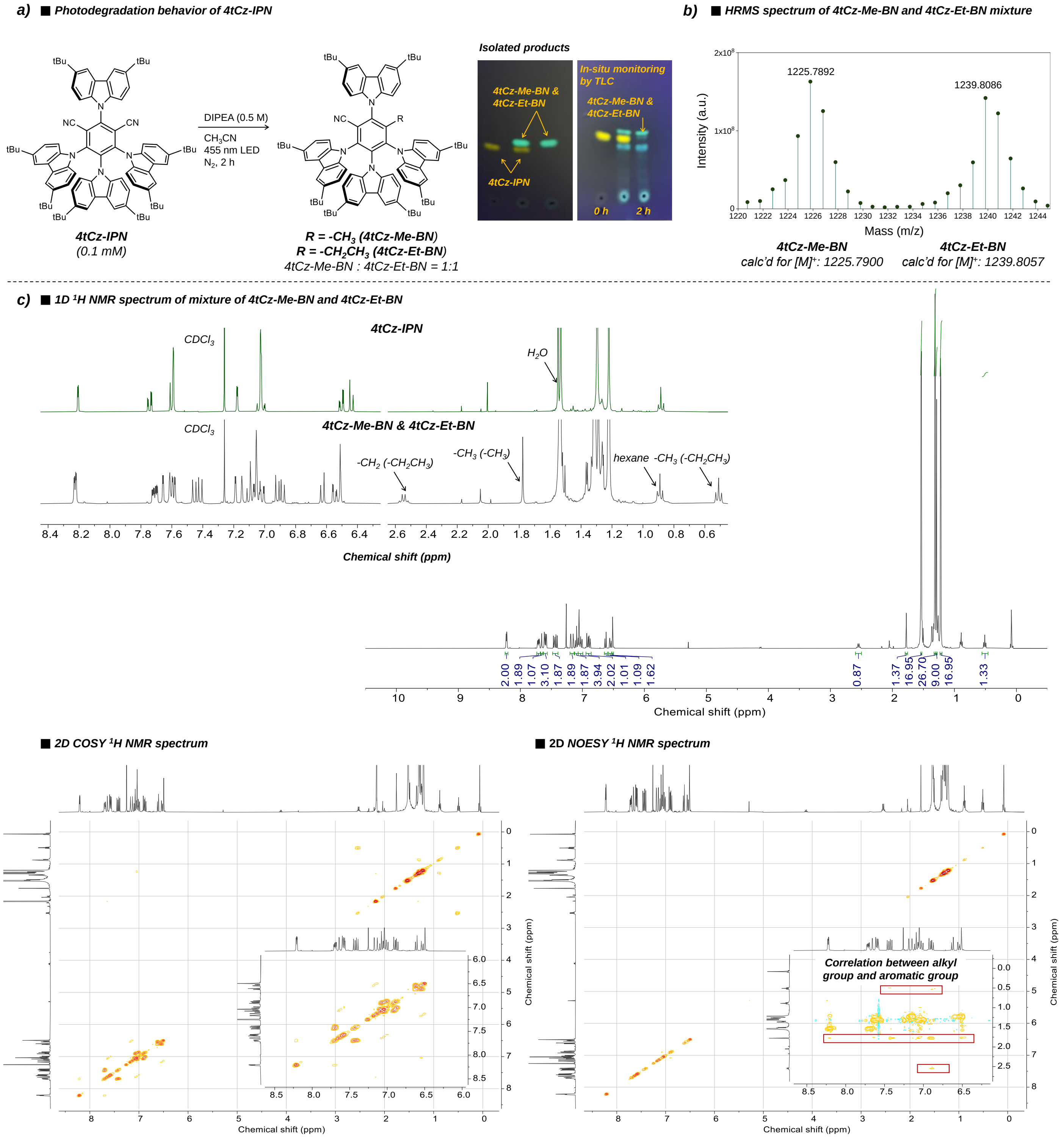

Supplementary Figure 24 Photodegradation behavior of 4tCz-IPN. (**a**) Reactions were performed with PC (1.0×10^{-4} M) and DIPEA (0.5 M) in CH_3CN under the illumination of two 3W 455 nm LEDs for 2 hours at RT. PC degradations were monitored in-situ by TLC (EA:hexanes, 5:95 v/v); left/right spots were collected at before/after irradiation and middle spot was co-spot. (**b, c**) The mixture of photodegraded products was successfully isolated by column chromatography, which gives ^1H NMR spectra confirming that a methyl (or ethyl) substitution reaction occurred at the CN position combined with HRMS, MS (GC-FAB-HRMS): calc'd for 4tCz-Me-BN ($C_{88}H_{99}N_5$) $[M]^+$: 1225.7900; found as 1225.7892, calc'd for 4tCz-Et-BN ($C_{89}H_{101}N_5$) $[M]^+$: 1239.8057; found as 1239.8086.

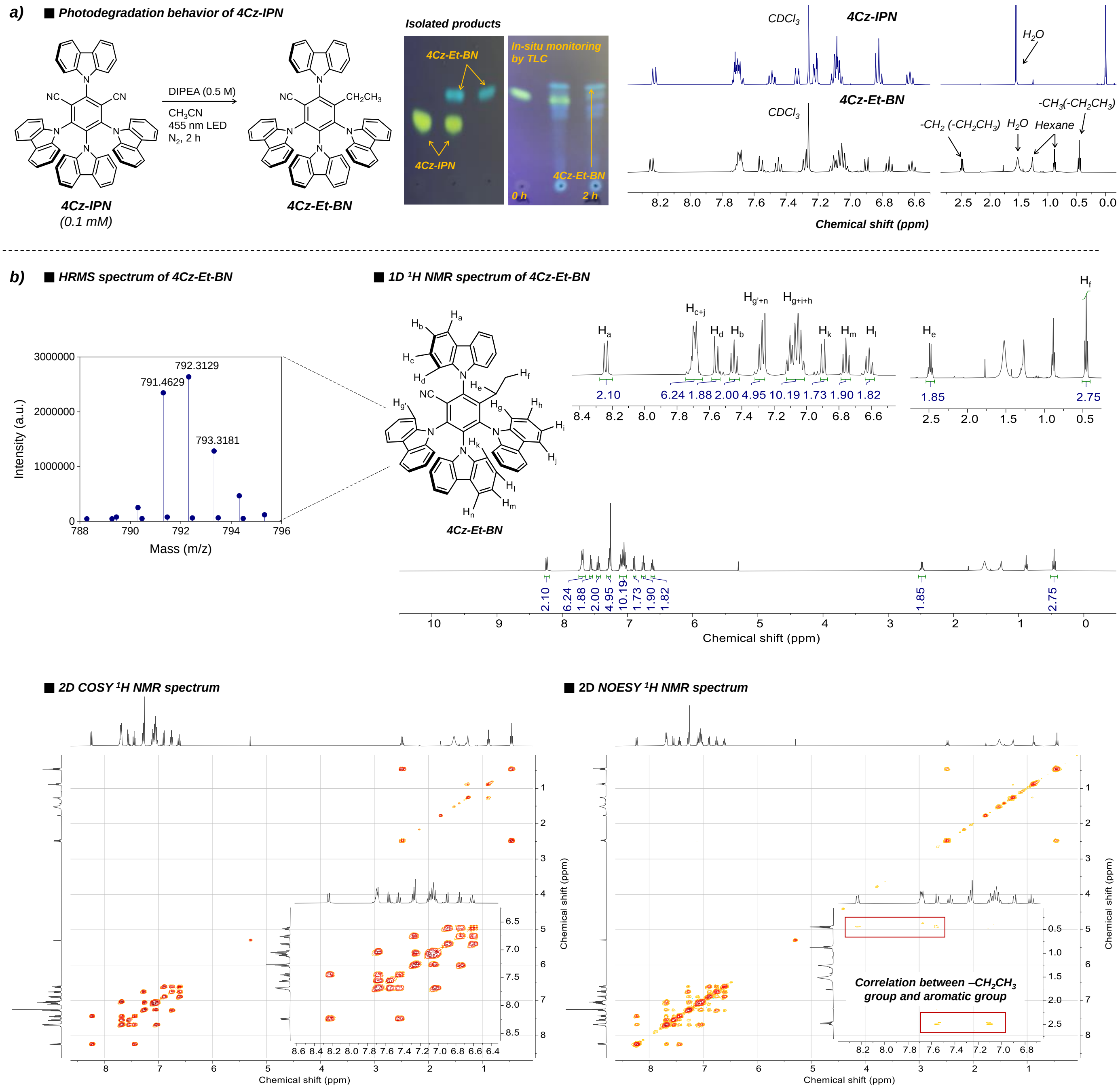

Supplementary Figure 25 Photodegradation behavior of 4Cz-IPN. (**a**) Reactions were performed with PC (1.0×10^{-4} M) and DIPEA (0.5 M) in CH_3CN under the illumination of two 3W 455 nm LEDs for 2 hours at RT. PC degradations were monitored in-situ by TLC (EA:hexanes, 1:4 v/v); left/right spots were collected at before/after irradiation and middle spot was co-spot. The photodegraded products were successfully isolated by column chromatography, which gives ^1H NMR spectra confirming that an ethyl substitution reaction occurred at the CN position. (**b**) Structural analysis of 4Cz-Et-BN with 2D ^1H NMR analyses combined with HRMS. ^1H NMR (400 MHz, $CDCl_3$): δ 8.23 (d, 2H), 7.69 (m, 6H), 7.55 (d, 2H), 7.44 (t, 2H), 7.27 (t, 4H), 7.15–7.00 (m, 10H), 6.89 (d, 2H), 6.76 (d, 2H), 6.60 (t, 2H), 2.47 (q, 2H), 0.45 (t, 3H). MS (GC-FAB-HRMS): calc'd for $C_{57}H_{38}N_5$ $[M+H]^+$: 792.3127; found as 792.3129.

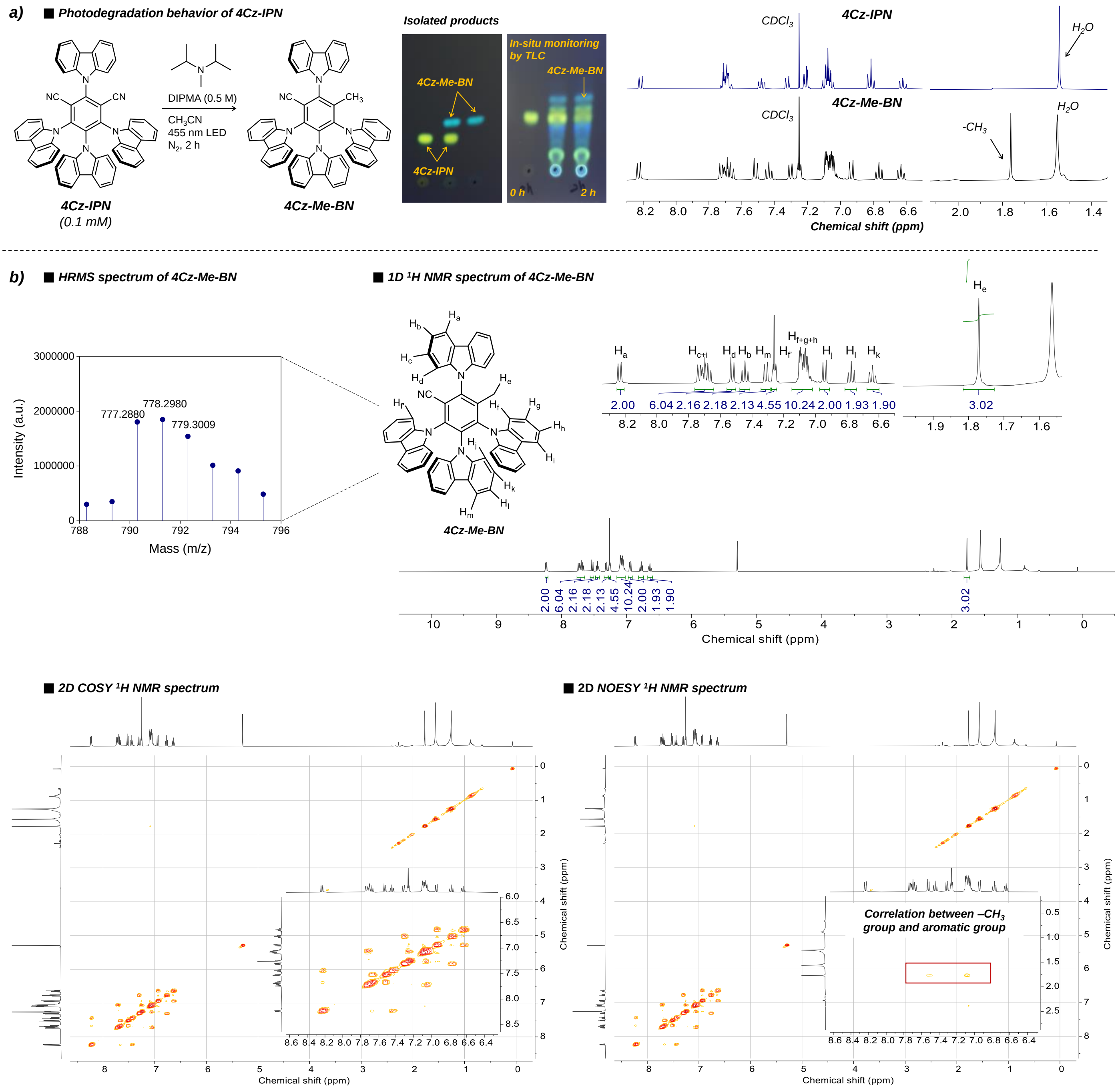

Supplementary Figure 26 Photodegradation behavior of 4Cz-IPN. (**a**) Reactions were performed with PC (1.0×10^{-4} M) and DIPMA (0.5 M) in CH_3CN under the illumination of two 3W 455 nm LEDs for 2 hours at RT. PC degradations were monitored in-situ by TLC (EA:hexanes, 1:4 v/v); left/right spots were collected at before/after irradiation and middle spot was co-spot. The photodegraded products were successfully isolated by column chromatography, which gives ^{1}H NMR spectra confirming that a methyl substitution reaction occurred at the CN position. (**b**) Structural analysis of 4Cz-Me-BN with 2D ^{1}H NMR analyses combined with HRMS. ^{1}H NMR (400 MHz, $CDCl_3$): δ 8.24 (d, 2H), 7.76–7.64 (m, 6H), 7.52 (d, 2H), 7.44 (t, 2H), 7.31 (d, 2H), 7.28–7.24 (m, 2H), 7.15–7.02 (m, 10H), 6.94 (d, 2H), 6.77 (t, 2H), 6.64 (t, 2H), 1.77 (s, 3H). MS (GC-FAB-HRMS): calc'd for $C_{56}H_{36}N_5$ $[M+H]^+$: 778.2971; found as 778.2980.

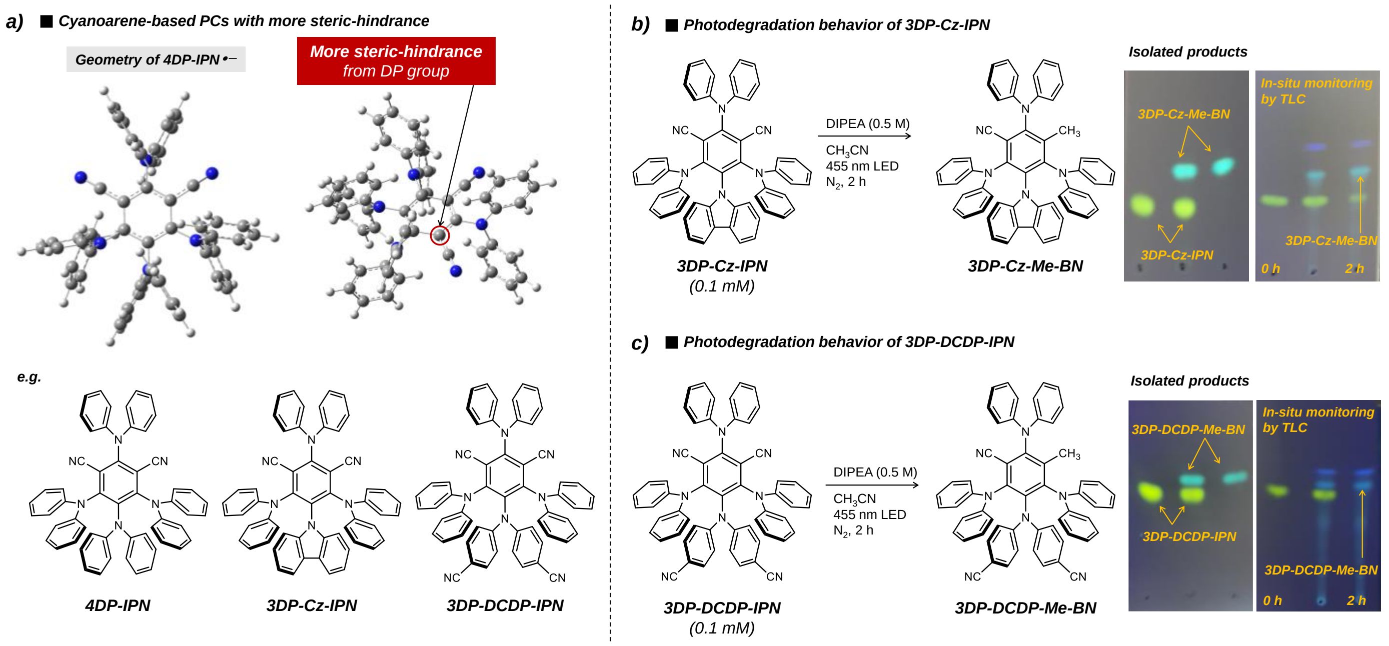

Supplementary Figure 27 (**a**) Photodegradation behaviors of cyanoarene-based PCs with more steric-hindrance. The geometry of radical anions of 4DP-IPN was obtained by DFT calculation, B3LYP/6-311++G*, in CH_3CN with PCM solvation model. In the precene of DIPEA, Photodegradation behavior of (**b**) 3DP-Cz-IPN and (**c**) 3DP-DCDP-IPN. PC degradations were monitored in-situ by TLC (EA:hexanes, 1:2 v/v for 3DP-Cz-IPN and EA:hexanes, 1:1 v/v for 3DP-DCDP-IPN); left/right spots were collected at before/after irradiation and middle spot was co-spot.

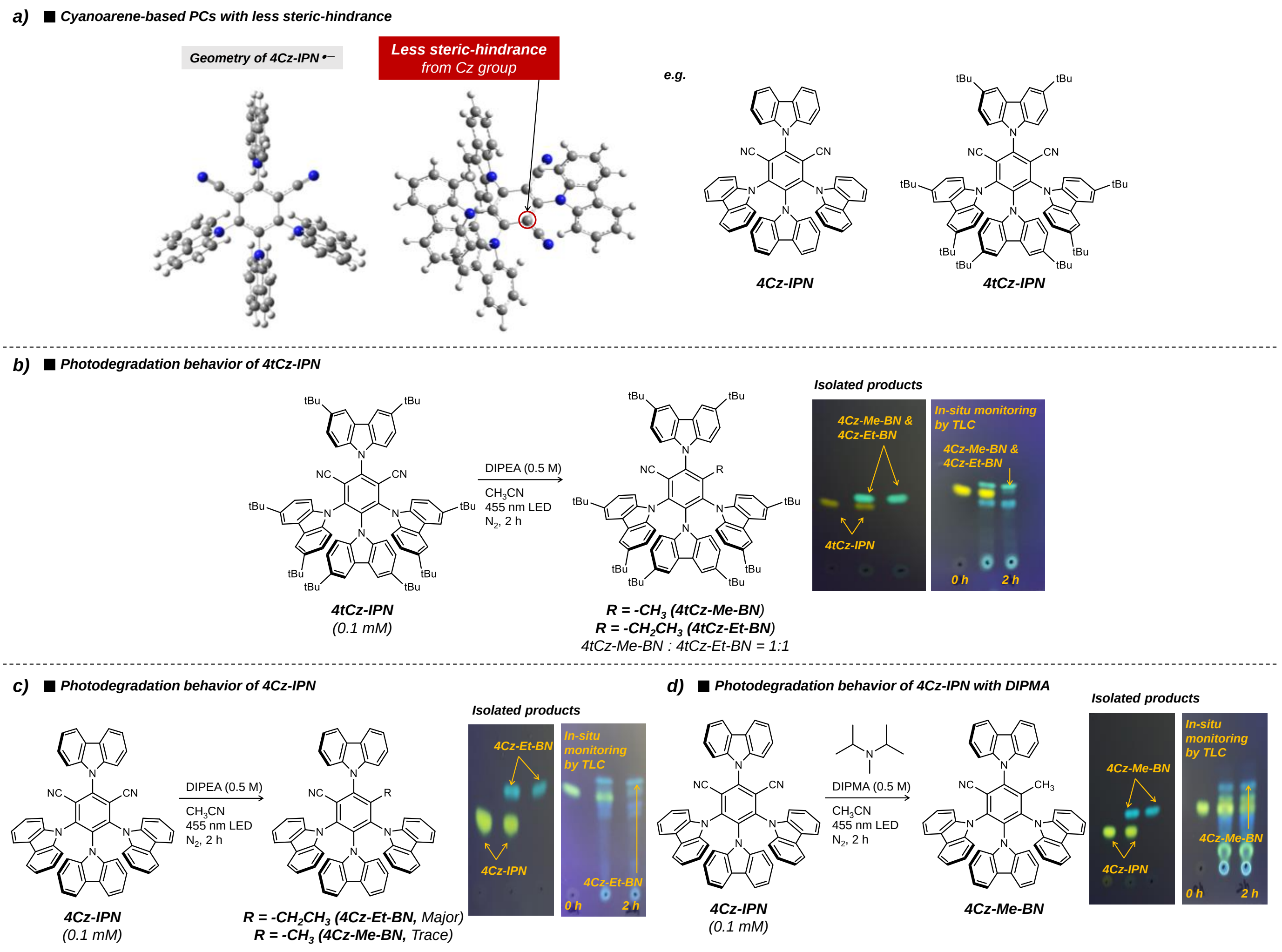

Supplementary Figure 28 (**a**) Photodegradation behaviors of cyanoarene-based PCs with less steric-hindrance. The geometry of radical anions of 4Cz-IPN was obtained by DFT calculation, B3LYP/6-311++G*, in CH_3CN with PCM solvation model. In the presence of DIPEA, Photodegradation behavior of (**b**) 4tCz-IPN and (**c**) 4Cz-IPN. (**d**) Photodegradation behavior of 4Cz-IPN using DIPMA instead of DIPEA. PC degradations were monitored in-situ by TLC (EA:hexanes, 5:95 v/v for 4tCz-IPN and EA:hexanes, 1:1 v/v for 4Cz-IPN); left/right spots were collected at before/after irradiation and middle spot was co-spot.

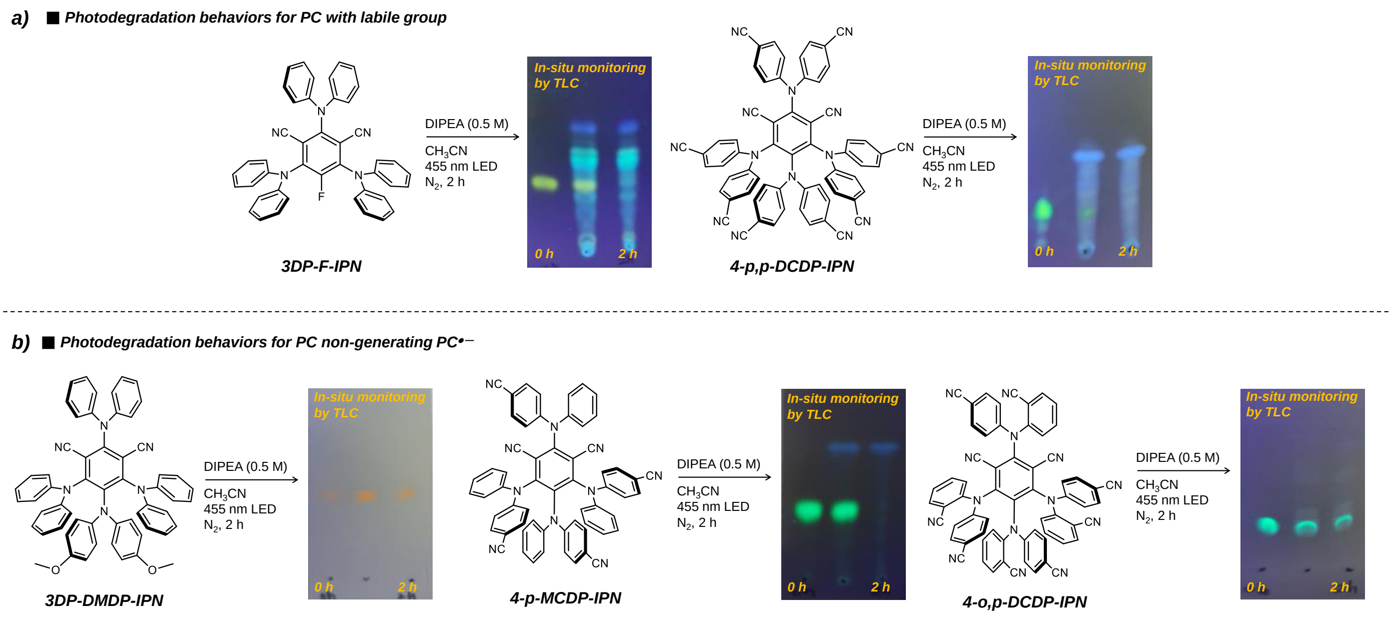

Supplementary Figure 29 (**a**) Photodegradation behavior of PCs with labile group, 3DP-F-IPN and 4-p,p-DCDP-IPN. Reactions were performed with PC (1.0×10^{-4} M) and DIPEA (0.5 M) in CH_3CN under the illumination of two 3W 455 nm LEDs for 2 hours at RT. PC degradations were monitored in-situ by TLC (EA:hexanes, 1:6 v/v for 3DP-F-IPN and acetone:hexanes, 2:3 v/v for 4-p,p-DCDP-IPN); left/right spots were collected at before/after irradiation and middle spot was co-spot. (**b**) Photodegradation behavior of PCs non-generating $PC^{\bullet-}$, 3DP-DMDP-IPN, 4-p-MCDP-IPN and 4-o,p-DCDP-IPN. Reactions were performed with PC (1.0×10^{-4} M) and DIPEA (0.5 M) in CH_3CN un der the illumination of two 3W 455 nm LEDs for 2 hours at RT. PC degradations were monitored in-situ by TLC (EA:hexanes, 1:1 v/v for 3DP-DMDP-IPN, EA:hexanes, 7:3 v/v for 4-p-MCDP-IPN and MeOH:$CHCl_3$ = 1:4 v/v for 4-o,p-DCDP-IPN); left/right spots were collected at before/after irradiation and middle spot was co-spot.

Supplementary Note 11. Oxygen tolerance in photoredox reductive dehalogenation

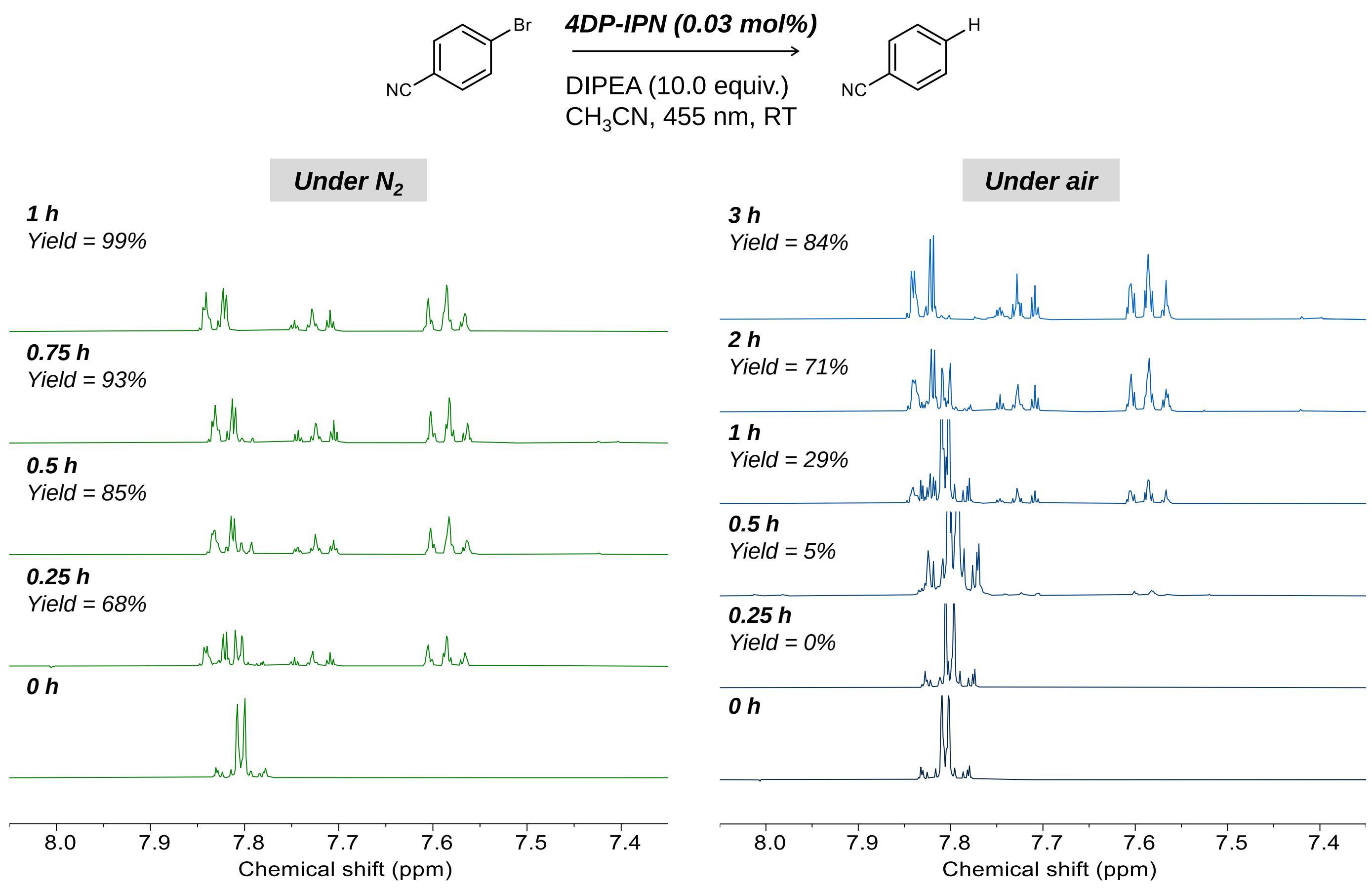

Supplementary Figure 30 Estimation of O_2 effect in photoredox reductive dehalogenation. Reaction condition was the same described above for the general procedure, 4-bromobenzonitrile (0.2 mmol, 1 equiv.), DIPEA (10.0 equiv.) and 4DP-IPN (0.03 mol%) in CH_3CN (2 mL, 0.1 M of aryl halides) under illumination of two 3W 455 nm LEDs at RT. All the samples were prepared in situ and all of yields were determined by ^1H NMR using 1,3,5-trimethoxybenzene as an internal standard (400 MHz, DMSO-d_6).

Supplementary Table 8 Oxygen tolerance test for the dehalogenation of 4-bromobenzonitrile varying concentration of DIPEA.

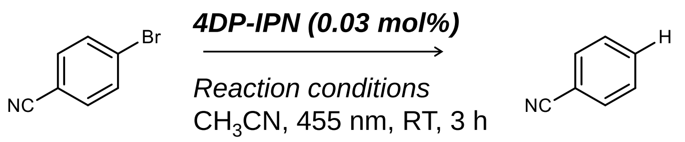

Entry	*Reaction conditions*	*Yield (%)*
1	DIPEA (2.0 equiv.) under N_2	62
2	DIPEA (2.0 equiv.) under air	0
3	DIPEA (5.0 equiv.) under air	12
4	DIPEA (10.0 equiv.) under air	84

Reactions were performed with 4-bromobenzonitrile (0.1 M), 4DP-IPN (0.03 mol%), DIPEA (2.0-10.0 equiv.) in CH_3CN (1 mL) under two 3W 455 nm LEDs for 3 hours under air atmosphere in the closed glass vial. All solutions were prepared outside under ambient conditions. Yields were determined by ^{1}H NMR using 1,3,5-trimethoxybenzene as an internal standard.

Supplementary Note 12. Subsequential injection of 4DP-IPN in photoredox reductive dehalogenation

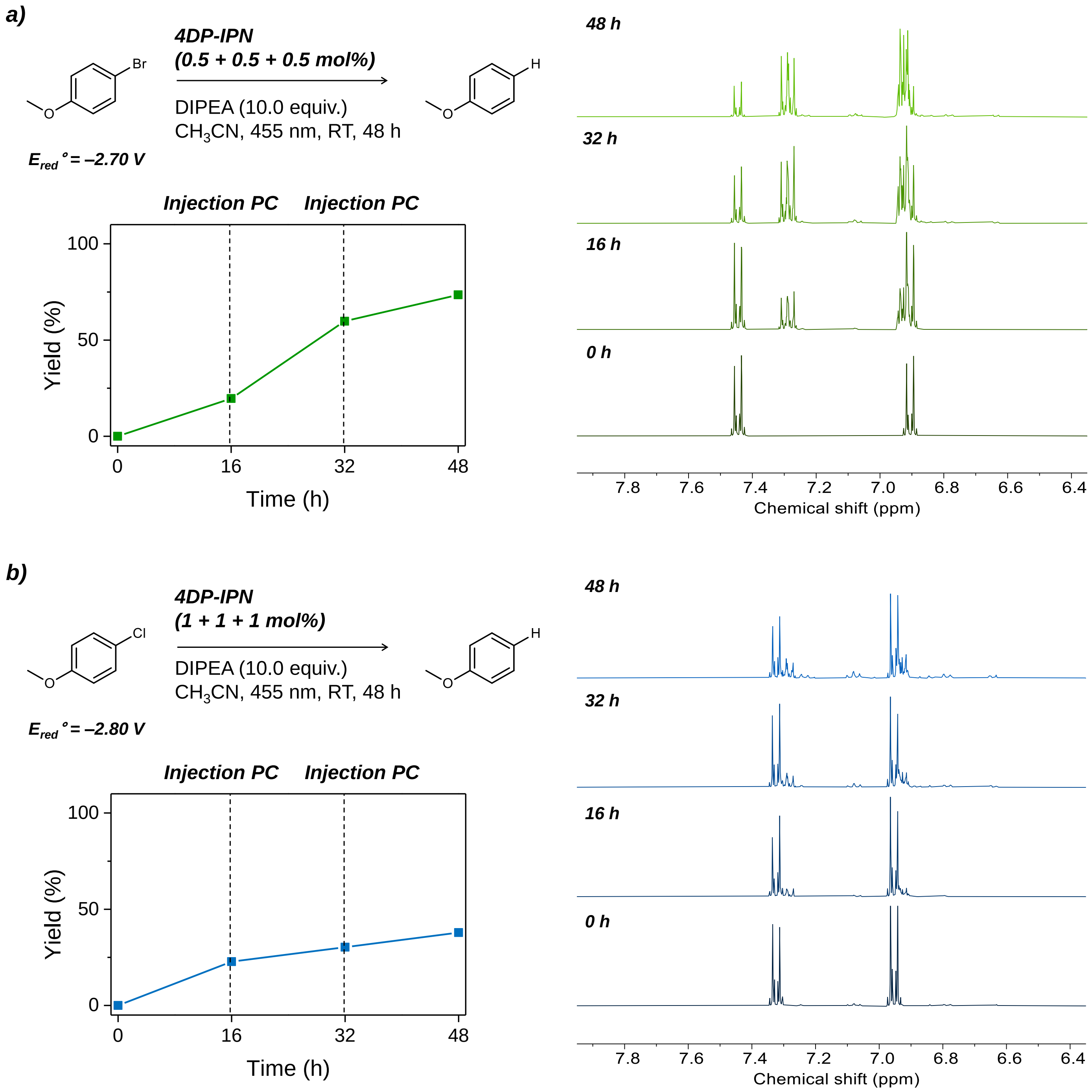

Supplementary Figure 31 Subsequential addition of PC into photoredox reductive dehalogenation. Reaction condition was similar with general procedure, aryl halides (0.2 mmol, 1 equiv.), DIPEA (10.0 equiv.) and (**a**) 0.5 mol% of 4DP-IPN, (**b**) 1 mol% of 4DP-IPN in CH_3CN (2 mL, 0.1 M of aryl halides) under illumination of four 3W 455 nm LEDs at RT. Additional solution (0.1 ml) of (**a**) 0.5 mol% of 4DP-IPN and (**b**) 1 mol% of 4DP-IPN were injected every 16 hours. To monitor yield and conversion, all the samples were prepared in situ and all of yields were determined by ^{1}H NMR using 1,3,5-trimethoxybenzene as an internal standard (400 MHz, DMSO-d_6).

Supplementary Note 13. NMR analysis of PCs

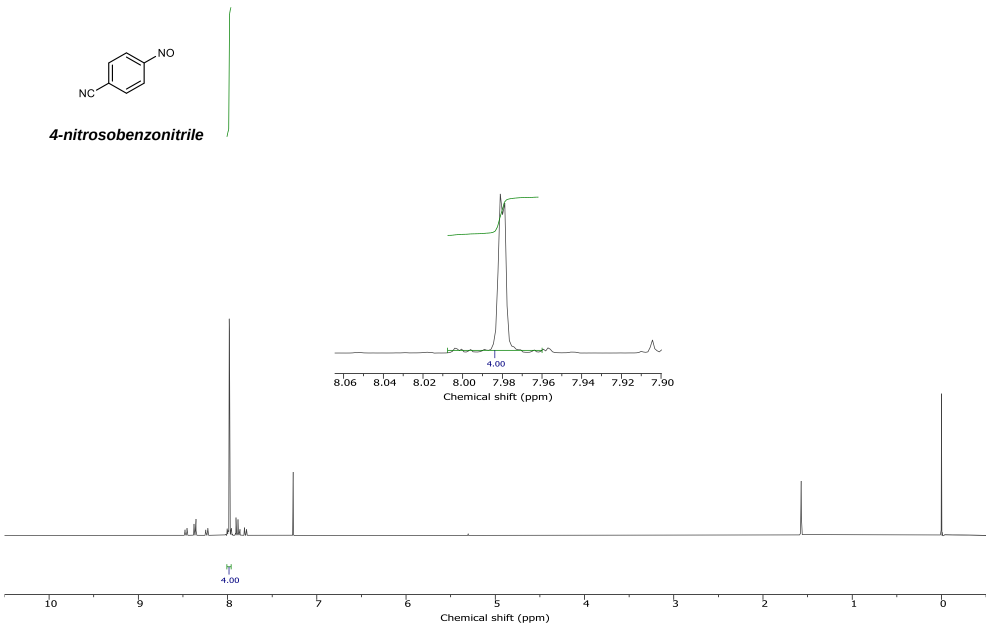

Supplementary Figure 32 ^{1}H NMR data of 4-nitrosobenzonitrile at RT (400 MHz, $CDCl_3$). ^{1}H NMR (400 MHz, $CDCl_3$): δ 7.97 (br s, 4H).

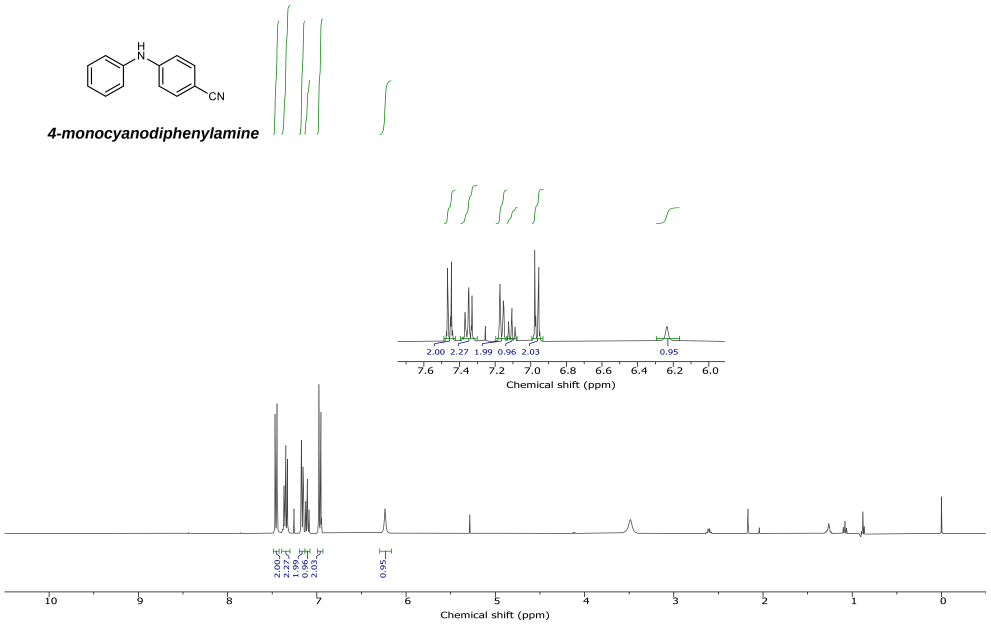

Supplementary Figure 33 ^{1}H NMR data of 2,4′-dicyanodiphenylamine at RT (400 MHz, $CDCl_3$). ^{1}H NMR (400 MHz, $CDCl_3$): δ 7.4 –7.42 (m, 2H), 7.39–7.30 (m, 2H), 7.20–7.13 (m, 2H), 7.13–7.08 (m, 1H), 6.99–6.93 (m, 2H), 6.24 (s, 1H).

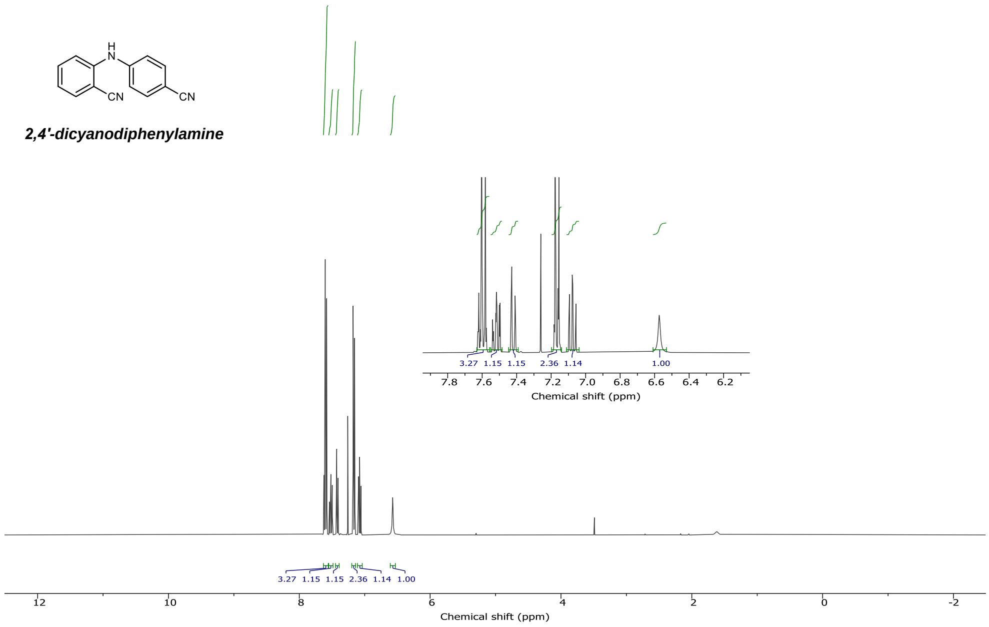

Supplementary Figure 34 ^{1}H NMR data of 2,4′-dicyanodiphenylamine at RT (400 MHz, $CDCl_3$). ^{1}H NMR (400 MHz, $CDCl_3$): δ 7.60 (dd, 3H), 7.55–7.48 (m, 1H), 7.42 (dd, 1H), 7.20–7.14 (m, 2H), 7.08 (td, 1H), 6.57 (s, 1H).

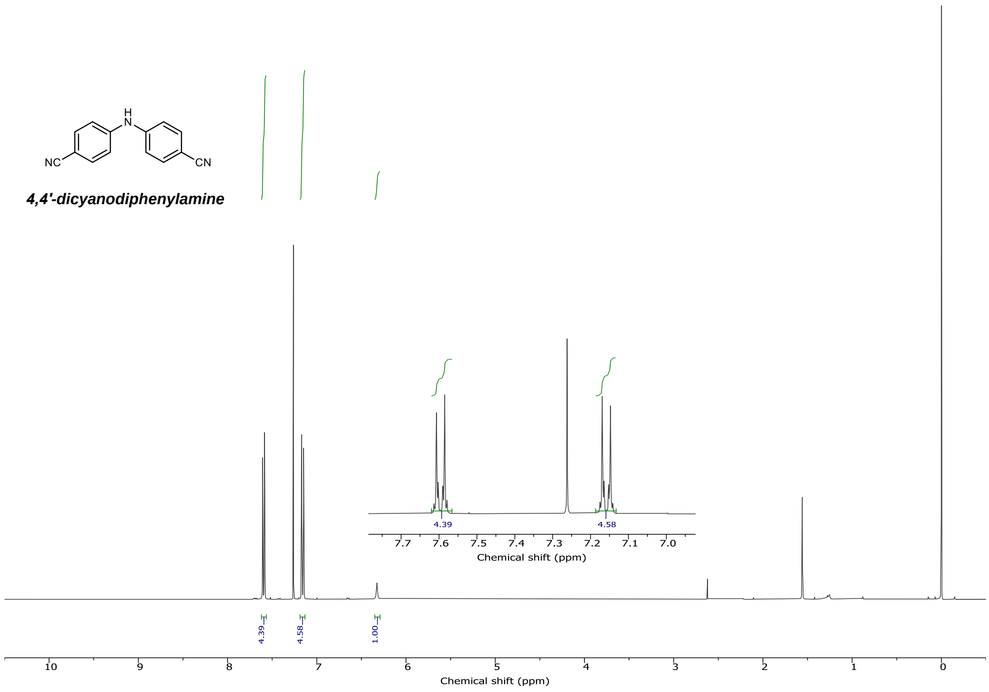

Supplementary Figure 35 ^{1}H NMR data of 4,4′-dicyanodiphenylamine at RT (400 MHz, $CDCl_3$). ^{1}H NMR (400 MHz, $CDCl_3$): δ 7.62–7.57 (dt, 4H), 7.18–7.15 (dt, 4H), 6.38–7.15 (s, 1H).

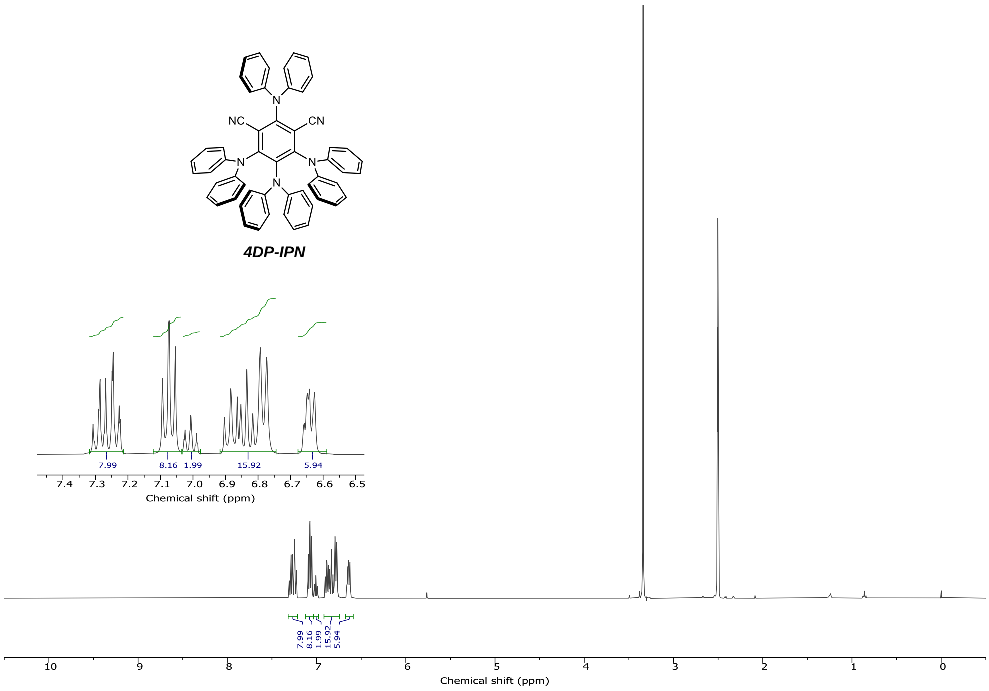

Supplementary Figure 36 ^{1}H NMR data of 4DP-IPN at RT (400 MHz, DMSO-d_6). ^{1}H NMR (400 MHz, DMSO-D_6): δ 7.31 7.21 (m, 8H), 7.10–7.05 (t, 8H), 7.03–6.97 (t. 2H), 6.91–6.75 (m, 16H), 6.68–6.62 (m, 6H).

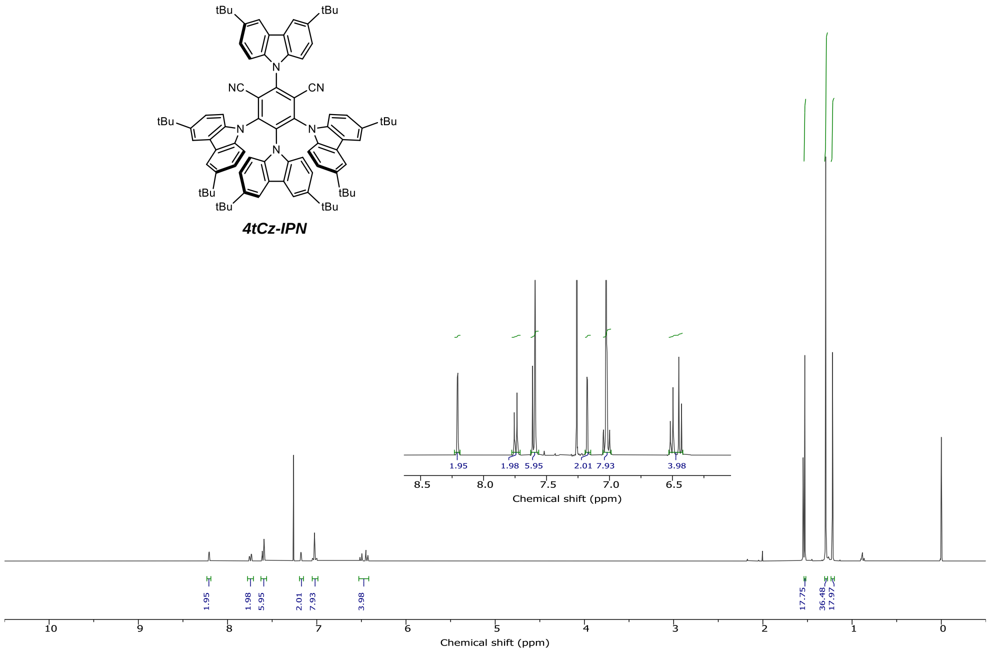

Supplementary Figure 37 ^{1}H NMR data of 4tCz-IPN at RT (400 MHz, $CDCl_3$). ^{1}H NMR (400 MHz, $CDCl_3$): δ 8.21 (d, 2H), 7.74 (dd, 2H), 7.61–7.59 (m, 6H), 7.18 (d, 2H), 7.05–7.00 (m, 8H), 6.51 (dd, 2H), 6.44 (d, 2H), 1.53 (s, 18H), 1.30 (s, 36H), 1.22 (s, 18H).

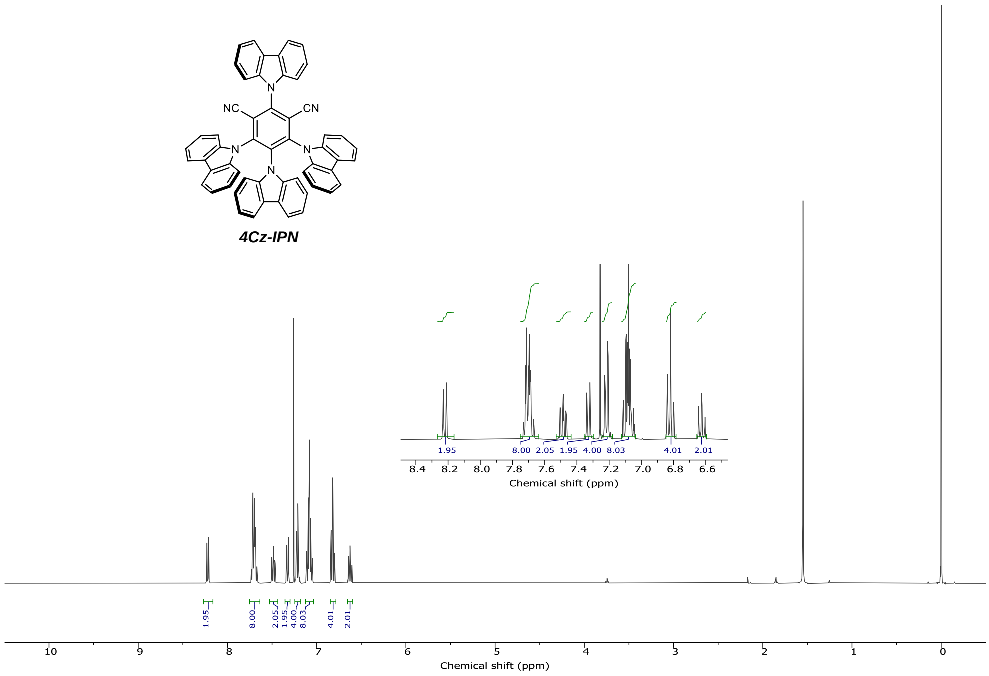

Supplementary Figure 38 ^{1}H NMR data of 4Cz-IPN at RT (400 MHz, $CDCl_3$). ^{1}H NMR (400 MHz, CDCl3): δ 8.22 (dt, 2H), 7.74–7.67 (m, 8H), 7.49 (ddd, 2H), 7.33 (dt, 2H), 7.23–7.21 (m, 4H), 7.12–7.05 (m, 8H), 6.82 (td, 4H), 6.63 (ddd, 2H).

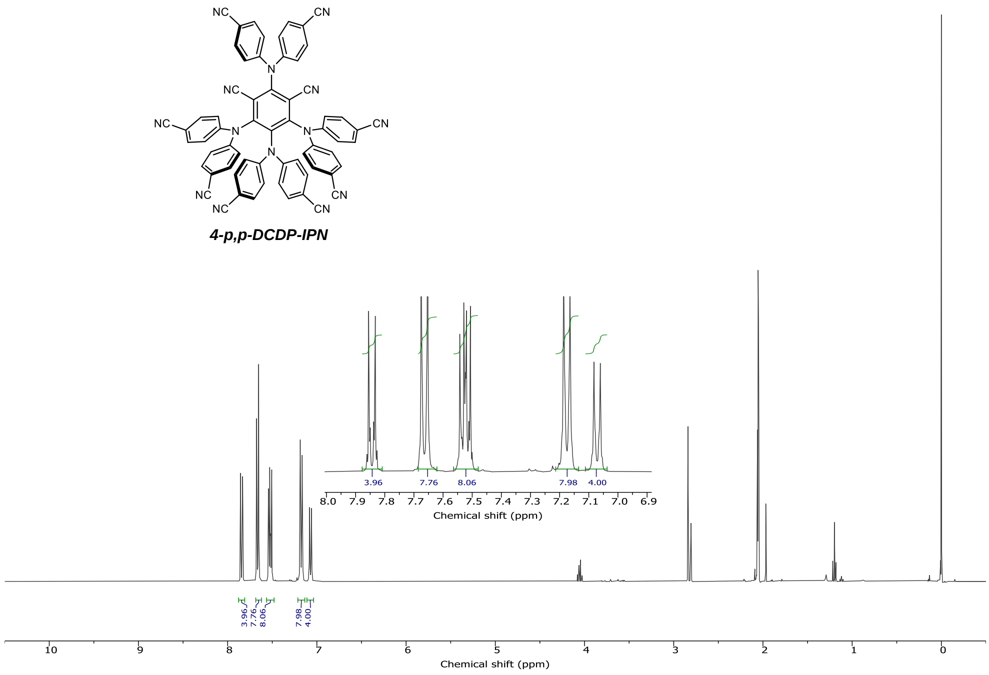

Supplementary Figure 39 ^{1}H NMR data of 4-p,p-DCDP-IPN at RT (400 MHz, acetone-d_6). ^{1}H NMR (400 MHz, acetone-d_6): δ 7.87–7.81 (d, 4H), 7.70–7.62 (d, 8H), 7.55–7.48 (td. 8H), 7.20–7.15 (d, 8H), 7.10–7.03 (d, 4H).

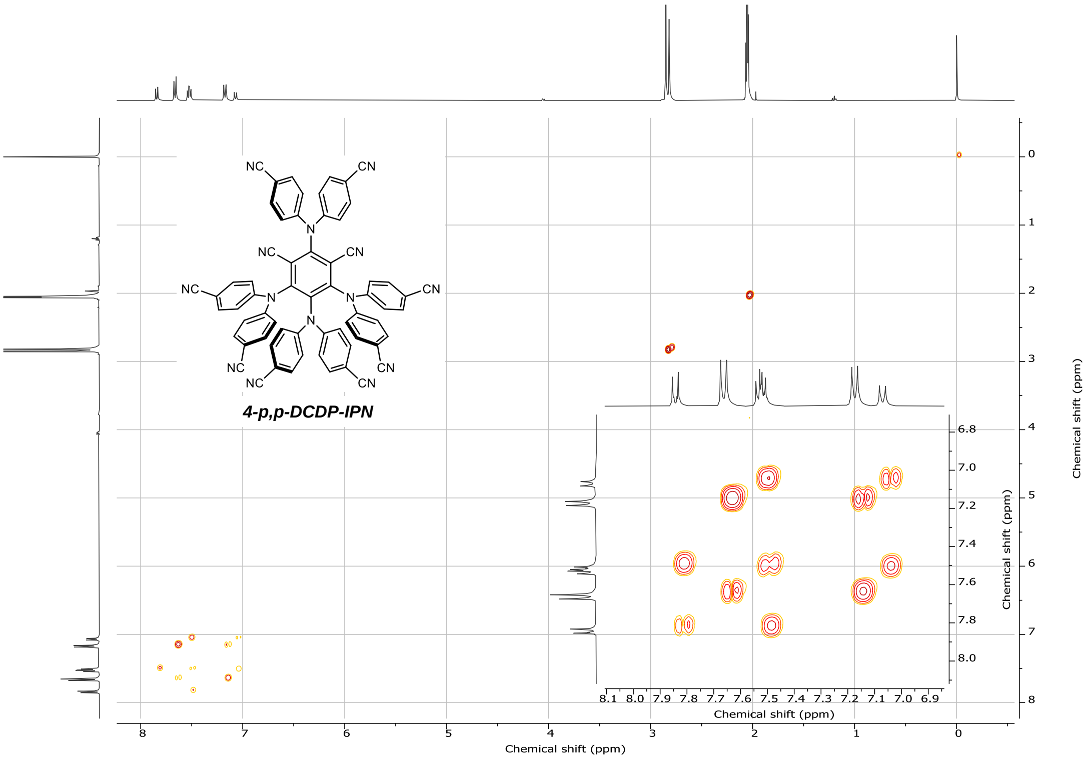

Supplementary Figure 40 COSY NMR data of 4-p,p-DCDP-IPN at RT (400 MHz, acetone-d_6).

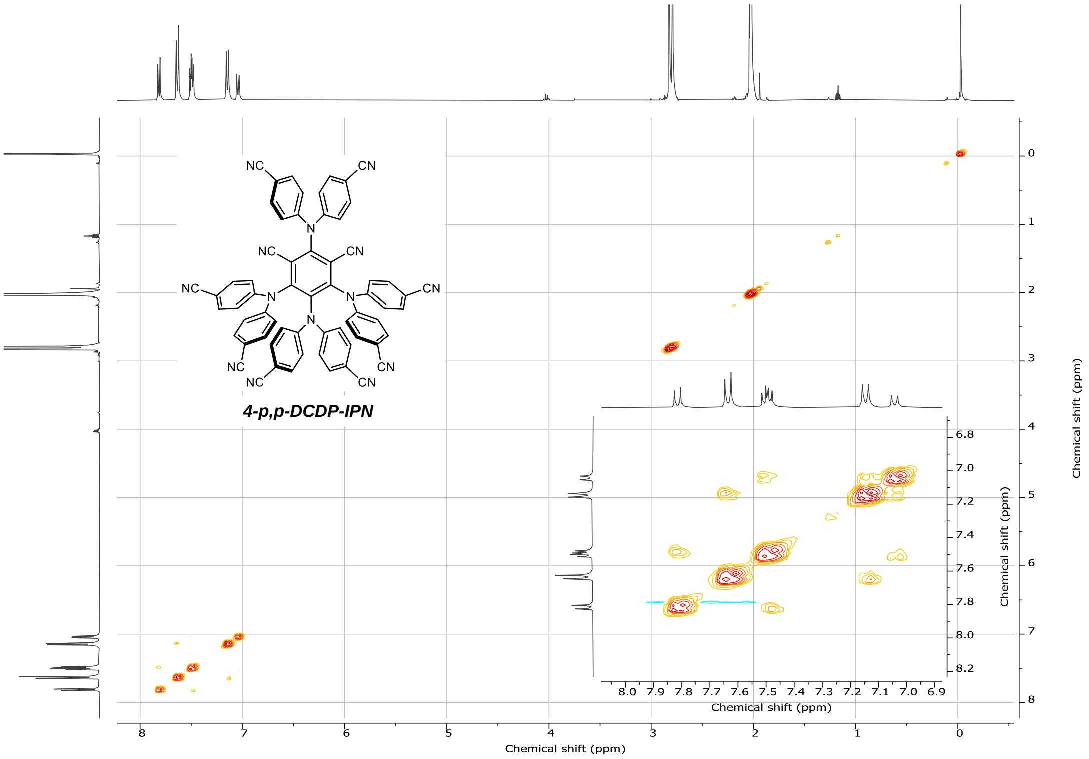

Supplementary Figure 41 NOESY NMR data of 4-p,p-DCDP-IPN at RT (400 MHz, acetone-d_6).

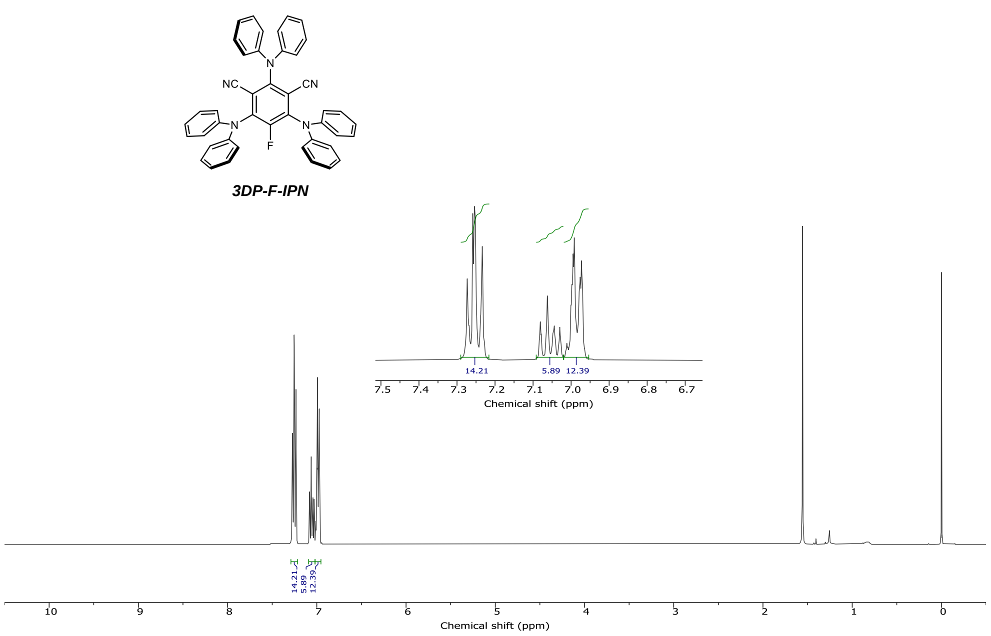

Supplementary Figure 42 ^{1}H NMR data of 3DP-F-IPN at RT (400 MHz, $CDCl_3$). ^{1}H NMR (400 MHz, $CDCl_3$): δ 7.29–7.22 (m, 12H), 7.09–7.02 (m, 6H), 7.02–6.95 (m, 12H).

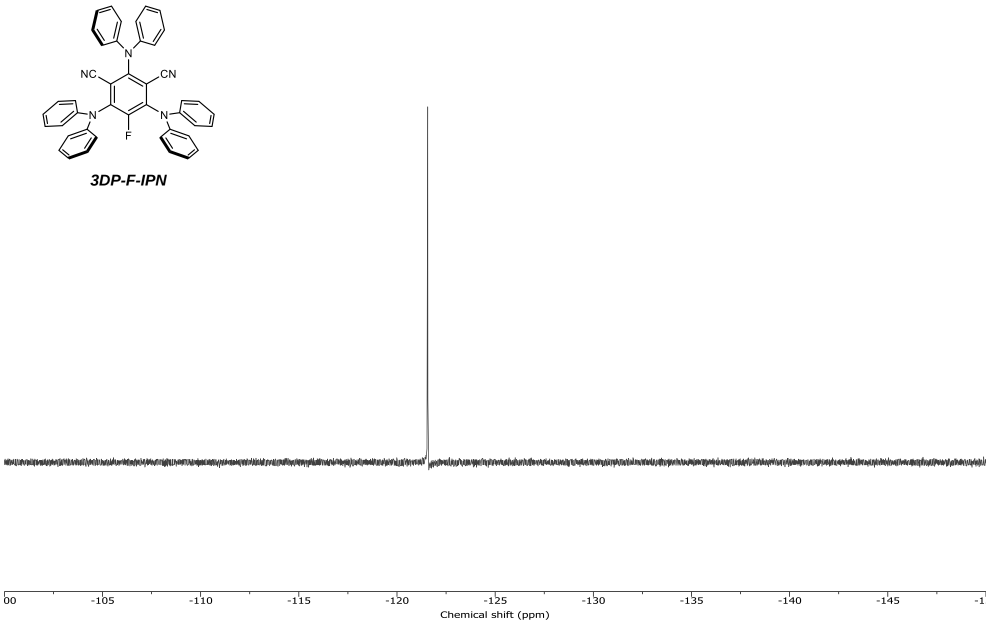

Supplementary Figure 43 ^{19}F NMR data of 3DP-F-IPN at RT (400 MHz, DMSO-d_6). ^{19}F NMR (377 MHz, DMSO-d_6): δ -121.56.

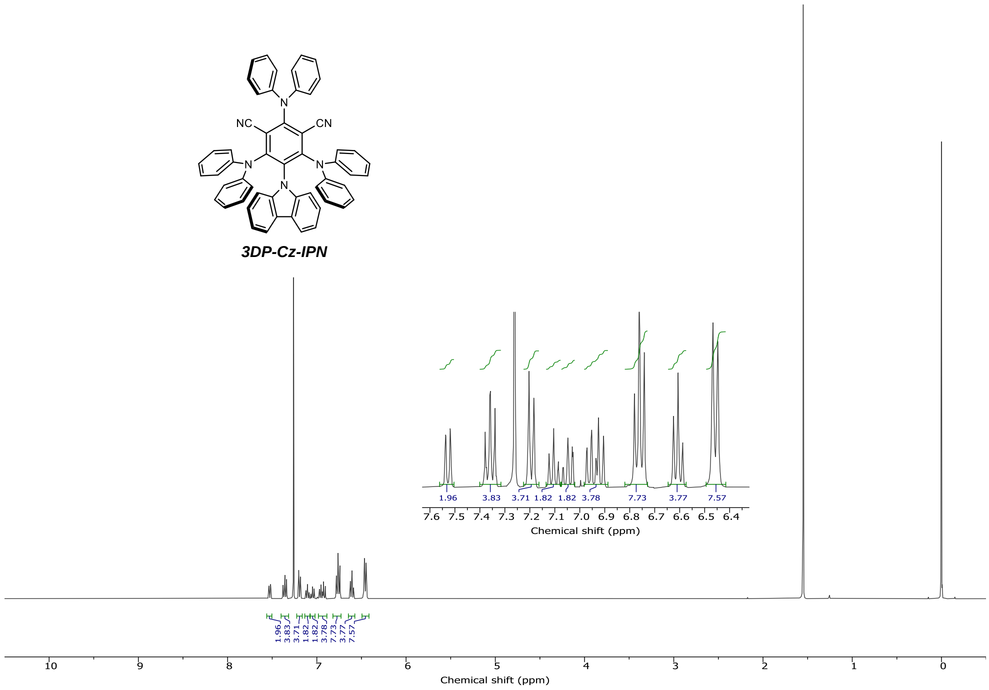

Supplementary Figure 44 ^{1}H NMR data of 3DP-Cz-IPN at RT (400 MHz, $CDCl_3$). ^{1}H NMR (400 MHz, $CDCl_3$): δ 7.55–7.50 (d, 2H), 7.40–7.35 (t, 4H), 7.20–7.15 (d, 4H), 7.14–7.08 (t, 2H), 7.07–7.03 (t, 2H), 6.98– 6.88 (dt, 4H), 6.80–6.71 (t, 8H), 6.65–6.58 (t, 4H), 6.48–6.40 (t, 8H).

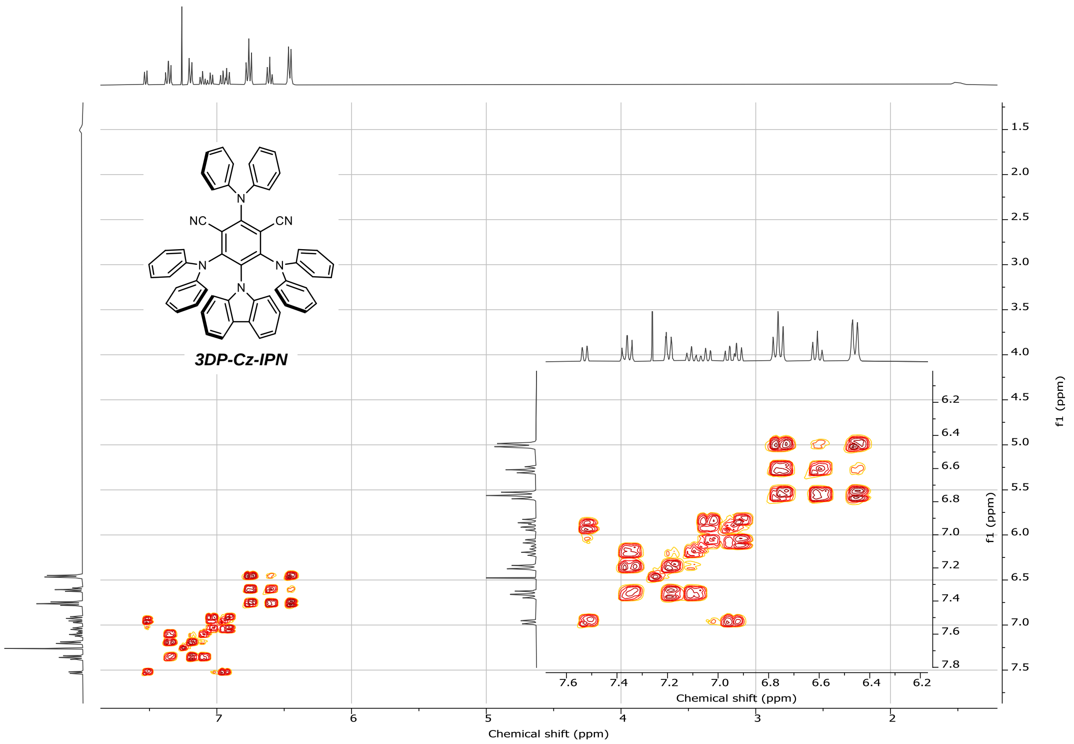

Supplementary Figure 45 COSY NMR data of 3DP-Cz-IPN at RT (400 MHz, $CDCl_3$).

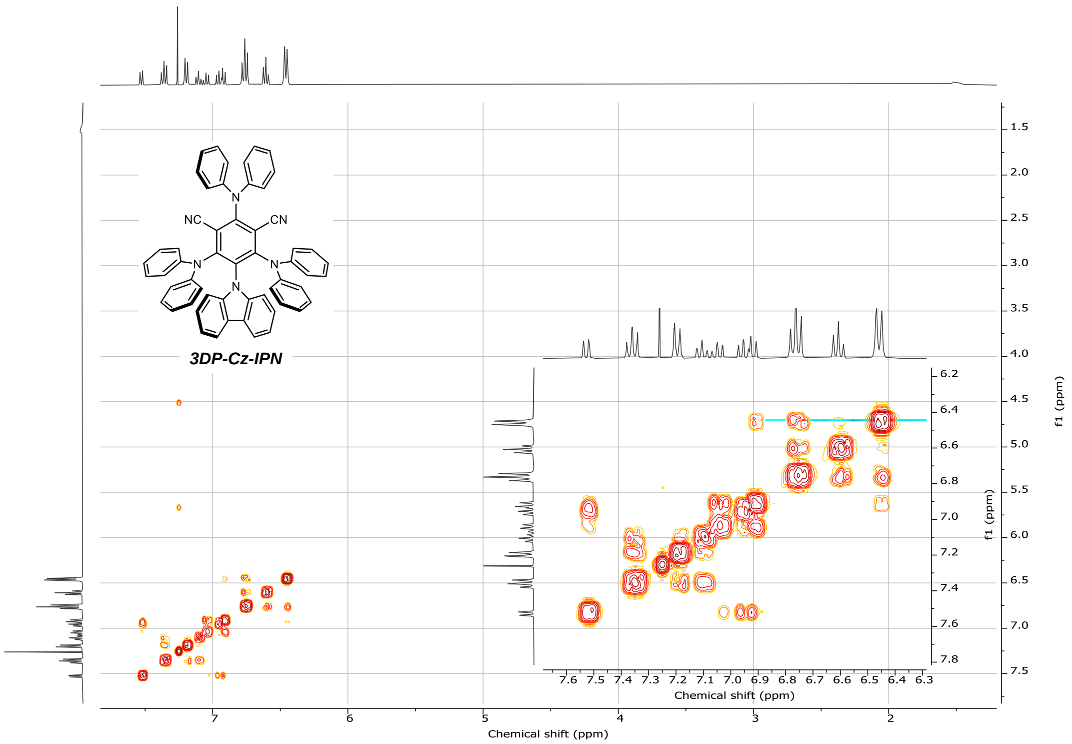

Supplementary Figure 46 NOESY NMR data of 3DP-Cz-IPN at RT (400 MHz, $CDCl_3$).

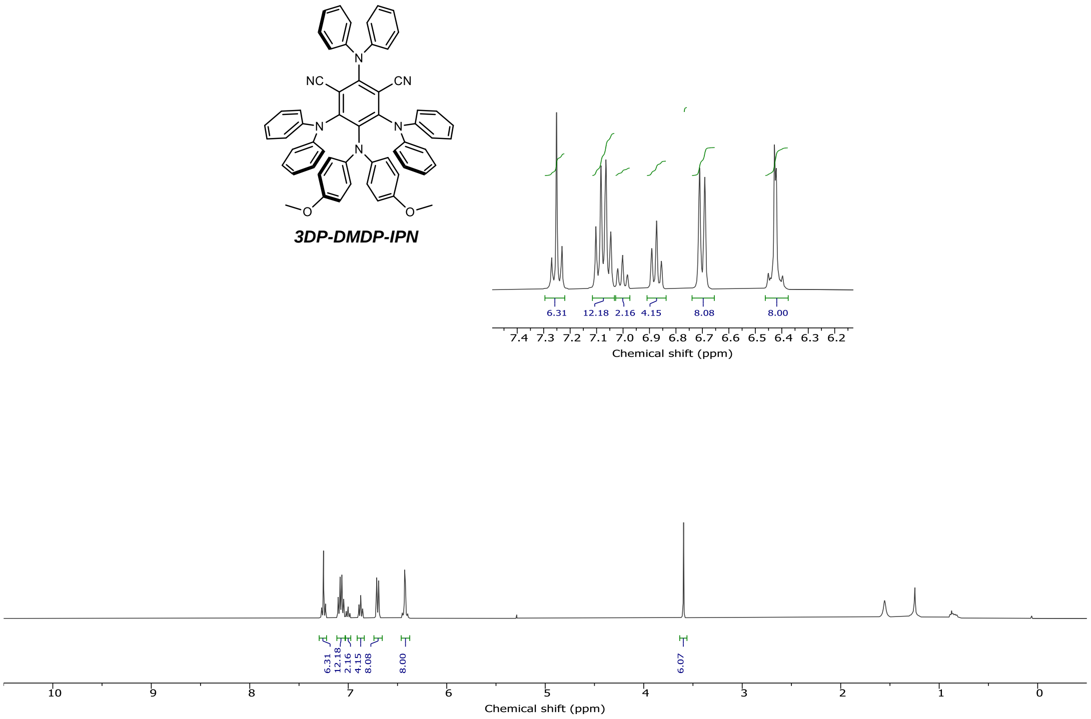

Supplementary Figure 47 ^{1}H NMR data of 3DP-DMDP-IPN at RT (400 MHz, $CDCl_3$). ^{1}H NMR (400 MHz, $CDCl_3$): δ 7.25 (d, 4H), 7.07 (m, 12H), 7.00 (t, 2H), 6.87 (t, 4H), 6.70 (d, 8H), 6.46–6.38 (m, 8H), 3.60 (s, 6H).

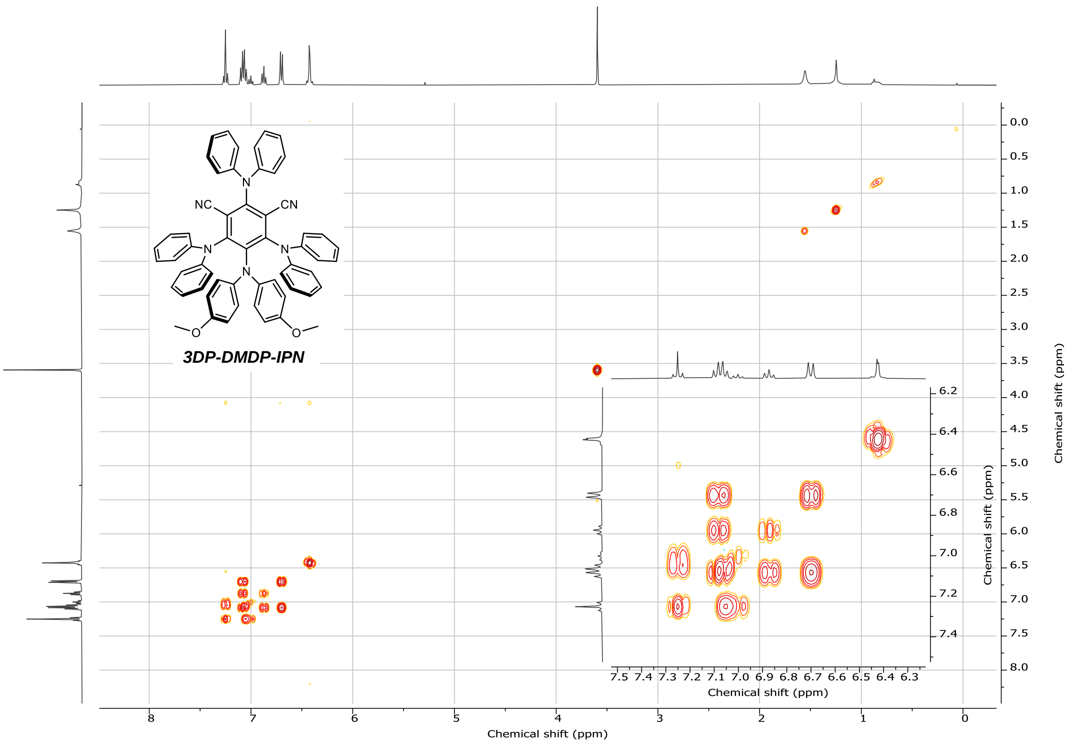

Supplementary Figure 48 COSY NMR data of 3DP-DMSO-IPN at RT (400 MHz, DMSO-d_6).

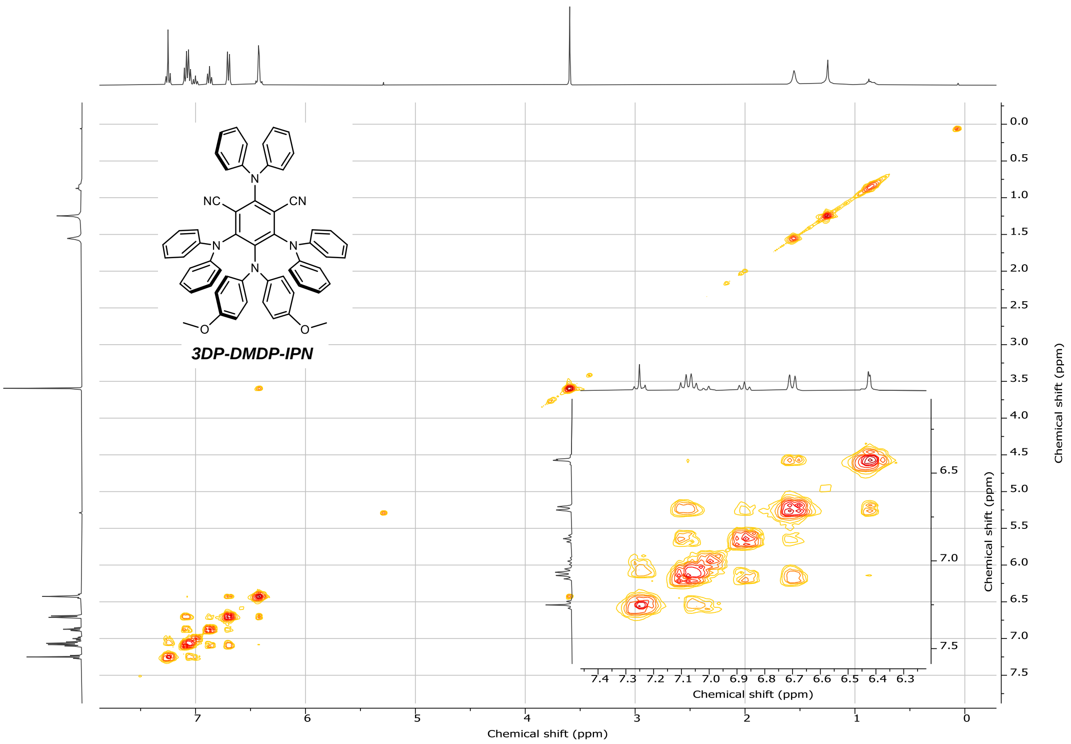

Supplementary Figure 49 NOESY NMR data of 3DP-DMSO-IPN at RT (400 MHz, DMSO-d_6).

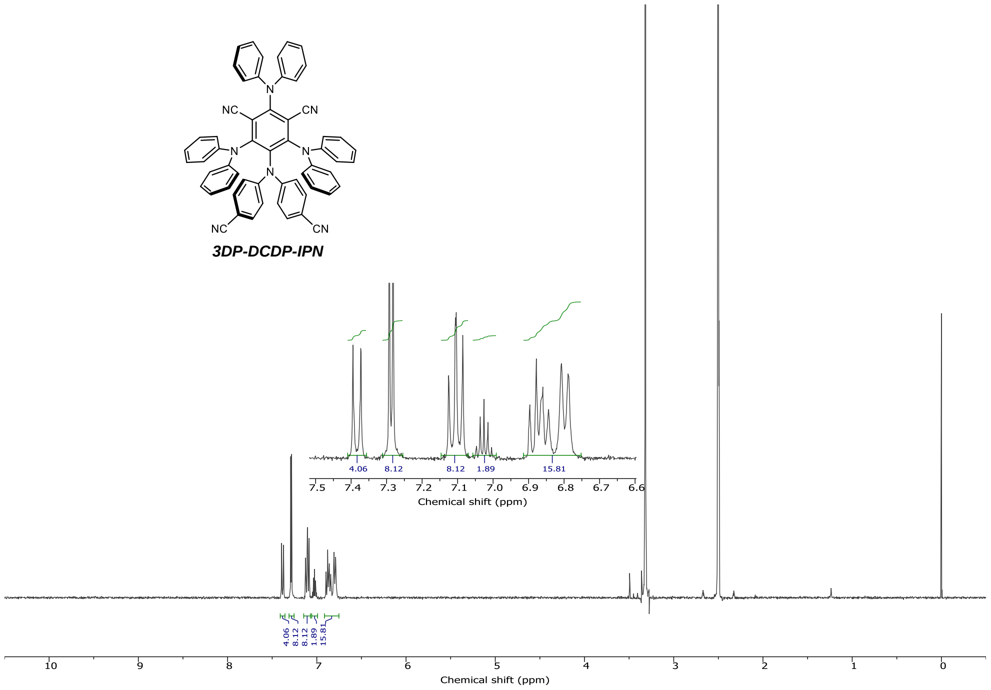

Supplementary Figure 50 ^{1}H NMR data of 3DP-DCDP-IPN at RT (400 MHz, DMSO-d_6). ^{1}H NMR (400 MHz, DMSO-d_6): δ 7.40–7.35 (d, 4H), 7.30–7.25 (d, 8H), 7.13–7.07 (t, 8H), 7.05–7.00 (q, 2H), 6.90–6.75 (m, 16H).

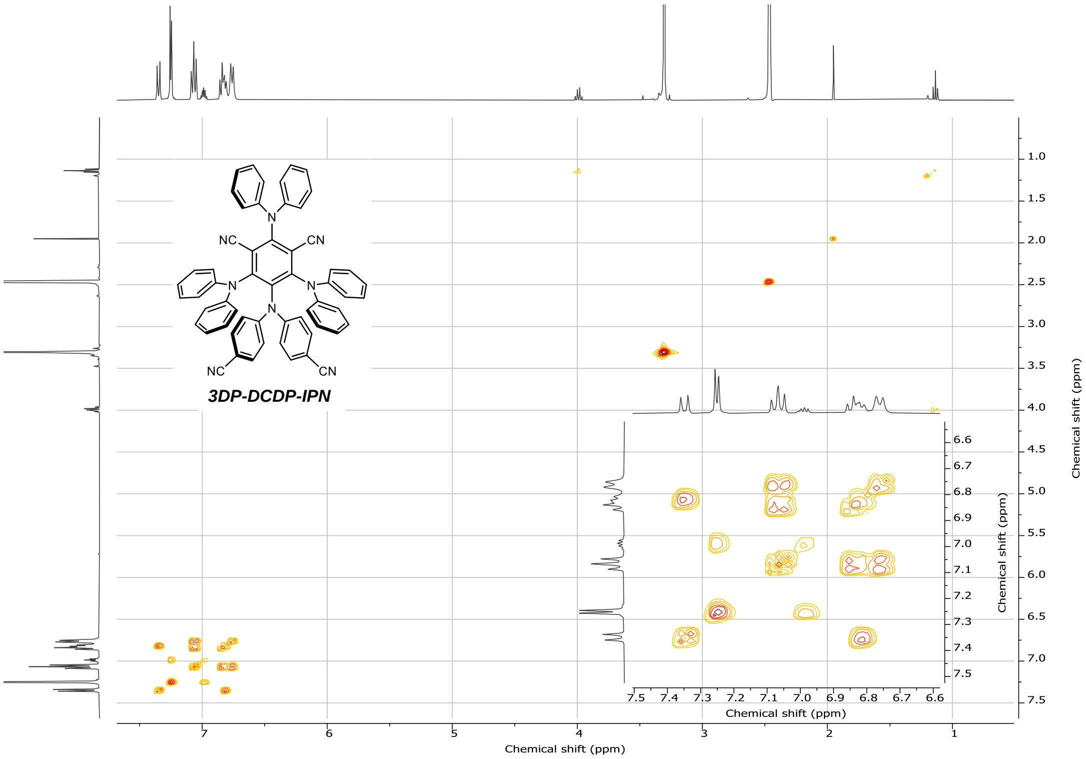

Supplementary Figure 51 COSY NMR data of 3DP-DCDP-IPN at RT (400 MHz, DMSO-d_6).

.

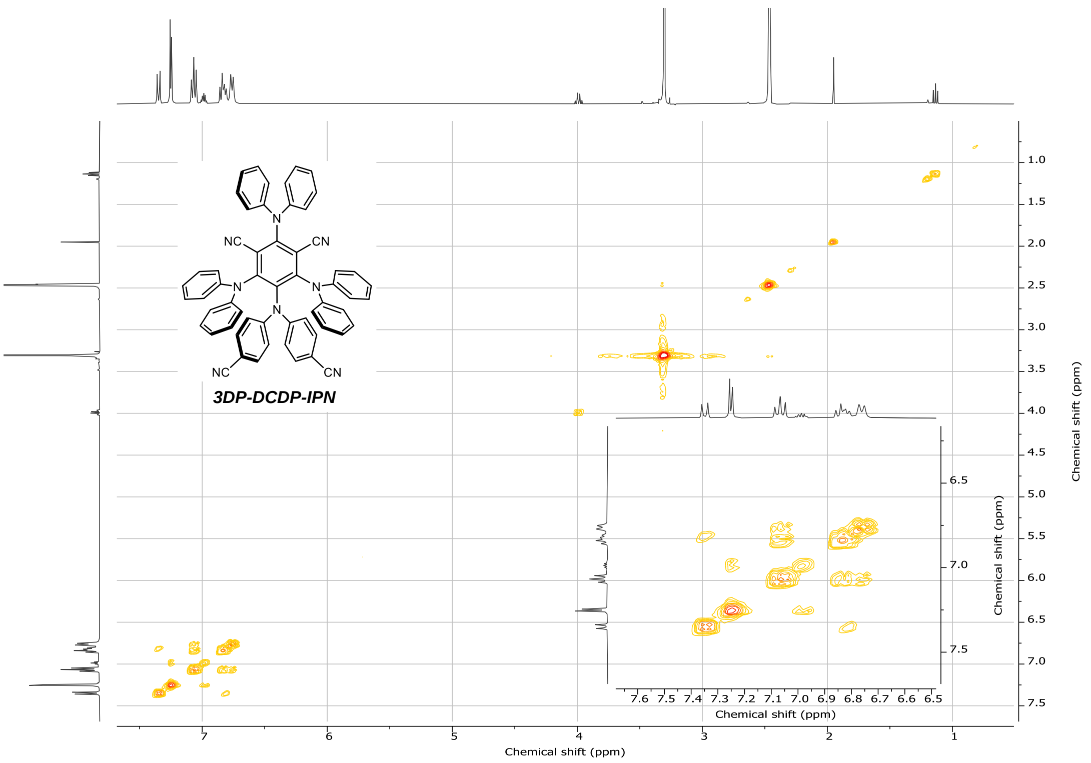

Supplementary Figure 52 NOSEY NMR data of 3DP-DCDP-IPN at RT (400 MHz, DMSO-d_6).

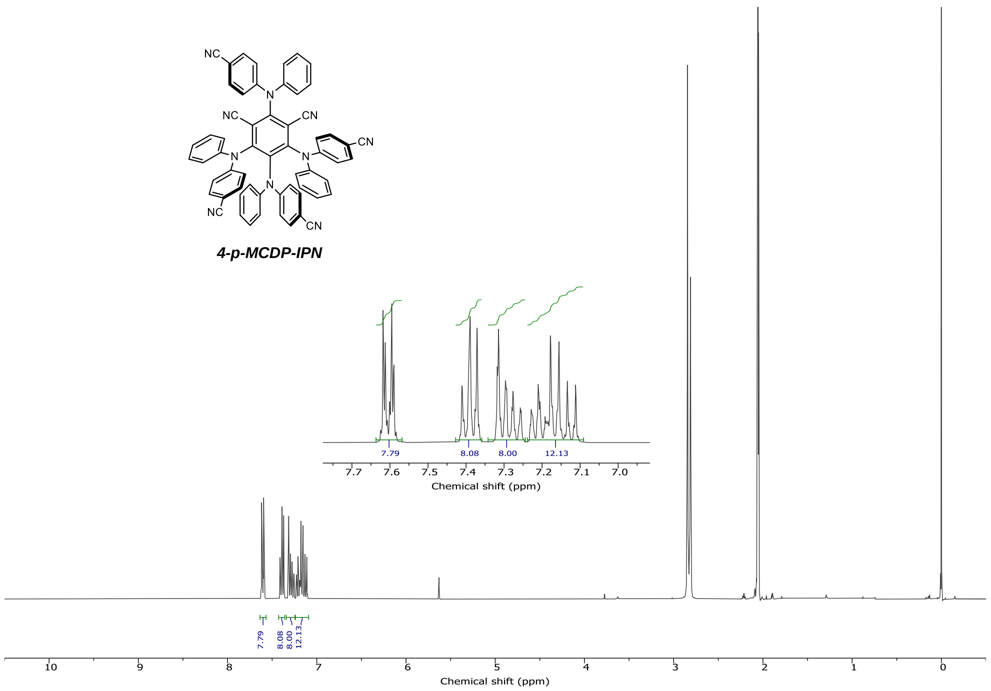

Supplementary Figure 53 ^{1}H NMR data of 4-p-MCDP-IPN at RT (400 MHz, acetone-d_6). ^{1}H NMR (400 MHz, acetone-d_6): δ 7.64–7.57 (m, 8H), 7.39 (dd, 8H), 7.34–7.24 (m, 8H), 7.24–7.09 (m, 12H).

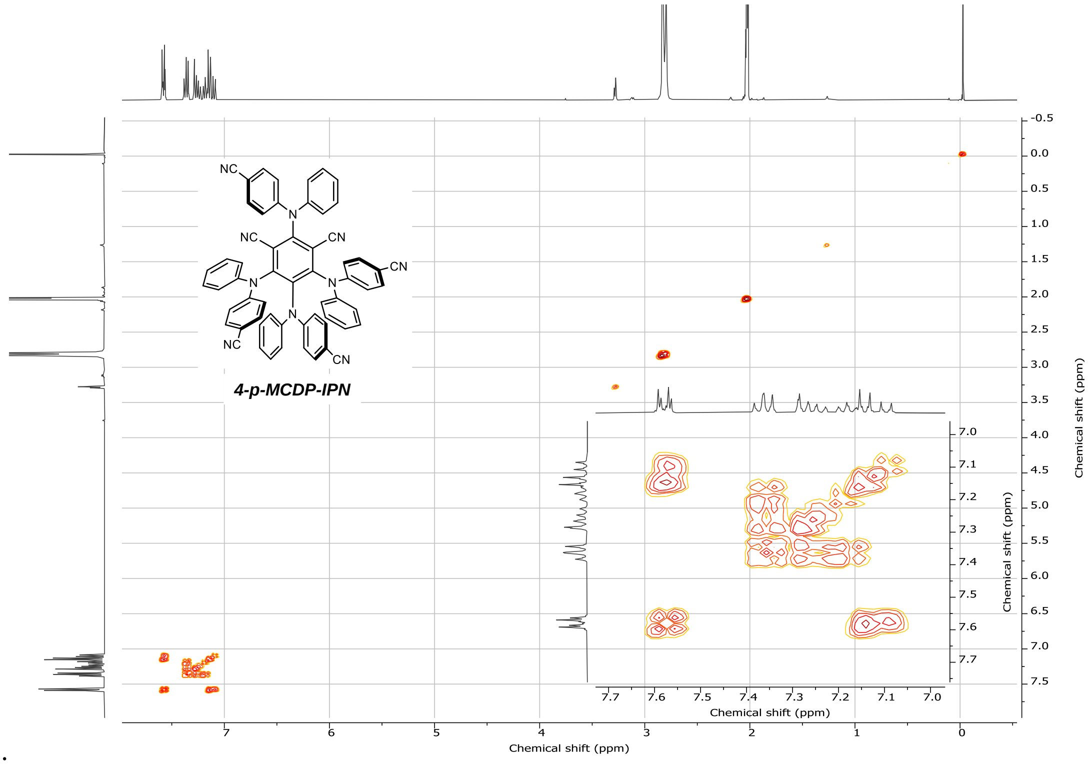

Supplementary Figure 54 COSY NMR data of 4-p-MCDP-IPN at RT (600 MHz, acetone-d_6).

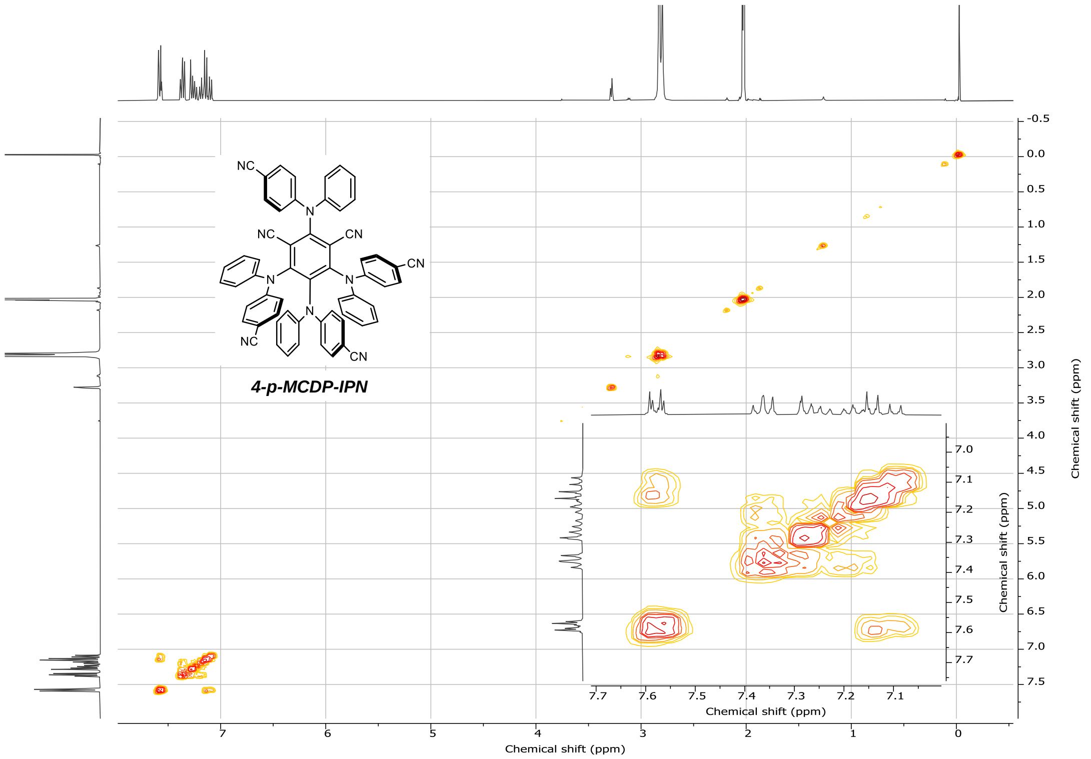

Supplementary Figure 55 NOESY NMR data of 4-p-MCDP-IPN at RT (400 MHz, acetone-d_6).

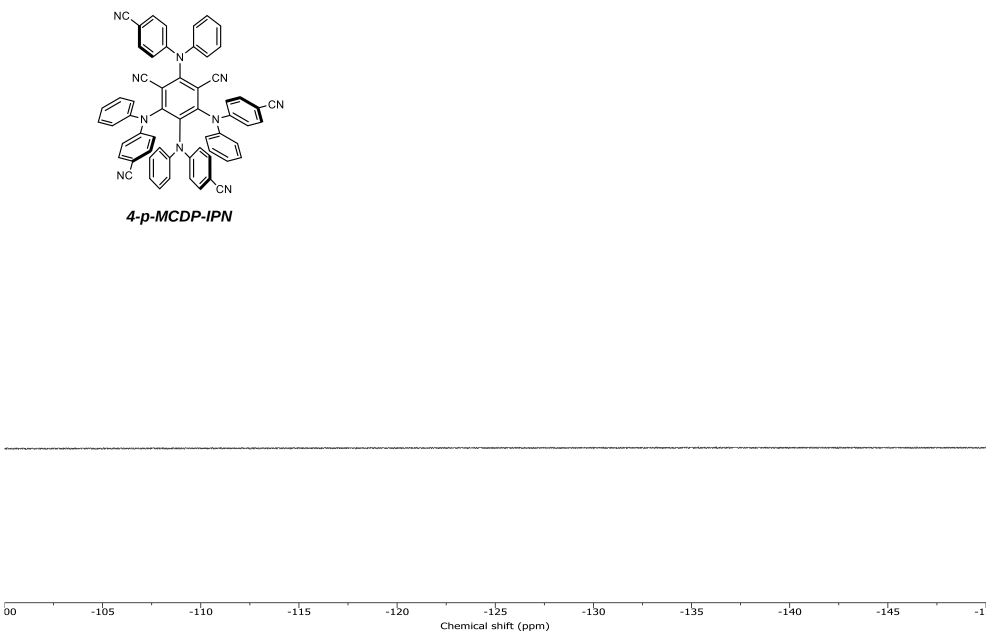

Supplementary Figure 56 ^{19}F NMR data of 4-p-MCDP-IPN at RT (565 MHz, DMSO-d_6). ^{19}F NMR (377 MHz, DMSO-d_6): no peak

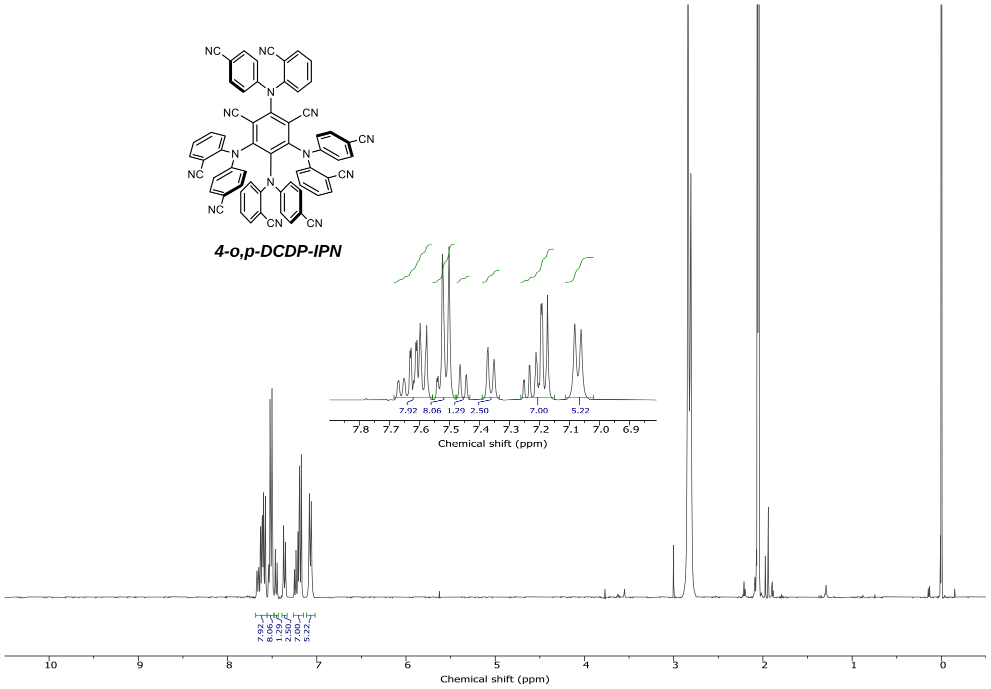

Supplementary Figure 57 ^{1}H NMR data of 4-o,p-DCDP-IPN at RT (400 MHz, acetone-d_6). ^{1}H NMR (400 MHz, acetone-d_6): δ 7.68–7.56 (m, 8H), 7.51 (d, 8H), 7.45 (d, 1H), 7.36 (d, 3H), 7.26–7.15 (m, 7H), 7.07 (d, 5H).

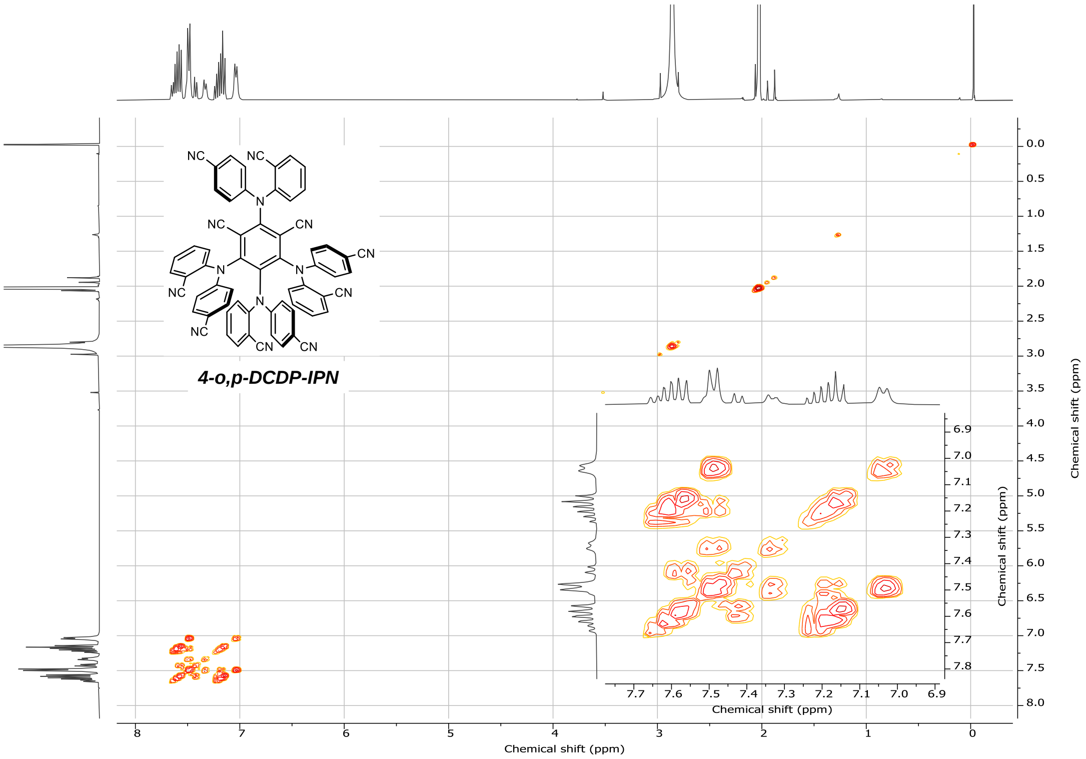

Supplementary Figure 58 COSY NMR data of 4-o,p-DCDP-IPN at RT (400 MHz, acetone-d_6).

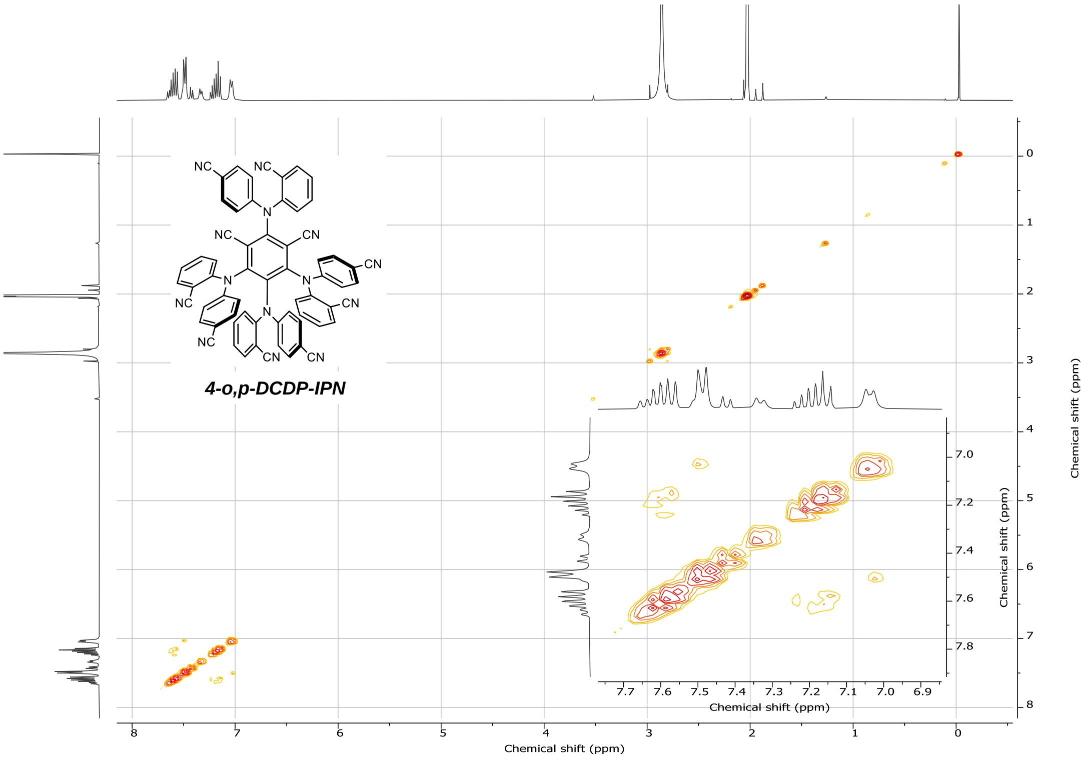

Supplementary Figure 59 NOESY NMR data of 4-o,p-DCDP-IPN at RT (400 MHz, acetone-d_6).

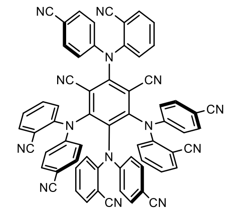

4-o,p-DCDP-IPN

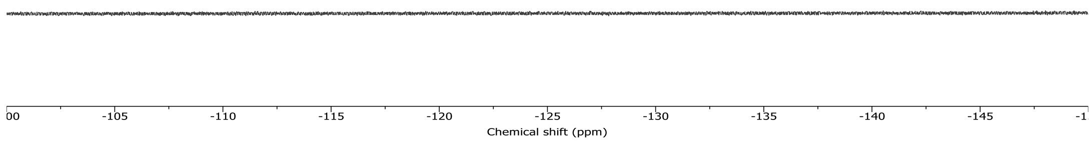

Supplementary Figure 60 ^{19}F NMR data of 4-o,p-DCDP-IPN at RT (565 MHz, acetone-d_6). ^{19}F NMR (565 MHz, acetone-d_6): no peak

Supplementary Note 14. Validation of broad scope of aryl/alkyl halides

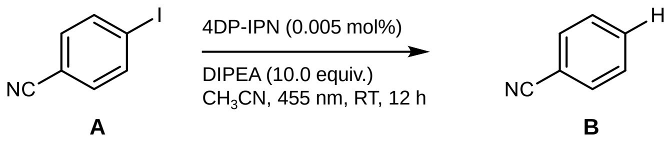

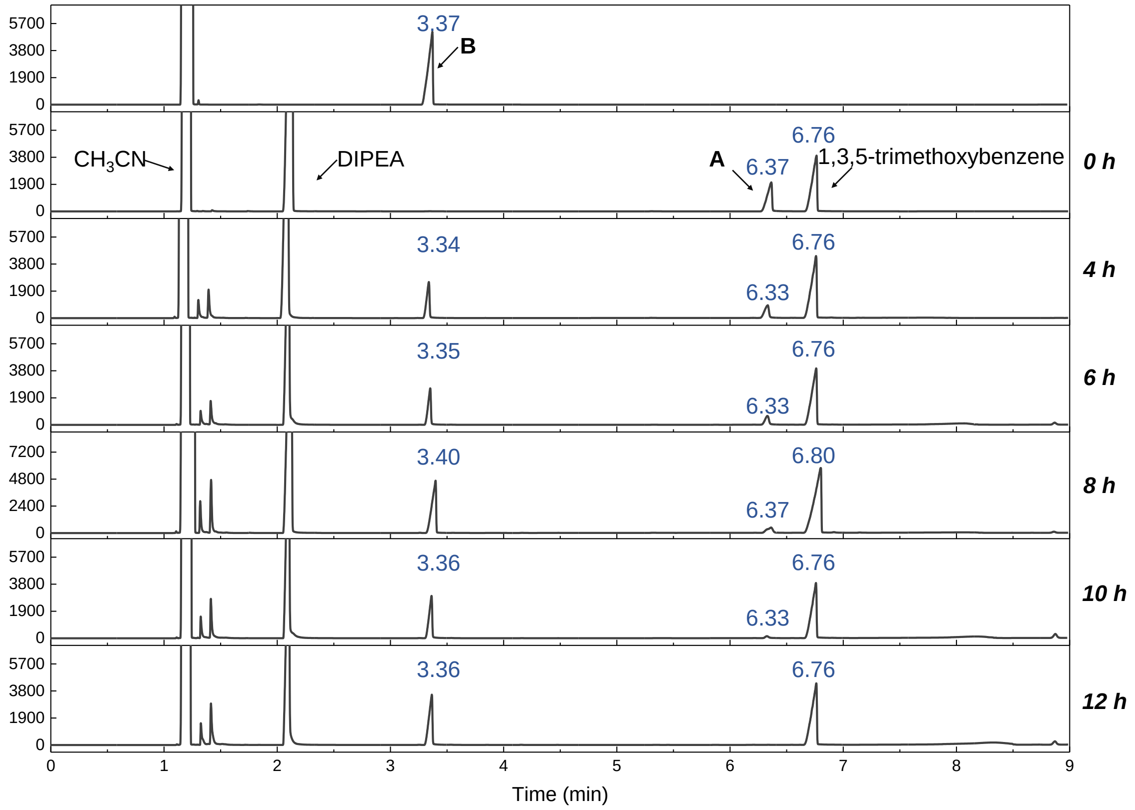

Supplementary Figure 61 Gas chromatography (GC-FID) spectra for photoredox reductive dehalogenation of 4-iodobenzonitrile. Yield was measured by GC-FID using 1,3,5-trimethoxybenzene as an internal standard.

Substrate screening (4-iodobenzonitrile), 0 h

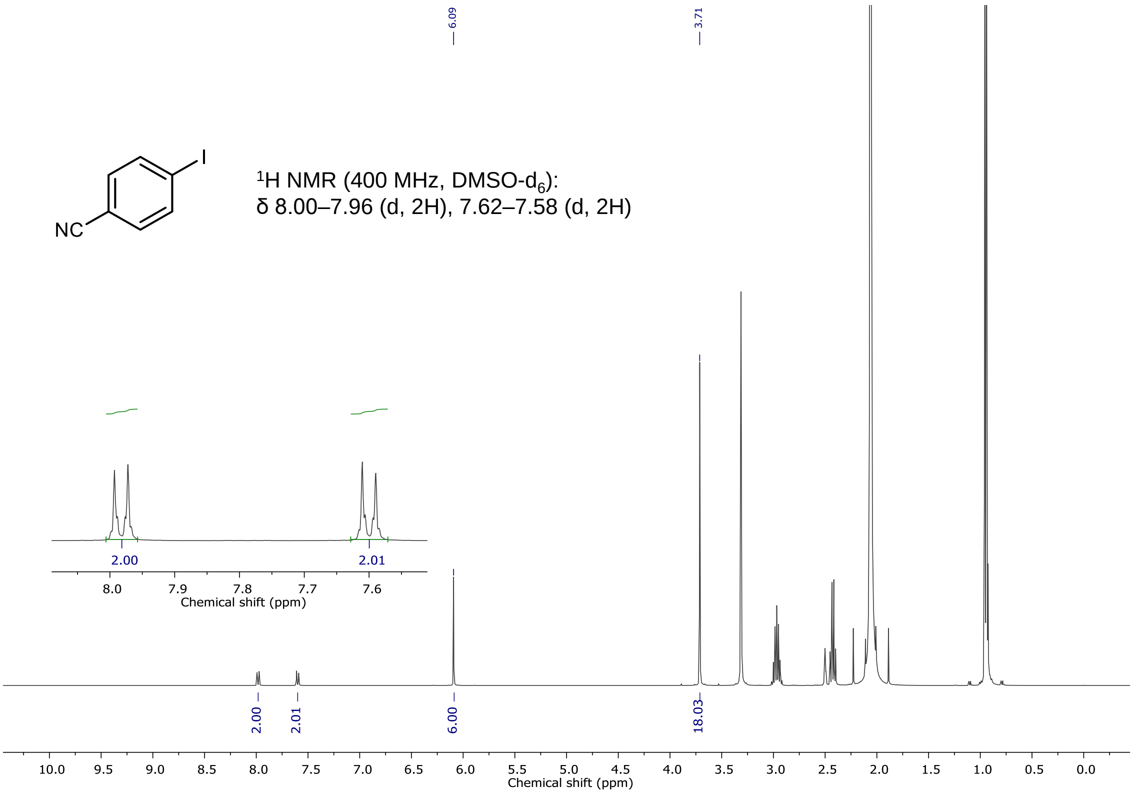

Substrate screening (4-iodobenzonitrile), 12 h

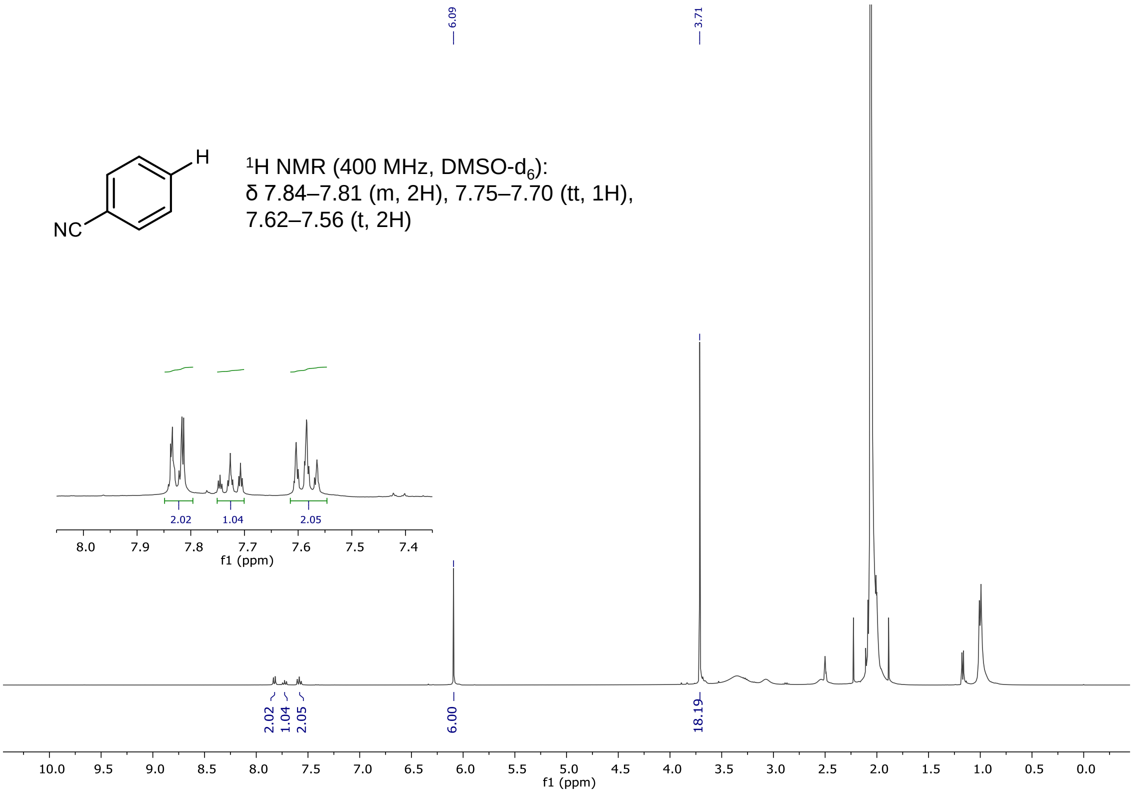

Supplementary Figure 62 ^{1}H NMR data of photoredox reductive dehalogenation for 4-iodobenzonitrile (400 MHz, DMSO-d_6); 1,3,5-trimethoxybenzene: δ 6.09 (s, 3H), 3.71 (s, 9H).

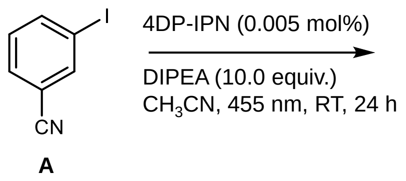

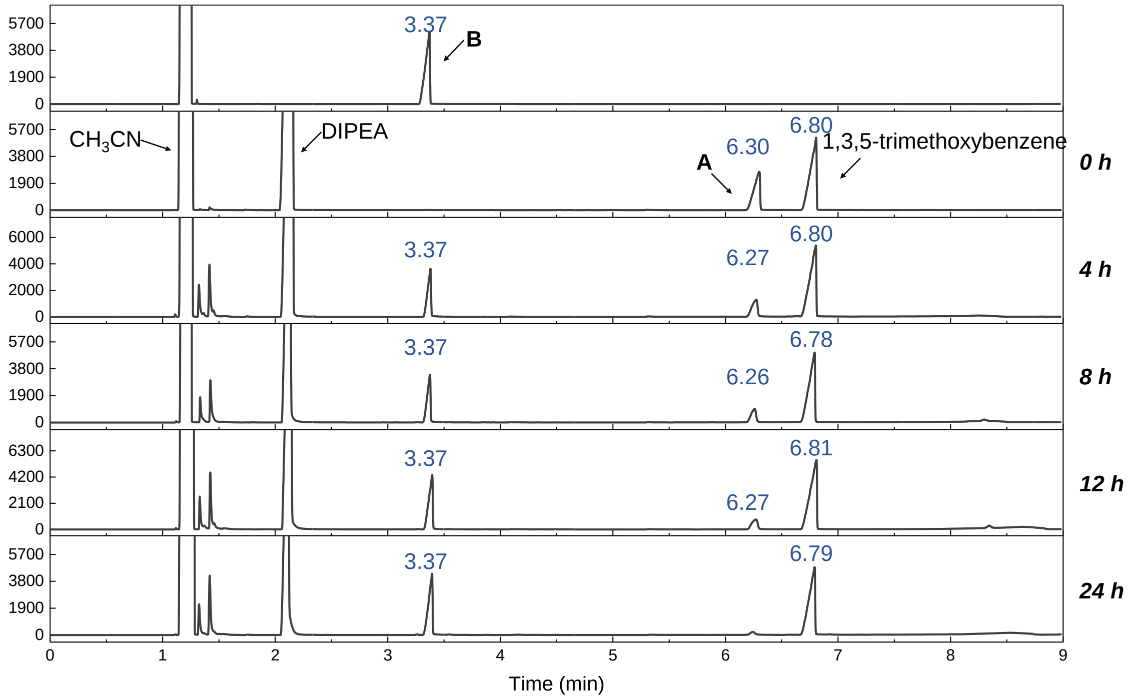

Supplementary Figure 63 GC-FID spectra of photoredox reductive dehalogenation for 3-iodobenzonitrile. Yield was measured by GC-FID using 1,3,5-trimethoxybenzene as an internal standard.

Substrate screening (3-iodobenzonitrile), 0 h

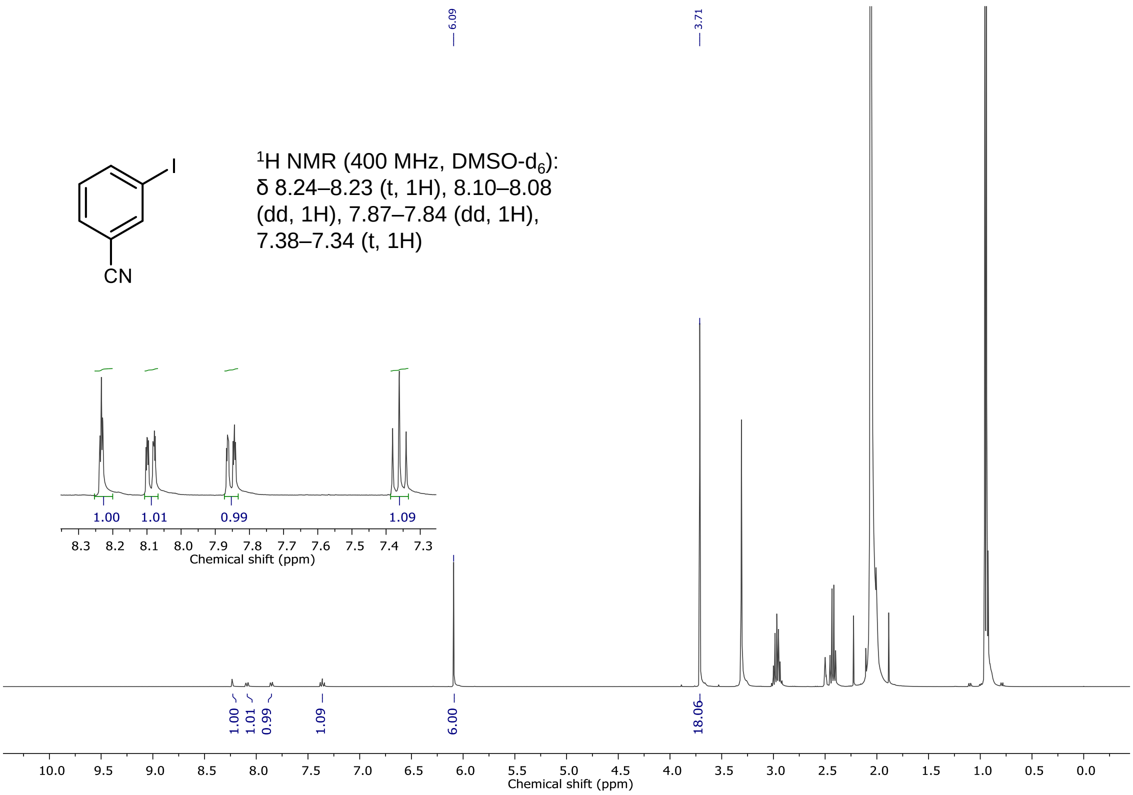

Substrate screening (3-iodobenzonitrile), 24 h

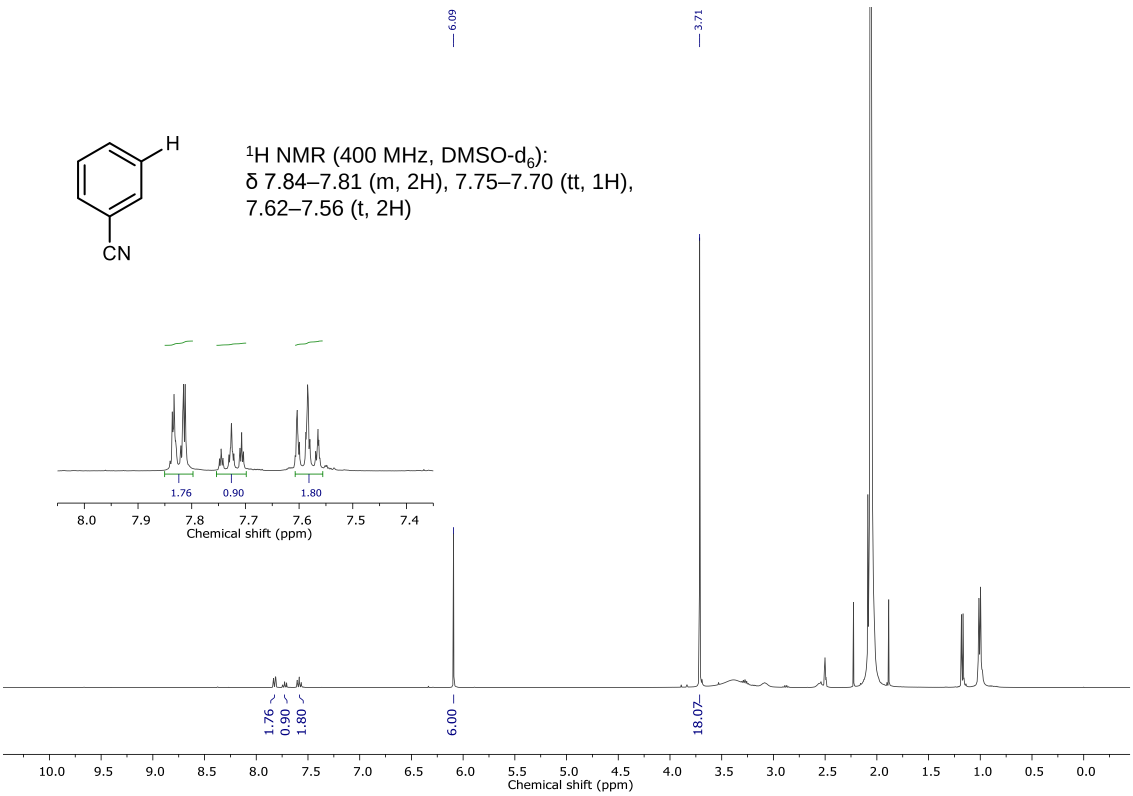

Supplementary Figure 64 ^{1}H NMR data of photoredox reductive dehalogenation for 3-iodobenzonitrile (400 MHz, DMSO-d_6); 1,3,5-trimethoxybenzene: δ 6.09 (s, 3H), 3.71 (s, 9H).

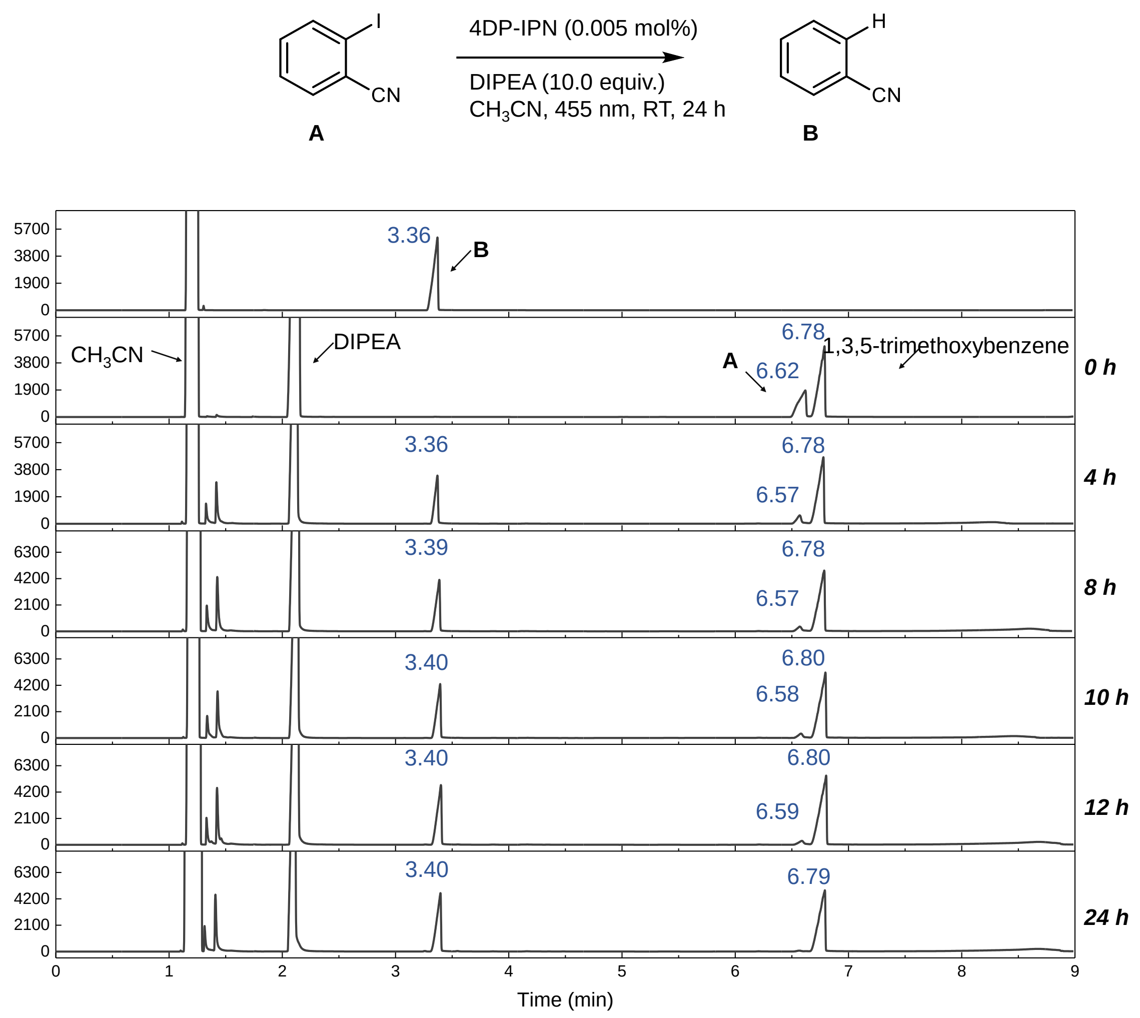

Supplementary Figure 65 GC-FID spectra of photoredox reductive dehalogenation for 2-iodobenzonitrile. Yield was measured by GC-FID using 1,3,5-trimethoxybenzene as an internal standard.

Substrate screening (2-iodobenzonitrile), 0 h

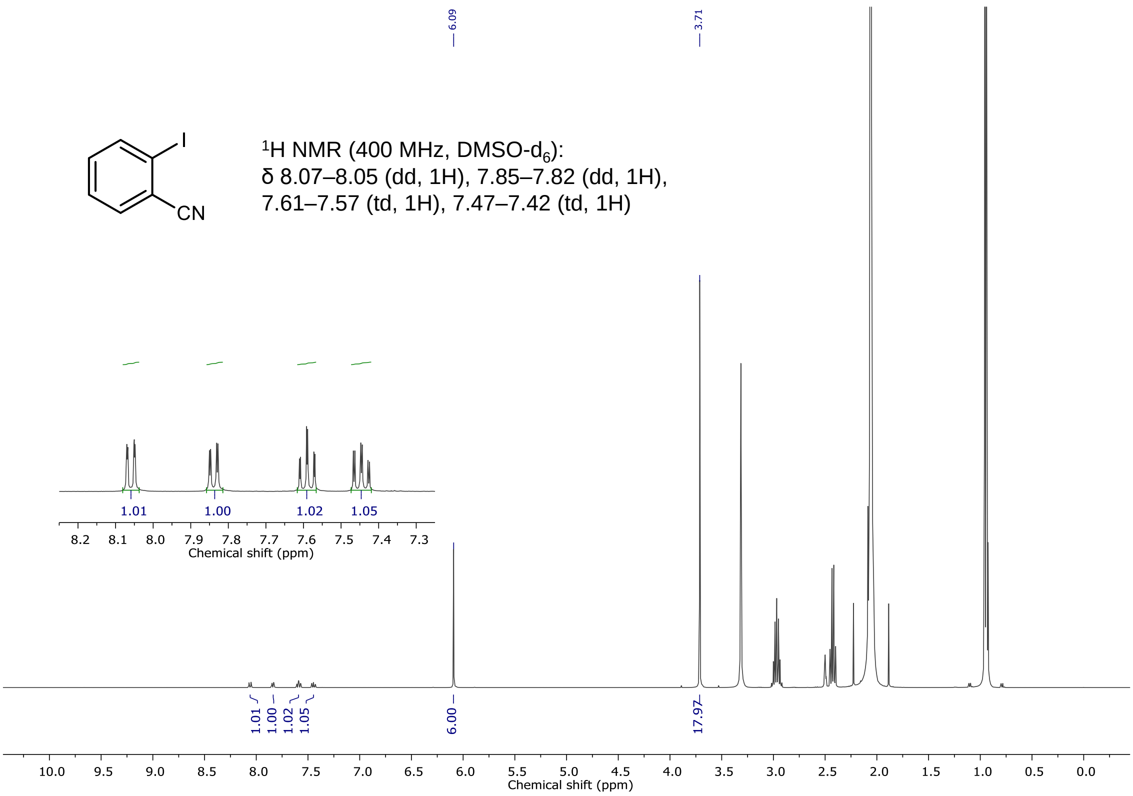

Substrate screening (2-iodobenzonitrile), 24 h

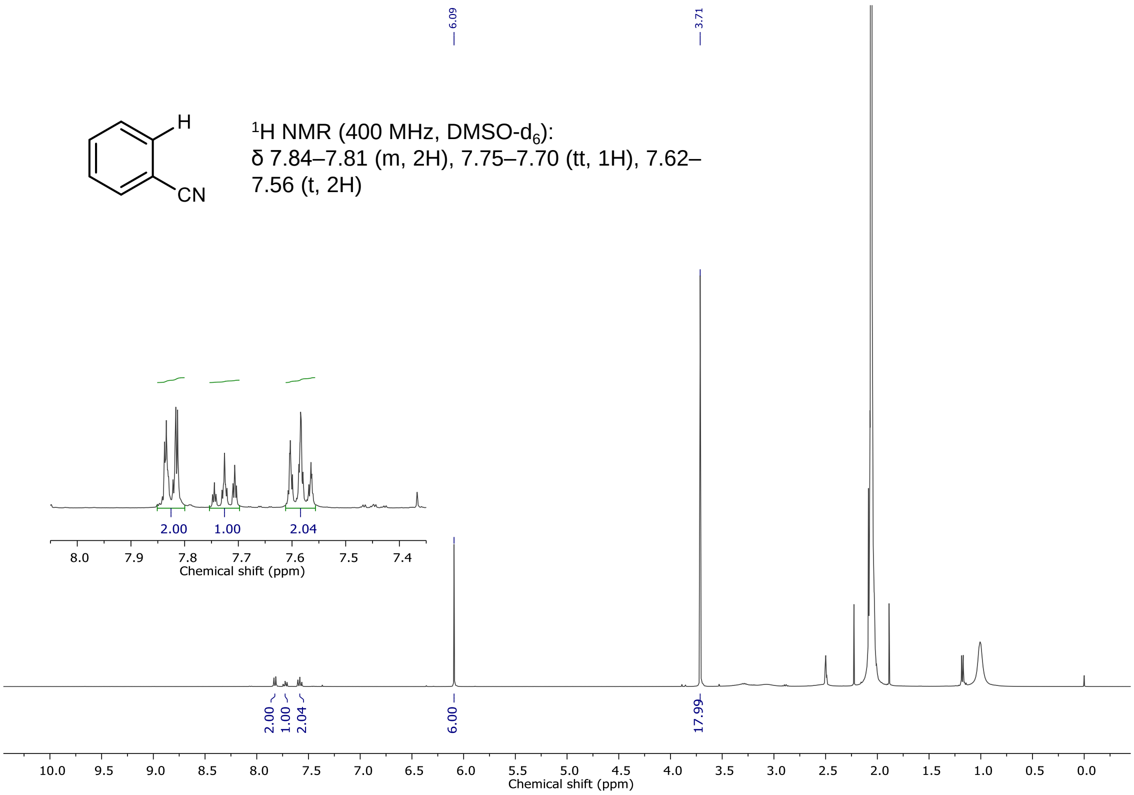

Supplementary Figure 66 ^{1}H NMR data of photoredox reductive dehalogenation for 2-iodobenzonitrile (400 MHz, DMSO-d_6); 1,3,5-trimethoxybenzene: δ 6.09 (s, 3H), 3.71 (s, 9H).

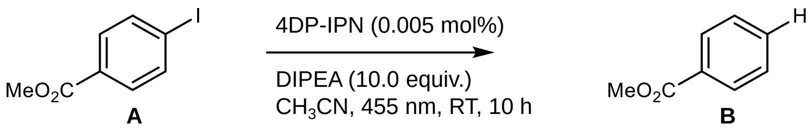

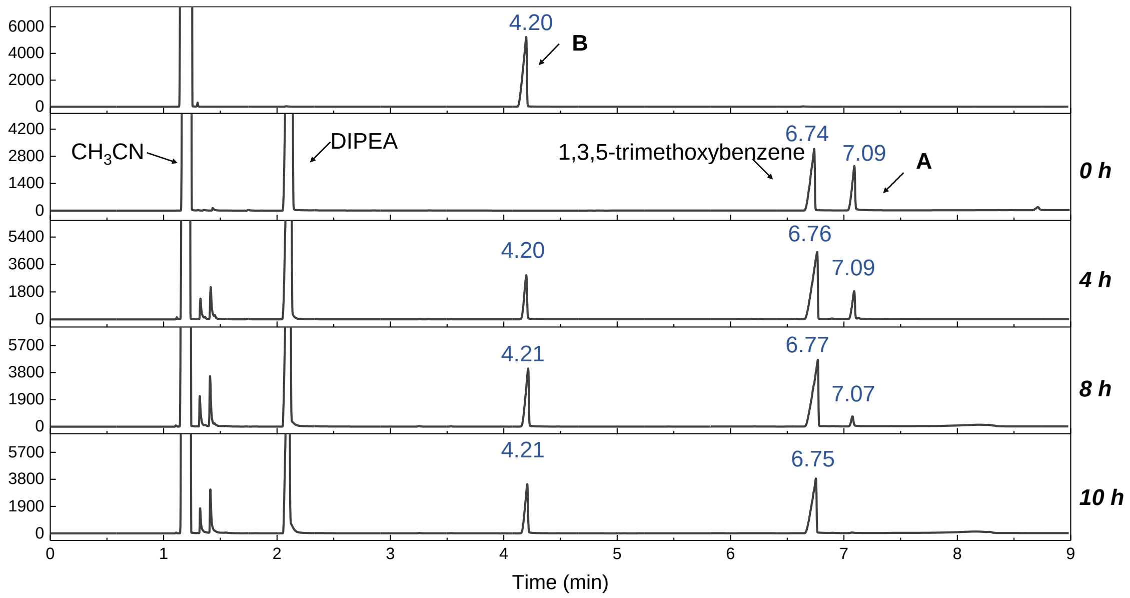

Supplementary Figure 67 GC-FID spectra of photoredox reductive dehalogenation for methyl-4-iodobenzoate. Yield was measured by GC-FID using 1,3,5-trimethoxybenzene as an internal standard.

Substrate screening (methyl 4-iodobenzoate), 0 h

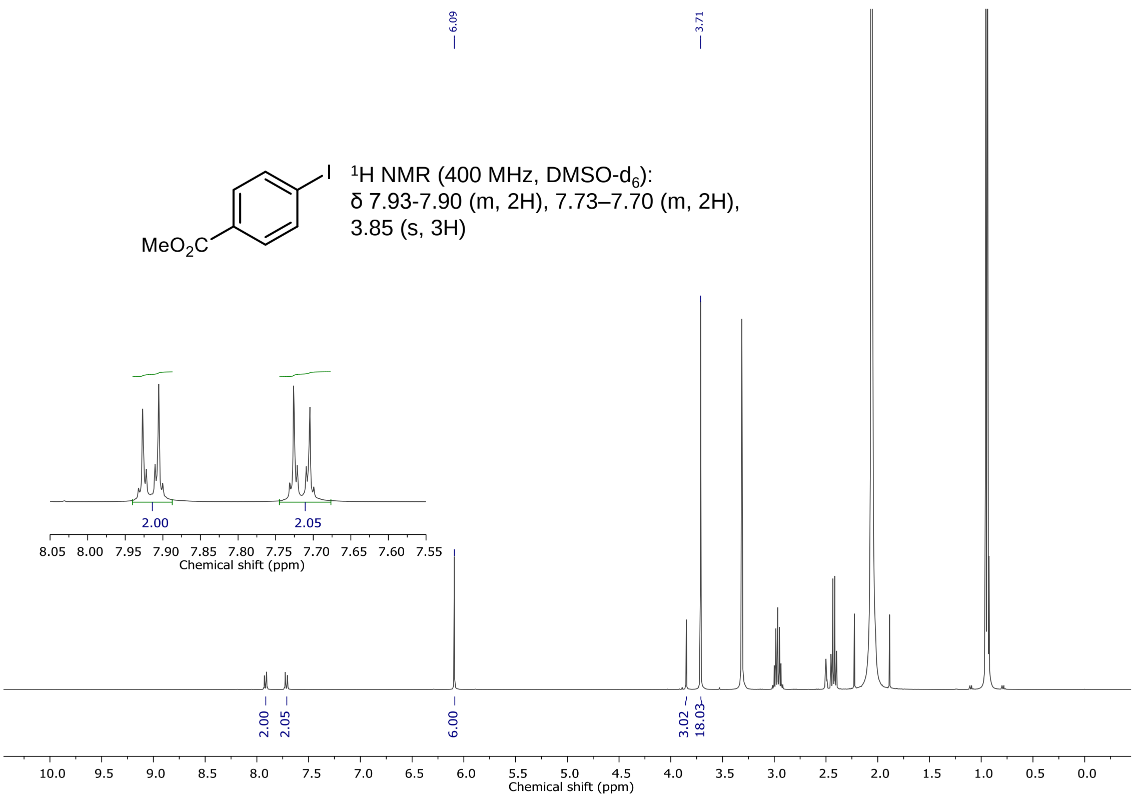

Substrate screening (methyl 4-iodobenzoate), 10 h

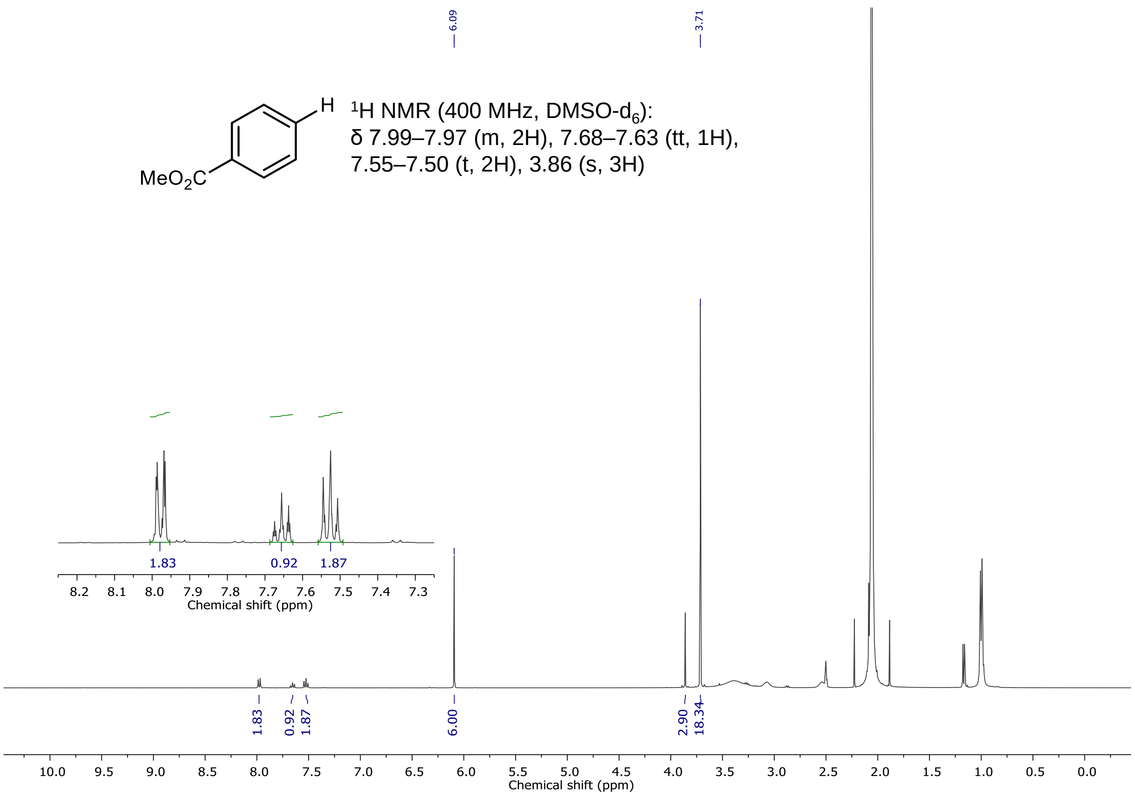

Supplementary Figure 68 ¹H NMR data of photoredox reductive dehalogenation for methyl 4-iodobenzoate (400 MHz, DMSO-d_6); 1,3,5-trimethoxybenzene: δ 6.09 (s, 3H), 3.71 (s, 9H).

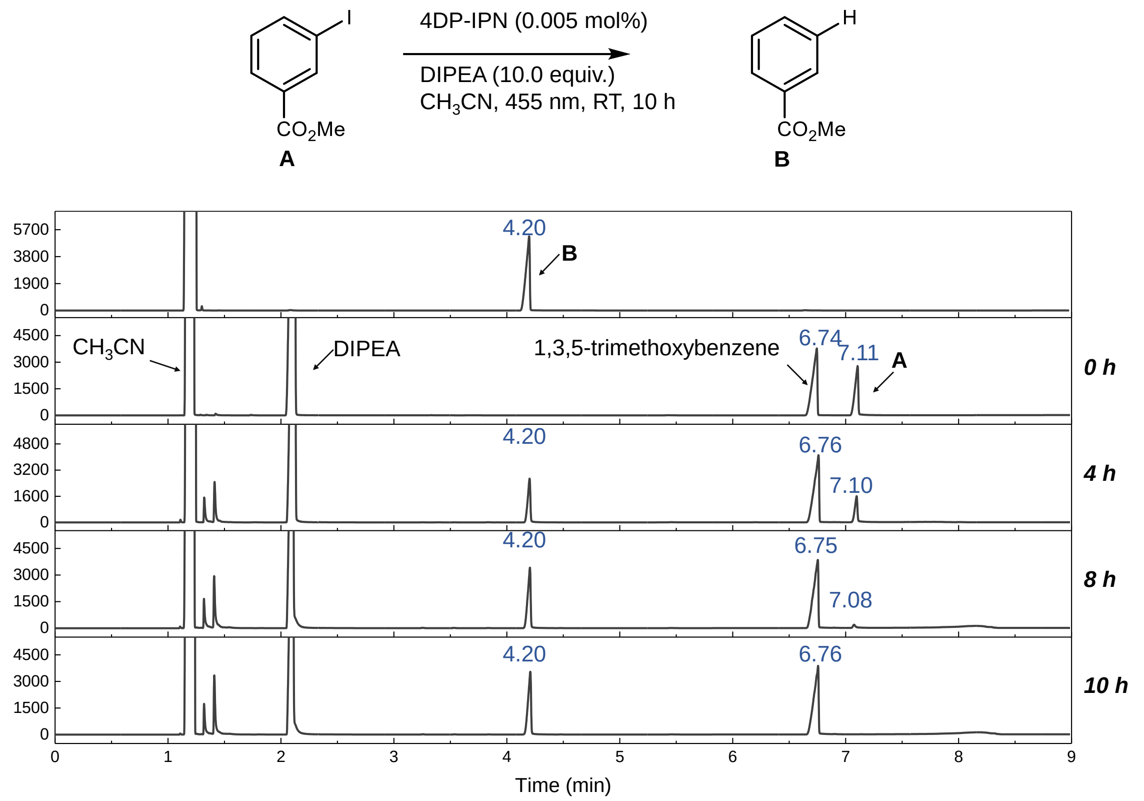

Supplementary Figure 69 GC-FID spectra of photoredox reductive dehalogenation for methyl-3-iodobenzoate. Yield was measured by GC-FID using 1,3,5-trimethoxybenzene as an internal standard.

Substrate screening (methyl 3-iodobenzoate), 0 h

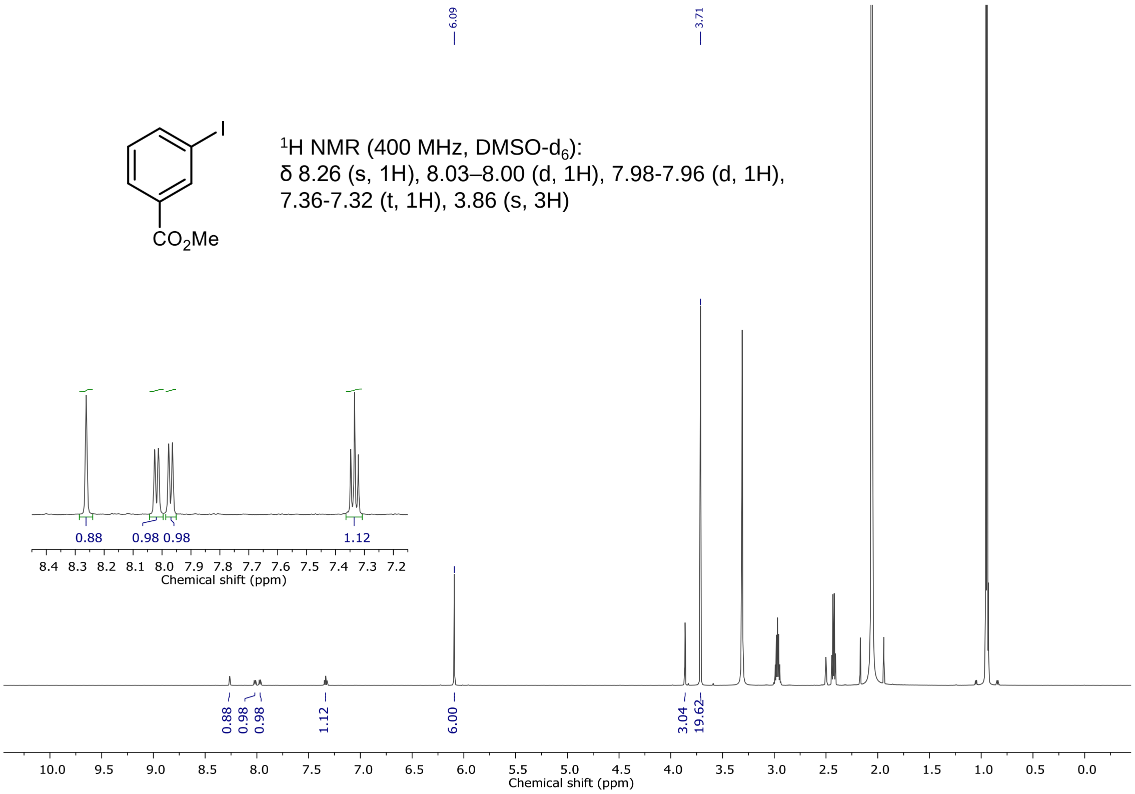

Substrate screening (methyl 3-iodobenzoate), 10 h

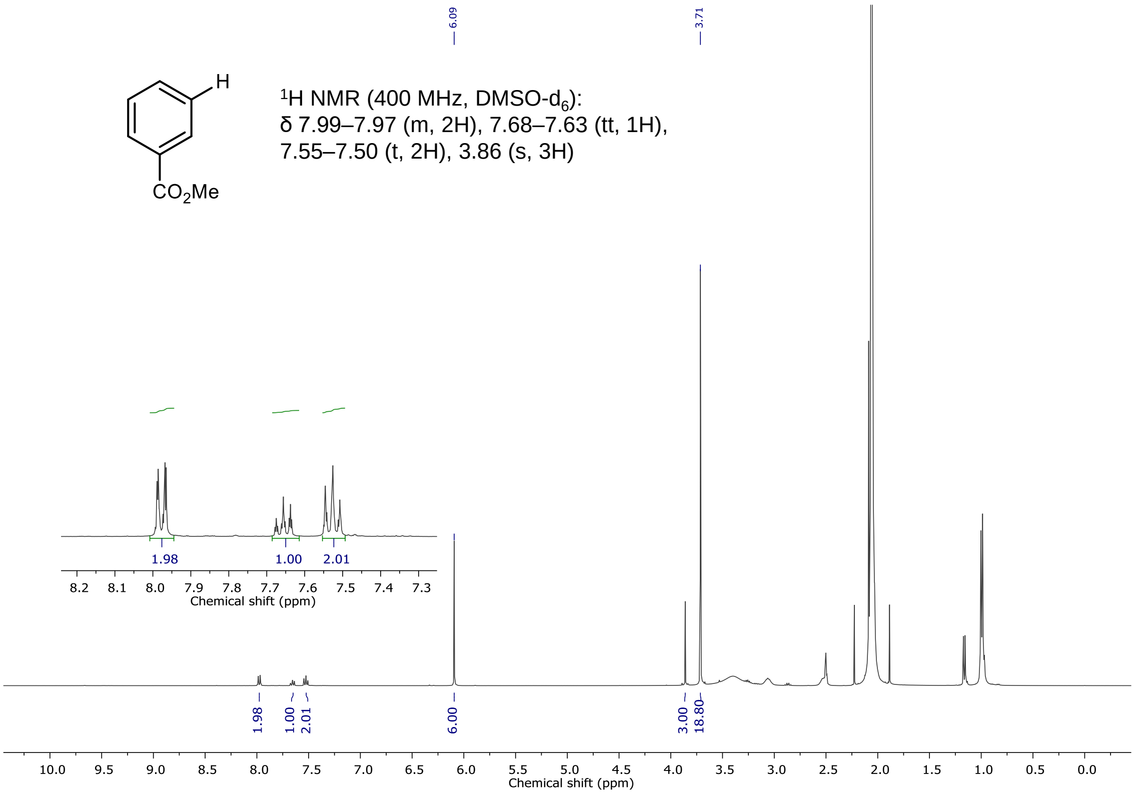

Supplementary Figure 70 ^{1}H NMR data of photoredox reductive dehalogenation for methyl 3-iodobenzoate (400 MHz, DMSO-d_6); 1,3,5-trimethoxybenzene: δ 6.09 (s, 3H), 3.71 (s, 9H).

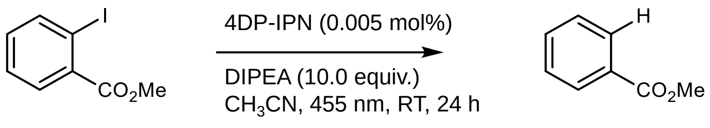

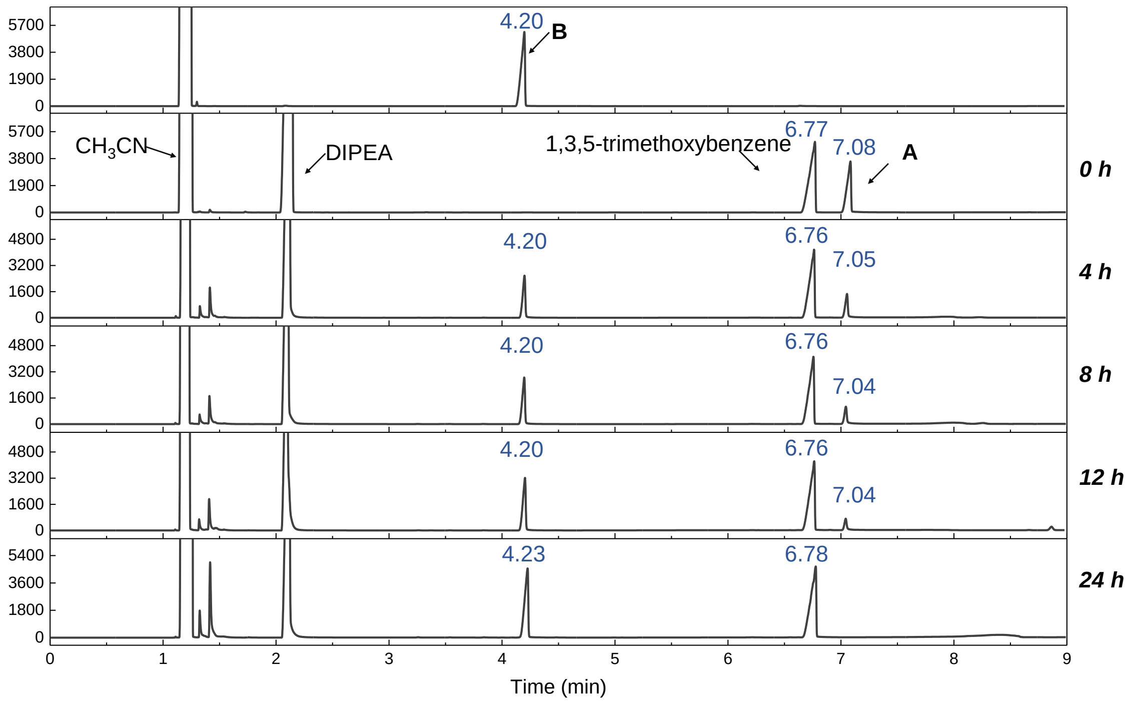

Supplementary Figure 71 GC-FID spectra of photoredox reductive dehalogenation for methyl-2-iodobenzoate. Yield was measured by GC-FID using 1,3,5-trimethoxybenzene as an internal standard.

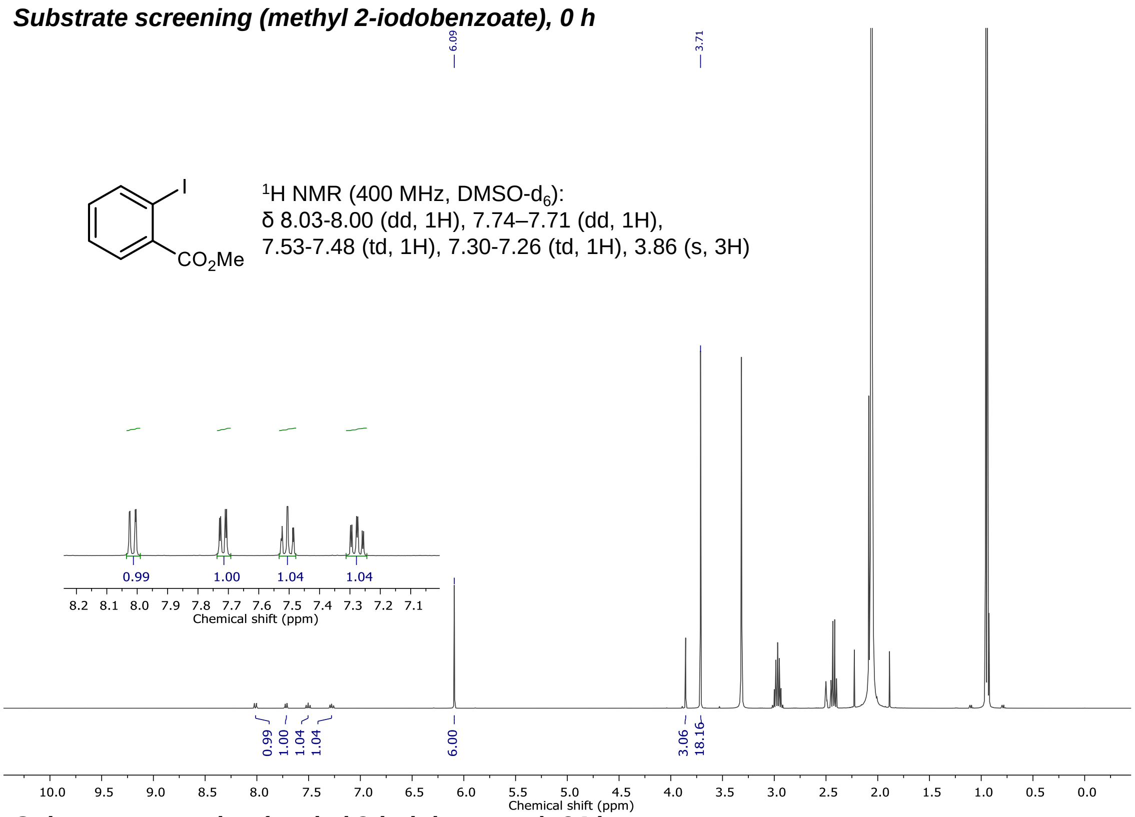

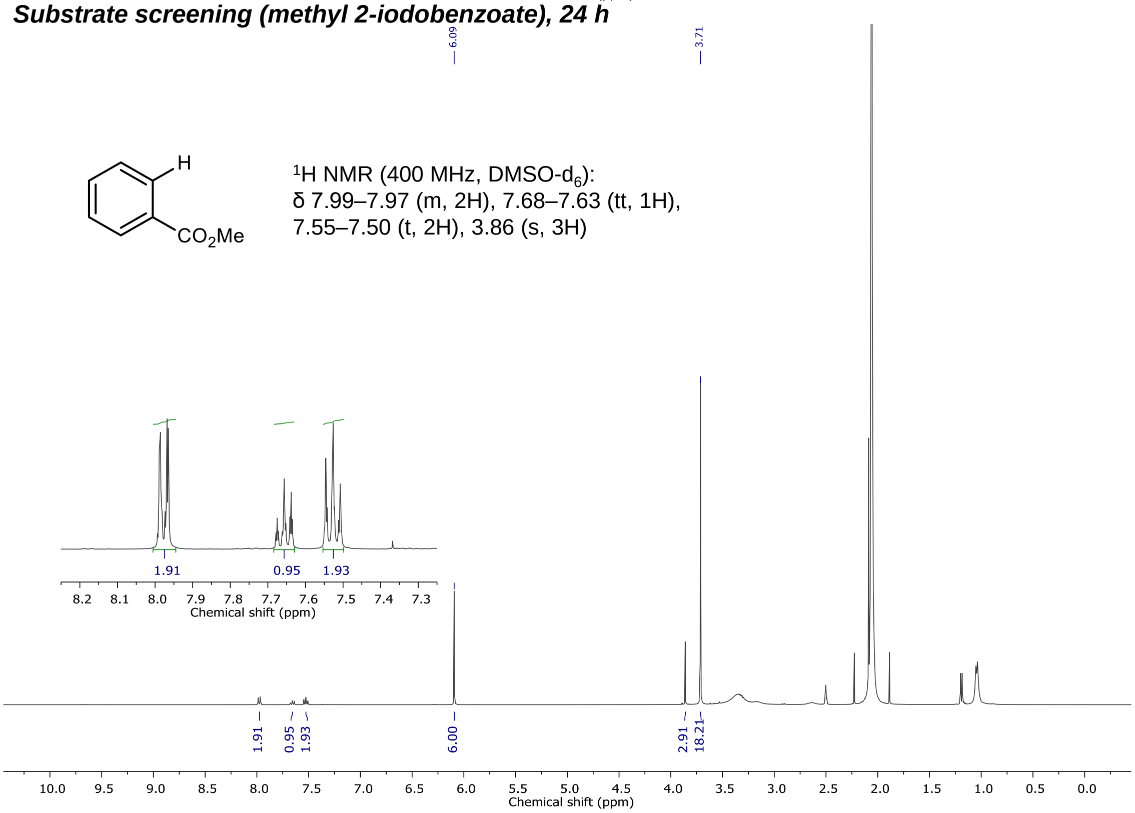

Supplementary Figure 72 ^{1}H NMR data of photoredox reductive dehalogenation for methyl 2-iodobenzoate (400 MHz, DMSO-d_6); 1,3,5-trimethoxybenzene: δ 6.09 (s, 3H), 3.71 (s, 9H).

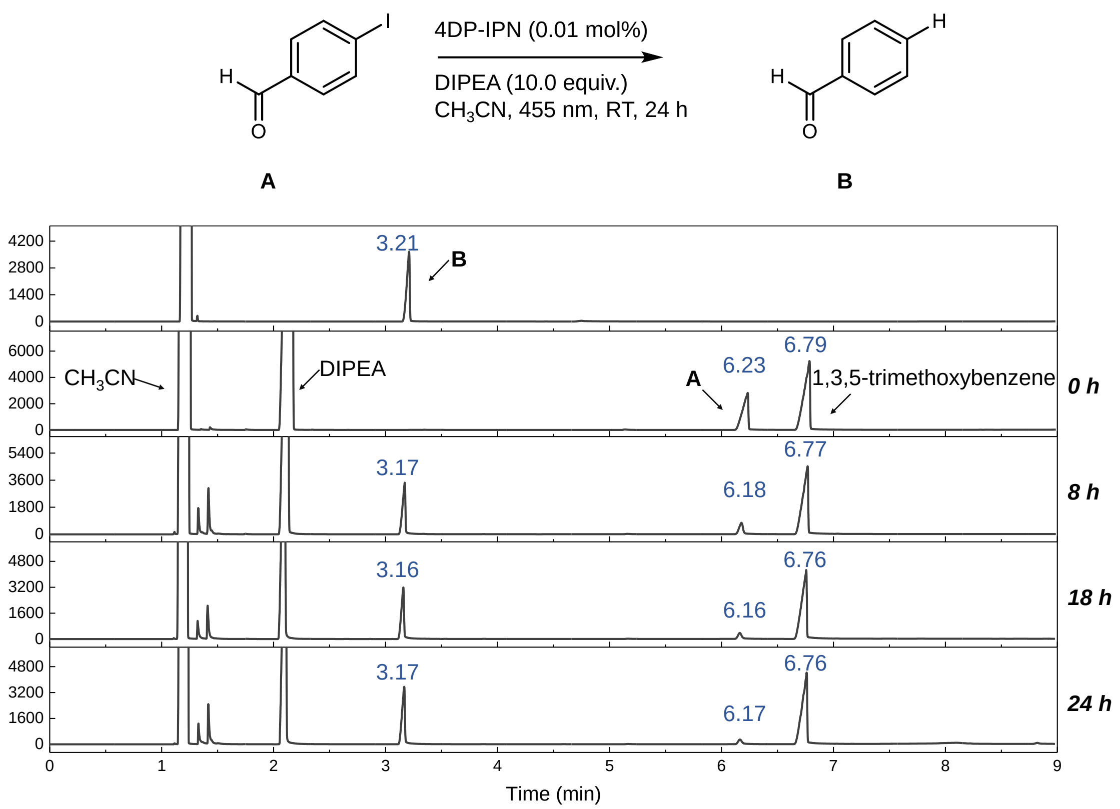

Supplementary Figure 73 GC-FID spectra of photoredox reductive dehalogenation for 4-iodobenzaldehyde. Yield was measured by GC-FID using 1,3,5-trimethoxybenzene as an internal standard.

Substrate screening (4-iodobenzaldehyde), 0 h

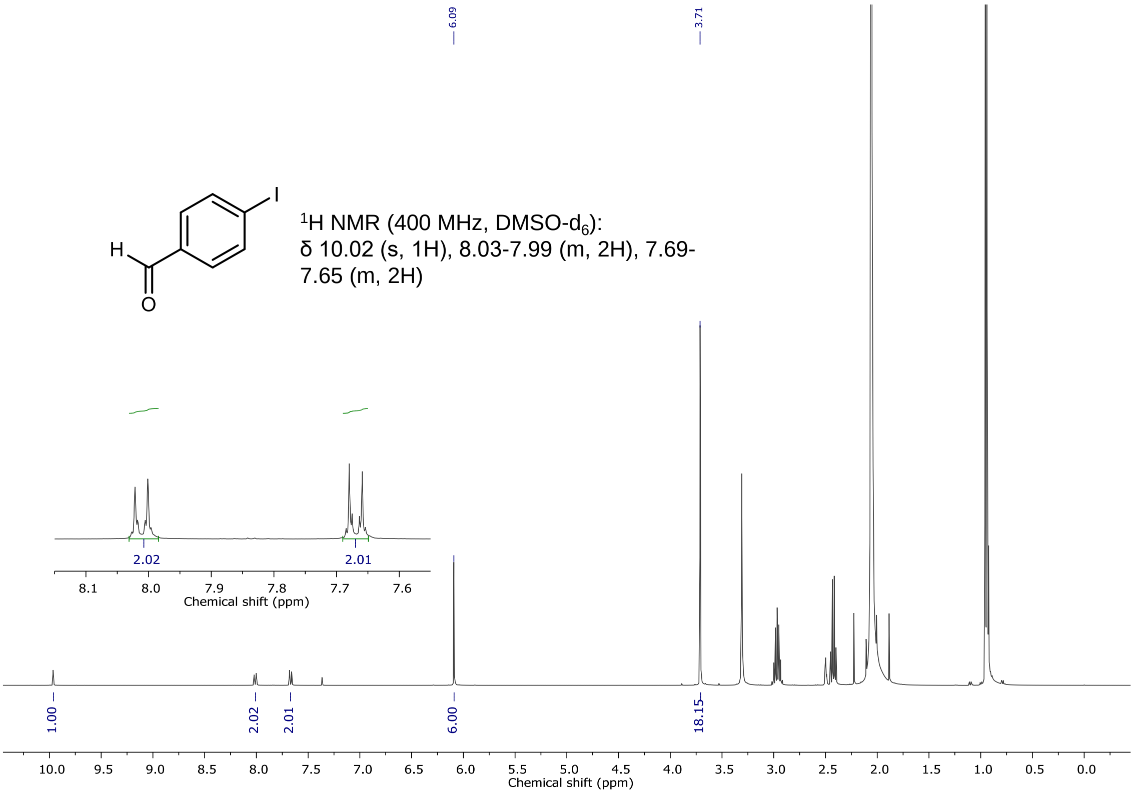

Substrate screening (4-iodobenzaldehyde), 24 h

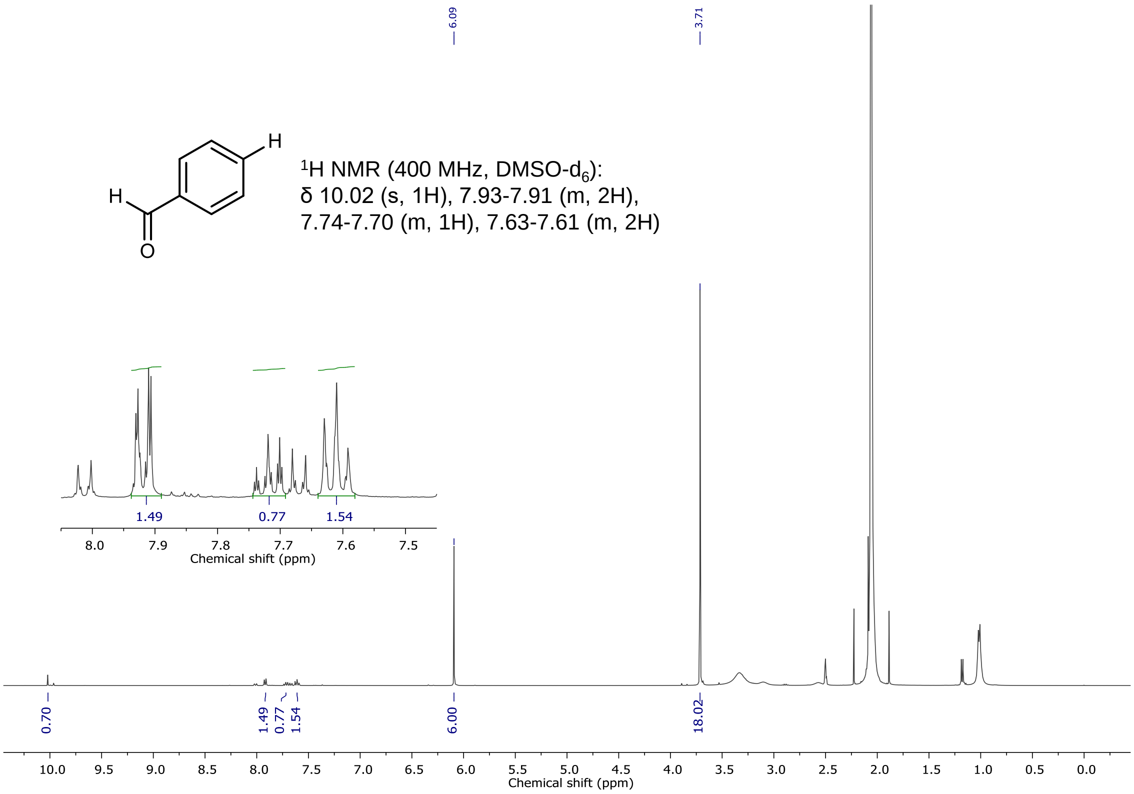

Supplementary Figure 74 ^{1}H NMR data of photoredox reductive dehalogenation for 4-iodobenzaldehyde (400 MHz, DMSO-d_6); 1,3,5-trimethoxybenzene: δ 6.09 (s, 3H), 3.71 (s, 9H).

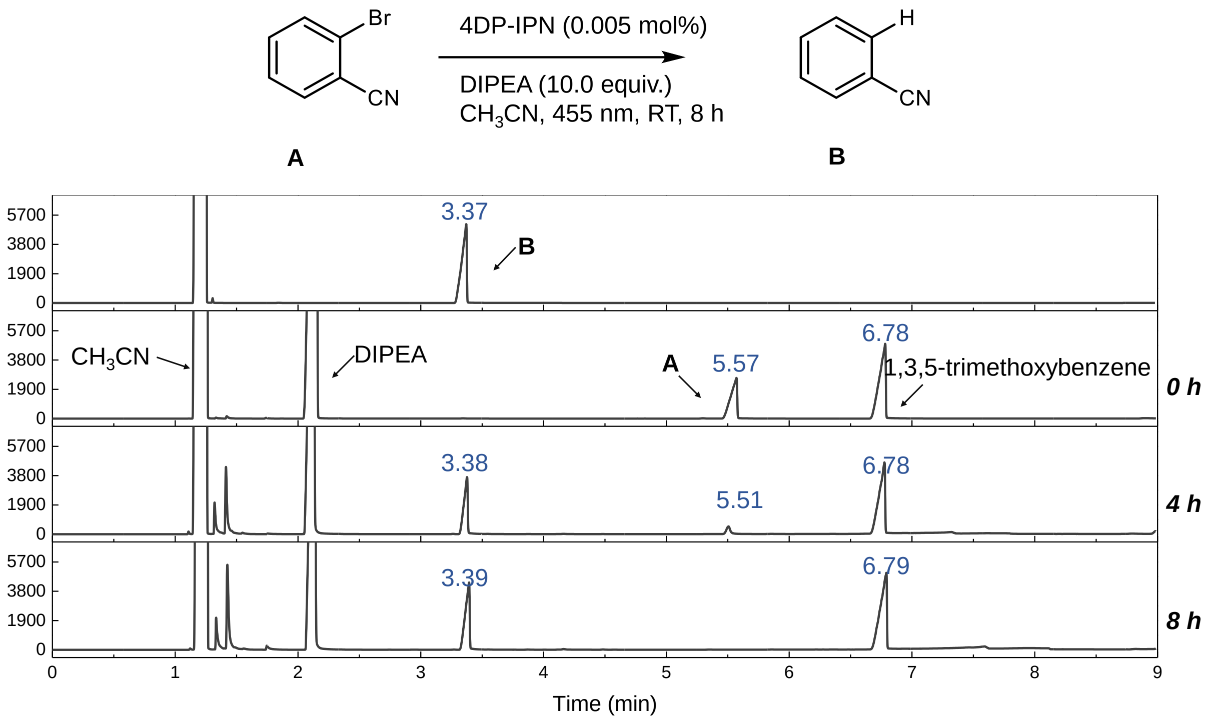

Supplementary Figure 75 GC-FID spectra of photoredox reductive dehalogenation for 2-bromobenzonitrile. Yield was measured by GC-FID using 1,3,5-trimethoxybenzene as an internal standard.

Substrate screening (2-bromobenzonitrile), 0 h

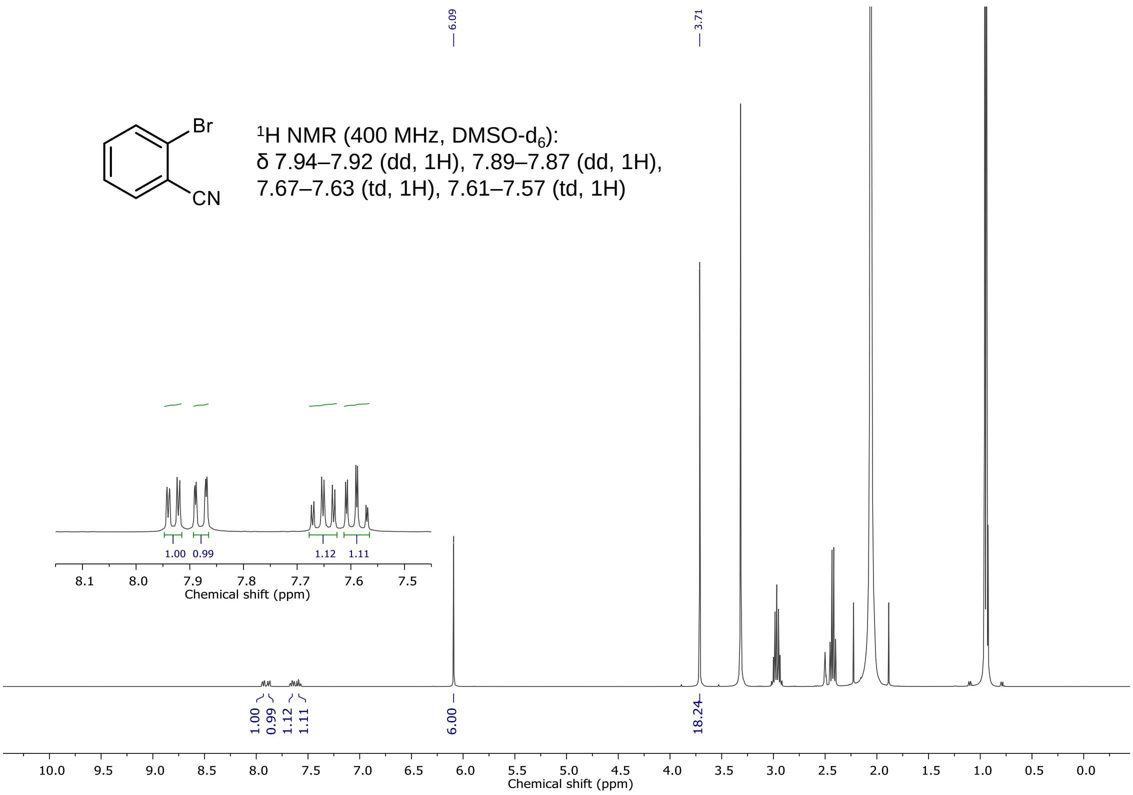

Substrate screening (2-bromobenzonitrile), 8 h

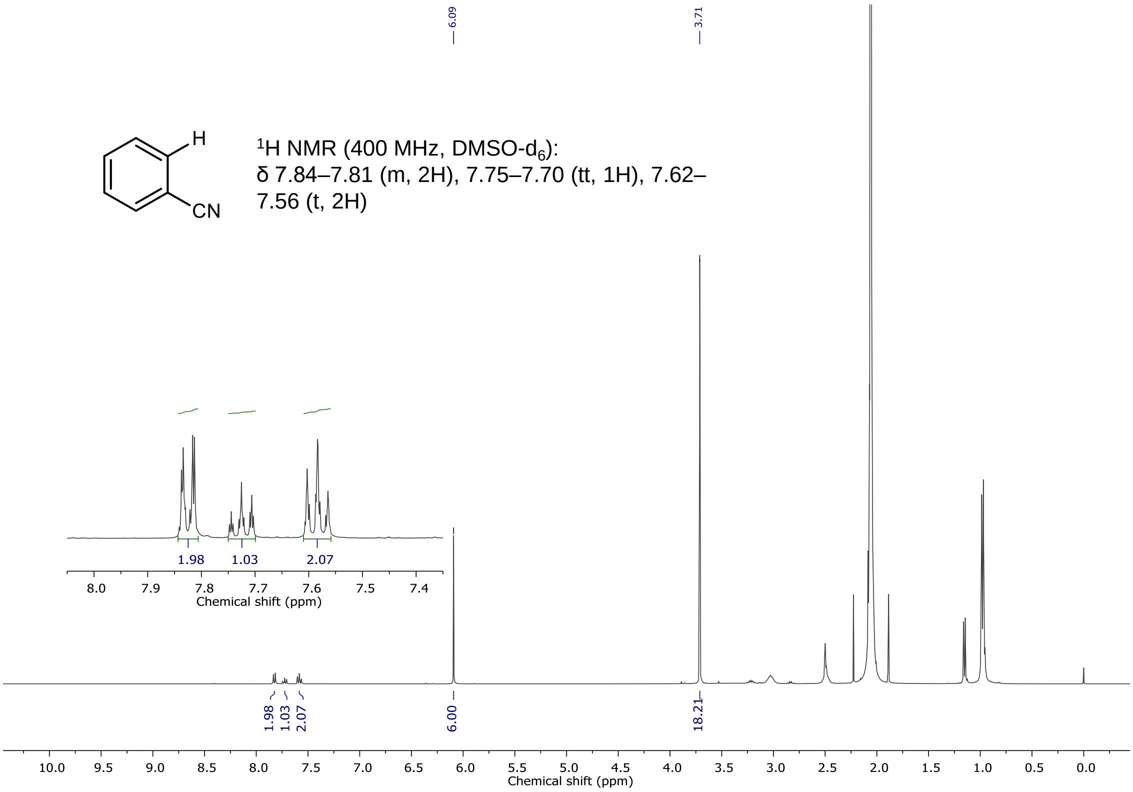

Supplementary Figure 76 ^{1}H NMR data of photoredox reductive dehalogenation for 2-bromobenzonitrile (400 MHz, DMSO-d_6); 1,3,5-trimethoxybenzene: δ 6.09 (s, 3H), 3.71 (s, 9H).

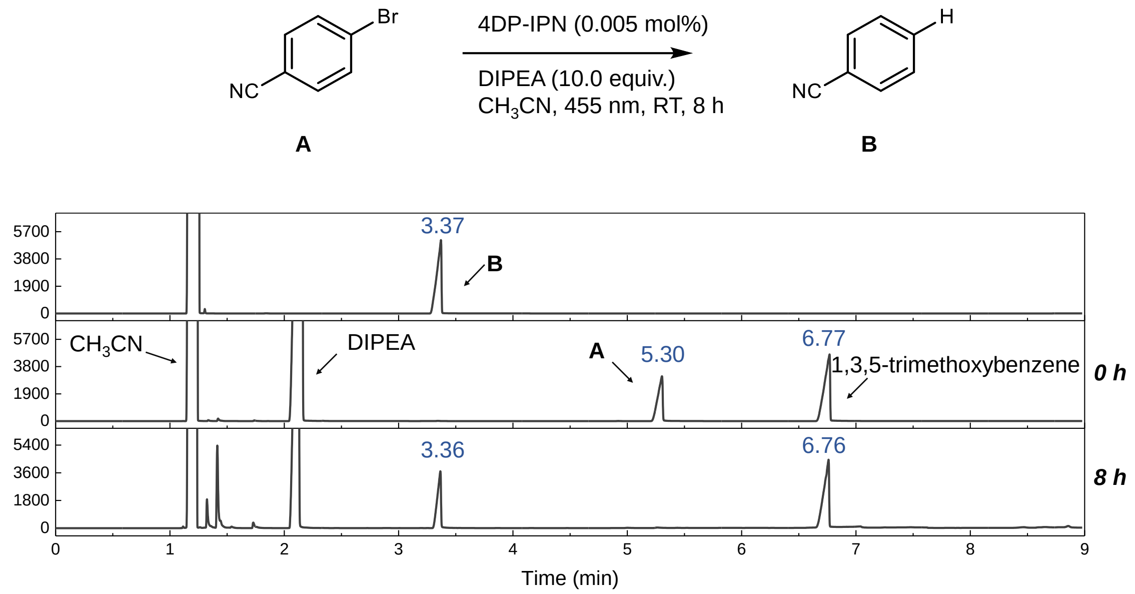

Supplementary Figure 77 GC-FID spectra of photoredox reductive dehalogenation for 4-bromobenzonitrile. Yield was measured by GC-FID using 1,3,5-trimethoxybenzene as an internal standard.

Substrate screening (4-bromobenzonitrile), 0 h

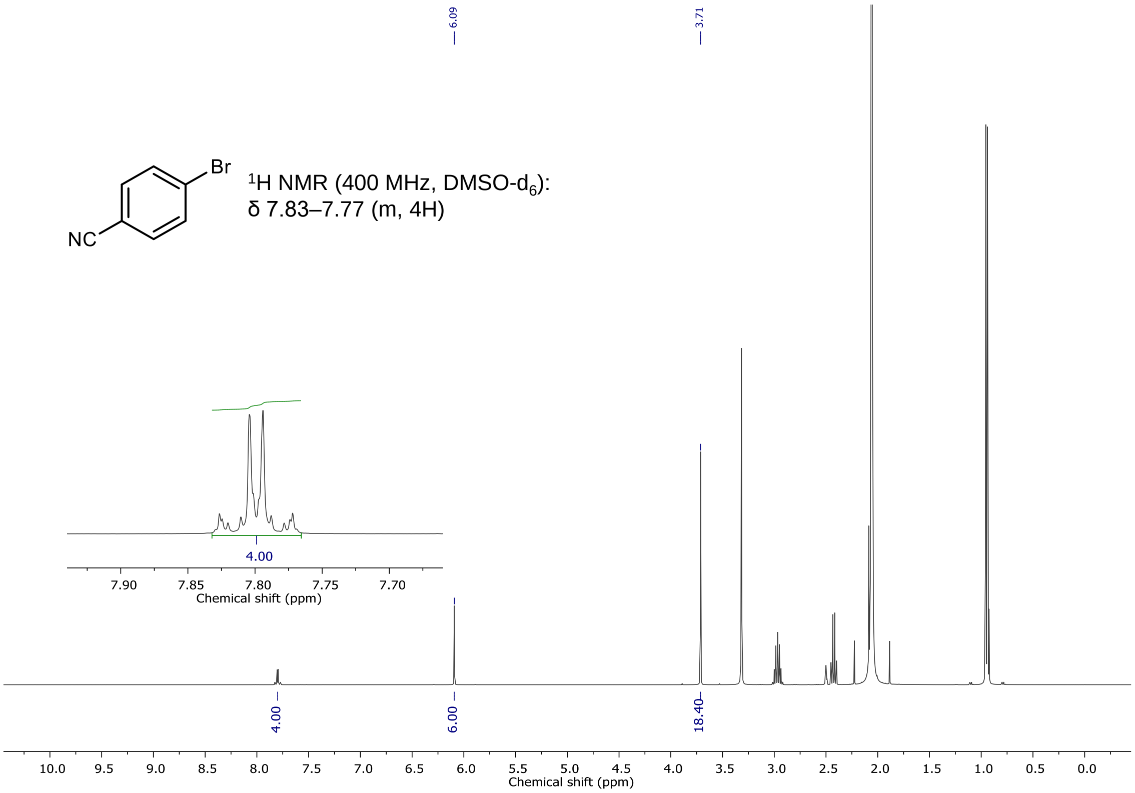

Substrate screening (4-bromobenzonitrile), 8 h

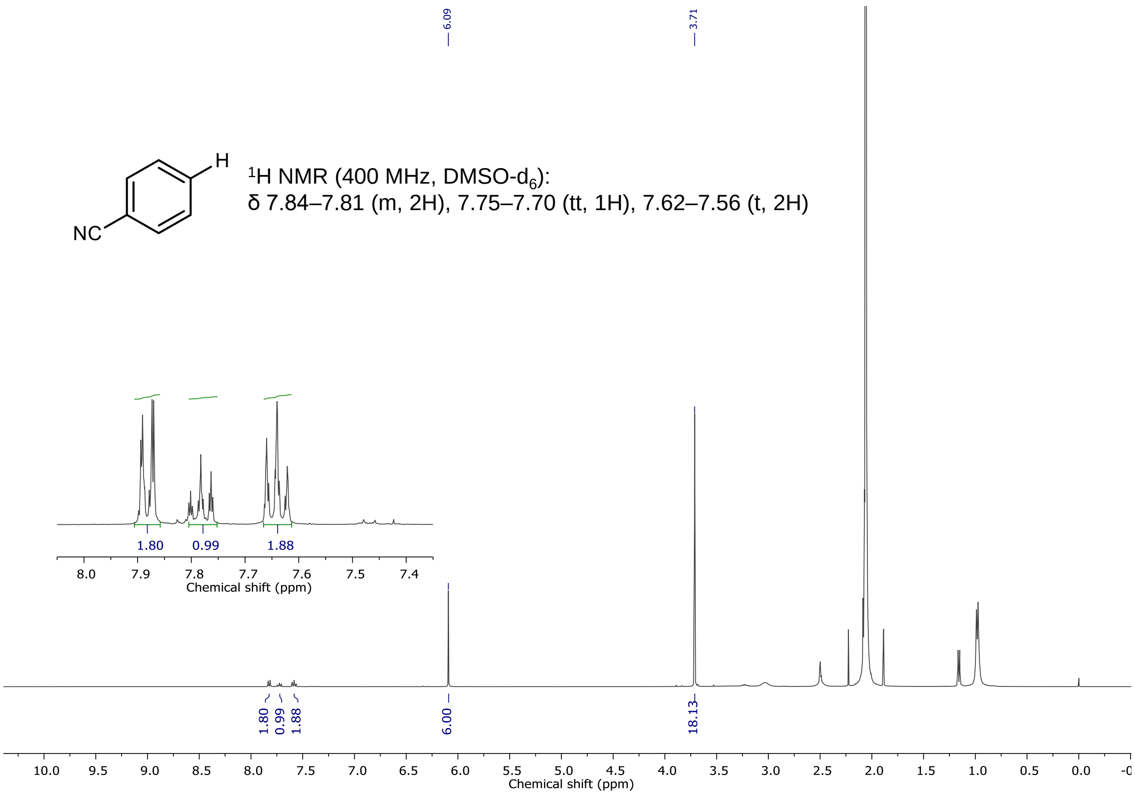

Supplementary Figure 78 ^{1}H NMR data of photoredox reductive dehalogenation for 4-bromobenzonitrile (400 MHz, DMSO-d_6); 1,3,5-trimethoxybenzene: δ 6.09 (s, 3H), 3.71 (s, 9H).

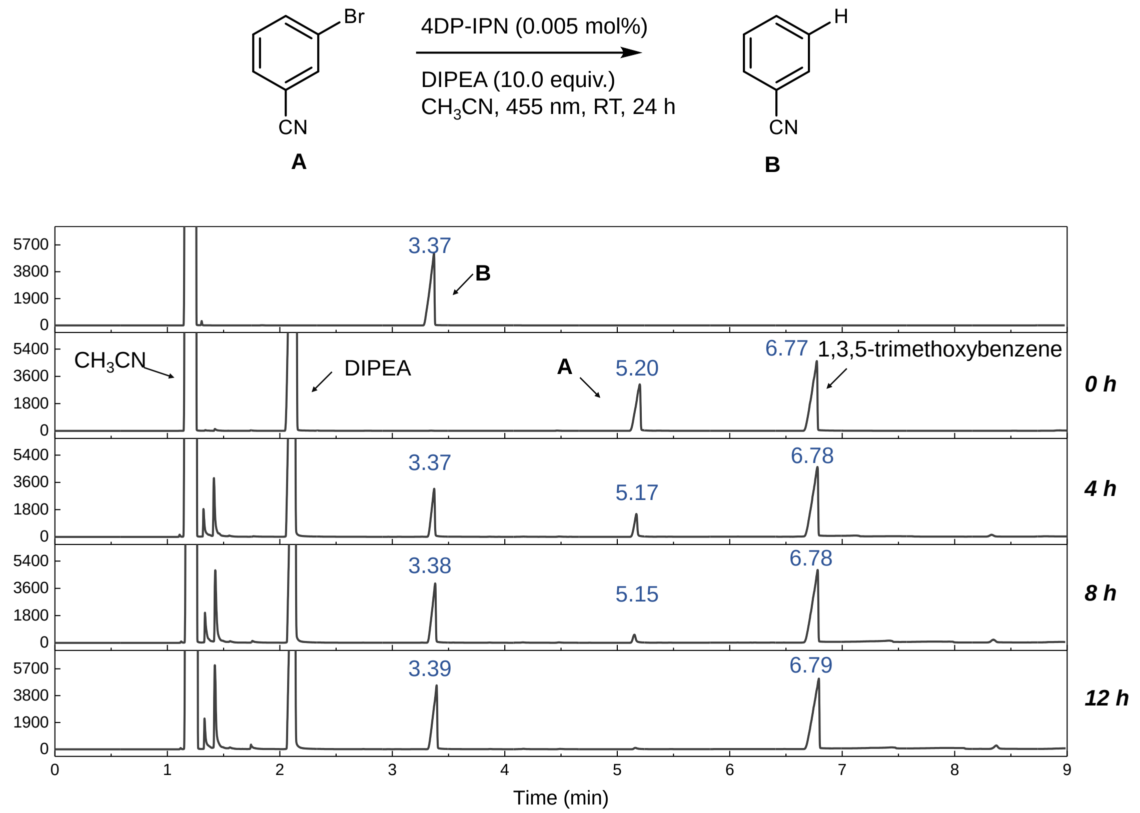

Supplementary Figure 79 GC-FID spectra of photoredox reductive dehalogenation for 3-bromobenzonitrile. Yield was measured by GC-FID using 1,3,5-trimethoxybenzene as an internal standard.

Substrate screening (3-bromobenzonitrile), 0 h

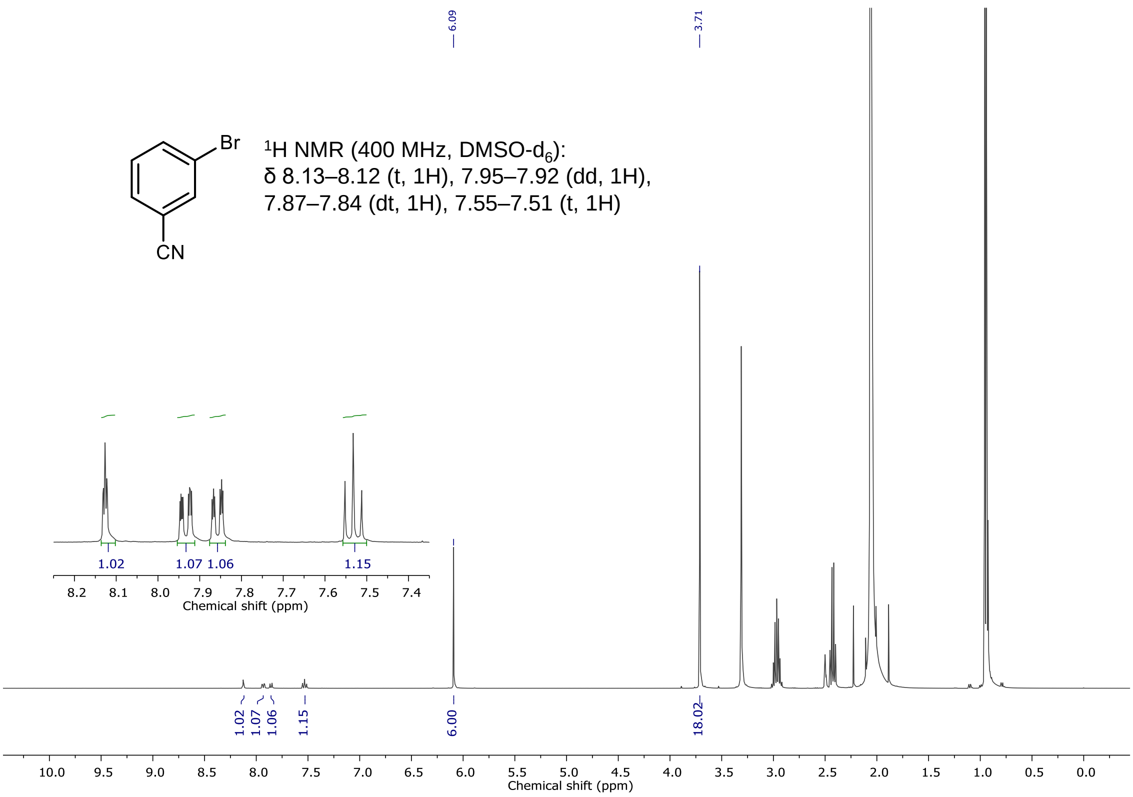

Substrate screening (3-bromobenzonitrile), 12 h

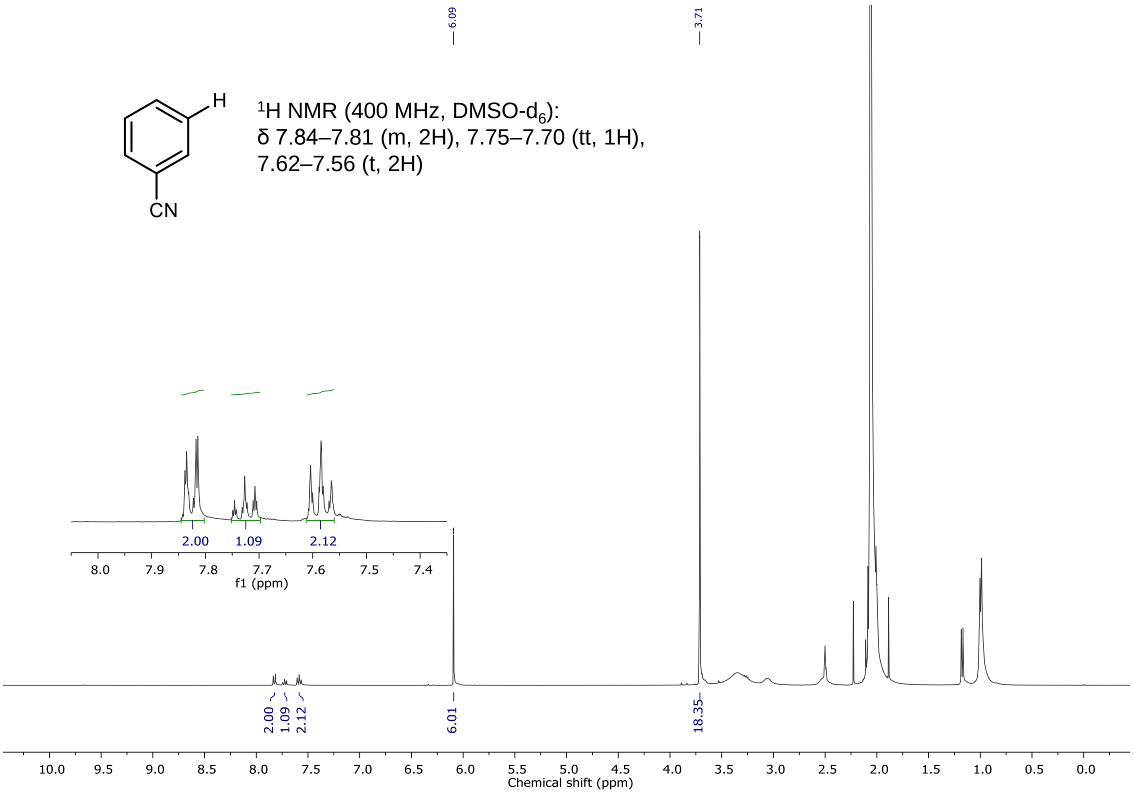

Supplementary Figure 80 ^{1}H NMR data of photoredox reductive dehalogenation for 3-bromobenzonitrile (400 MHz, DMSO-d_6); 1,3,5-trimethoxybenzene: δ 6.09 (s, 3H), 3.71 (s, 9H).

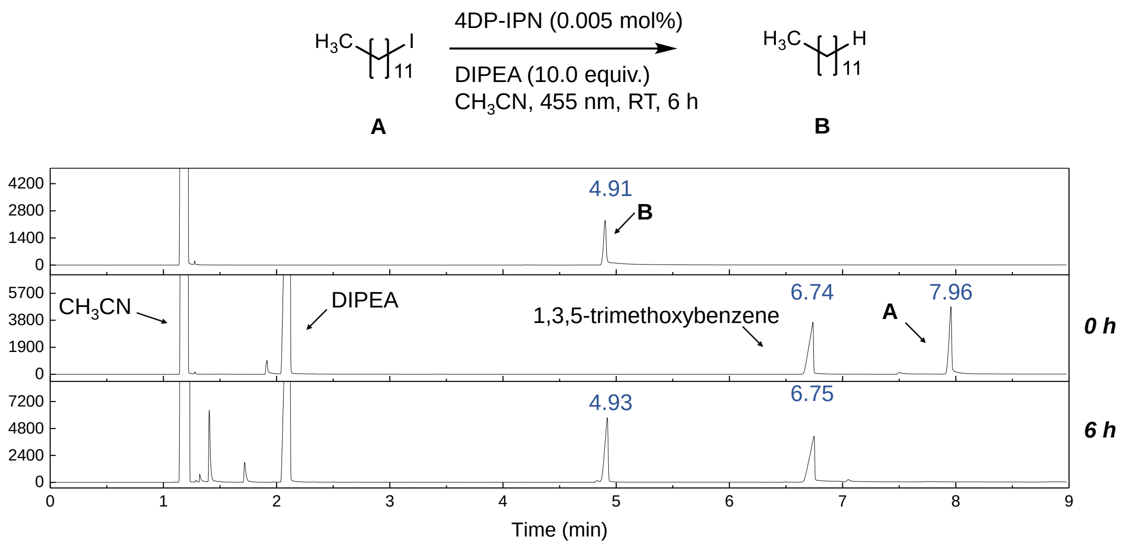

Supplementary Figure 81 GC-FID spectra of photoredox reductive dehalogenation for 1-iodododecane. Yield was measured by GC-FID using 1,3,5-trimethoxybenzene as an internal standard.

Substrate screening (1-iododecane), 0 h

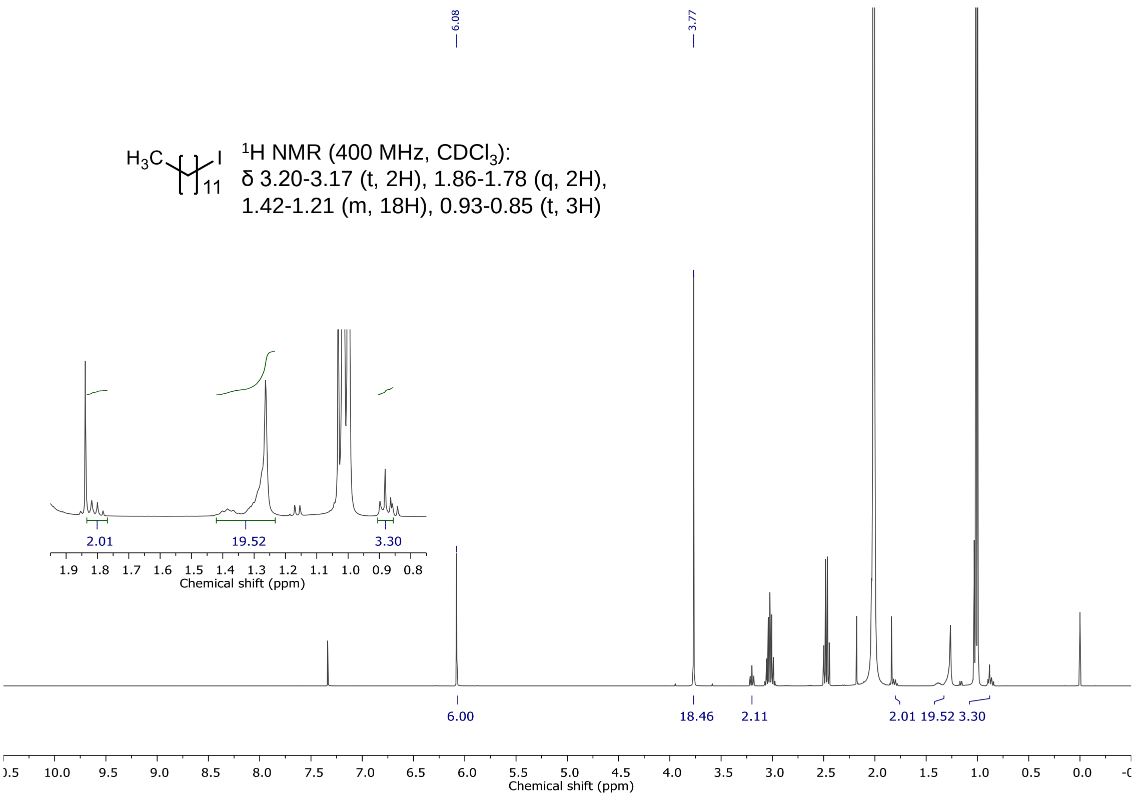

Substrate screening (1-iododecane), 6 h

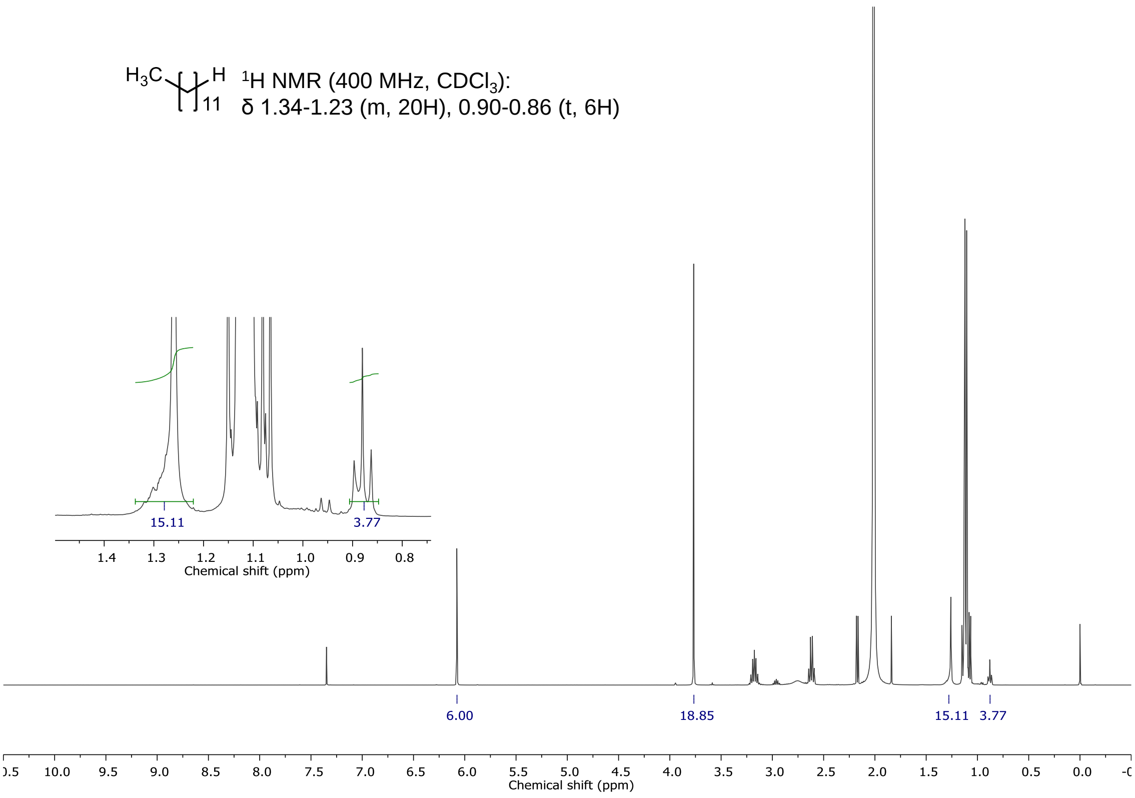

Supplementary Figure 82 ^{1}H NMR data of photoredox reductive dehalogenation for 1-iodododecane (400 MHz, $CDCl_3$); 1,3,5-trimethoxybenzene: δ 6.09 (s, 3H), 3.71 (s, 9H).

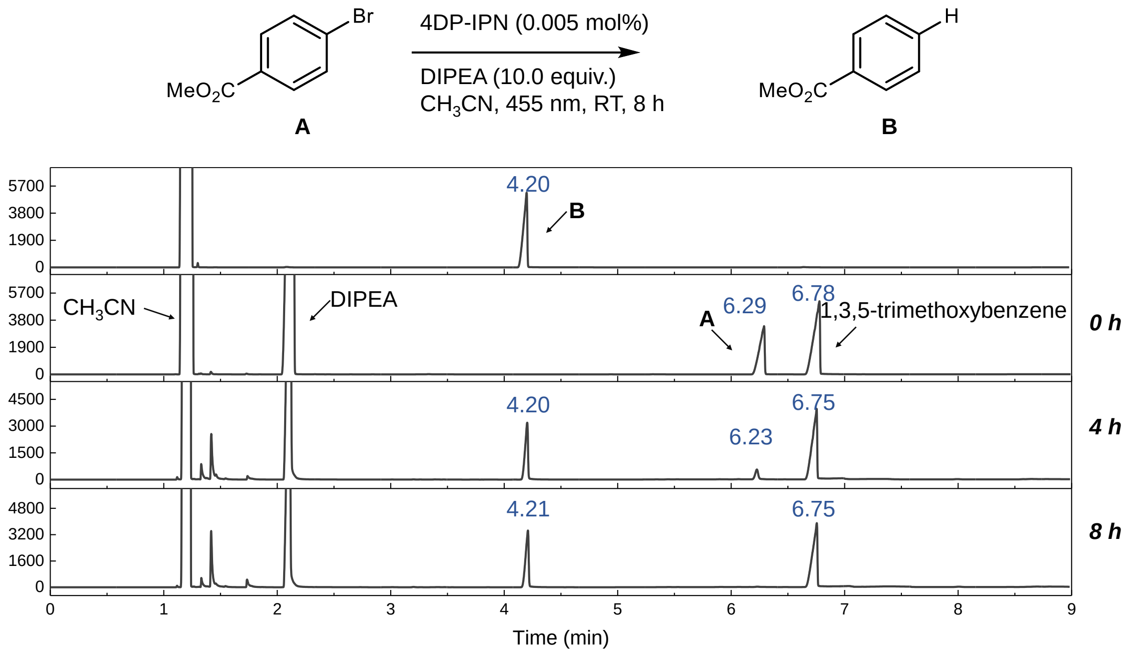

Supplementary Figure 83 GC-FID spectra of photoredox reductive dehalogenation for methyl 4-bromobenzoate. Yield was measured by GC-FID using 1,3,5-trimethoxybenzene as an internal standard.

Substrate screening (methyl 4-bromobenzoate), 0 h

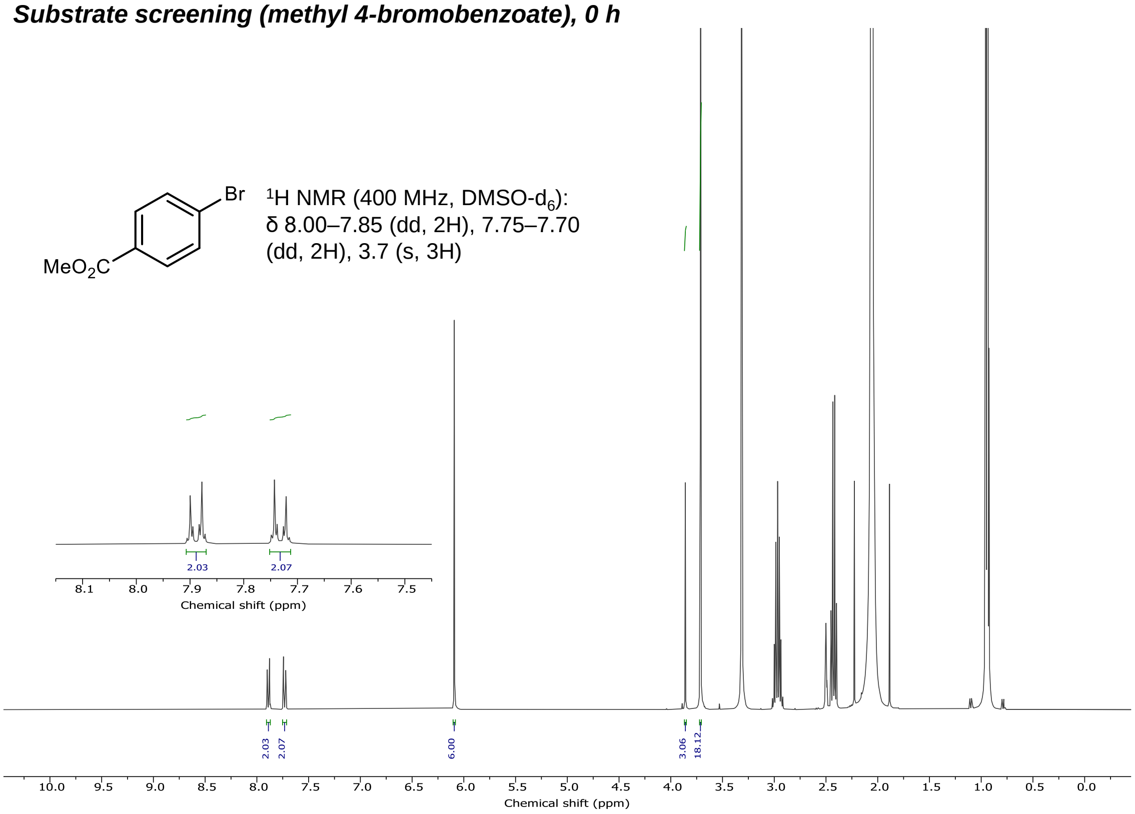

Substrate screening (methyl 4-bromobenzoate), 8 h

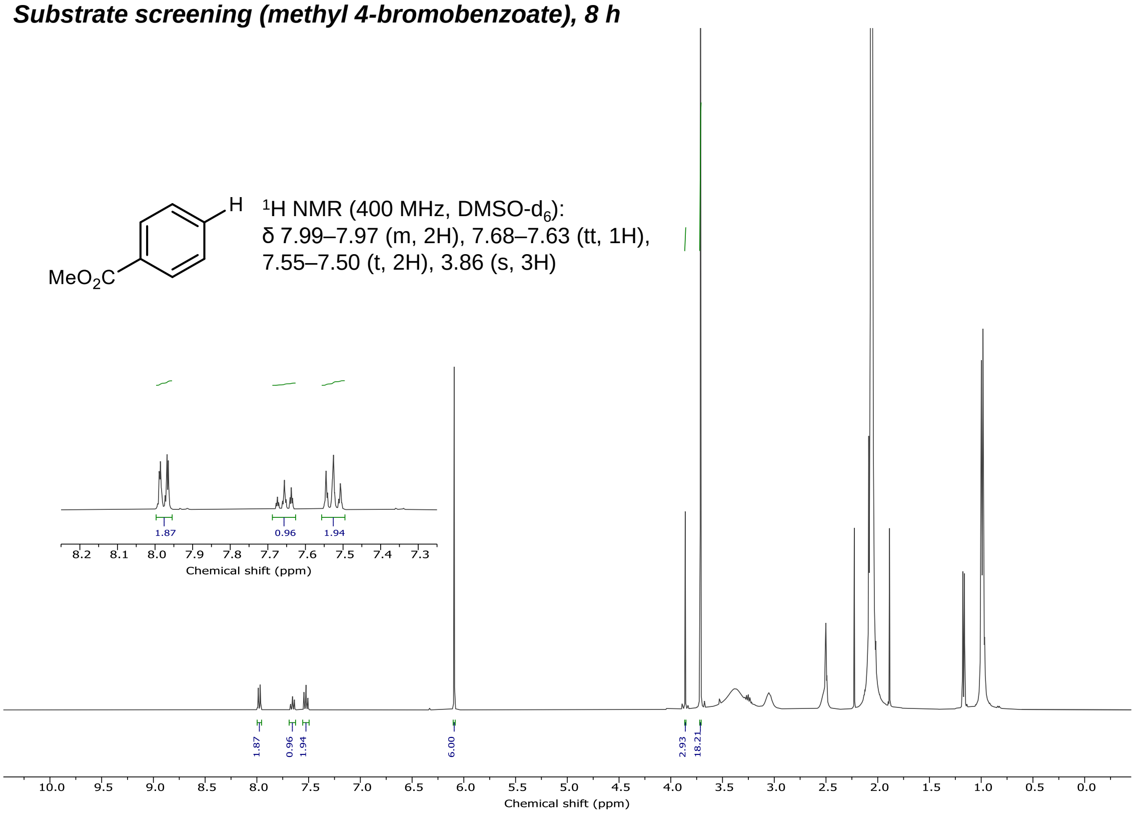

Supplementary Figure 84 ^{1}H NMR data of photoredox reductive dehalogenation for methyl 4-bromobenzoate (400 MHz, DMSO-d_6); 1,3,5-trimethoxybenzene: δ 6.09 (s, 3H), 3.71 (s, 9H).

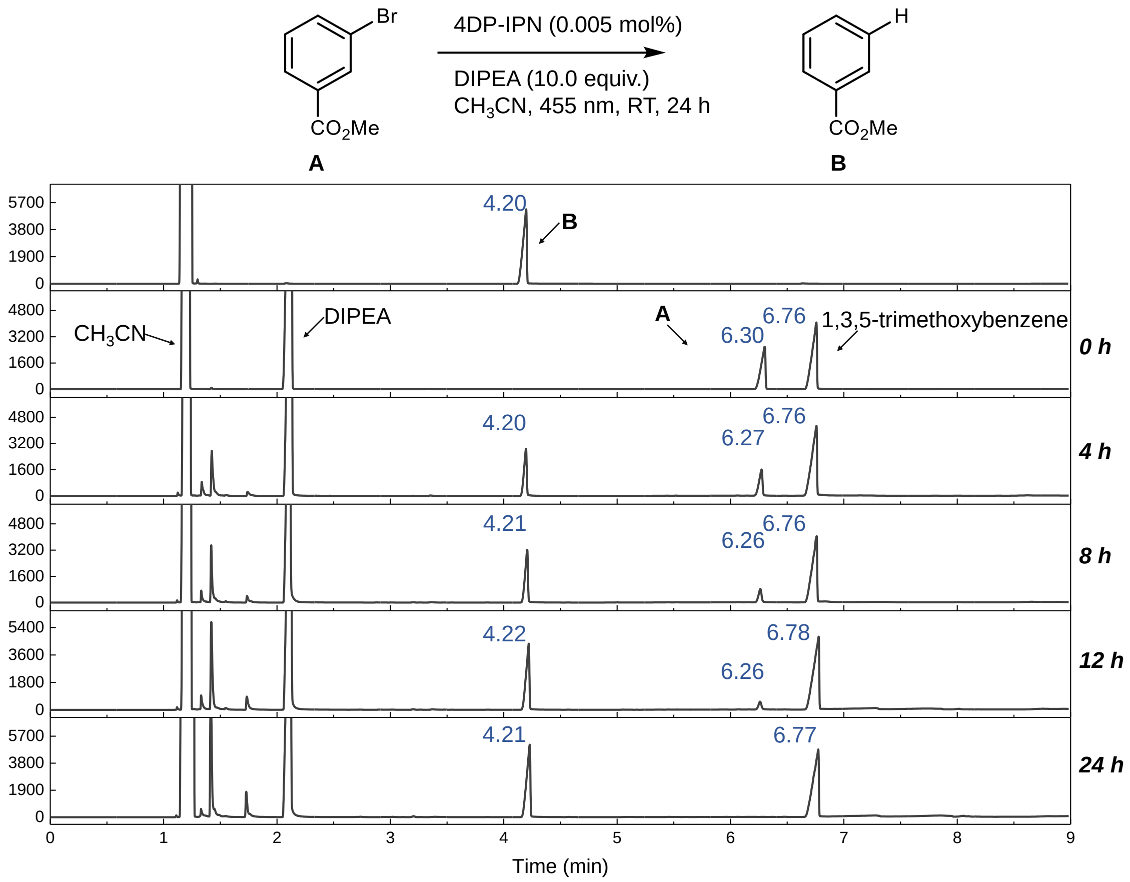

Supplementary Figure 85 GC-FID spectra of photoredox reductive dehalogenation for methyl 3-bromobenzoate. Yield was measured by GC-FID using 1,3,5-trimethoxybenzene as an internal standard.

Substrate screening (methyl 3-bromobenzoate), 0 h

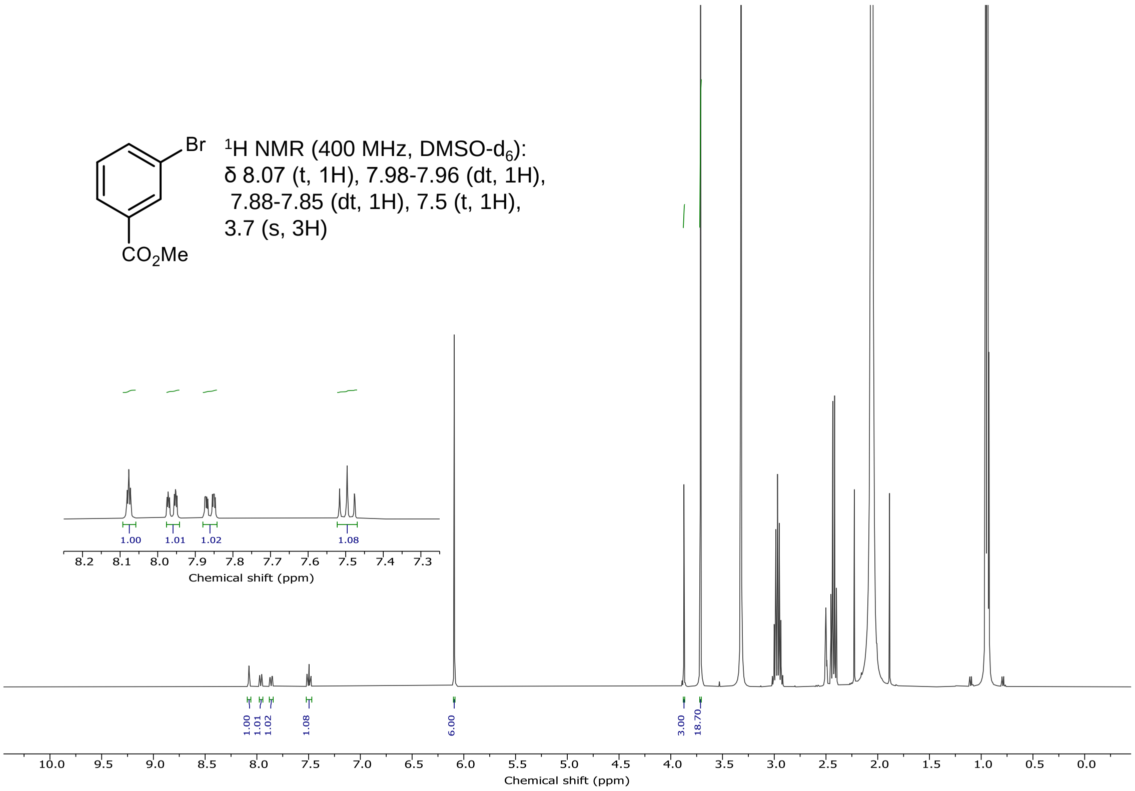

Substrate screening (methyl 3-bromobenzoate), 24 h

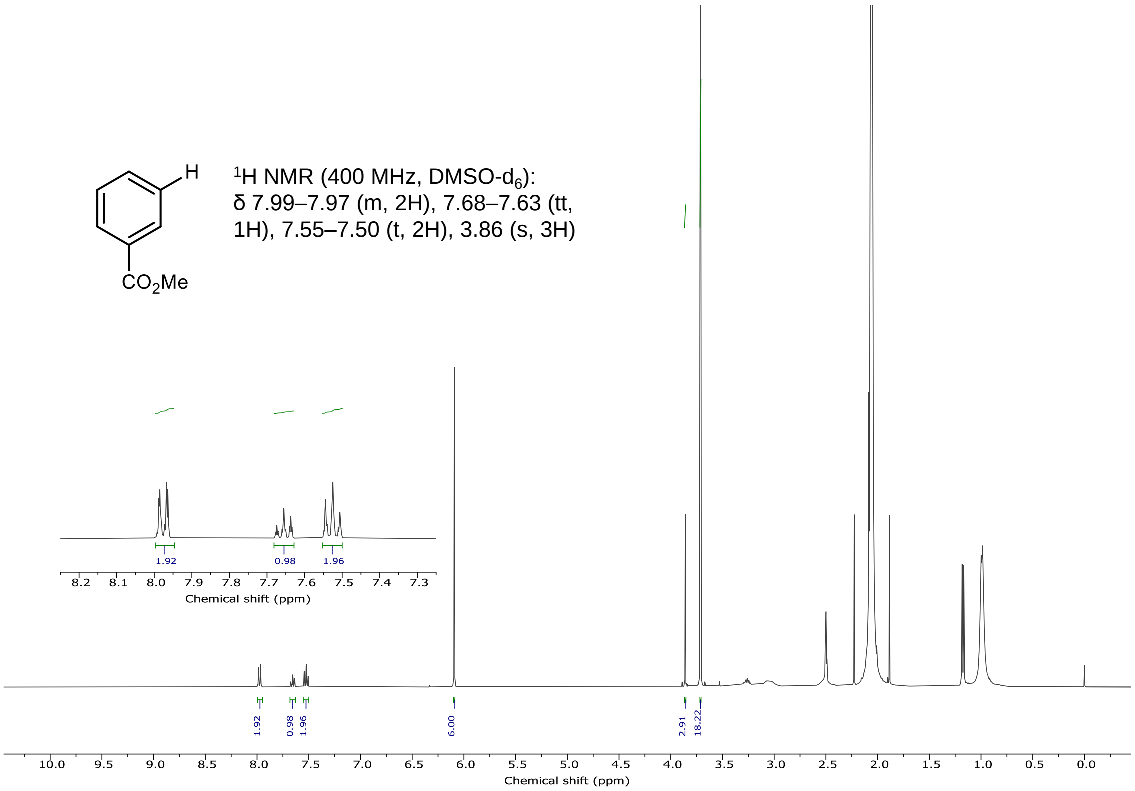

Supplementary Figure 86 ^{1}H NMR data of photoredox reductive dehalogenation for methyl 3-bromobenzoate (400 MHz, DMSO-d_6); 1,3,5-trimethoxybenzene: δ 6.09 (s, 3H), 3.71 (s, 9H).

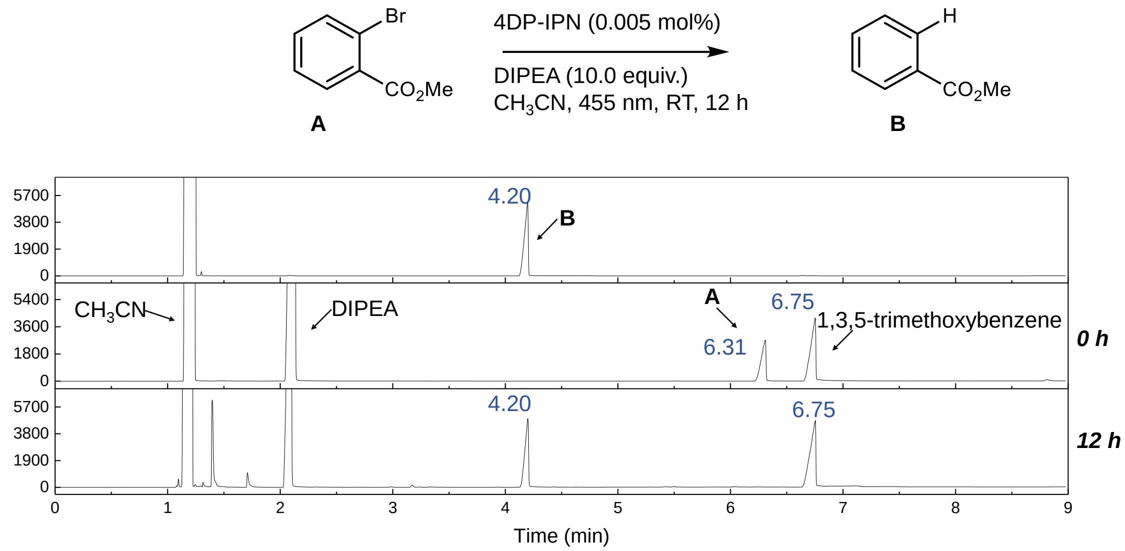

Supplementary Figure 87 GC-FID spectra of photoredox reductive dehalogenation for methyl 2-bromobenzoate. Yield was measured by GC-FID using 1,3,5-trimethoxybenzene as an internal standard.

Substrate screening (methyl 2-bromobenzoate), 0 h

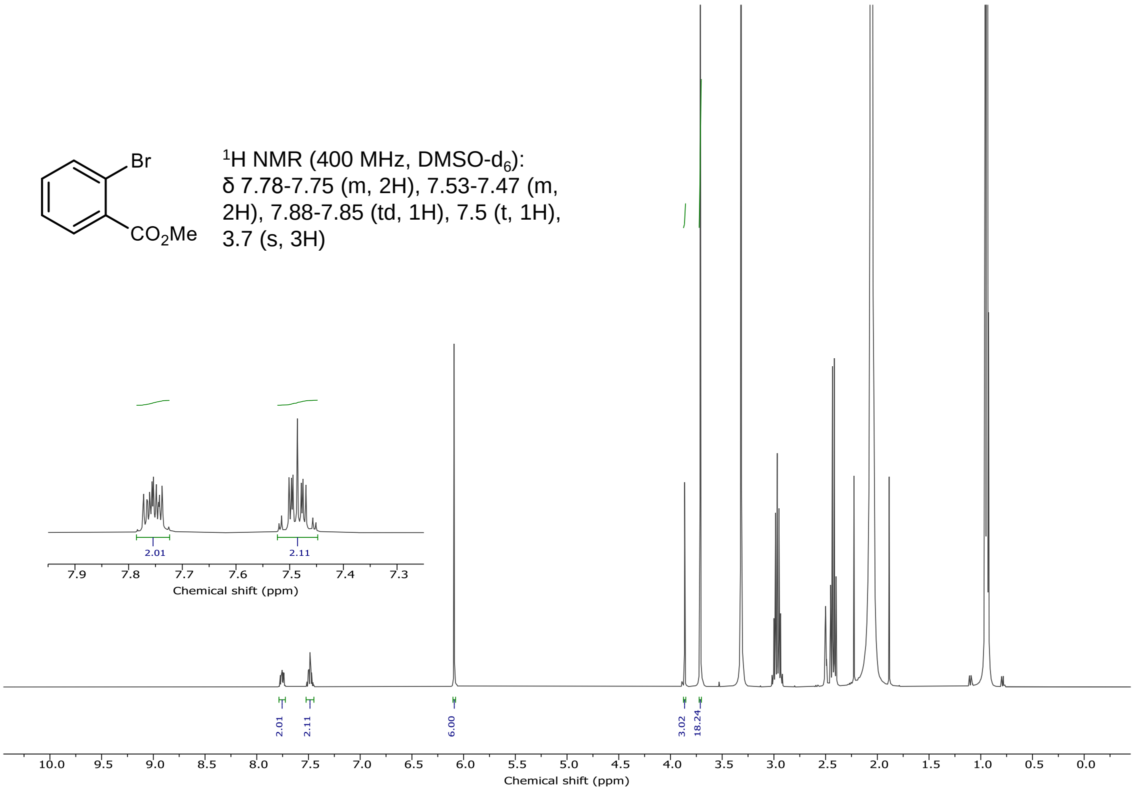

Substrate screening (methyl 2-bromobenzoate), 12 h

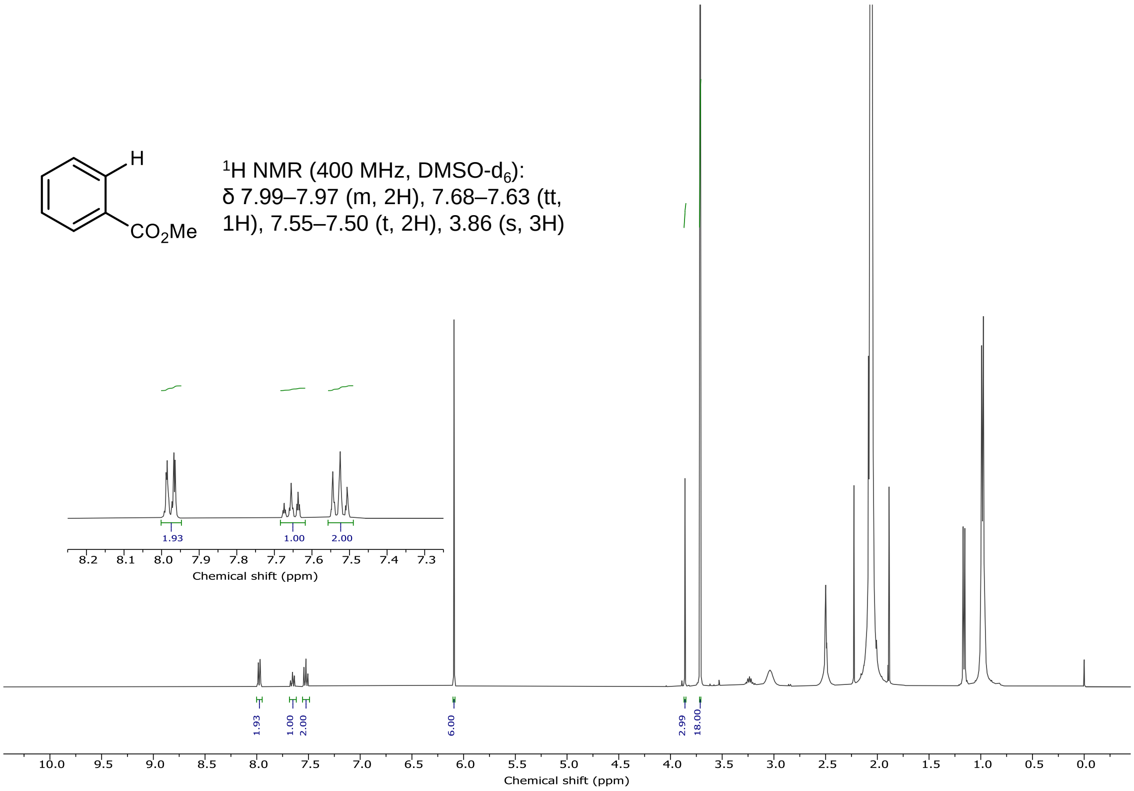

Supplementary Figure 88 ^{1}H NMR data of photoredox reductive dehalogenation for methyl 2-bromobenzoate (400 MHz, DMSO-d_6); 1,3,5-trimethoxybenzene: δ 6.09 (s, 3H), 3.71 (s, 9H).

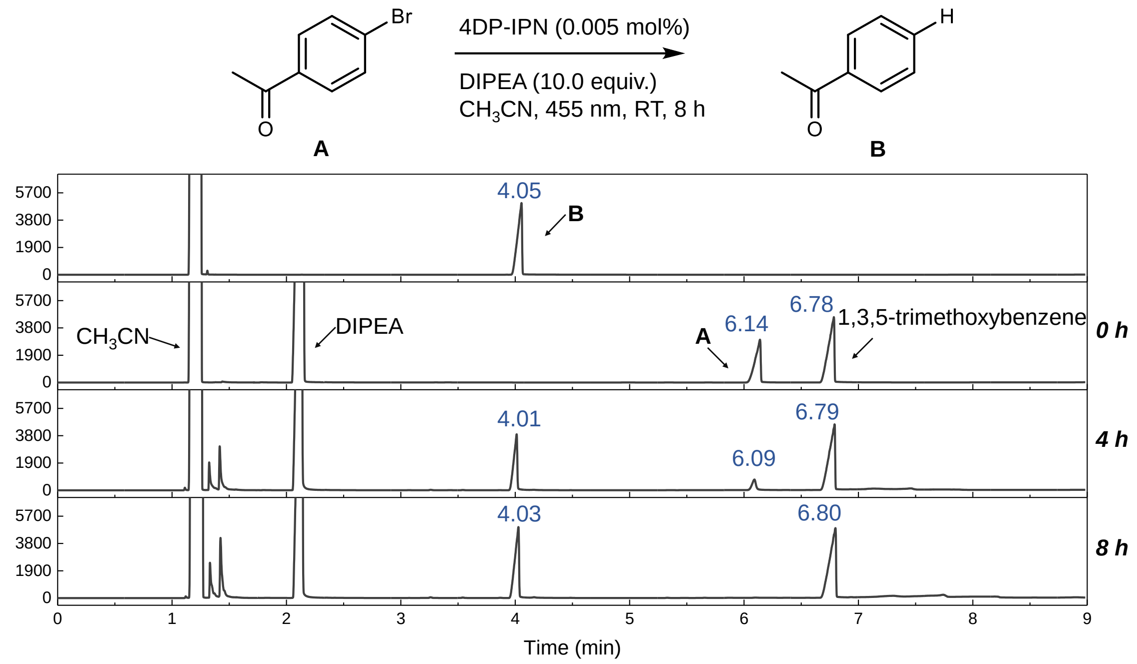

Supplementary Figure 89 GC-FID spectra of photoredox reductive dehalogenation for 4-bromoacetophenone. Yield was measured by GC-FID using 1,3,5-trimethoxybenzene as an internal standard.

Substrate screening (4-bromoacetophenone), 0 h

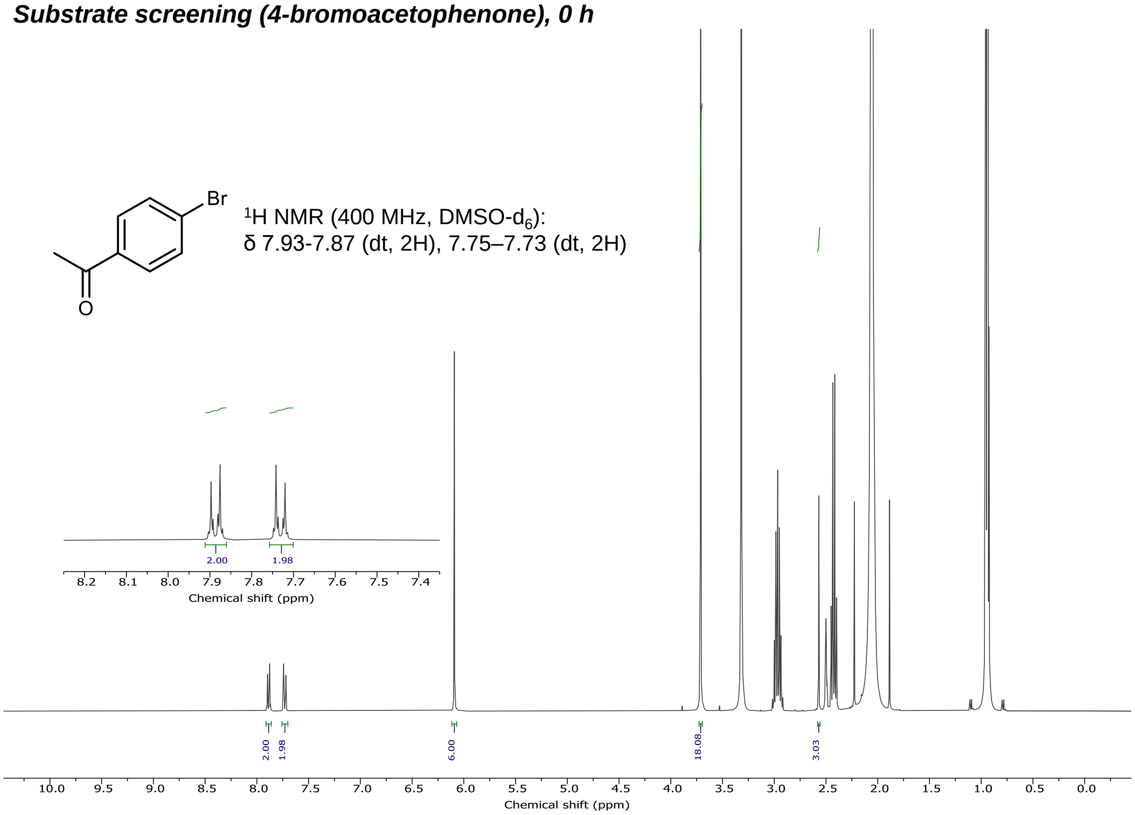

Substrate screening (4-bromoacetophenone), 8 h

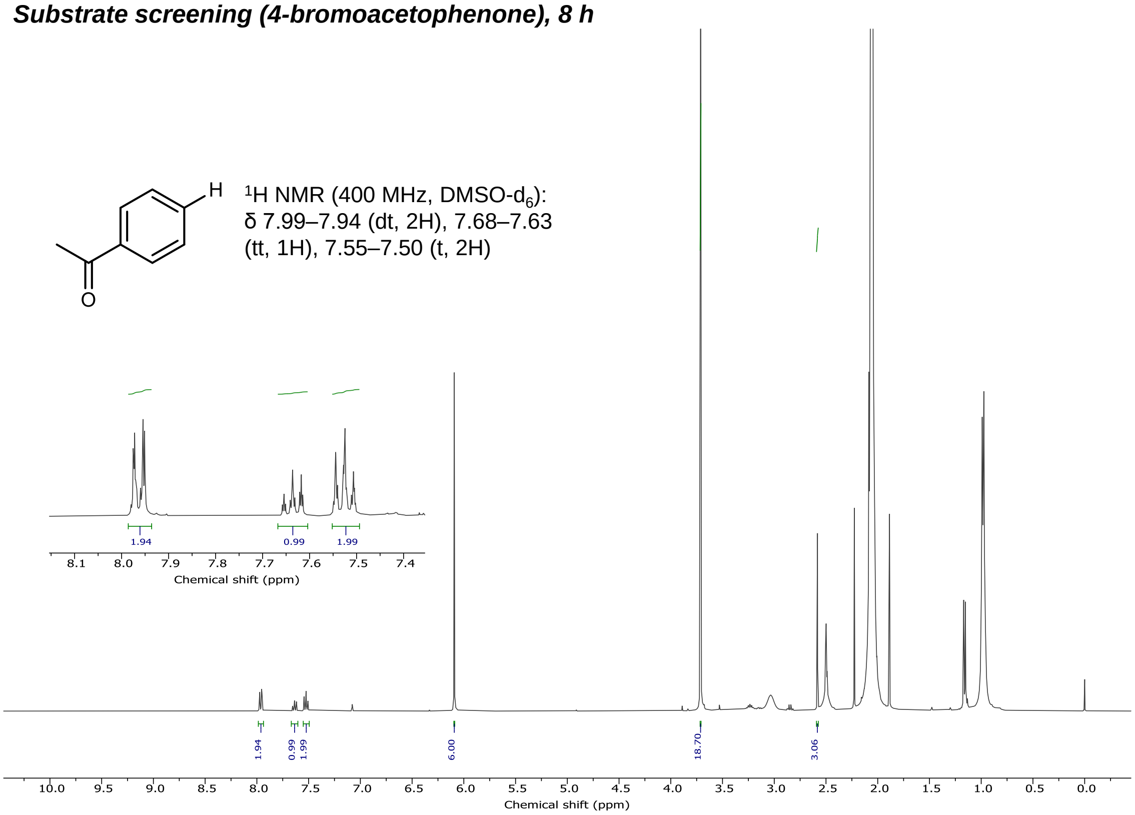

Supplementary Figure 90 ^{1}H NMR data of photoredox reductive dehalogenation for 4-bromoacetophenone (400 MHz, DMSO-d_6); 1,3,5-trimethoxybenzene: δ 6.09 (s, 3H), 3.71 (s, 9H).

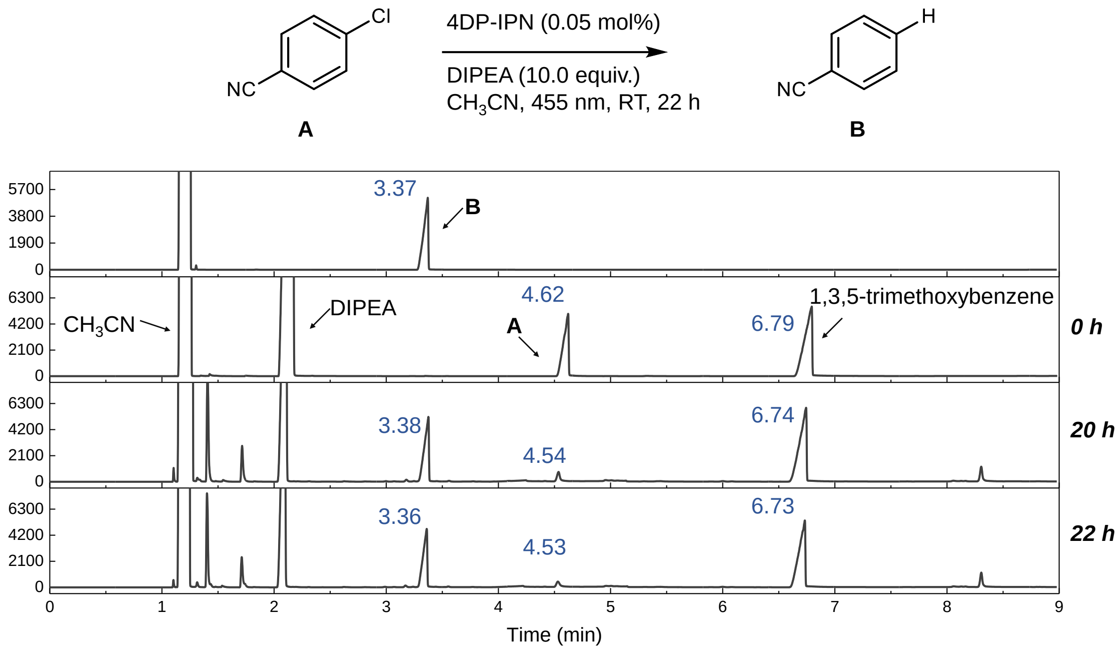

Supplementary Figure 91 GC-FID spectra of photoredox reductive dehalogenation for 4-chlorobenzonitrile. Yield was measured by GC-FID using 1,3,5-trimethoxybenzene as an internal standard.

Substrate screening (4-chlorobenzonitrile), 0 h

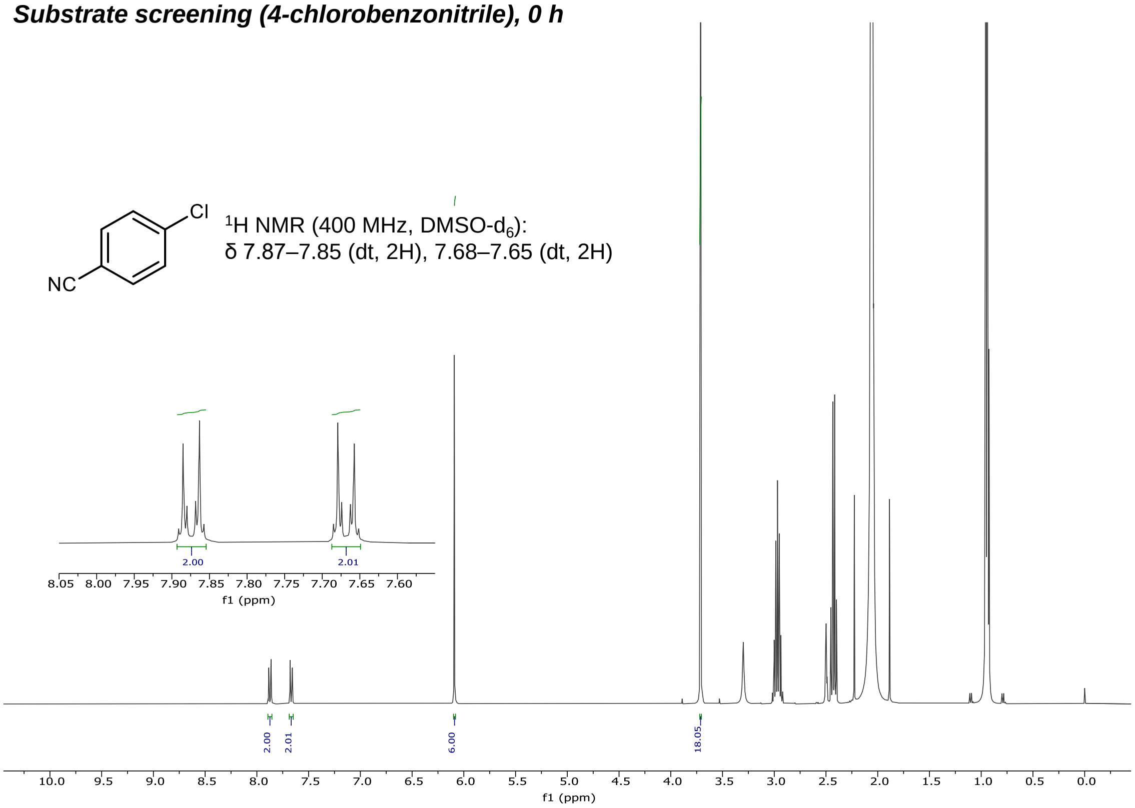

Substrate screening (4-chlorobenzonitrile), 22 h

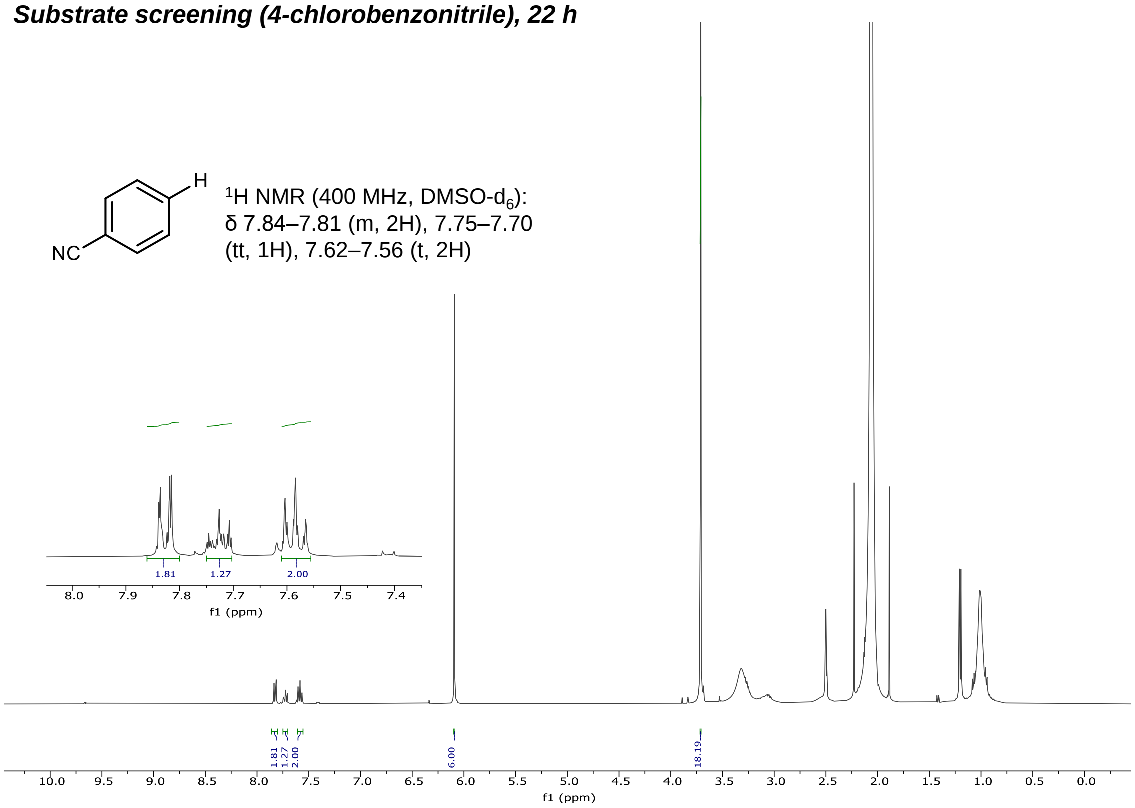

Supplementary Figure 92 ^{1}H NMR data of photoredox reductive dehalogenation for 4-chlorobenzonitrile (400 MHz, DMSO-d_6); 1,3,5-trimethoxybenzene: δ 6.09 (s, 3H), 3.71 (s, 9H).

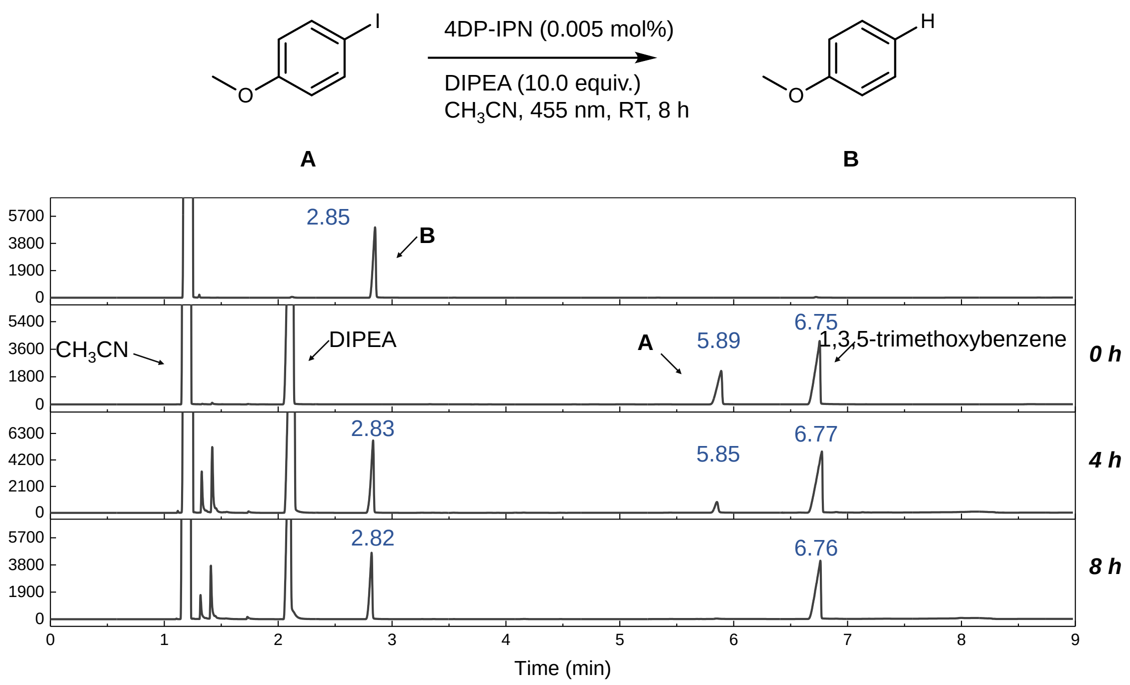

Supplementary Figure 93 GC-FID spectra of photoredox reductive dehalogenation for 4-iodoanisole. Yield was measured by GC-FID using 1,3,5-trimethoxybenzene as an internal standard.

Substrate screening (4-iodoanisole), 0 h

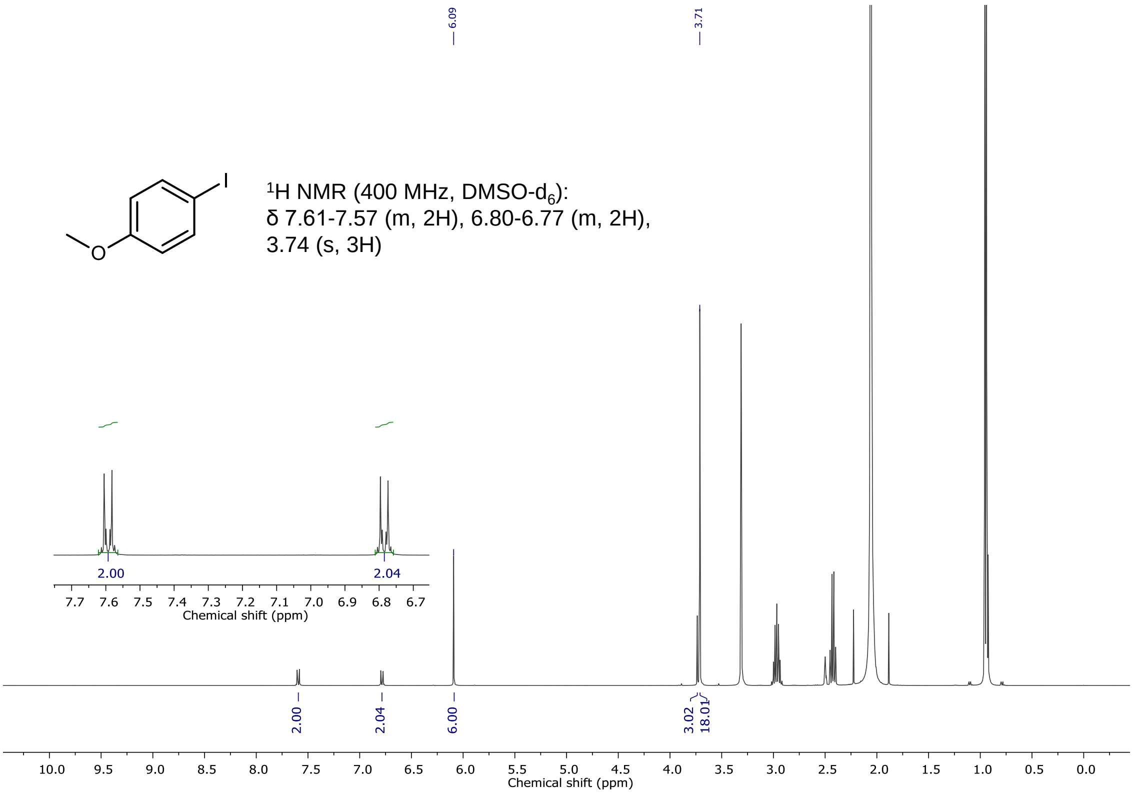

Substrate screening (4-iodoanisole), 8 h

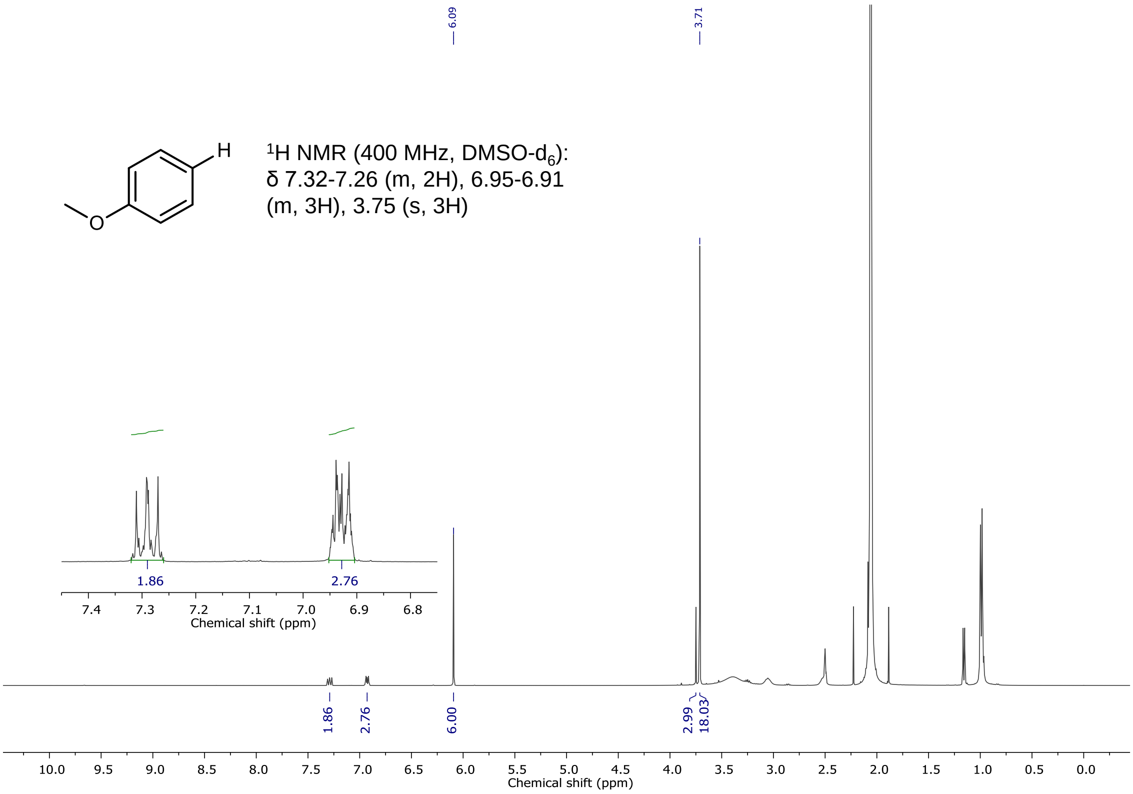

Supplementary Figure 94 ^{1}H NMR data of photoredox reductive dehalogenation for 4-iodoanisole (400 MHz, DMSO-d$_6$); 1,3,5-trimethoxybenzene: δ 6.09 (s, 3H), 3.71 (s, 9H).

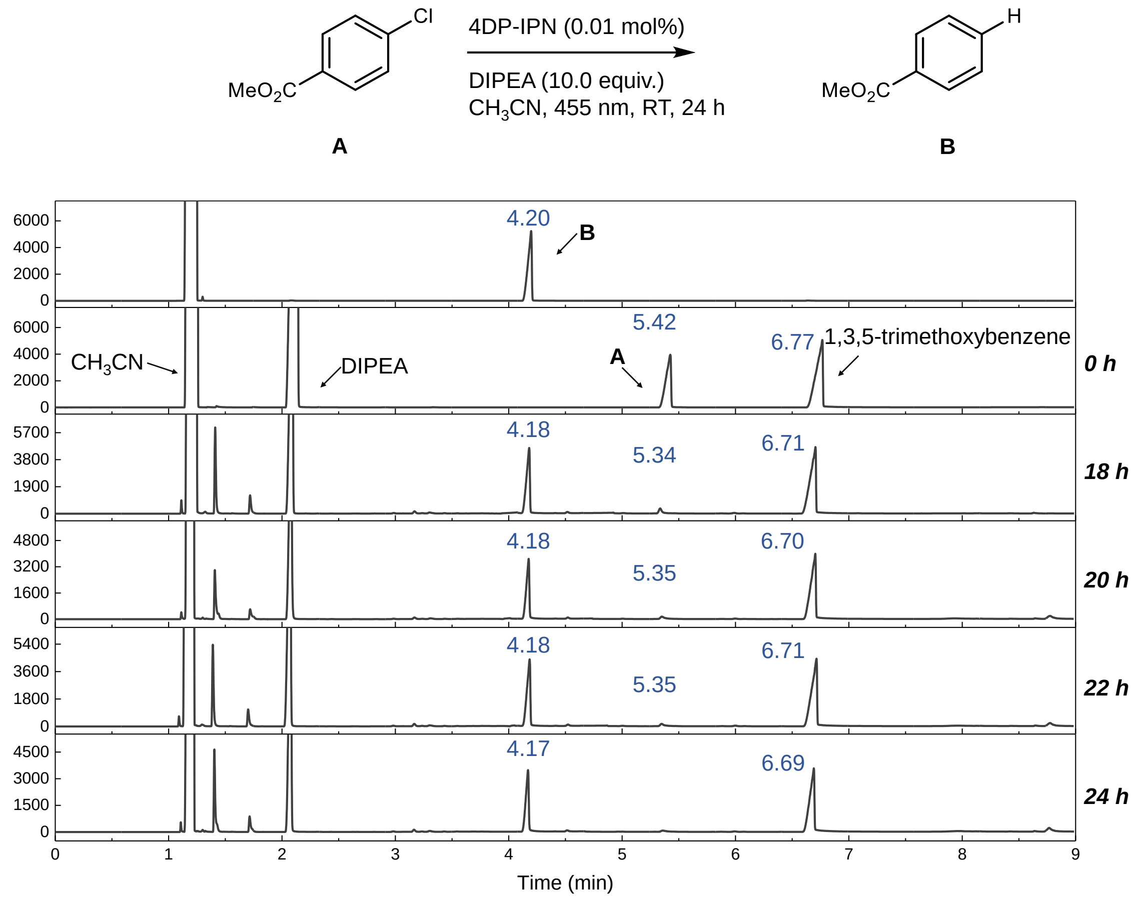

Supplementary Figure 95 GC-FID spectra of photoredox reductive dehalogenation for methyl 4-chlorobenzoate. Yield was measured by GC-FID using 1,3,5-trimethoxybenzene as an internal standard.

Substrate screening (methyl 4-chlorobenzoate), 0 h

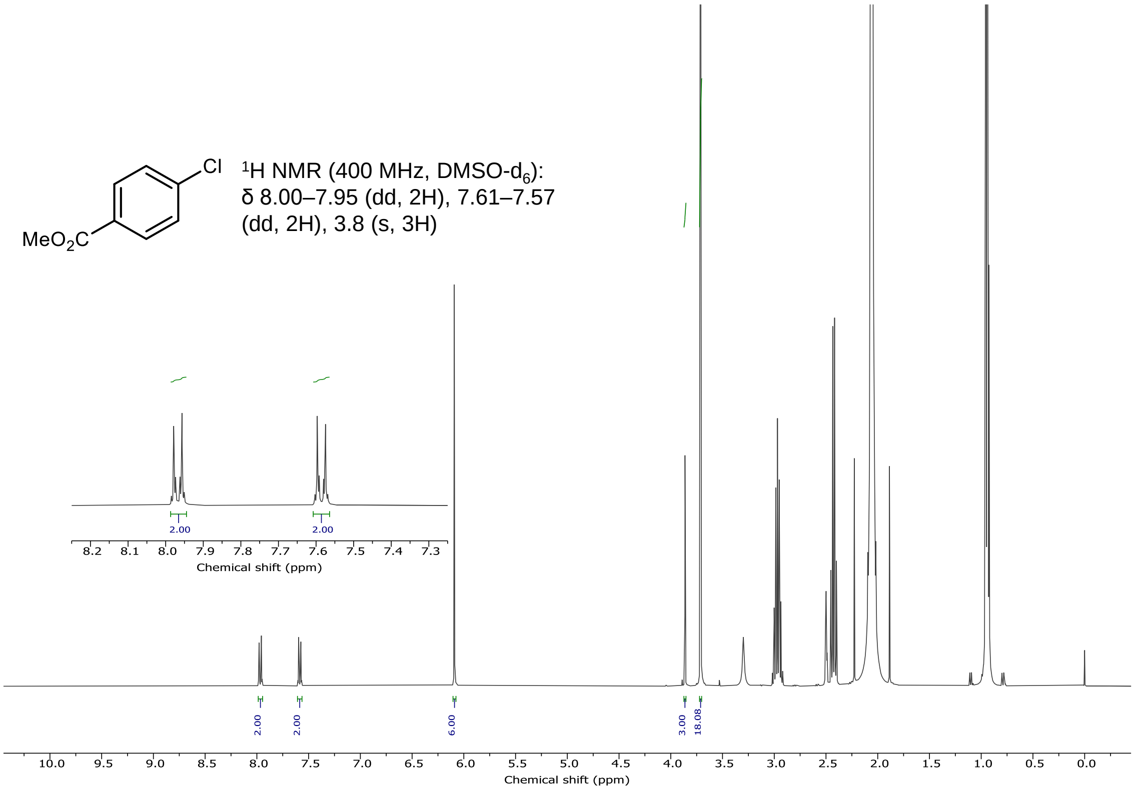

Substrate screening (methyl 4-chlorobenzoate), 24 h

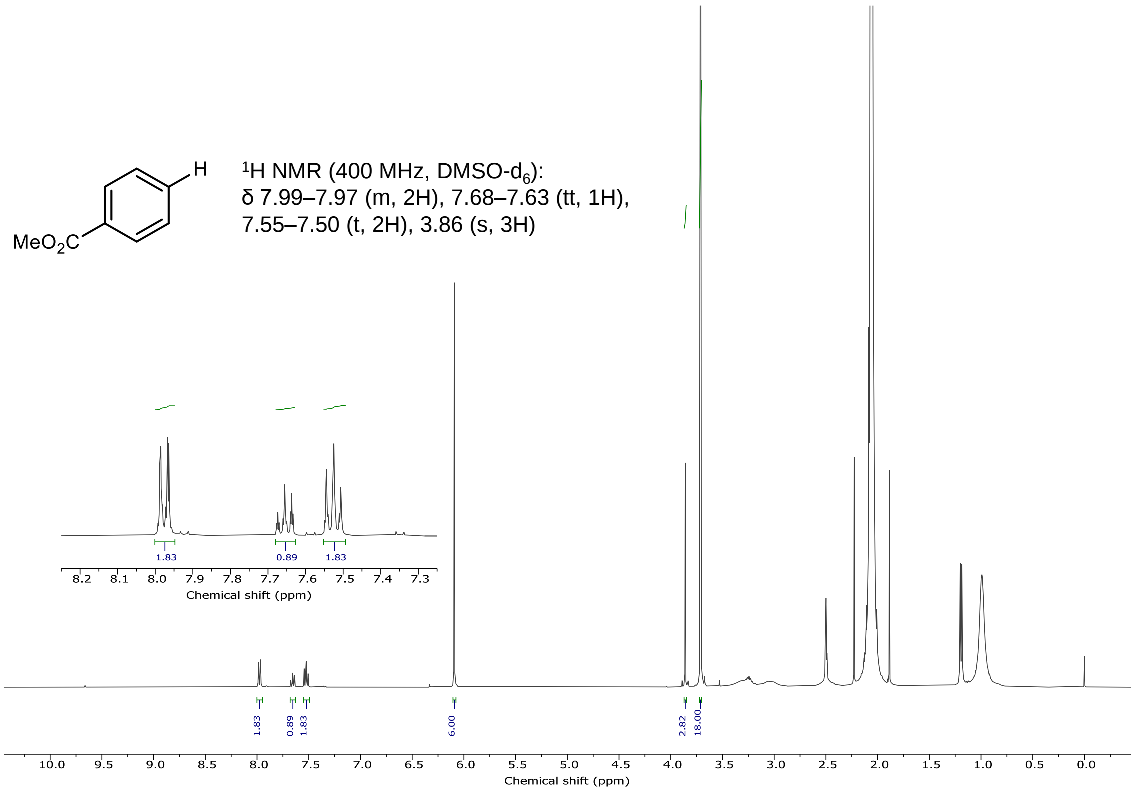

Supplementary Figure 96 ^{1}H NMR data of photoredox reductive dehalogenation for methyl 4-chlorobenzoate (400 MHz, DMSO-d_6); 1,3,5-trimethoxybenzene: δ 6.09 (s, 3H), 3.71 (s, 9H).

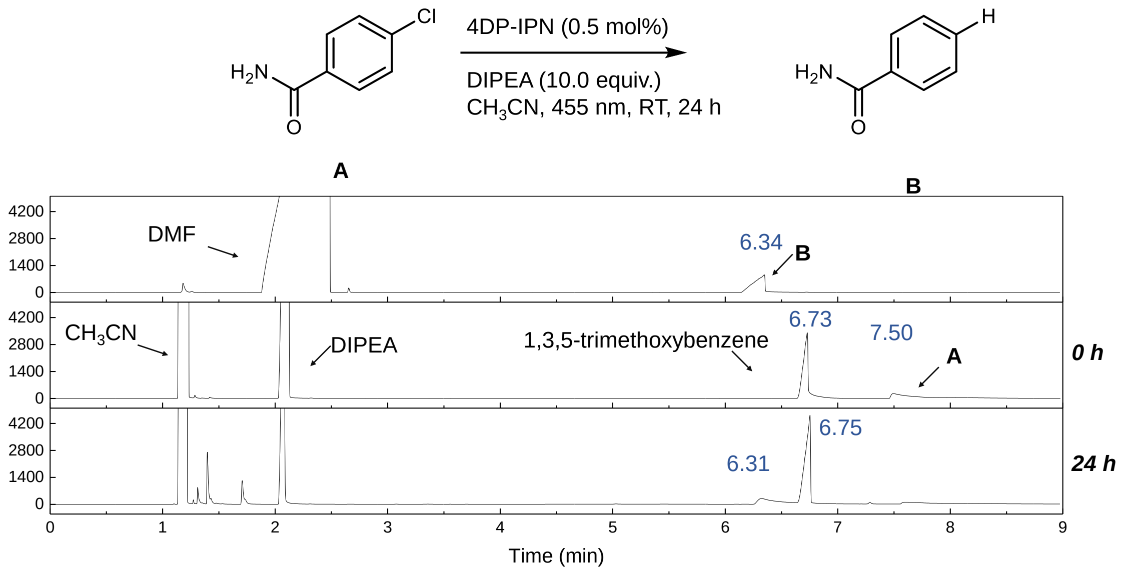

Supplementary Figure 97 GC-FID spectra of photoredox reductive dehalogenation for 4-chlorobenzamide.

Substrate screening (4-chlorobenzamide), 0 h

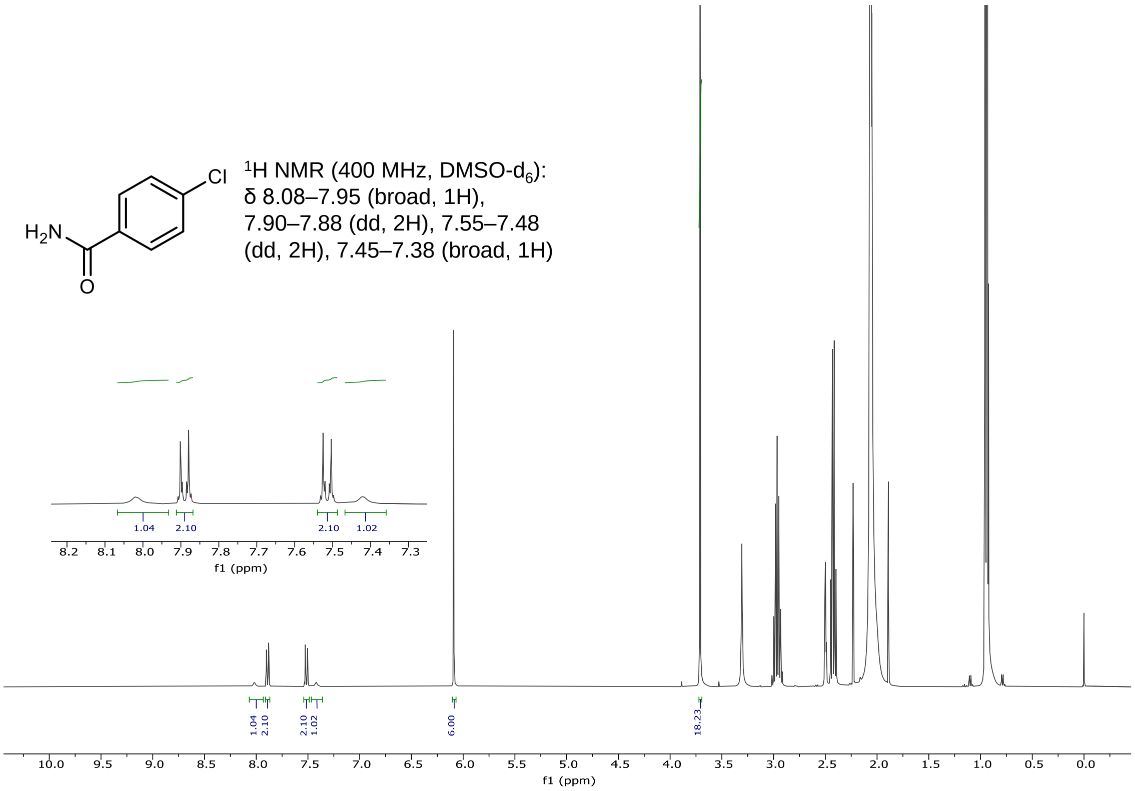

Substrate screening (4-chlorobenzamide), 24 h

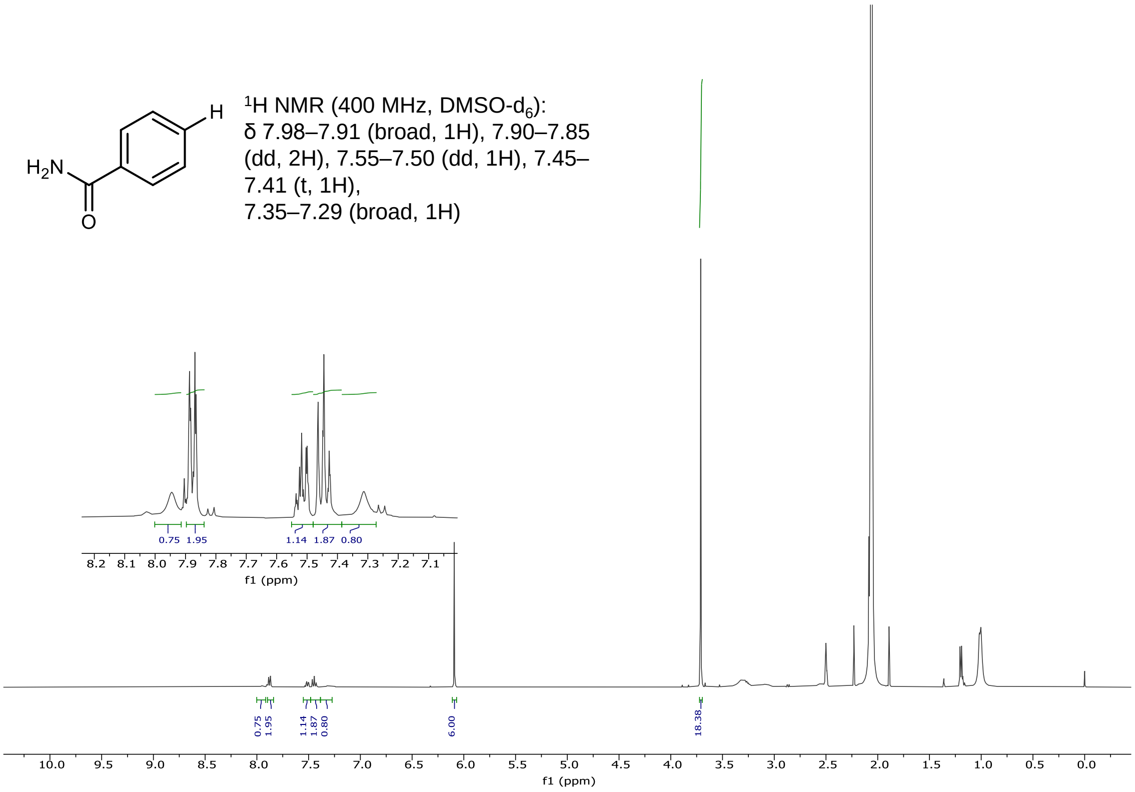

Supplementary Figure 98 ^{1}H NMR data of photoredox reductive dehalogenation for 4-chlorobenzamide (400 MHz, DMSO-d_6): 1,3,5-trimethoxybenzene: δ 6.09 (s, 3H), 3.71 (s, 9H). Yield was measured by ^{1}H NMR using 1,3,5-trimethoxybenzene as an internal standard.

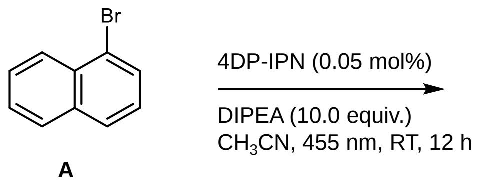

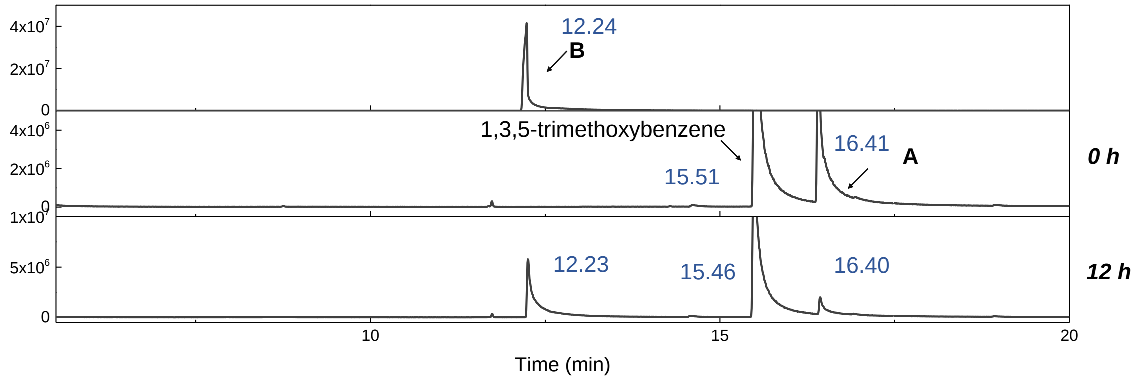

Supplementary Figure 99 GC-FID spectra of photoredox reductive dehalogenation for 1-bromonaphthalene.

Substrate screening (1-bromonaphthalene), 0 h

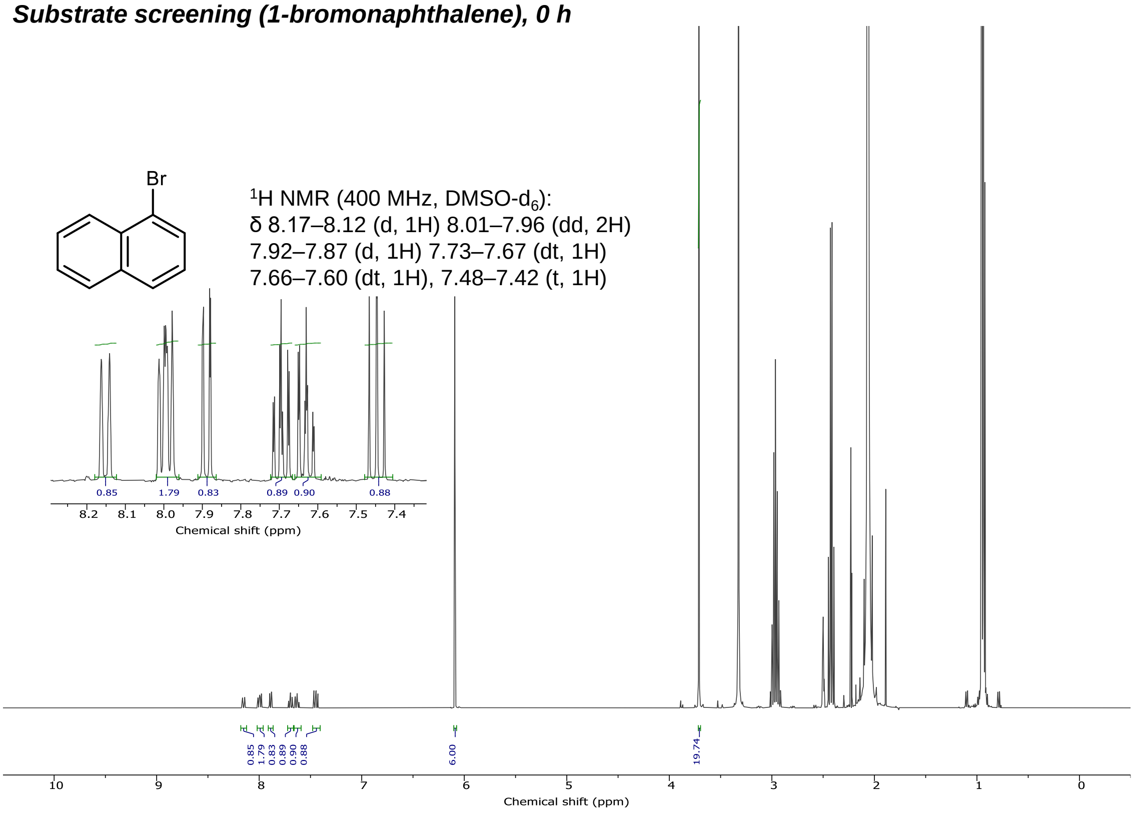

Substrate screening (1-bromonaphthalene), 12 h

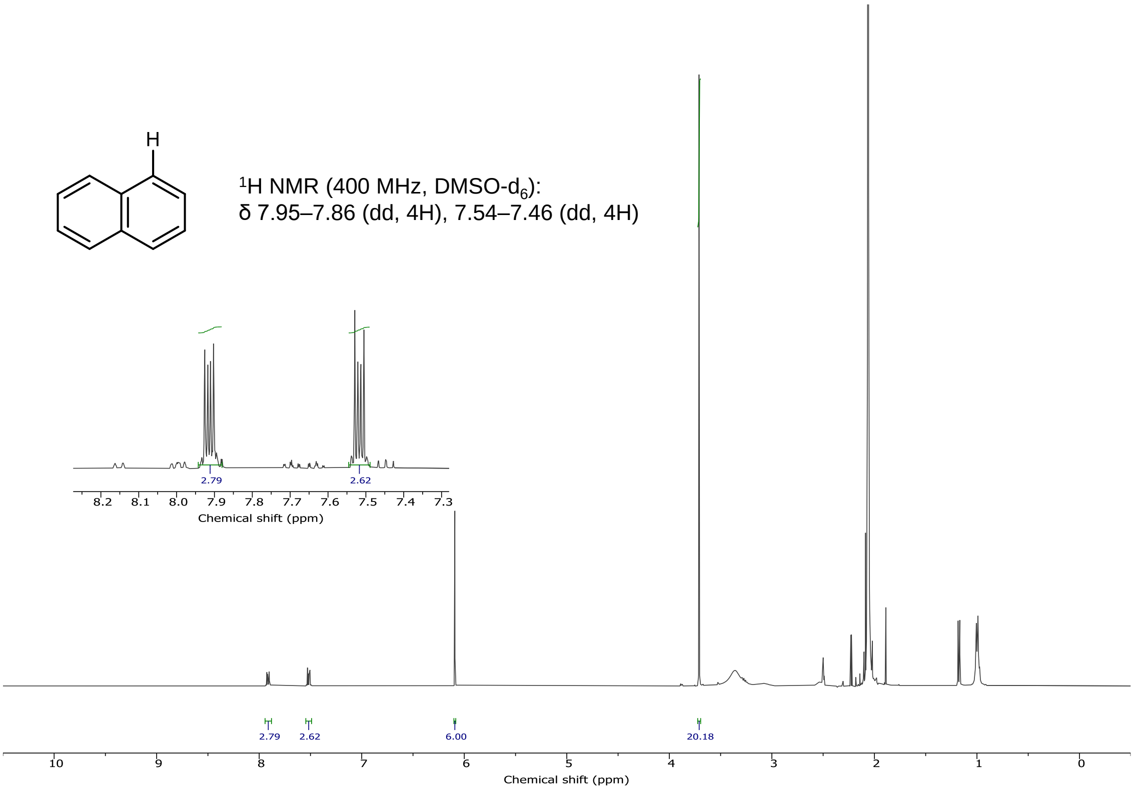

Supplementary Figure 100 ^{1}H NMR data of photoredox reductive dehalogenation for 1-bromonaphthalene (400 MHz, DMSO-d_6); 1,3,5-trimethoxybenzene: δ 6.09 (s, 3H), 3.71 (s, 9H). Yield was measured by ^{1}H NMR using 1,3,5-trimethoxybenzene as an internal standard.

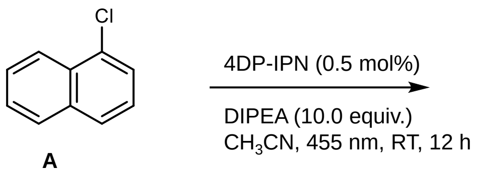

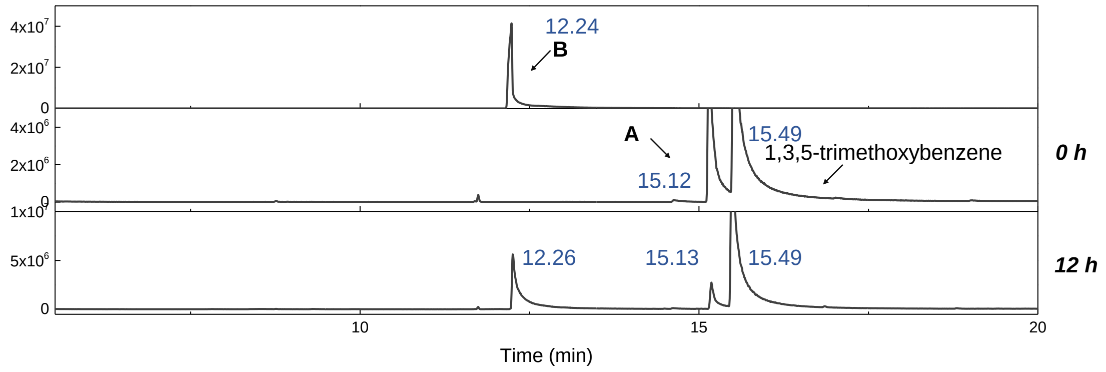

Supplementary Figure 101 GC-FID spectra of photoredox reductive dehalogenation for 1-chloronaphthalene.

Substrate screening (1-chloronaphthalene), 0 h

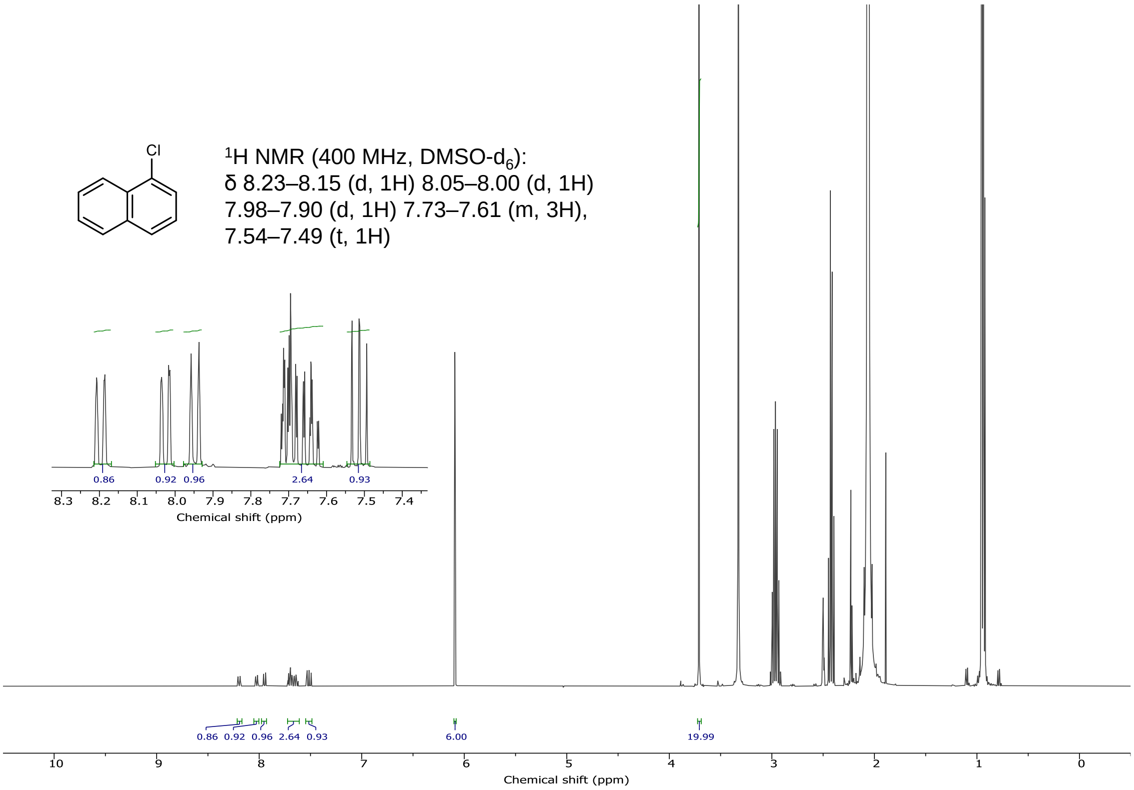

Substrate screening (1-chloronaphthalene), 12 h

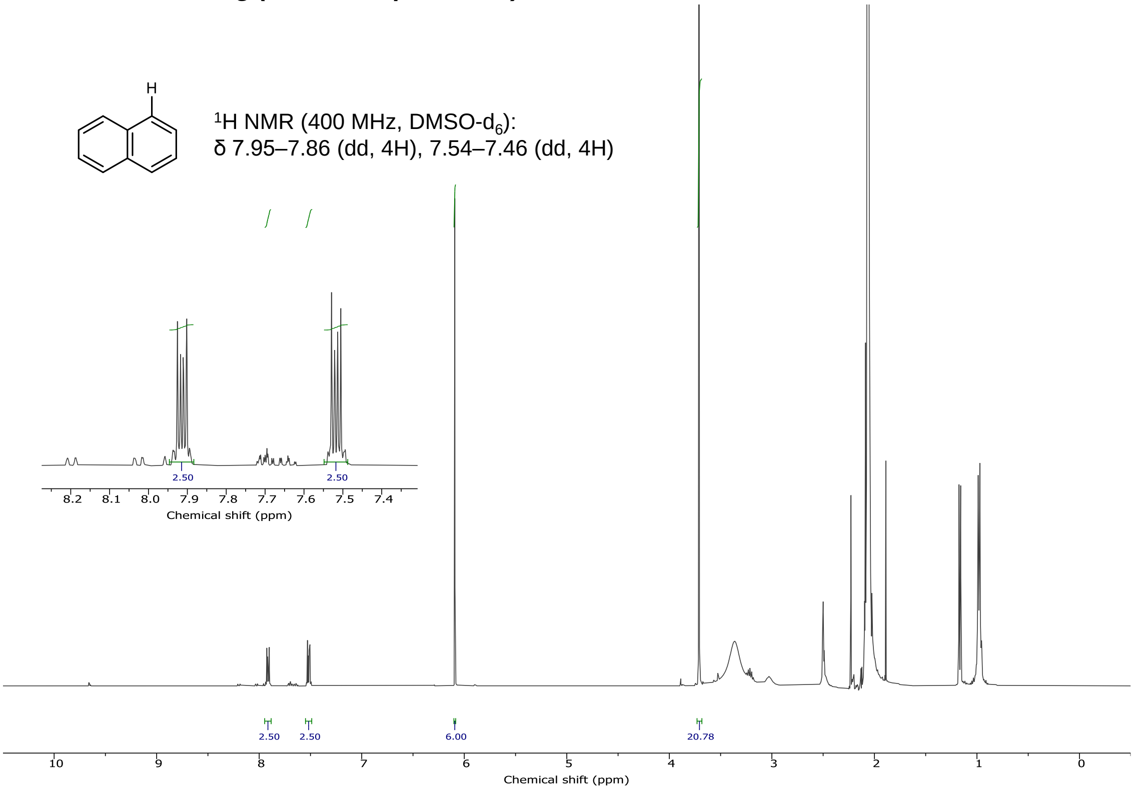

Supplementary Figure 102 ^{1}H NMR data of photoredox reductive dehalogenation for 1-chloronaphthaelene (400 MHz, DMSO-d$_6$); 1,3,5-trimethoxybenzene: δ 6.09 (s, 3H), 3.71 (s, 9H). Yield was measured by ^{1}H NMR using 1,3,5-trimethoxybenzene as an internal standard.

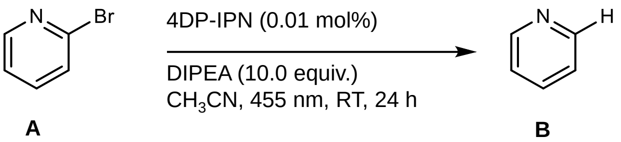

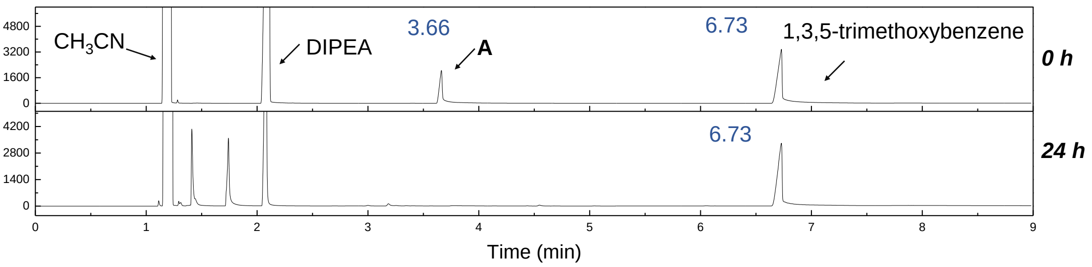

Supplementary Figure 103 GC-FID spectra of photoredox reductive dehalogenation for 2-bromopyridine.

Substrate screening (2-bromopyridine), 0 h

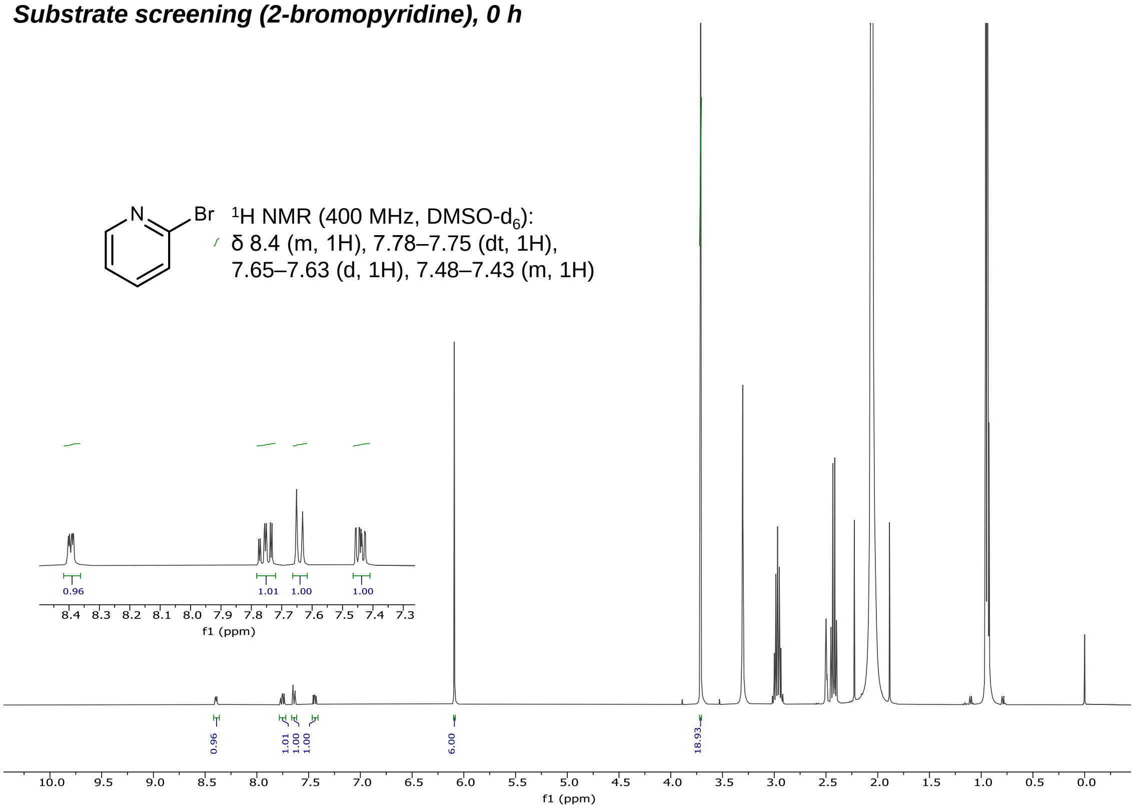

Substrate screening (2-bromopyridine), 24 h

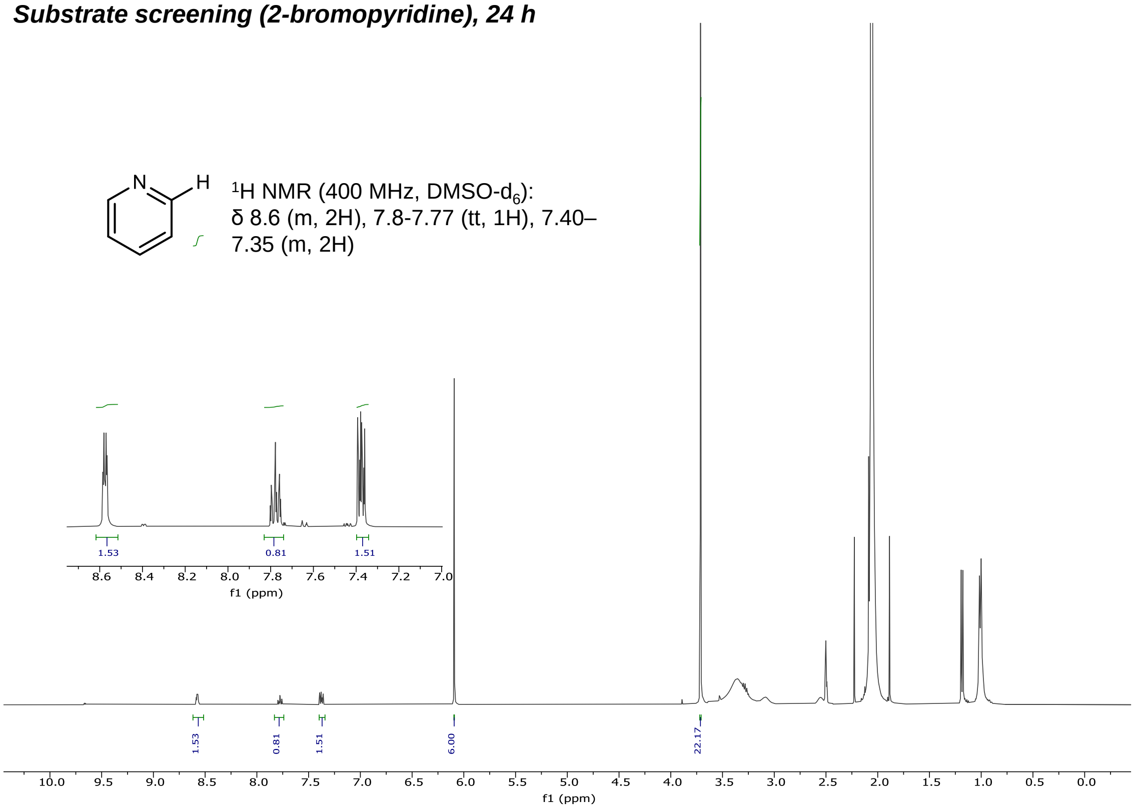

Supplementary Figure 104 ^{1}H NMR data of photoredox reductive dehalogenation for 2-bromopyridine (400 MHz, DMSO-d_6); 1,3,5-trimethoxybenzene: δ 6.09 (s, 3H), 3.71 (s, 9H). Yield was measured by ^{1}H NMR using 1,3,5-trimethoxybenzene as an internal standard.

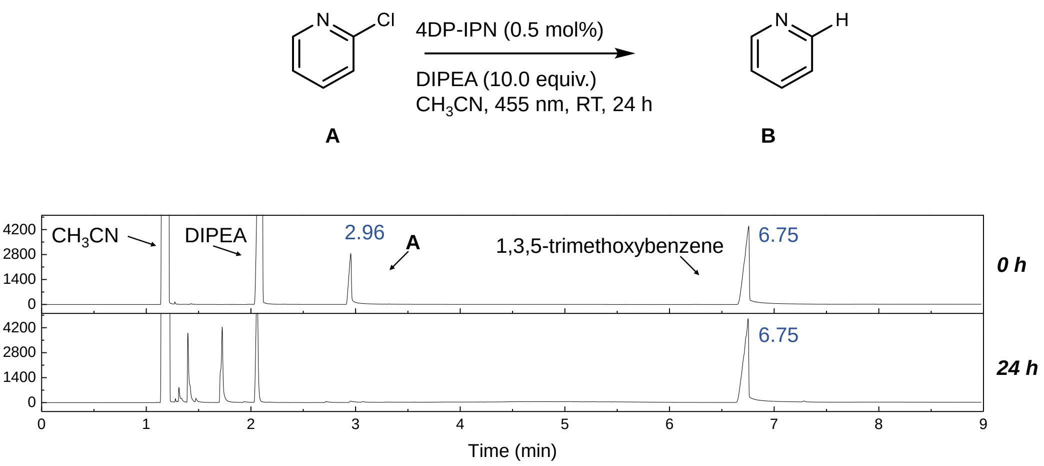

Supplementary Figure 105 GC-FID spectra of photoredox reductive dehalogenation for 2-chloropyridine.

Substrate screening (2-chloropyridine), 0 h

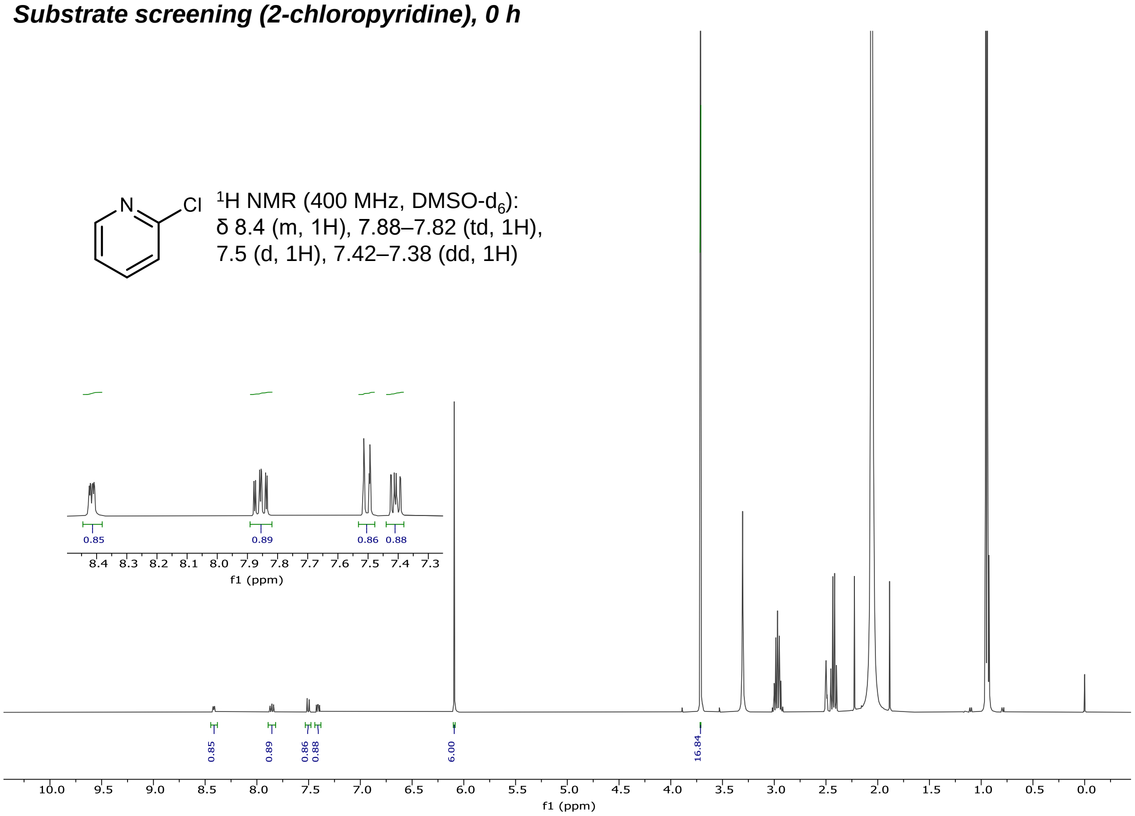

Substrate screening (2-chloropyridine), 24 h

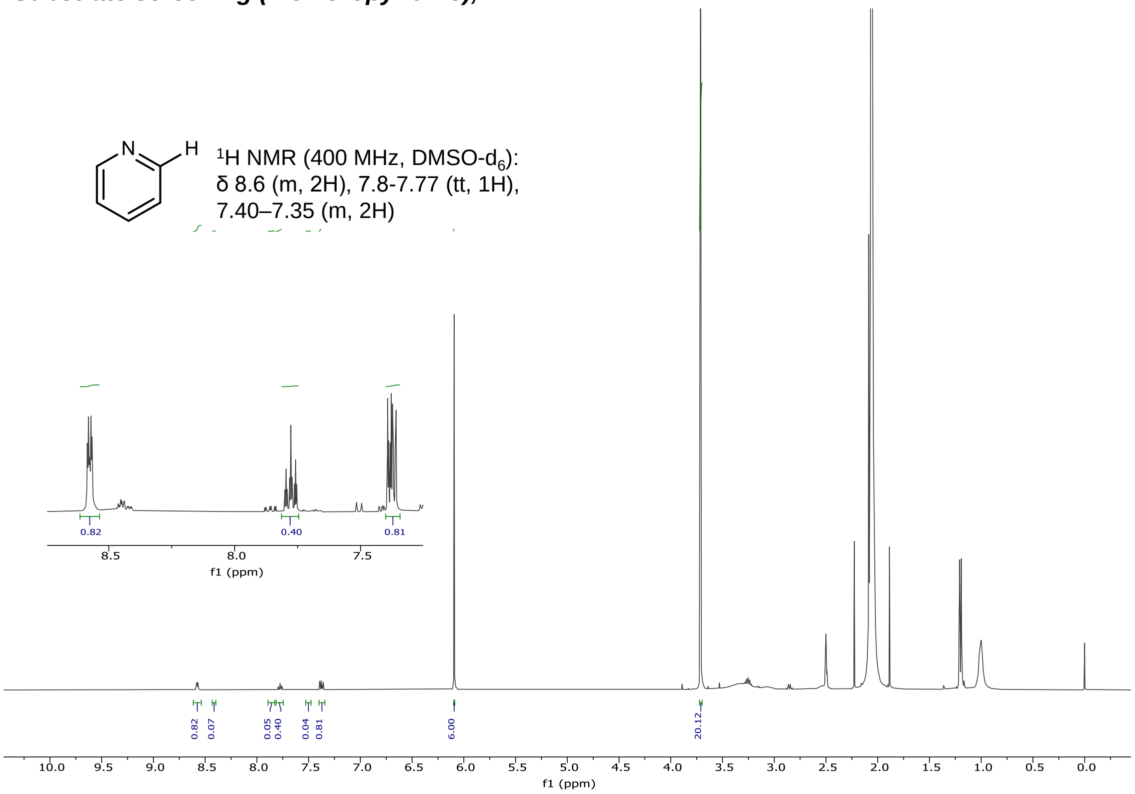

Supplementary Figure 106 ^{1}H NMR data of photoredox reductive dehalogenation for 2-chloropyridine (400 MHz, DMSO-d_6); 1,3,5-trimethoxybenzene: δ 6.09 (s, 3H), 3.71 (s, 9H). Yield was measured by ^{1}H NMR using 1,3,5-trimethoxybenzene as an internal standard.

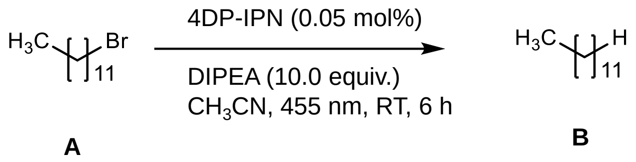

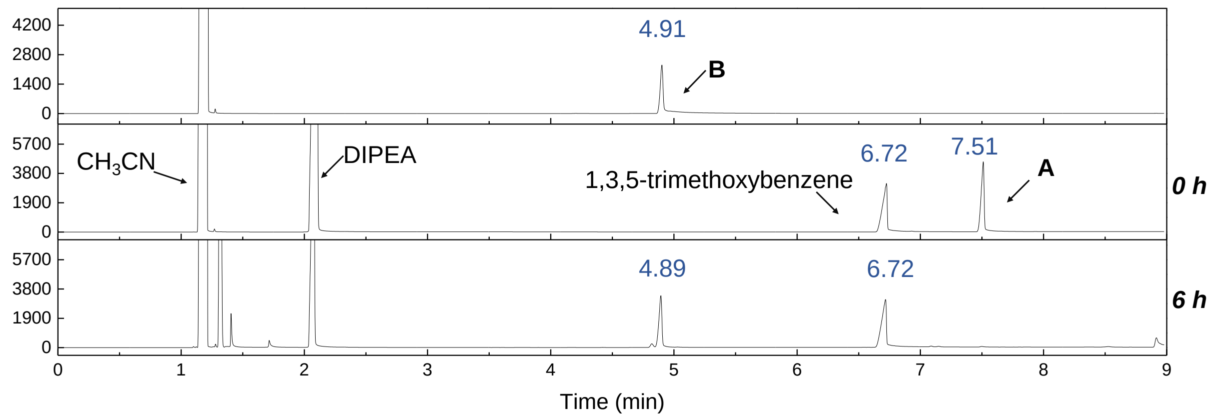

Supplementary Figure 107 GC-FID spectra of photoredox reductive dehalogenation for 1-bromododecane.

Substrate screening (1-bromododecane), 0 h

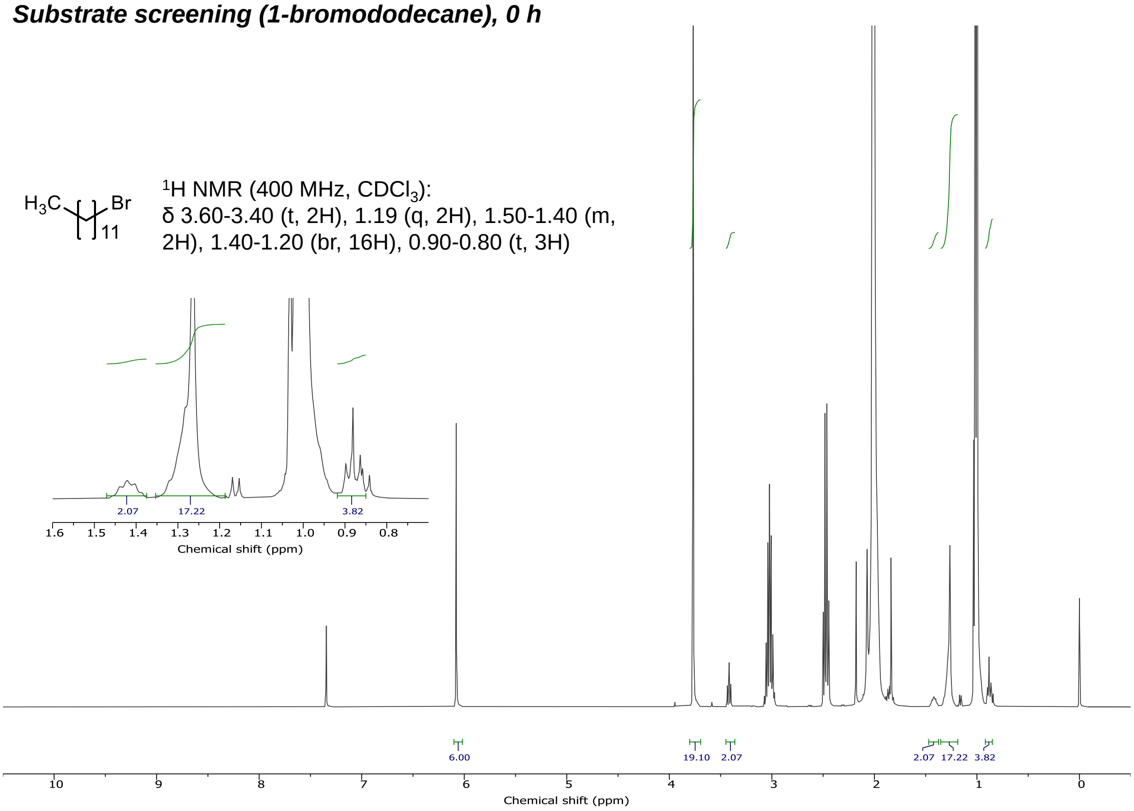

Substrate screening (1-bromododecane), 6 h

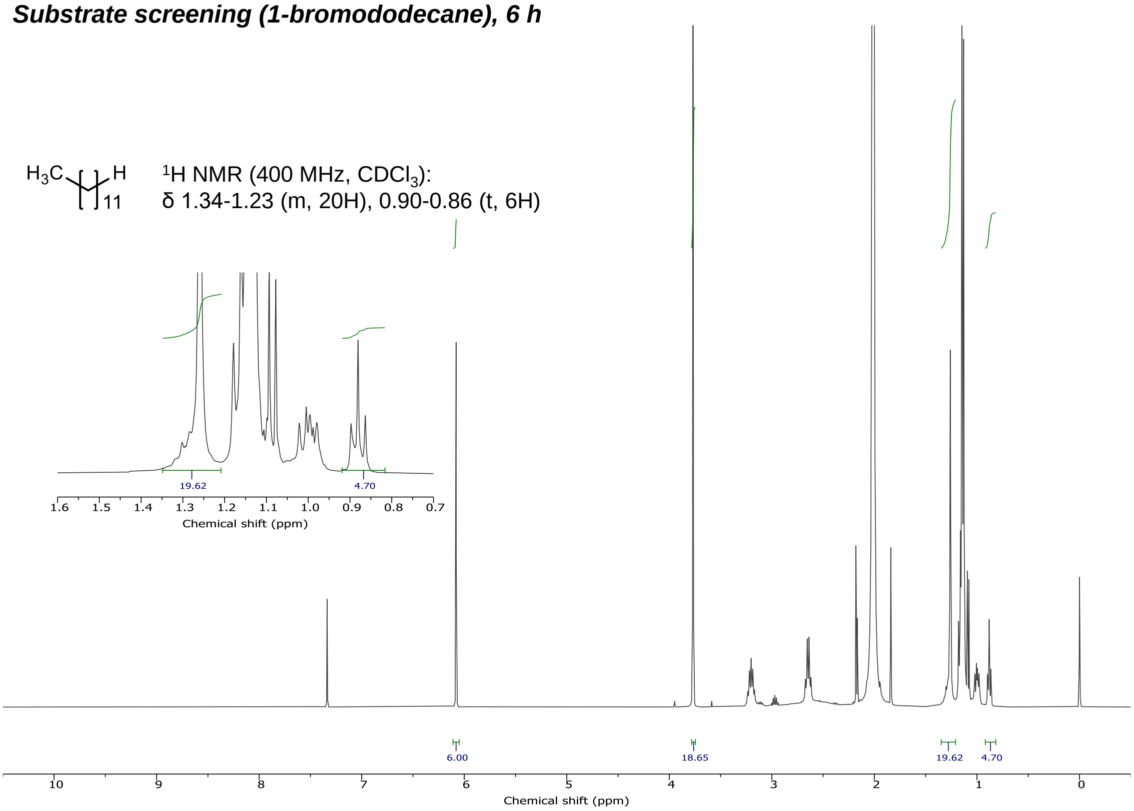

Supplementary Figure 108 ^{1}H NMR data of photoredox reductive dehalogenation for 1-bromododecane (400 MHz, $CDCl_3$); 1,3,5-trimethoxybenzene: δ 6.09 (s, 3H), 3.71 (s, 9H). Yield was measured by ^{1}H NMR using 1,3,5-trimethoxybenzene as an internal standard.

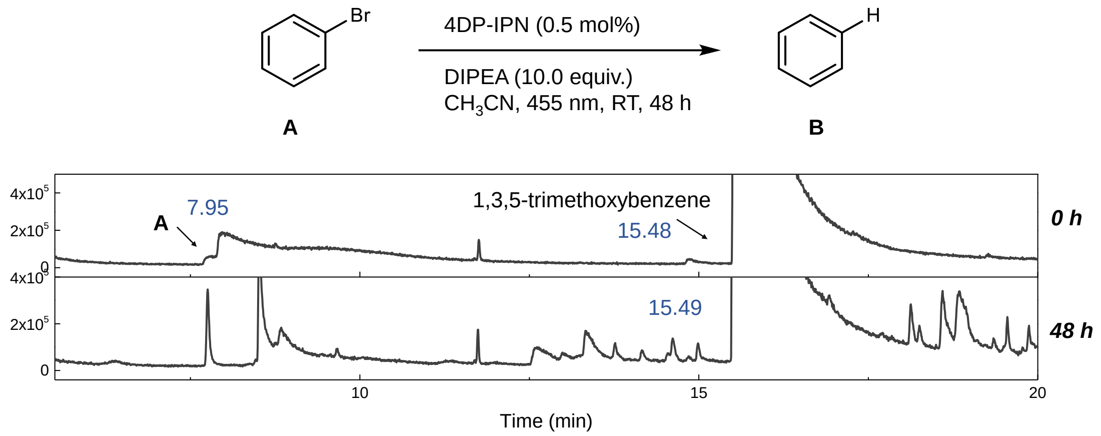

Supplementary Figure 109 GC-FID spectra of photoredox reductive dehalogenation for bromobenzene. Due to high injection-temperature of GC-FID, benzene product was not observed in our GC-FID system. Therefore, we evaluated the conversion and yield from ^{1}H NMR using 1,3,5-trimethoxybenzene as an internal standard.

Substrate screening (bromobenzene), 0 h

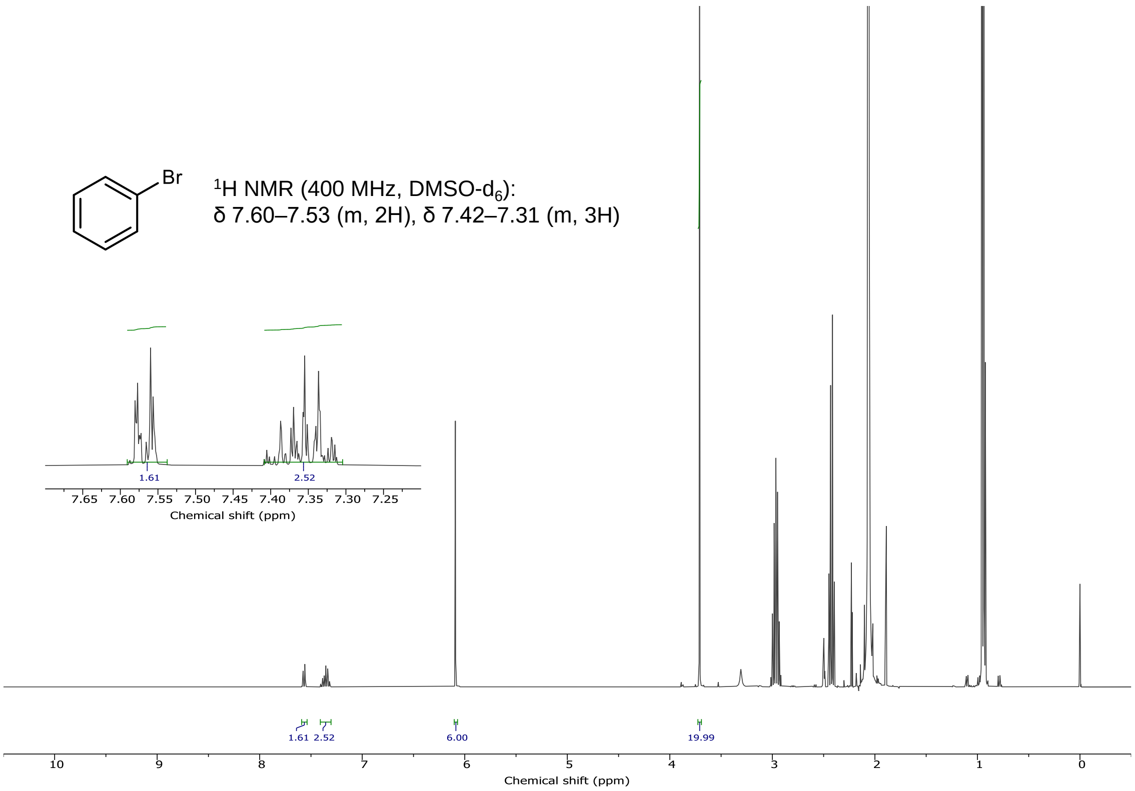

Substrate screening (bromobenzene), 48 h

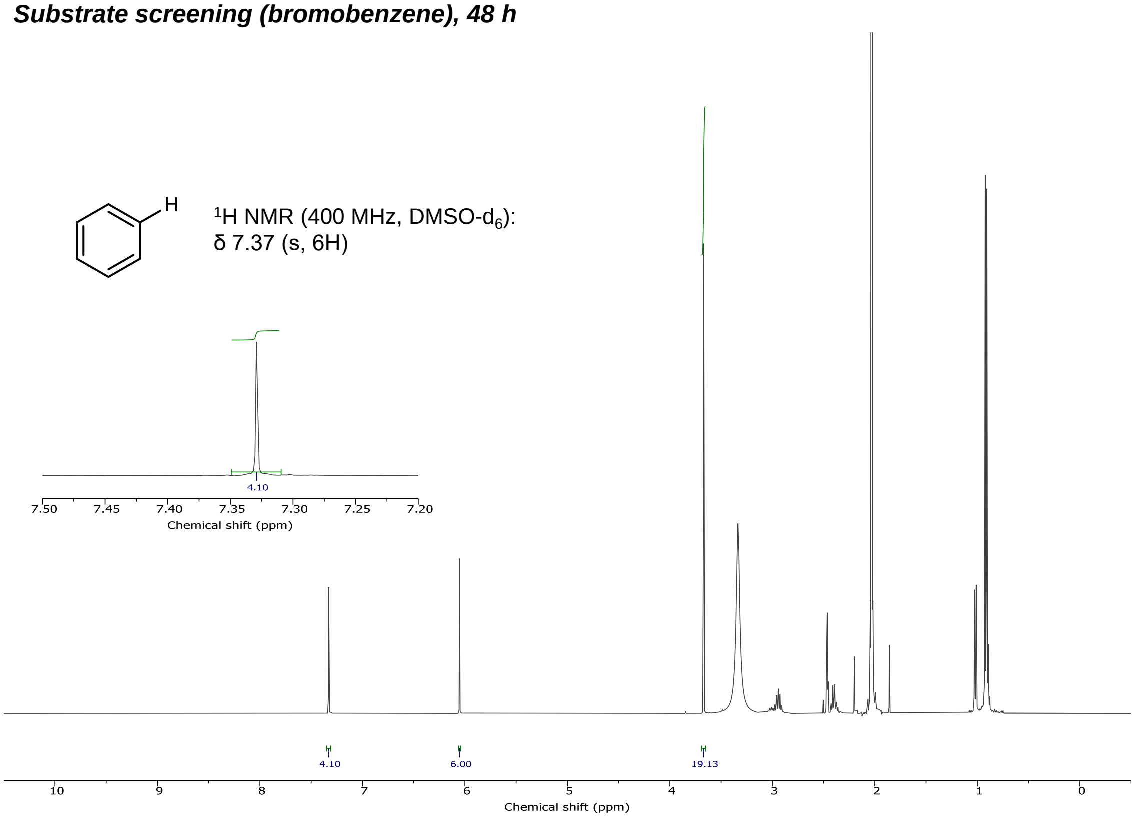

Supplementary Figure 110 ^1H NMR data for photoredox reductive dehalogenation for bromobenzene (400 MHz, DMSO-d_6); 1,3,5-trimethoxybenzene: δ 6.09 (s, 3H), 3.71 (s, 9H). Yield was measured by ^1H NMR using 1,3,5-trimethoxybenzene as an internal standard.

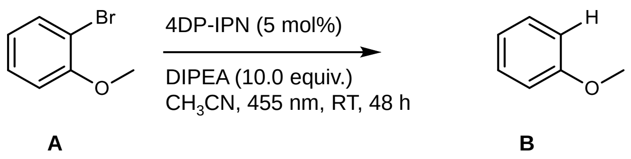

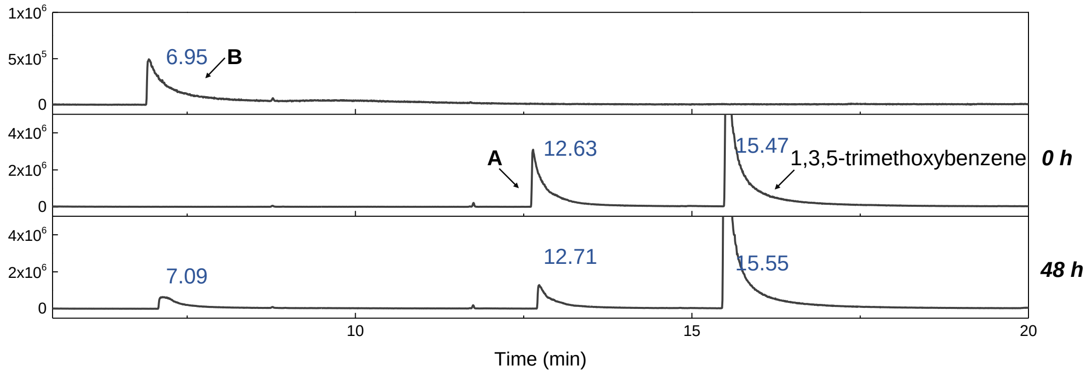

Supplementary Figure 111 GC-FID spectra for photoredox reductive dehalogenation for 2-bromoanisole.

Substrate screening (2-bromoanisole), 0 h

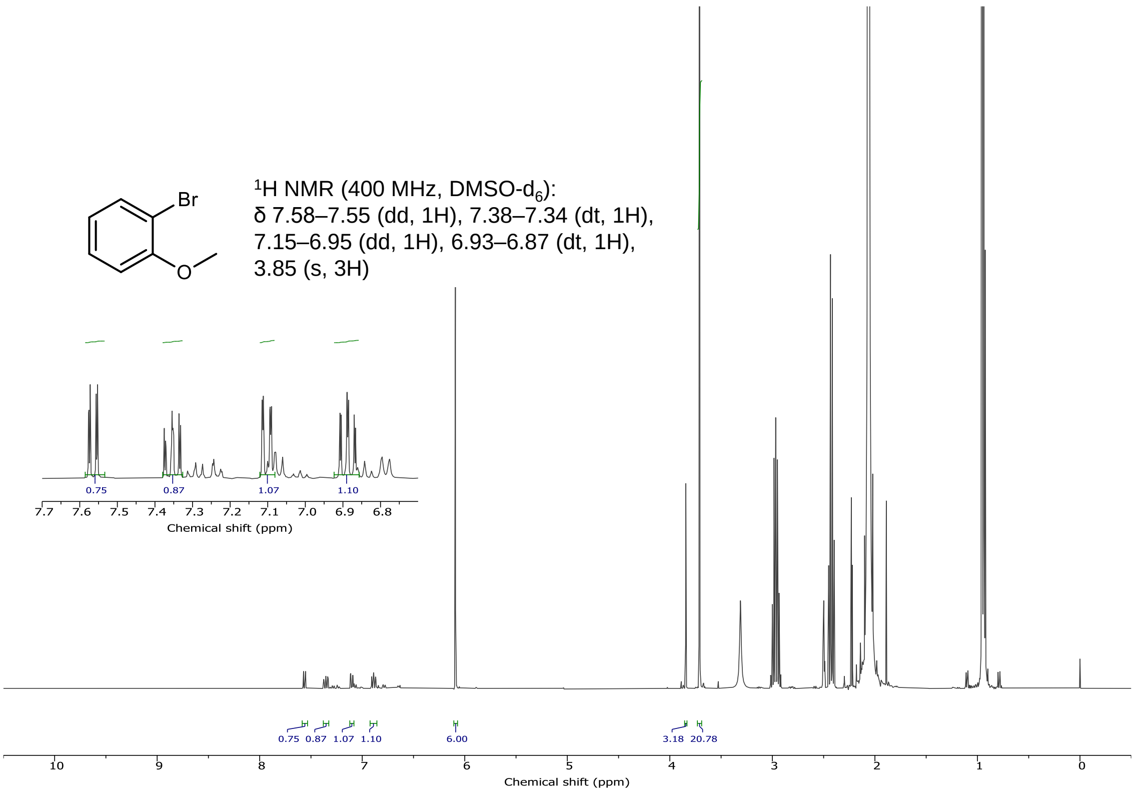

Substrate screening (2-bromoanisole), 48 h

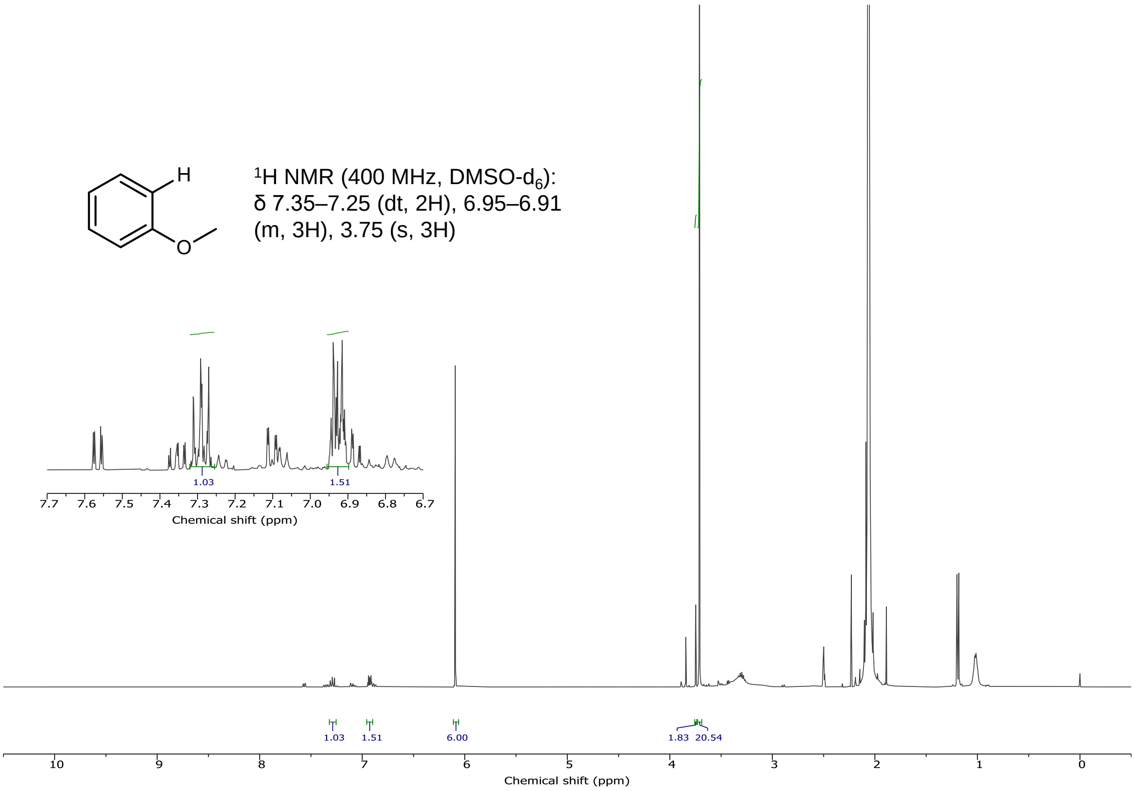

Supplementary Figure 112 ^{1}H NMR data for photoredox reductive dehalogenation for 2-bromoanisole (400 MHz, DMSO-d_6); 1,3,5-trimethoxybenzene: δ 6.09 (s, 3H), 3.71 (s, 9H). Yield was measured by ^{1}H NMR using 1,3,5-trimethoxybenzene as an internal standard.

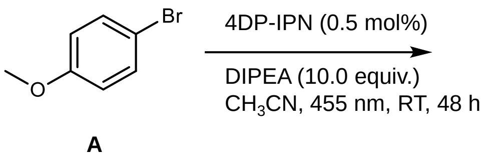

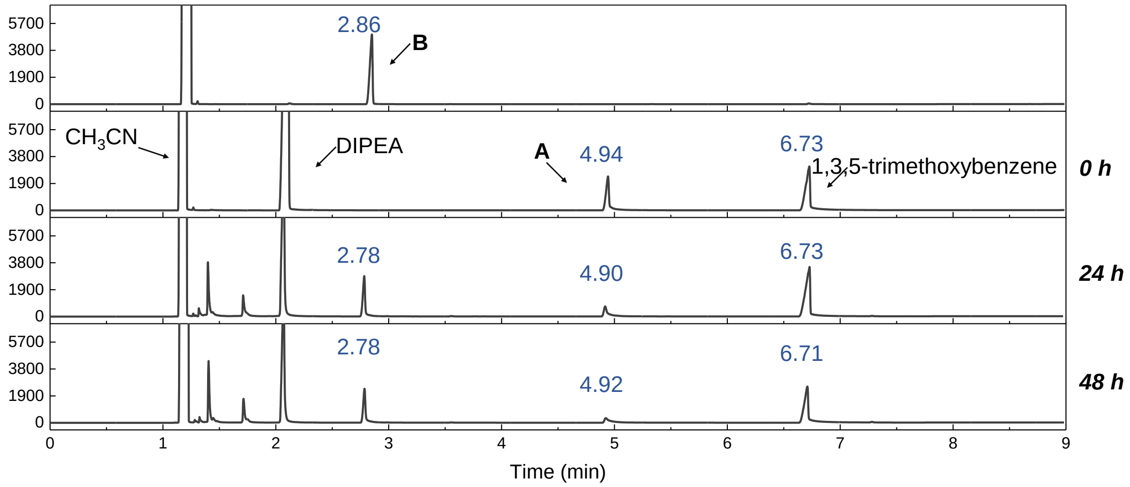

Supplementary Figure 113 GC-FID spectra for photoredox reductive dehalogenation for 4-bromoanisole.

Substrate screening (4-bromoanisole), 0 h

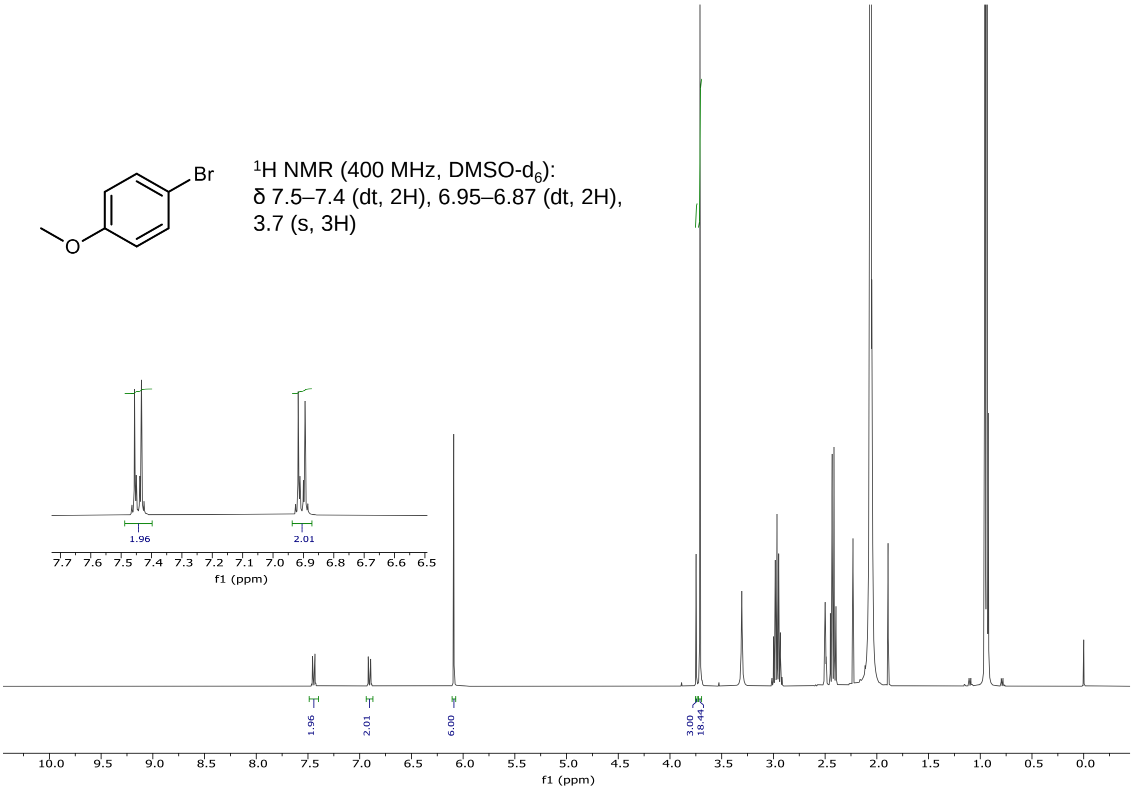

Substrate screening (4-bromoanisole), 48 h

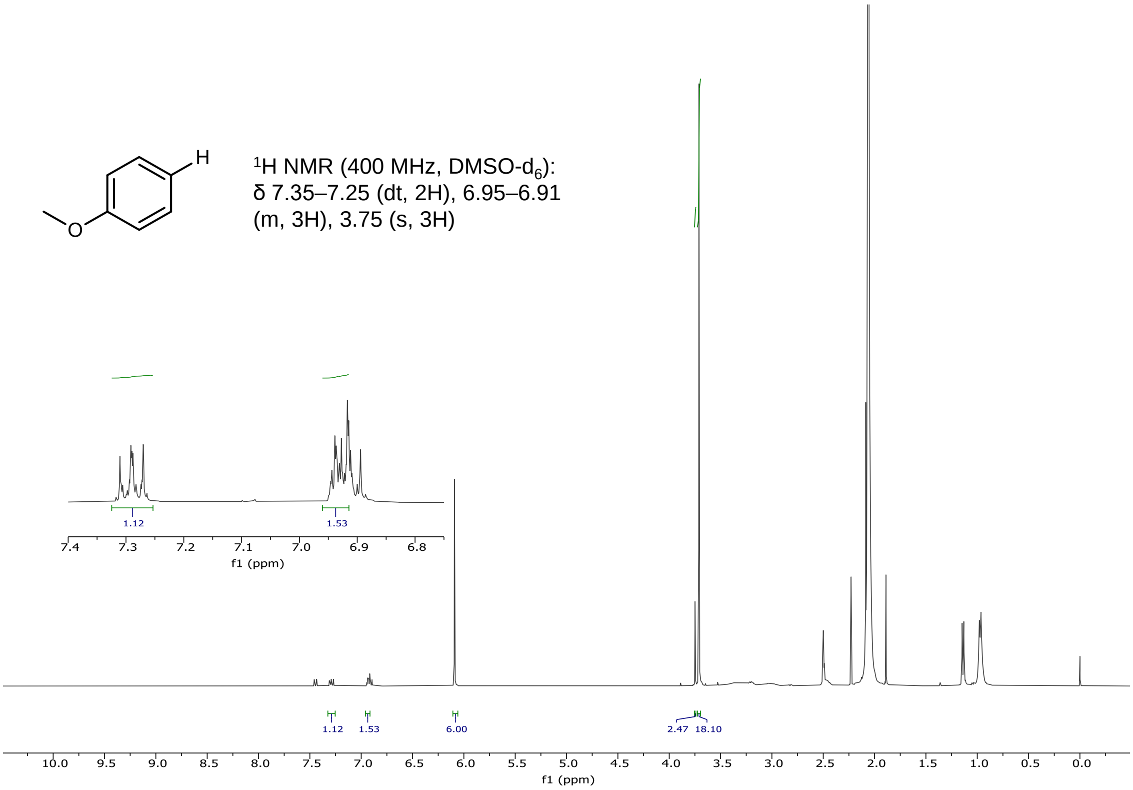

Supplementary Figure 114 ^{1}H NMR data for photoredox reductive dehalogenation for 4-bromoanisole (400 MHz, DMSO-d_6); 1,3,5-trimethoxybenzene: δ 6.09 (s, 3H), 3.71 (s, 9H). Yield was measured by ^{1}H NMR using 1,3,5-trimethoxybenzene as an internal standard.

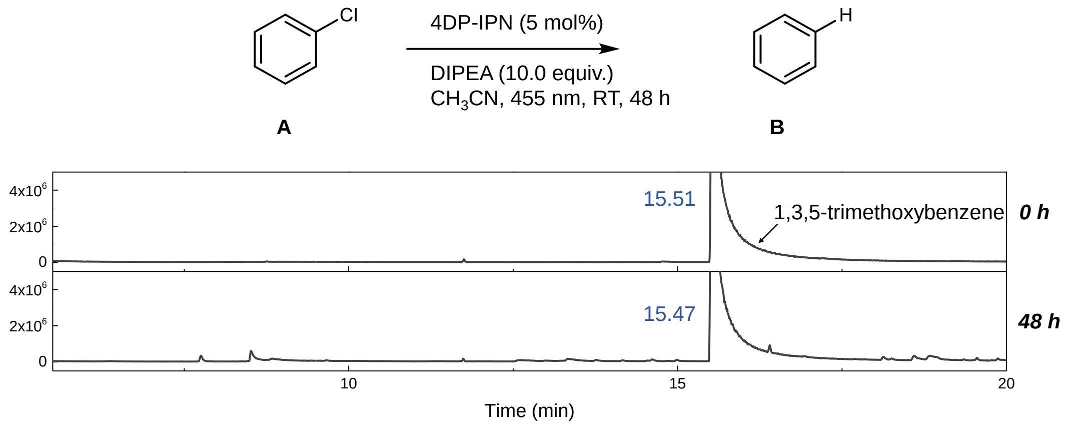

Supplementary Figure 115 GC-FID spectra for photoredox reductive dehalogenation for chlorobenzene. Due to high injection-temperature of GC-FID, chlorobenzene and benzene product was not observed in our GC-FID system. Therefore, we evaluated the conversion and yield from ^{1}H NMR using 1,3,5-trimethoxybenzene as an internal standard.

Substrate screening (chlorobenzene), 0 h

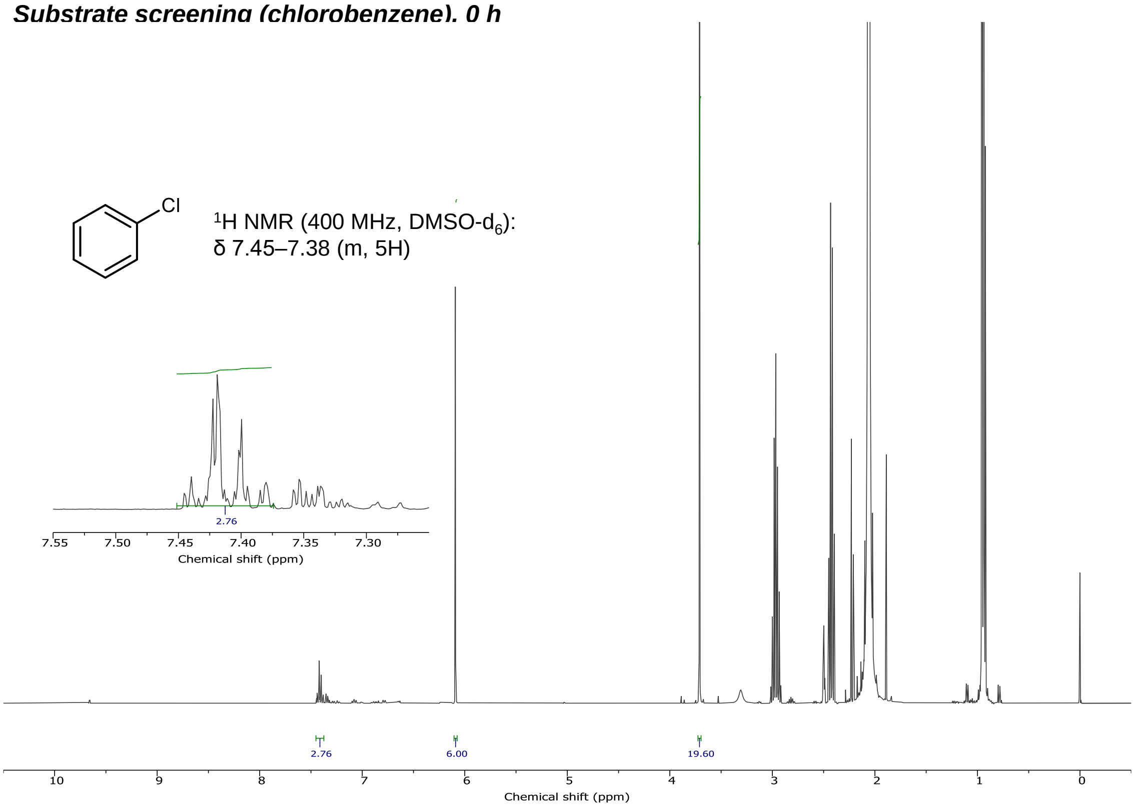

Substrate screening (chlorobenzene), 48 h

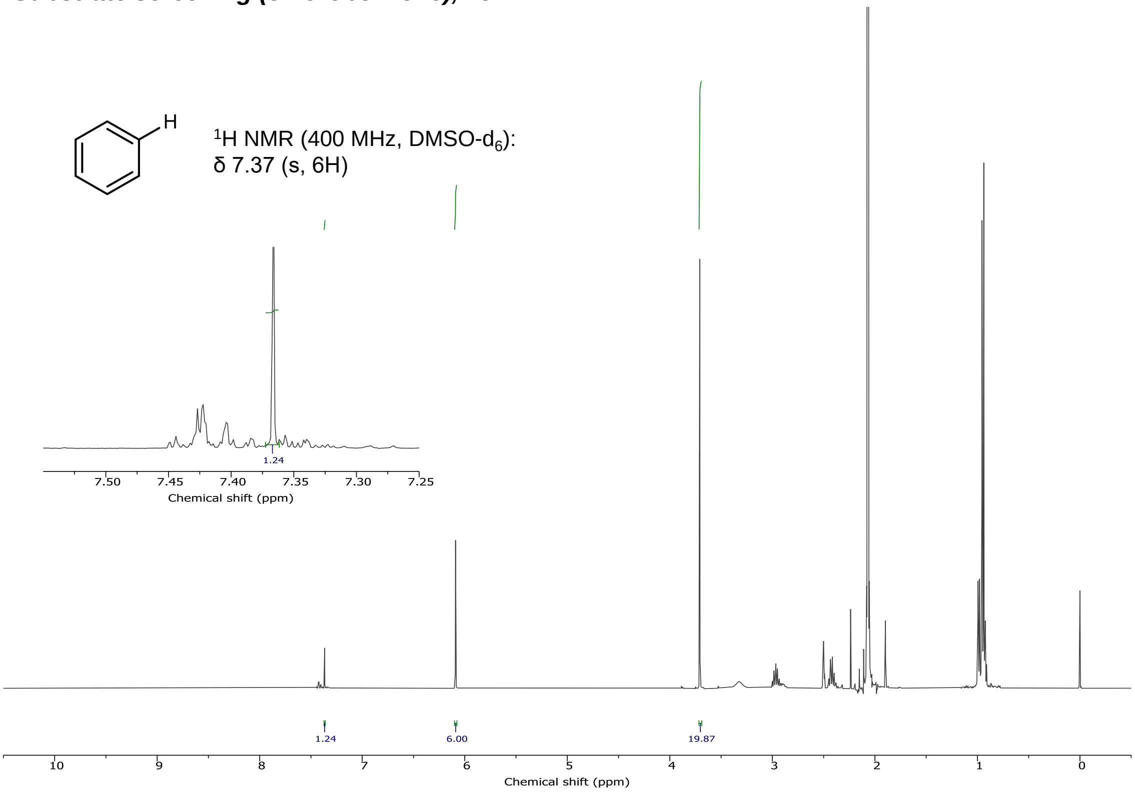

Supplementary Figure 116 ^{1}H NMR data for photoredox reductive dehalogenation for chlorobenzene (400 MHz, DMSO-d_6); 1,3,5-trimethoxybenzene: δ 6.09 (s, 3H), 3.71 (s, 9H). Yield was measured by ^{1}H NMR using 1,3,5-trimethoxybenzene as an internal standard.

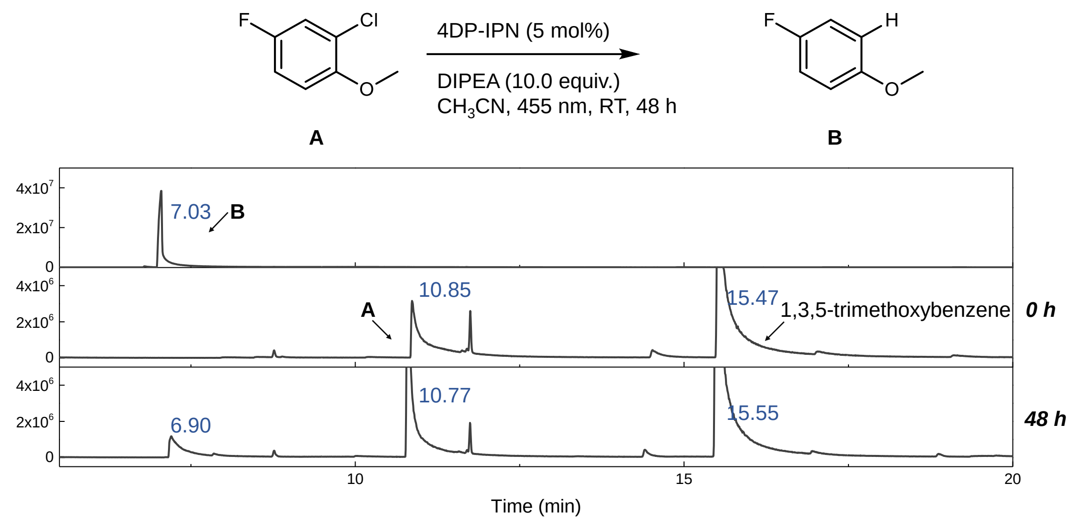

Supplementary Figure 117 GC-FID spectra for photoredox reductive dehalogenation for 2-chloro-4-fluoroanisole.

Substrate screening (2-chloro-4-fluoroanisole), 0 h

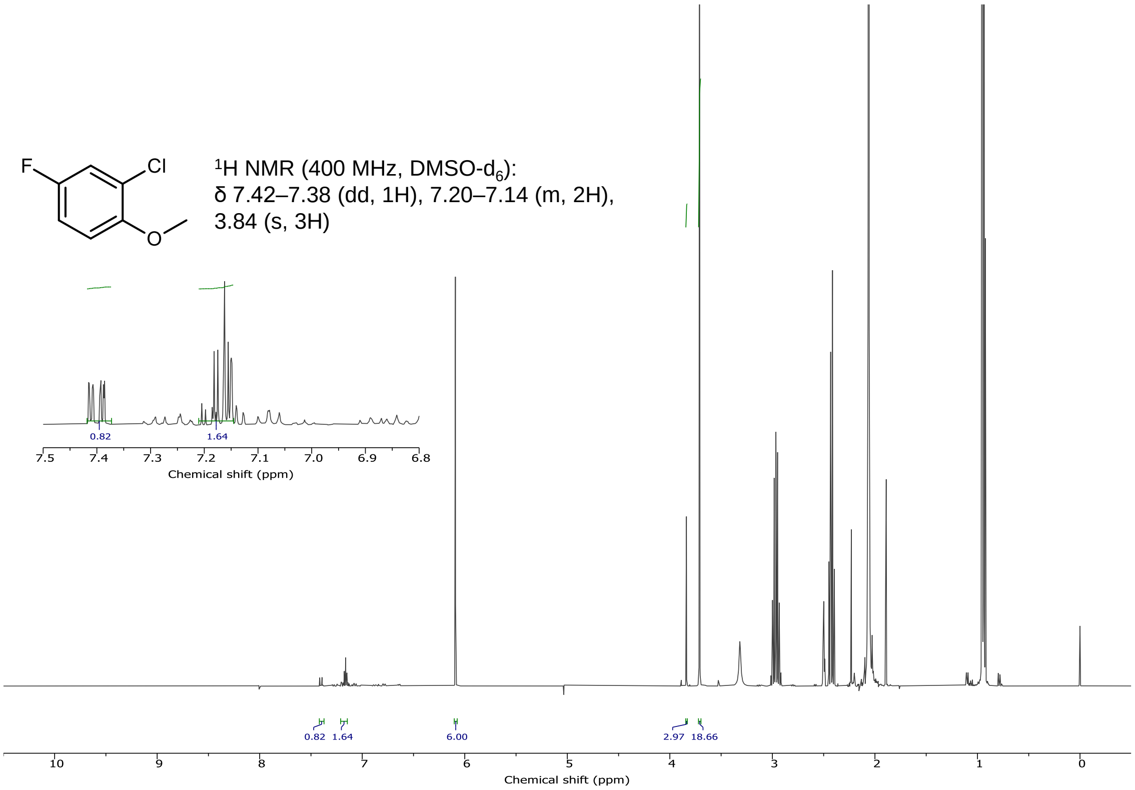

Substrate screening (2-chloro-4-fluoroanisole), 48 h

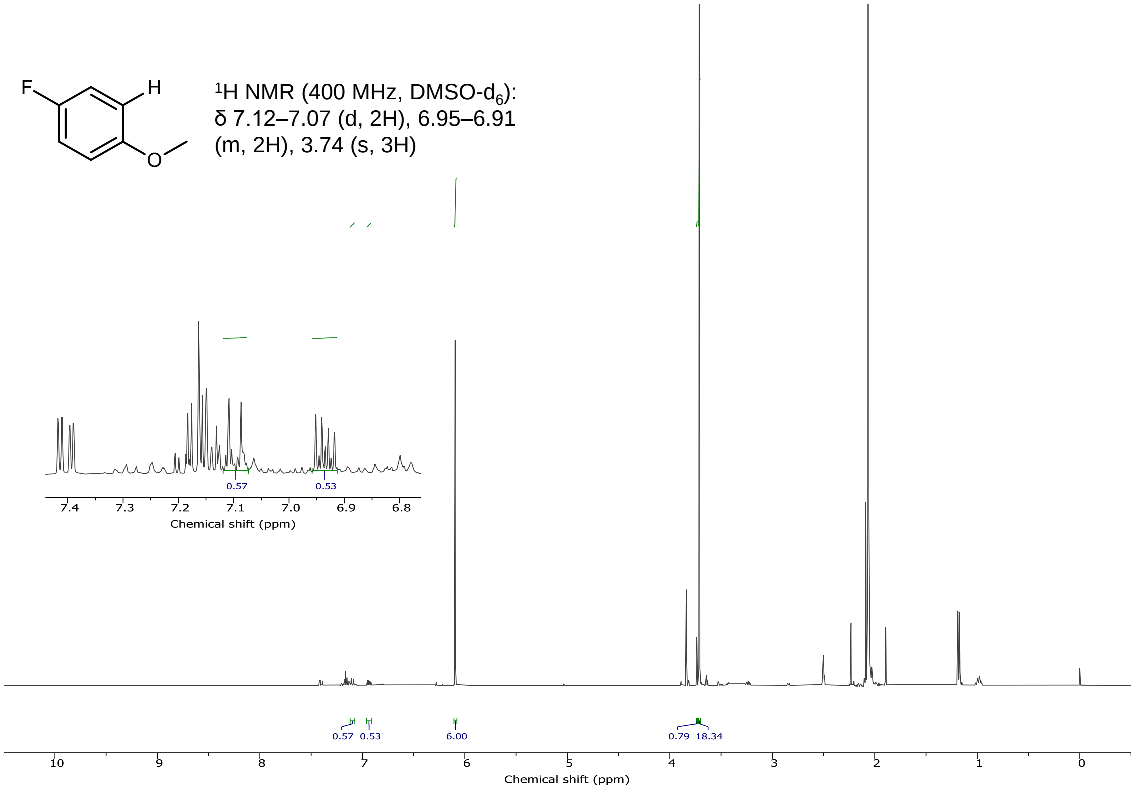

Supplementary Figure 118 ^{1}H NMR data for photoredox reductive dehalogenation for 2-chloro-4-fluoroanisole (400 MHz, DMSO-d_6); 1,3,5-trimethoxybenzene: δ 6.09 (s, 3H), 3.71 (s, 9H). Yield was measured by ^{1}H NMR using 1,3,5-trimethoxybenzene as an internal standard.

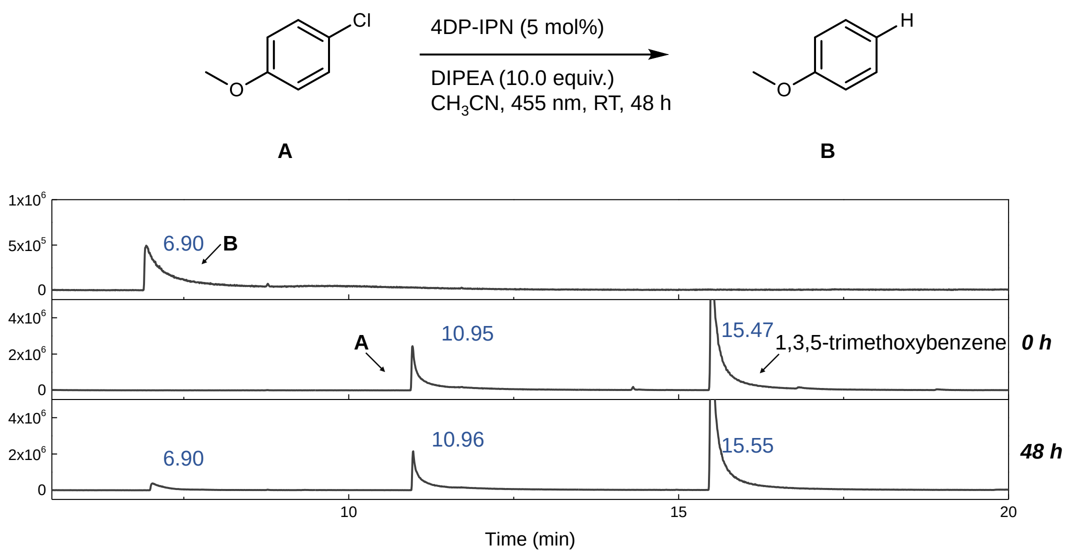

Supplementary Figure 119 GC-FID spectra for photoredox reductive dehalogenation for 4-chloroanisole. Yield was measured by ^{1}H NMR using 1,3,5-trimethoxybenzene as an internal standard.

Substrate screening (4-chloroanisole), 0 h

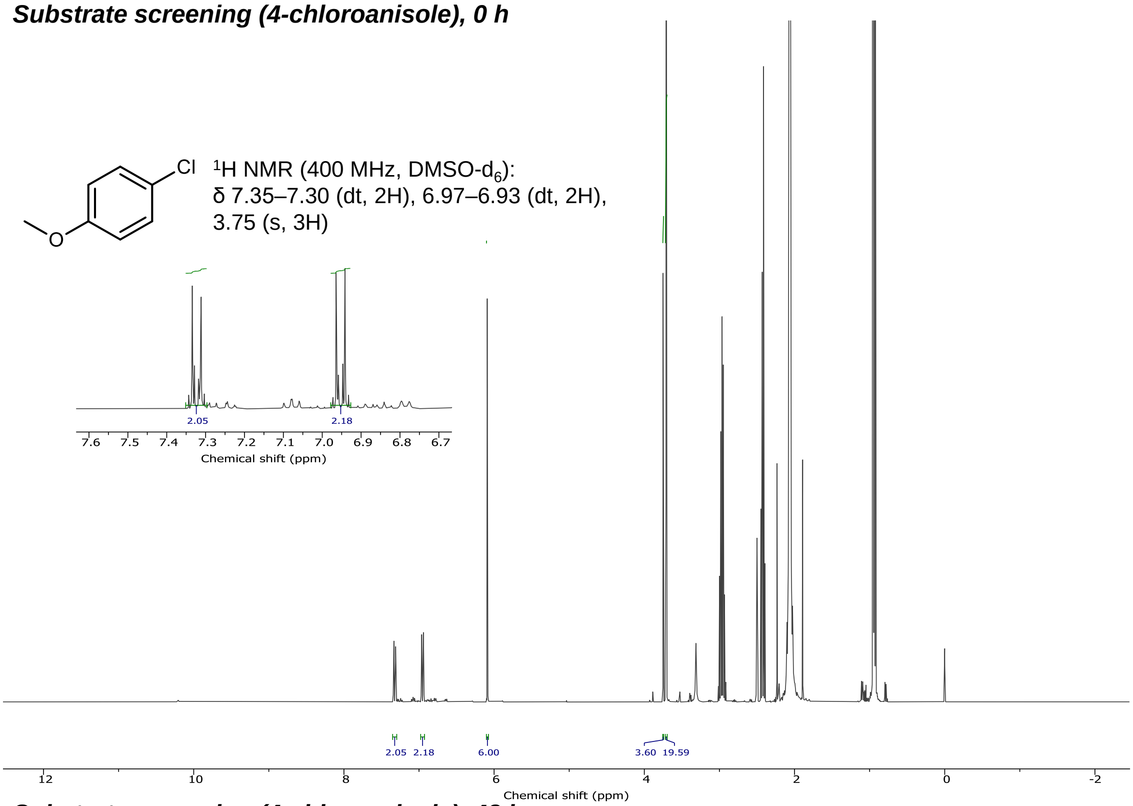

Substrate screening (4-chloroanisole), 48 h

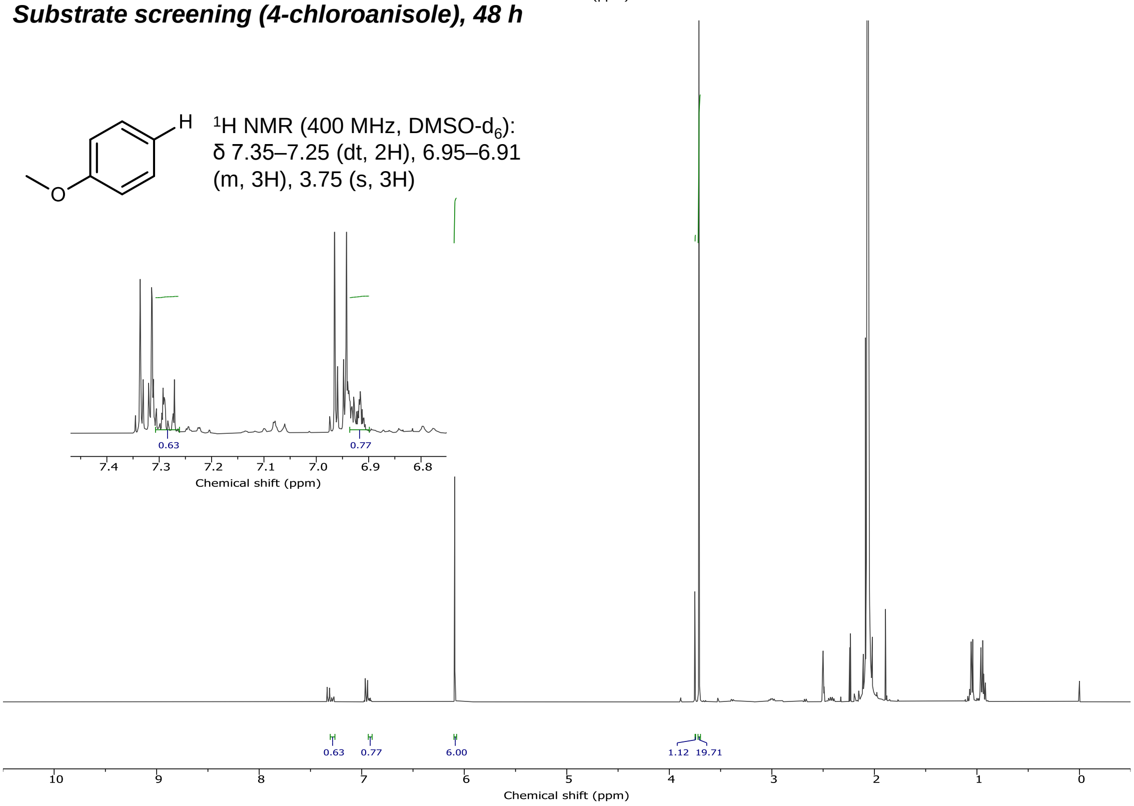

Supplementary Figure 120 ^{1}H NMR data for photoredox reductive dehalogenation for 4-chloroanisole (400 MHz, DMSO-d_6); 1,3,5-trimethoxybenzene: δ 6.09 (s, 3H), 3.71 (s, 9H).

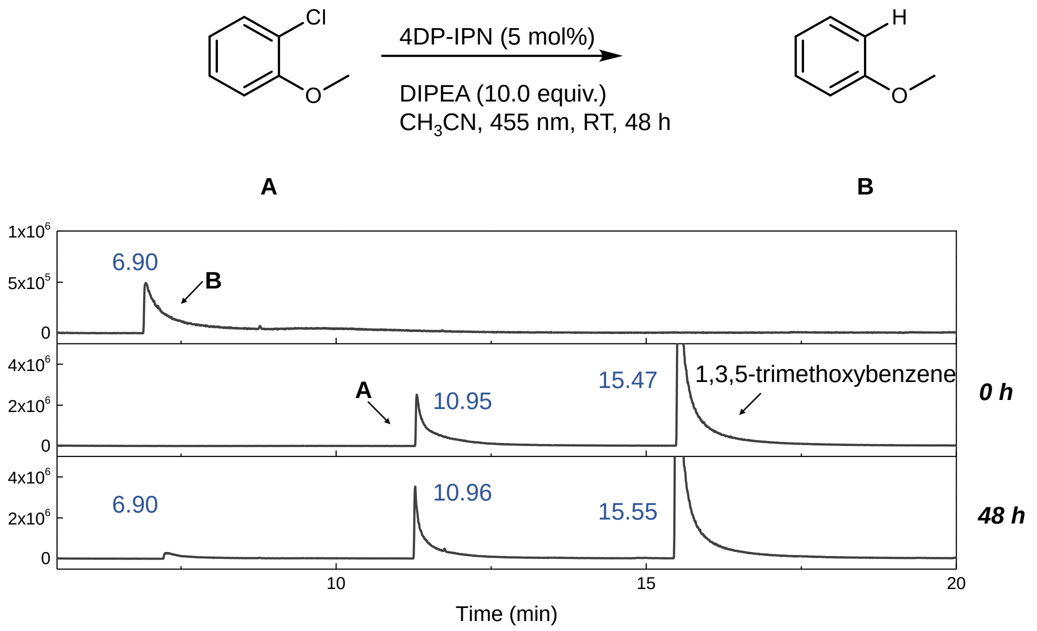

Supplementary Figure 121 GC-FID spectra for photoredox reductive dehalogenation for 2-chloroanisole.

Substrate screening (2-chloroanisole), 0 h

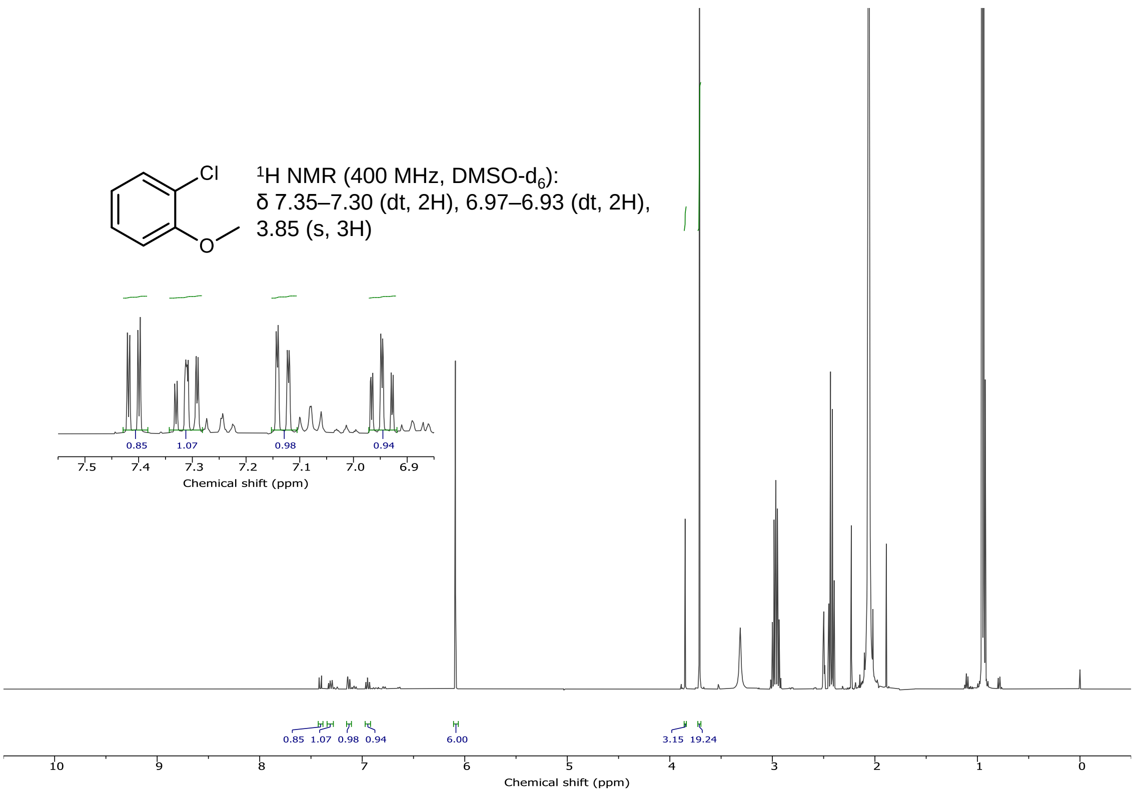

Substrate screening (2-chloroanisole), 48 h

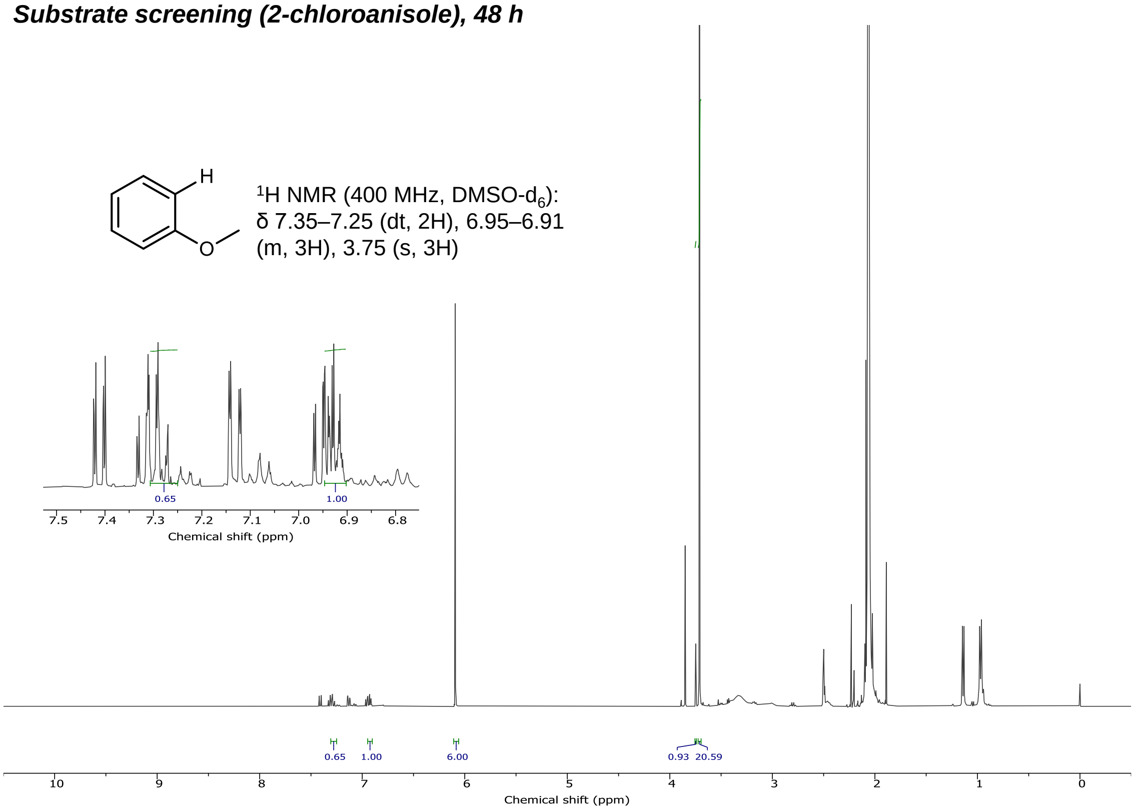

Supplementary Figure 122 ^{1}H NMR data for photoredox reductive dehalogenation for 2-chloroanisole (400 MHz, DMSO-d_6); 1,3,5-trimethoxybenzene: δ 6.09 (s, 3H), 3.71 (s, 9H). Yield was measured by ^{1}H NMR using 1,3,5-trimethoxybenzene as an internal standard.

Supplementary Note 15. Gram-scaled reaction of photoredox reductive dehalogenation

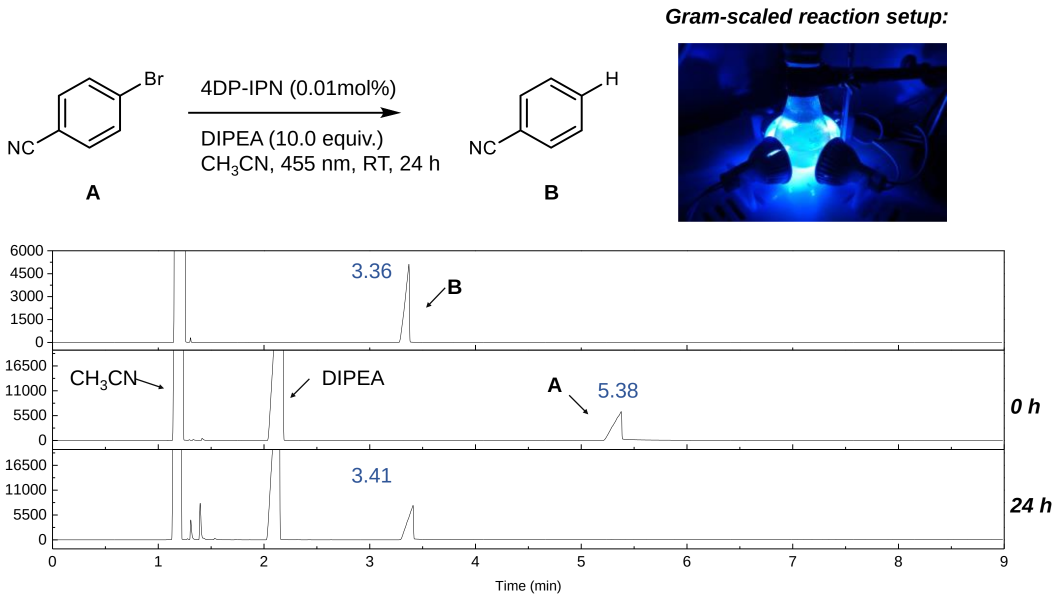

Supplementary Figure 123 Gram-scaled reaction was performed with 4-bromobenzonitrile (3.0 g, 16.48 mmol), DIPEA (10.0 equiv.) and 4DP-IPN (0.01 mol%) in CH_3CN (0.55 M) under illumination of four 3W 455 nm LEDs under ambient conditions without any degassing process in 250 mL one-neck round flask. Due to solubility issue, an internal standard was not involved. Yield was determined by conversion of starting material because of no side-reaction in GC or NMR data.

Gram-scaled reaction (4-bromobenzonitrile), 0 h

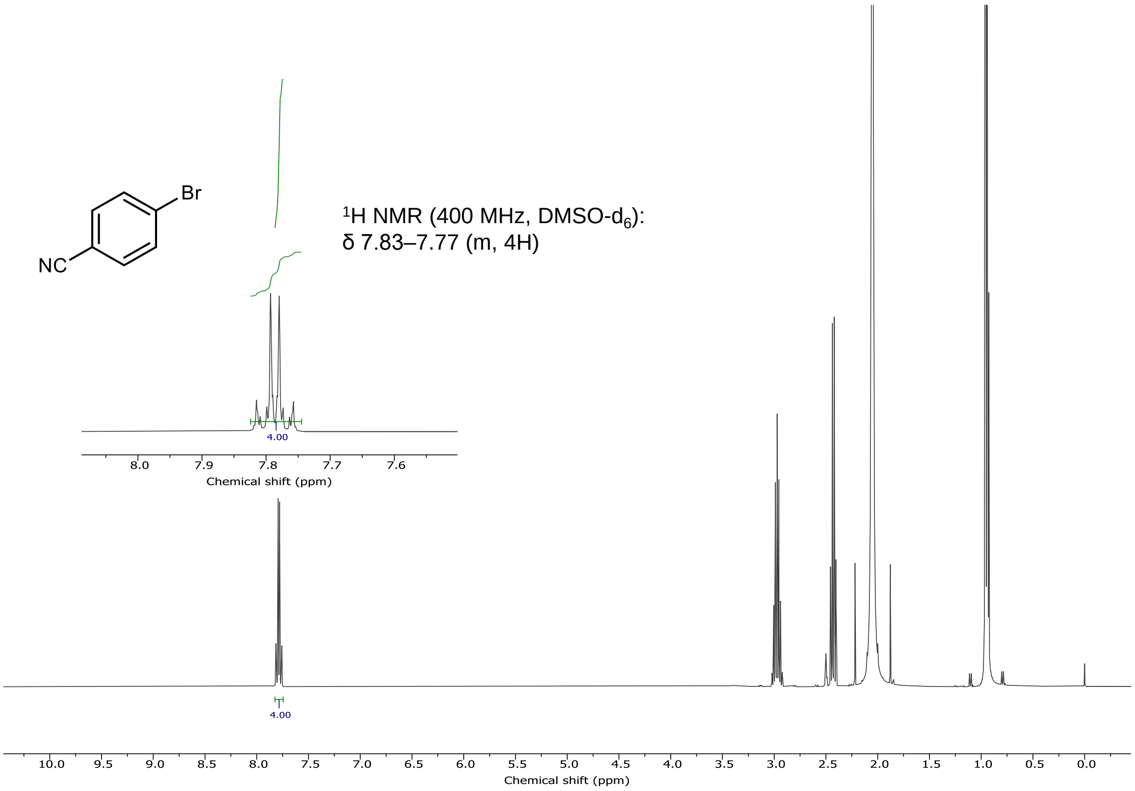

Gram-scaled reaction (4-bromobenzonitrile), 24 h

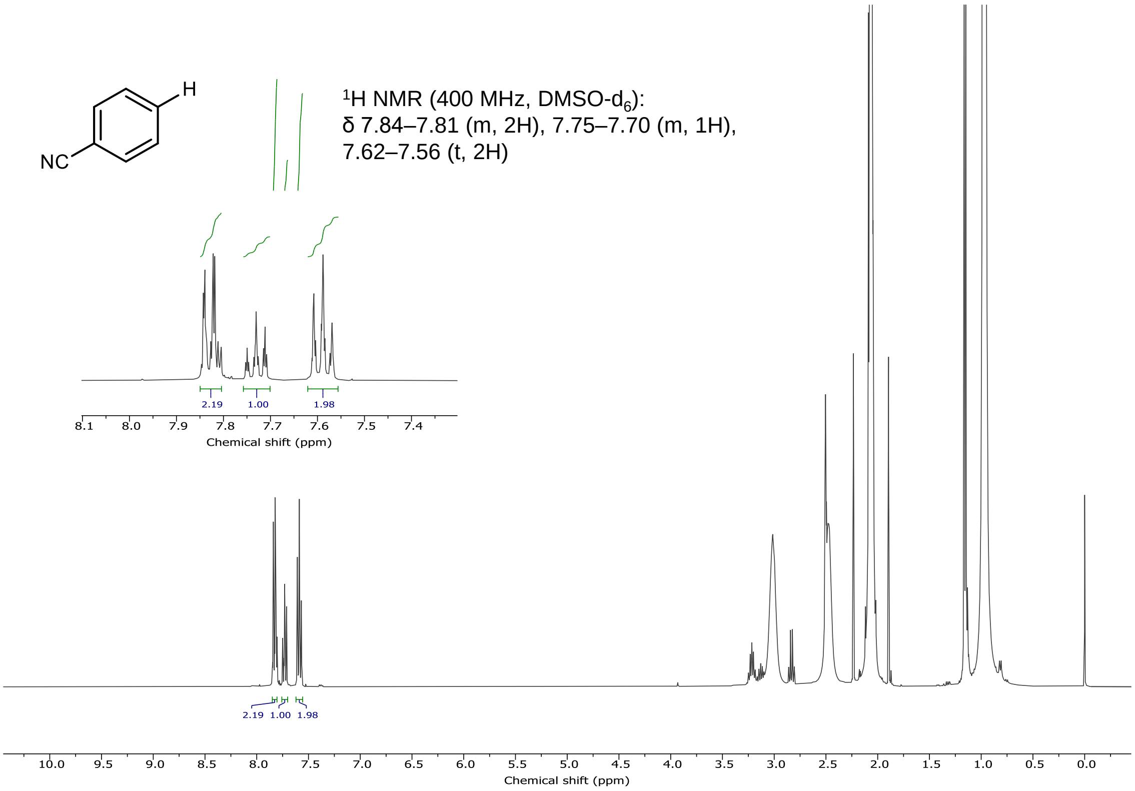

Supplementary Figure 124 ^{1}H NMR data for gram-scaled photoredox reductive dehalogenation for 4-bromobenzonitrile (400 MHz, DMSO-d$_6$).

Supplementary References

1. Singh, V. K. et al. Highly efficient organic photocatalysts discovered via a computer-aided-design strategy for visible-light-driven atom transfer radical polymerization. *Nat. Catal.* **1**, 794–804 (2018).
2. Grotjahn, S. & König, B. Photosubstitution in dicyanobenzene-based photocatalysts. *Org. Lett.* **23**, 3146–3150 (2021).
3. Zarwell, S. & Rück-Braun, K. Synthesis of an azobenzene-linker-conjugate with tetrahedrical shape. *Tetrahedron Lett.* **49**, 4020–4025 (2008).
4. Roscales, S. & CsákÿA. G. Synthesis of di(hetero)arylamines from nitrosoarenes and boronic acids: A general, mild, and transition-metal-free coupling. *Org. Lett.* **20**, 1667–1671 (2018).
5. Kataoka, N., Shelby, Q., Stambuli, J. P. & Hartwig, J. F. Air stable, sterically hindered ferrocenyl dialkylphosphines for palladium-catalyzed C-C, C-N, and C-O bond-forming cross-couplings. *J. Org. Chem.* **67**, 5553–5566 (2002).
6. Maiti, D., Fors, B. P., Henderson, J. L., Nakamura, Y. & Buchwald, S. L. Palladium-catalyzed coupling of functionalized primary and secondary amines with aryl and heteroaryl halides: two ligands suffice in most cases. *Chem. Sci.* **2**, 57–68 (2011).
7. Murase, T. & Fujita, M. Highly blue luminescent triazine-amine conjugated oligomers. *J. Org. Chem.* **70**, 9269–9278 (2005).
8. Marion Garreau, Franck Le Vaillant, and Jerome Waser, C-terminal bioconjugation of peptides through photoredox catalyzed decarboxylative alkynylation, *Angew. Chem. Int. Ed.* **58**, 8182 –8186 (2019).
9. Kretzschmar, A. et al. Development of thermally activated delayed fluorescence materials with shortened emissive lifetimes, *J. Org. Chem.* **80**, 9126−9131 (2015).
10. Etherington, M. K. et al. Persistent dimer emission in thermally activated delayed fluorescence materials *J. Phys. Chem. C.* **123**, 11109−11117 (2019).
11. Speckmeier, E., Fischer, T. G. & Zeitler, K. A toolbox approach to construct broadly applicable metal-free catalysts for photoredox chemistry: Deliberate tuning of redox potentials and importance of halogens in donor-acceptor cyanoarenes. *J. Am. Chem. Soc.* **140**, 15353–15365 (2018).
12. Korth, H. G. & Mulder, P. Phenolic hydrogen transfer by molecular oxygen and hydroperoxyl radicals. Insights into the mechanism of the anthraquinone process. *J. Org. Chem.* **85**, 2560–2574 (2020).
13. Nguyen, J. D., D'Amato, E. M., Narayanam, J. M. R. & Stephenson, C. R. J. Engaging unactivated alkyl, alkenyl and aryl iodides in visible-light-mediated free radical reactions. *Nat. Chem.* **4**, 854–859 (2012).
14. Ghosh, I., Ghosh, T., Bardagi, J. I. & König, B. Reduction of aryl halides by consecutive visible light-induced electron transfer processes. *Science.* **346**, 725–728 (2014).
15. Discekici, E. H. *et al.* A highly reducing metal-free photoredox catalyst: Design and application in radical dehalogenations. *Chem. Commun.* **51**, 11705–11708 (2015).
16. Ghosh, I., Shaikh, R. S. & König, B. Sensitization-initiated electron transfer for photoredox catalysis. *Angew. Chem. Int. Ed.* **56**, 8544–8549 (2017).
17. Naumann, R., Lehmann, F. & Goez, M. Generating hydrated electrons for chemical syntheses by using a green

light-emitting diode (LED). *Angew. Chem. Int. Ed.* **57**, 1078–1081 (2018).

18. Christoph Kerzig, O. S. W. Sensitized triplet–triplet annihilation upconversion in water and its application to photochemical transformations. *Chem. Sci.* **9**, 6670 (2018).
19. Ding, T. H., Qu, J. P. & Kang, Y. B. Visible-light-induced, base-promoted transition-metal-free dehalogenation of aryl fluorides, chlorides, bromides, and iodides. *Org. Lett.* **22**, 3084–3088 (2020).
20. MacKenzie, I. A. *et al.* Discovery and characterization of an acridine radical photoreductant. *Nature* **580**, 76–80 (2020).
21. Constantin, T. *et al.* Aminoalkyl radicals as halogen-atom transfer agents for activation of alkyl and aryl halides. *Science* **367**, 1021–1026 (2020).
22. Cowper, N. G. W., Chernowsky, C. P., Williams, O. P. & Wickens, Z. K. Potent reductants via electron-primed photoredox catalysis: unlocking aryl chlorides for radical coupling. *J. Am. Chem. Soc.* **142**, 2093–2099 (2020).
23. Romero, N. A. & Nicewicz, D. A. Organic photoredox catalysis. *Chem. Rev.* **116**, 10075–10166 (2016).
24. Koike, T. & Akita, M. Visible-light radical reaction designed by Ru- and Ir-based photoredox catalysis. *Inorg. Chem. Front.* **1**, 562–576 (2014).
25. Würth, C., Grabolle, M., Pauli, J., Spieles, M. & Resch-Genger, U. Comparison of methods and achievable uncertainties for the relative and absolute measurement of photoluminescence quantum yields. *Anal. Chem.* **83**, 3431–3439 (2011).
26. Lee, Y. *et al.* A water-soluble organic photocatalyst discovered for highly efficient additive-free visible-light-driven grafting of polymers from proteins at ambient and aqueous environments. *Adv. Mater.* **2108446**, 2108446 (2022).
27. Gierschner, J. *et al.* Luminescence in crystalline organic Materials: from molecules to molecular solids. *Adv. Opt. Mater.* **9**, 1–46 (2021).
28. Shi, J. *et al.* Solid state luminescence enhancement in π-conjugated materials: unraveling the mechanism beyond the framework of AIE/AIEE. *J. Phys. Chem. C* **121**, 23166–23183 (2017).
29. Gierschner, J., Cornil, J. & Egelhaaf, H. J. Optical bandgaps of π-conjugated organic materials at the polymer limit: Experiment and theory. *Adv. Mater.* **19**, 173–191 (2007).
30. Kohtani, S., Mori, M., Yoshioka, E. & Miyabe, H. Photohydrogenation of acetophenone using coumarin dye-sensitized titanium dioxide under visible light irradiation. *Catalysts* **5**, 1417–1424 (2015).
31. Adenier, A., Chehimi, M. M., Gallardo, I., Pinson, J. & Vilà, N. Electrochemical oxidation of aliphatic amines and their attachment to carbon and metal surfaces. *Langmuir* **20**, 8243–8253 (2004).
32. Furukawa, T., Nakanotani, H., Inoue, M. & Adachi, C. Dual enhancement of electroluminescence efficiency and operational stability by rapid upconversion of triplet excitons in OLEDs. *Sci. Rep.* **5**, 8429 (2015).
33. Song, Y. *et al.* Organic photocatalyst for ppm-level visible-light-driven reversible addition-fragmentation chain-transfer (raft) polymerization with excellent oxygen tolerance. *Macromolecules* **52**, 5538–5545 (2019).
34. Shen, Z., Procházka, R., Daub, J., Fritz, N., Acar, N. & Schneider, S. Towards modelling light processes of blue-light Photoreceptors. Pyrene-isoalloxazine (flavin)-phenothiazine triad: Electrochemical, photophysical, investigations and quantum chemical calculations. *Phys. Chem. Chem. Phys.* **5**, 3257–3269 (2003).
35. Yan, Q., Yue, K., Yu, C. & Zhao, D. Oligo- and polyfluorene-tethered *fac*-Ir(ppy)$_3$: Substitution effects.

Macromolecules **43**, 8479–8487 (2010).

36. Hedley, G. J., Ruseckas, A. & Samuel, I. D. W. Ultrafast luminescence in Ir(ppy)$_3$. *Chem. Phys. Lett.* **450**, 292–296 (2008).
37. Deronzier, A. Photophysical properties of soluble polypyrrole - polypyridyl - ruthenium (II) complexes. *J. Phys. Chem. B* **105,** 4801–4809 (2001).
38. Costentin, C., Robert, M. & Savéant, J. M. Fragmentation of aryl halide π anion radicals. Bending of the cleaving bond and activation vs driving force relationships. *J. Am. Chem. Soc.* **126**, 16051–16057 (2004).
39. Pan, Y. *et al.* Graphene oxide and Rose Bengal: oxidative C–H functionalisation of tertiary amines using visible light. *Green Chem.* **13**, 3341–3344 (2011).
40. Baciocchi, E., Del Giacco, T. & Lapi, A. Quenching of singlet oxygen by tertiary aliphatic amines. Structural effects on rates and products. *Helv. Chim. Acta* **89**, 2273–2280 (2006).
41. Maafi, M. & Brown, R. G. The kinetic model for AB(1φ) systems. A closed-form integration of the differential equation with a variable photokinetic factor. *J. Photochem. Photobiol. A Chem.* **187**, 319–324 (2007).
42. Watts, D. C. Reaction kinetics and mechanics in photo-polymerised networks. *Dent. Mater.* **21**, 27–35 (2005).
43. Pause, L., Robert, M. & Savéant, J. M. Can single-electron transfer break an aromatic carbon–heteroatom bond in one step? A novel example of transition between stepwise and concerted mechanisms in the reduction of aromatic iodides. *J. Am. Chem. Soc.* **121**, 7158–7159 (1999).
44. Giovanna Battistuzzi Gavioli, Marco Borsari, C. F. Theoretical study of the electroreduction of halogenated aromatic compounds. Part 2.—Bromine and chlorine derivatives in different organic solvents. *J. Chem. Soc. Faraday Trans.* **89**, 3931–3939 (1993).
45. Costentin, C., Robert, M. & Savéant, J. M. Electron transfer and bond breaking: Recent advances. *Chem. Phys.* **324**, 40–56 (2006).
46. Houser, K. J., Bartak, D. E. & Hawley, M. D. Electrochemical studies of the formation and decomposition of the fluorobenzonitrile radical anions. *J. Am. Chem. Soc.* **95**, 6033–6040 (1973).
47. Vandewal, K., Benduhn, J. & Nikolis, V. C. How to determine optical gaps and voltage losses in organic photovoltaic materials. *Sustainable Energy & Fuels* **2** 538–544 (2018).
48. Pause, L., Robert, M. & Savéant, J. M. Stepwise and concerted pathways in photoinduced and thermal electron-transfer/bond-breaking reactions. Experimental illustration of similarities and contrasts. *J. Am. Chem. Soc.* **123**, 4886–4895 (2001).
49. Pan, X. *et al*. Mechanism of photoinduced metal-free atom transfer radical polymerization: experimental and computational studies. *J. Am. Chem. Soc.* **138**, 2411–2425 (2016).
50. Franco, C. & Olmsted, J. Photochemical determination of the solubility of oxygen in various media. *Talanta* **37**, 905–909 (1990).
51. C. Baleizão & M. N. Berberan-Santos. Thermally activated delayed fluorescence as a cycling process between excited singlet and triplet states: Application to the fullerenes. *J. Chem. Phys.* **126**, 204510 (2007).
52. Cai, S. *et al.* Visible-light-promoted C-C bond cleavage: photocatalytic generation of iminium ions and amino radicals. *Angew. Chem. Int. Ed.* **51**, 8050–8053 (2012).

53. Beatty, J. W. & Stephenson, C. R. J. Amine functionalization via oxidative photoredox catalysis: Methodology development and complex molecule synthesis. *Acc. Chem. Res.* **48**, 1474–1484 (2015).
54. Wang, J. & Zheng, N. The cleavage of a C-C bond in cyclobutylanilines by visible-light photoredox catalysis: Development of a [4+2] annulation method. *Angew. Chem. Int. Ed.* **54**, 11424–11427 (2015).
55. Wang, Q. & Zheng, N. A photocatalyzed synthesis of naphthalenes by using aniline as a traceless directing group in [4 + 2] annulation of amino-benzocyclobutenes with alkynes. *ACS Catal.* **7**, 4197–4201 (2017).
56. Staveness, D. *et al.* Providing a new aniline bioisostere through the photochemical production of 1-aminonorbornanes. *Chem* **5**, 215–226 (2019).
